# Supplementary material for: Coordination of metal center biogenesis in human cytochrome c oxidase
Source: Nat Commun. 2022 Jun 24;13:3615. doi: 10.1038/s41467-022-31413-1 (PMC9232578; doi:10.1038/s41467-022-31413-1)

Supplementary Materials for

Coordination of metal center biogenesis in human cytochrome *c* oxidase

Eva Nývltová, Jonathan V. Dietz, Javier Seravalli, Oleh Khalimonchuk, and Antoni Barrientos

Correspondence to: abarrientos@med.miami.edu

This PDF file includes:

Supplementary Figures 1 to 12
Supplementary Tables 1 to 3

Other Supplementary Materials for this manuscript include the following:

Source data files

Supplementary Figures

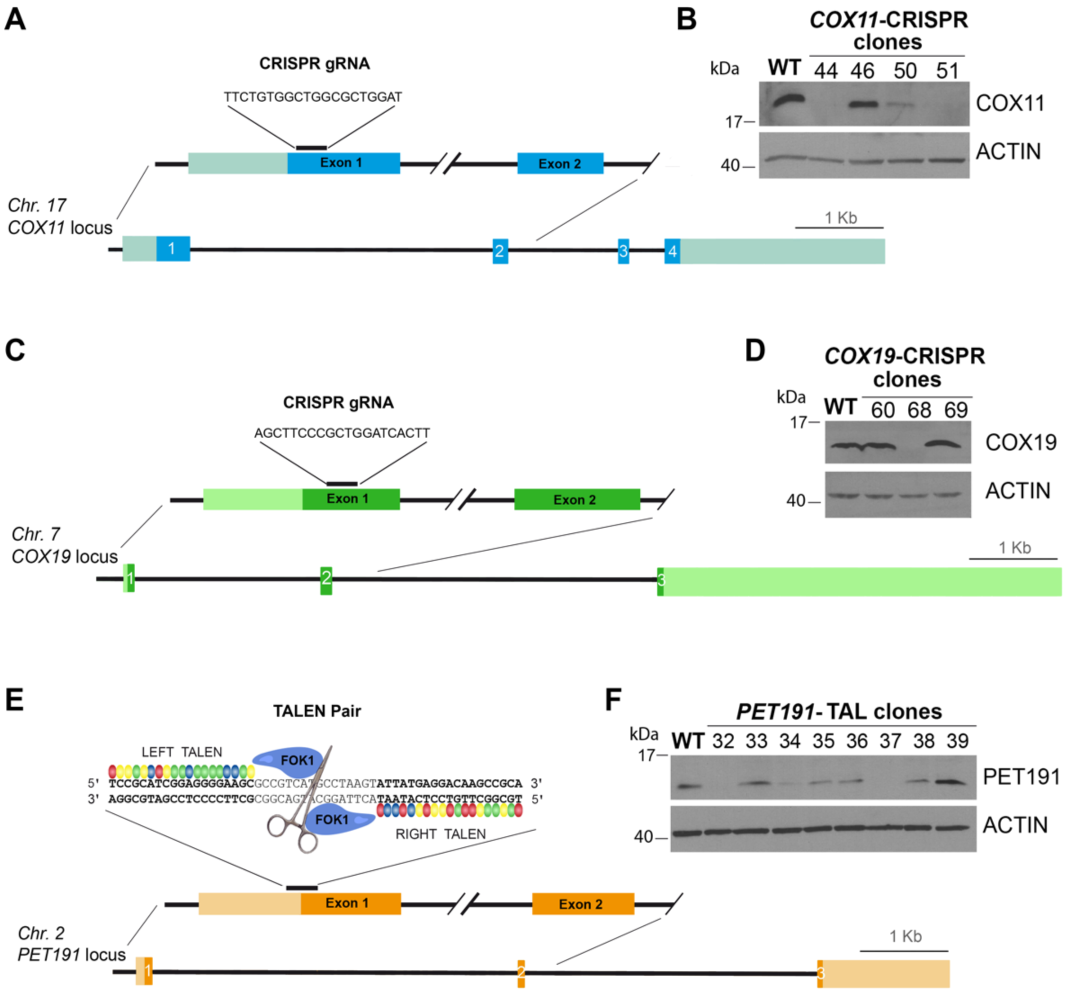

G

KO Clone	Genotype	DNA	Protein
***COX11*- KO44**	C. Hetero	c.42_82insATTCAGCTCCGGTTCCCAACGATCA AGGCGAGTTACATGT/c.57_68del/ c.68_75del	p.Phe14 fs/ p.Trp19 fs/p.Pro22
***COX11*- KO51**	C. Hetero	c.18_19del/c.30_41del/c.19_29del	p. Pro6 fs /p.Glu10 fs/p.Pro6 fs
***COX19*-KO68**	C. Hetero	c.71_88del/c.70_78del/c.65_75del	p.Pro24 fs/ p. Pro23 fs/ p.Ser21 fs
***PET191*-KO32**	Mut Homo	c.4_11del	p.Met1fs
***PET191*-KO37**	Mut Homo	c.-3_5del	p.0

Supplementary Fig. 1: Generation of *COX11*-KO, *COX19*-KO and *PET191*-KO cell lines in the HEK293T background.
(**A, C,** and **E**) Schematic showing the location of the target sites in the *COX11, COX19*, and *PET191* loci for the CRISPR guide RNAs or TALEN constructs used to knockout each gene. Chr., chromosome. (**B, D,** and **F**) Screening of CRISPR or TAL clones by immunoblotting to identify cell lines knockout (KO) for *COX11, COX19* or *PET191*. Once the KO cell lines for each gene were identified, each was tested three additional times and frozen stocks were kept in liquid nitrogen. Each time that a frozen vial was thawed, the KO cell lines were equally tested by immunoblotting. (**G**) Table detailing the genotype of the generated HEK293T cell lines carrying edited *COX11, COX19* or *PET191* alleles. C. Hetero, compound heterozygous; Mut Homo, mutant homozygous. Source data for panels B, D, and F are provided as a Source Data file.

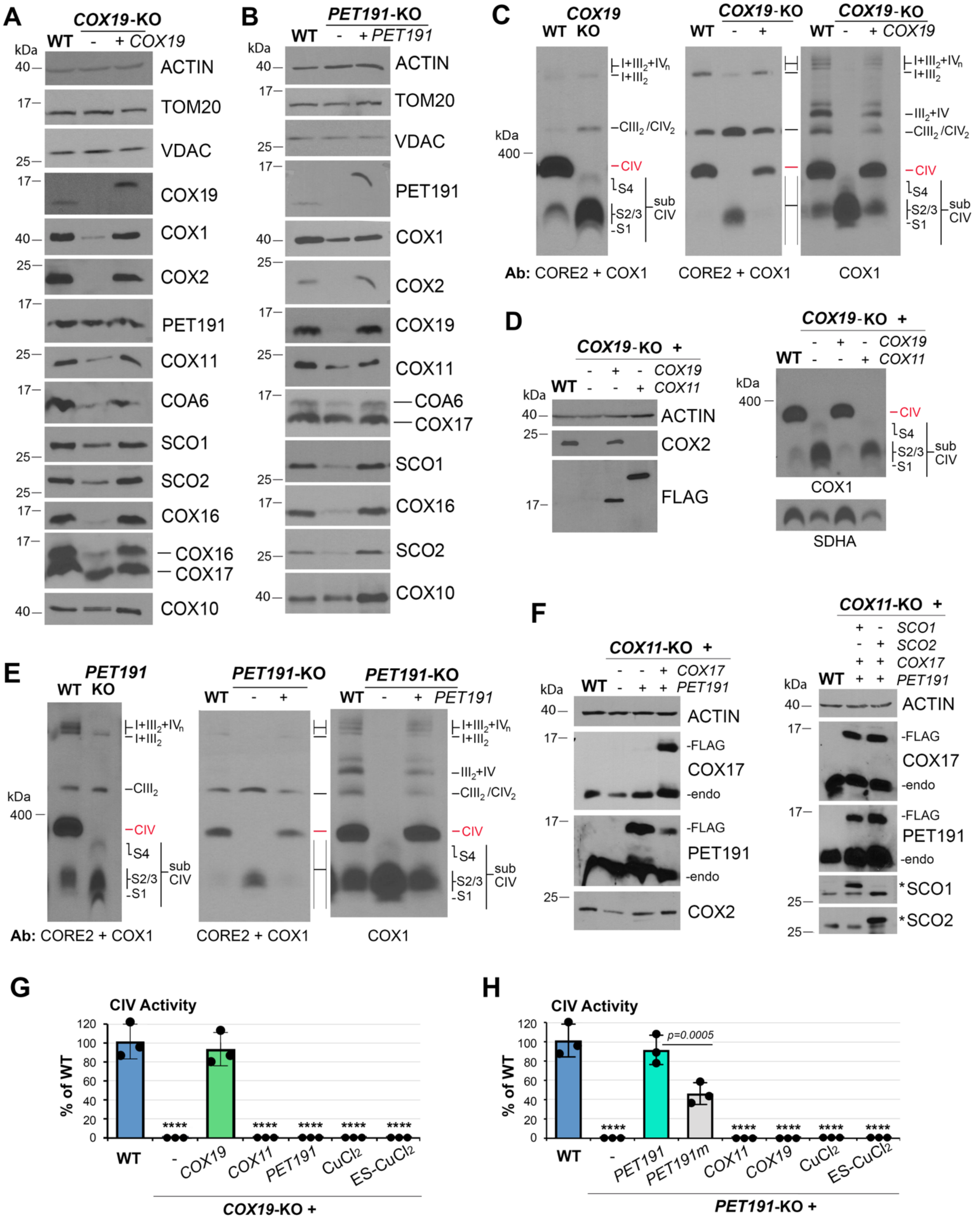

Supplementary Fig. 2: Steady state levels of mitochondrial respiratory chain complexes, subunits and assembly factors are altered in the *COX19*-KO, and *PET191*-KO cell lines.

(**A-B**) Steady-state levels of mitochondrial respiratory chain complex subunits and assembly factors in *COX19*-KO (**A**), or *PET191*-KO (**B**) cells reconstituted or not with the corresponding FLAG-tagged WT gene, assessed by immunoblotting. ACTIN served as the loading control. (**C**) Blue-Native (BN)-PAGE analysis of the steady state levels of respiratory complexes ($CIII_2$, CIV, CIV_2) and supercomplexes (III_2+CIV and I+III_2+CIV_n), as well as complex IV subcomplexes or assembly intermediates in *COX19*-KO cells reconstituted or not with a FLAG-tagged WT *COX19* gene. CIV subassemblies (sub CIV) are labeled

as S1-S3. S1 contains COX1-COX14-COA3-CMC1 and S2-S3 contain COX1-COX14-COA3-COX4-COX5a without or with assembly factors such as SURF1 or MITRAC7 [1]. S4 is a CIV subcomplex formed by the off-pathway joining of the COX1 and COX3 assembly modules when COX2 is absent or in limited amounts [2]. (**D**) SDS-PAGE (left panel) and BN-PAGE (right panel) analyses of respiratory complexes ($CIII_2$, CIV, CIV_2) and supercomplexes (III_2+CIV and I+III_2+CIV_n) in *COX19*-KO cells overexpressing *COX11*. (**E**) Blue-Native (BN)-PAGE analysis of the steady state levels of respiratory complexes and supercomplexes, as well as complex IV subcomplexes or assembly intermediates in *PET191*-KO cells reconstituted or not with a FLAG-tagged WT *PET191* gene. (**F**) Immunoblot analysis of the steady-state levels of the indicated proteins in HEK293T wild-type (WT) and *COX11*-KO cells transfected with constructs expressing FLAG-tagged PET191, alone or in combination with COX17, SCO1 or SCO2 as indicated. ACTIN was used as a loading control. Figures in panels A-F are representative of three independent repetitions with similar results. (**G**) Complex IV activity measured spectrophotometrically in *COX19*-KO cells reconstituted or not with FLAG-tagged *COX19* gene, overexpressing COX11 or PET191, or grown in the presence of exogenous copper. For copper supplementation assays, the complete medium was supplemented with (i) 1 mM $CuCl_2$, or (ii) 1 nM elesclomol (ES) + 1mM $CuCl_2$. (**H**) Complex IV activity measured as in panel G in *PET191*-KO cells reconstituted or not with FLAG-tagged WT *PET191* or mutant *PET191(C30A, C41A)* gene, overexpressing COX11 or COX19, or grown in the presence of exogenous copper. In panels G and H, the bars represent the average ± S.D. of three independent replicates. Black dots represent individual data points. Two-sided unpaired *t*-test, ****$p<0.0001$. Source data for panels A, B, D, and F-H are provided as a Source Data file.

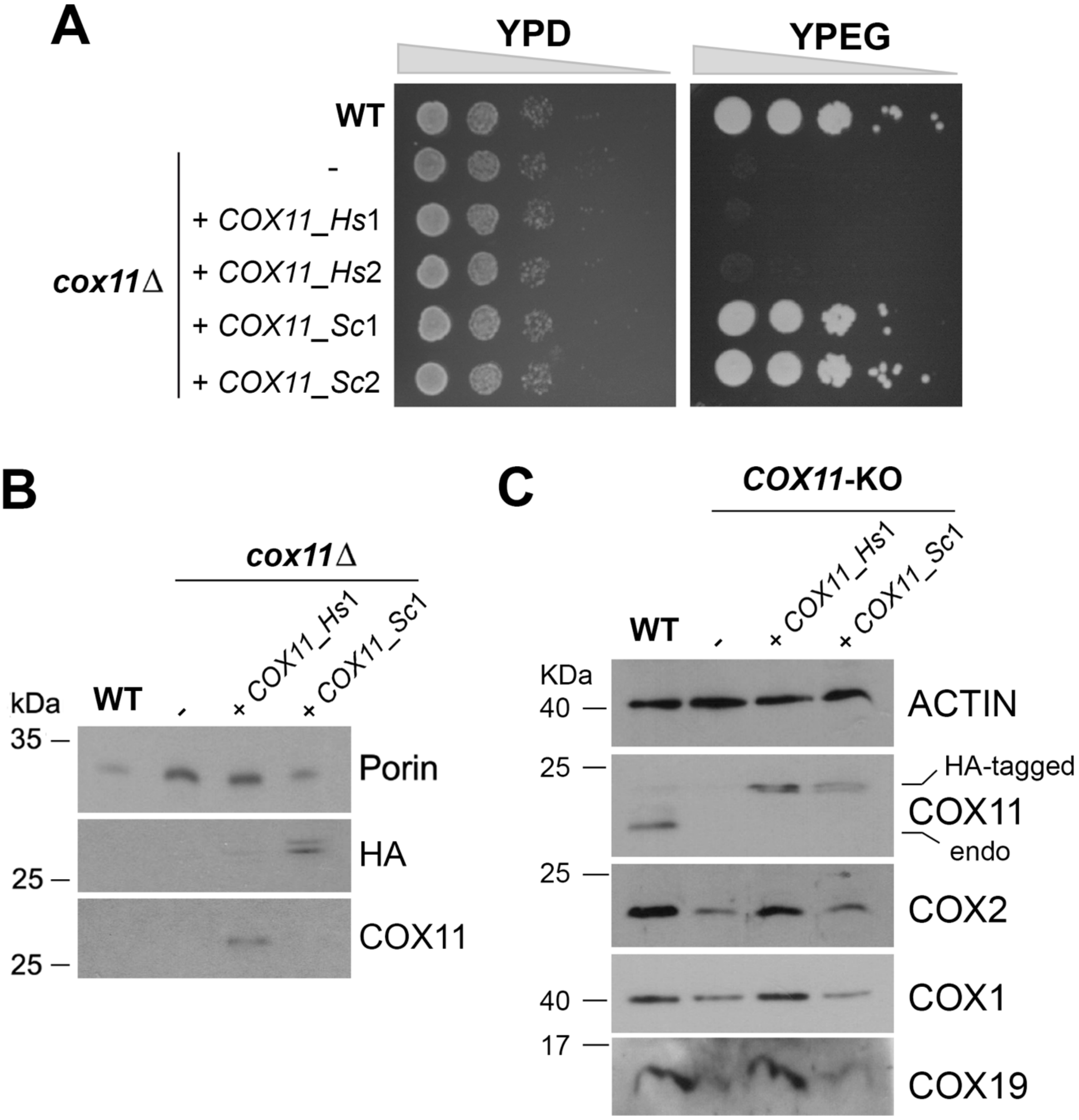

Supplementary Fig. 3: Lack of heterologous complementation of *Saccharomyces cerevisiae and human COX11*.

(**A**) Serial dilutions growth tests of *cox11*Δ cells overexpressing human (*COX11_Hs*) or HA-tagged yeast (*COX11_Sc*) COX11 in complete media containing glucose (YPD), or ethanol+glycerol (YPEG), as the carbon sources. Two different clones are presented in each case. (**B**) Immunoblot analysis in isolated mitochondria of the steady-state levels of HA-tagged yeast Cox11 or human *COX11* expressed in *cox11*Δ cells. Porin was used as a loading control. (**C**) Immunoblot analysis of human *COX11*-KO cell lines overexpressing HA-tagged human (*COX11_Hs*) or yeast (*COX11_Sc*) COX11. Figures in panels B-C are representative of three independent repetitions with similar results. Source data for panels B-C are provided as a Source Data file.

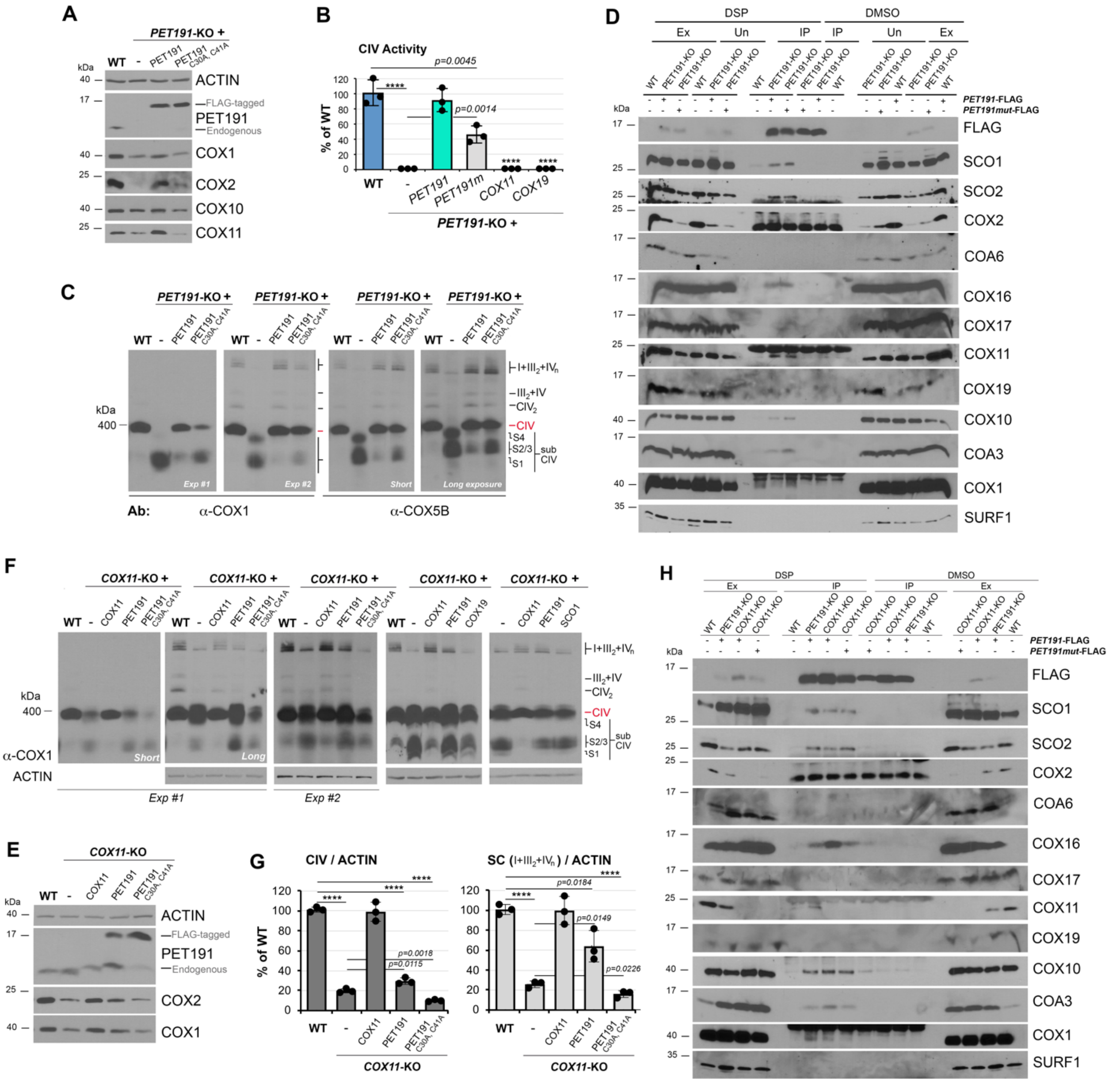

Supplementary Fig. 4: A double (C30A, C41A) mutation in PET191 partially impairs CcO assembly, but does not suppress the CcO assembly defect of *COX11*-KO cells despite having an interactome like that of WT PET191.

(**A** and **E**) Steady-state levels of PET191, COX1, COX10, and CcO subunits COX1 and COX2 in the indicated cell lines, assessed by immunoblotting. ACTIN served as the loading control. (**B**) CcO specific activity measured spectrophotometrically in frozen-thawed cells following the oxidation of exogenous reduced cytochrome *c*. The bars are the average ± S.D. of three independent replicates. Black dots represent individual data points. Two-sided unpaired *t*-test, ***p<0.0001. (**C** and **F**) Steady-state levels of complex IV subassemblies, holocomplex (CIV and CIV_2) and respiratory supercomplexes (III_2+CIV and I+III_2+CIV_n) in the indicated cell lines, separated by BN-PAGE and detected by immunoblotting with an antibody against COX1. ACTIN served as the loading control. In (F), short and long exposures of the same immunoblots are indicated. Two independent experiments are included to show the effect of the PET191 mutation on supercomplexes I+III_2+IV_n. (**G**) Quantification of the steady-state levels of mitochondrial SCs I+III_2+IV_n by densitometry of the images presented in panel (F). The values were normalized by the ACTIN levels and expressed as percentage of WT values. The bars are the average ± S.D. of at three independent replicates. Black dots represent individual data points. Two-sided unpaired *t*-test, ****p<0.0001. (**D** and **H**) Immunoprecipitation analysis of PET191-FLAG or PET191 mutant-FLAG in the indicated cellular backgrounds. Mitochondria were purified from each strain, treated with DSP or the vector DMSO, extracted with 0.4% DDM (n-dodecyl β-D-maltoside), incubated with anti-FLAG-conjugated protein A beads, and processed for immunoblot analyses to detect the indicated proteins. HEK293T cells without FLAG (WT) were used as negative control. Ex, extract; Un, unbound; IP, immunoprecipitate. Figures in panels A, D, E,

and H are representative of three independent repetitions with similar results. Source data for panels A, B, D, E, G, and H are provided as a Source Data file.

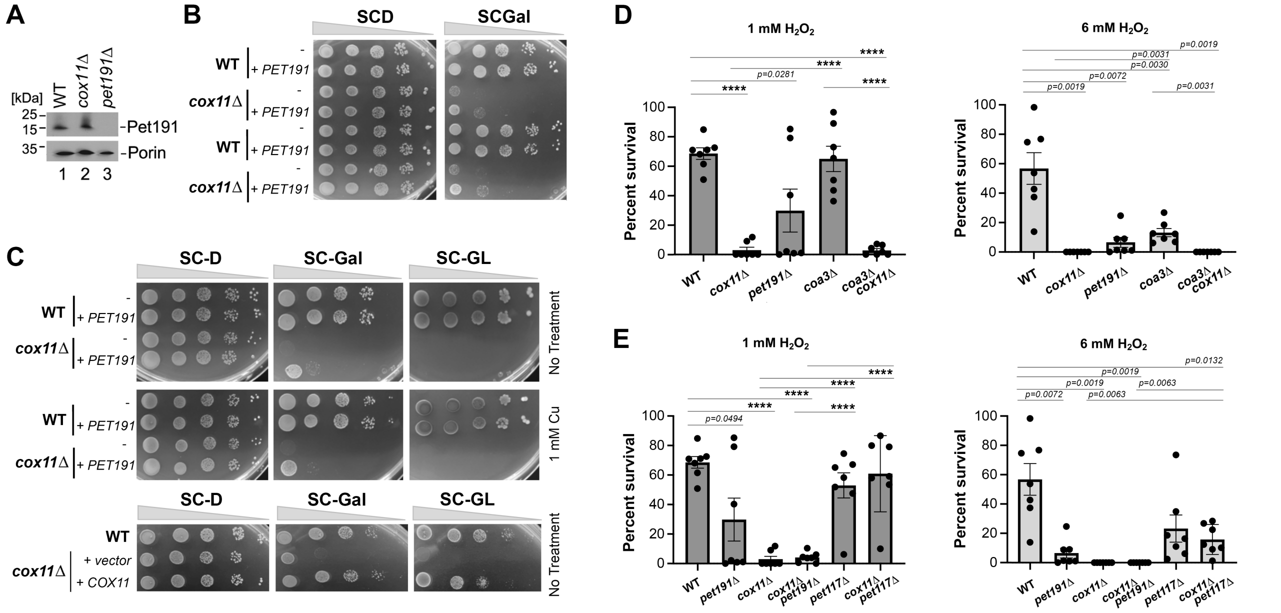

Supplementary Fig. 5: *Saccharomyces cerevisiae PET191* is a weak high-copy suppressor of *cox11*Δ mutant.

(A) Steady-state levels of Pet191 in mitochondria isolated from the indicated *S. cerevisiae* strains, assessed by immunoblotting with respective antibodies. Porin was used as the loading control. Figures in panel A are representative of three independent repetitions with similar results. Source data are provided as a Source Data file. (**B, C**) Serial dilutions growth tests of the indicated strains in synthetic media containing glucose (SCD), galactose (SCGal), or glycerol (GL) as the carbon source, with or without supplementation with 1 mM $CuCl_2$ (panel C). (**D-E**) Sensitivity of the indicated strains to hydrogen peroxide. The dominant negative effect of the *cox11*Δ mutation is shown in panel (**D**). The dominant positive effect of the *pet117*Δ mutation is shown in panel (**E**). Cells were cultured in YPD medium at 30 °C to mid-log phase and then normalized and acutely treated with 1 mM or 6 mM H_2O_2 for 1 h at 30 °C. Cultures were diluted and 300 cells were plated on YPD agar for assessment of colony forming units after 48 h incubation at 30 °C. The bars represent the average ± S.E.M. of seven independent replicates. Black dots represent individual data points. Two-sided paired *t*-test, ****p<0.0001.

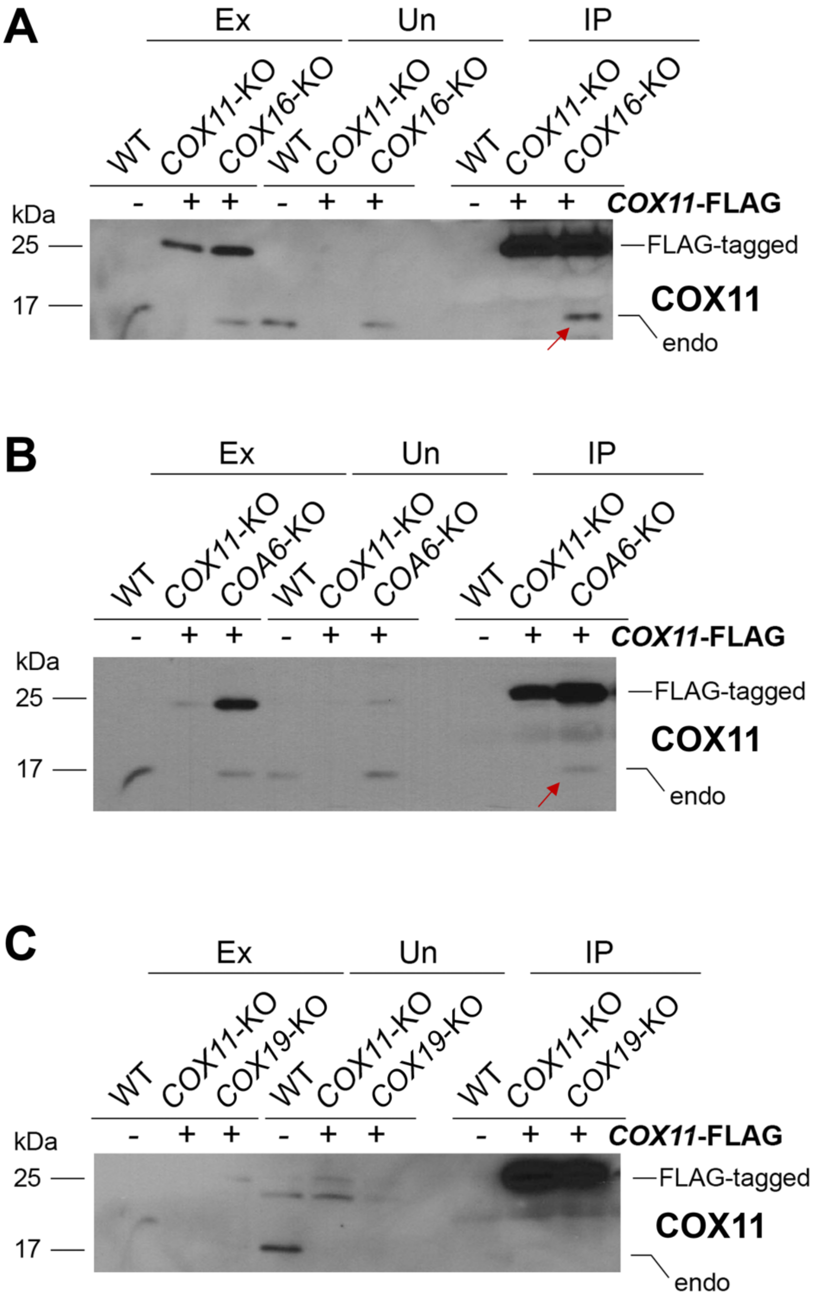

Supplementary Fig. 6: COX11 dimerization depends on COX19.

Co-IP of recombinant COX11-FLAG with endogenous COX11 in the indicated cell lines: (**A**) *COX16*-KO (B) *COA6*-KO, and (**C**) *COX19*-KO. Ex, extract; Un, unbound; IP, immunoprecipitate. Red arrows mark the coimmunoprecipitated endogenous COX11. Figures in panels A-C are representative of three independent repetitions with similar results.

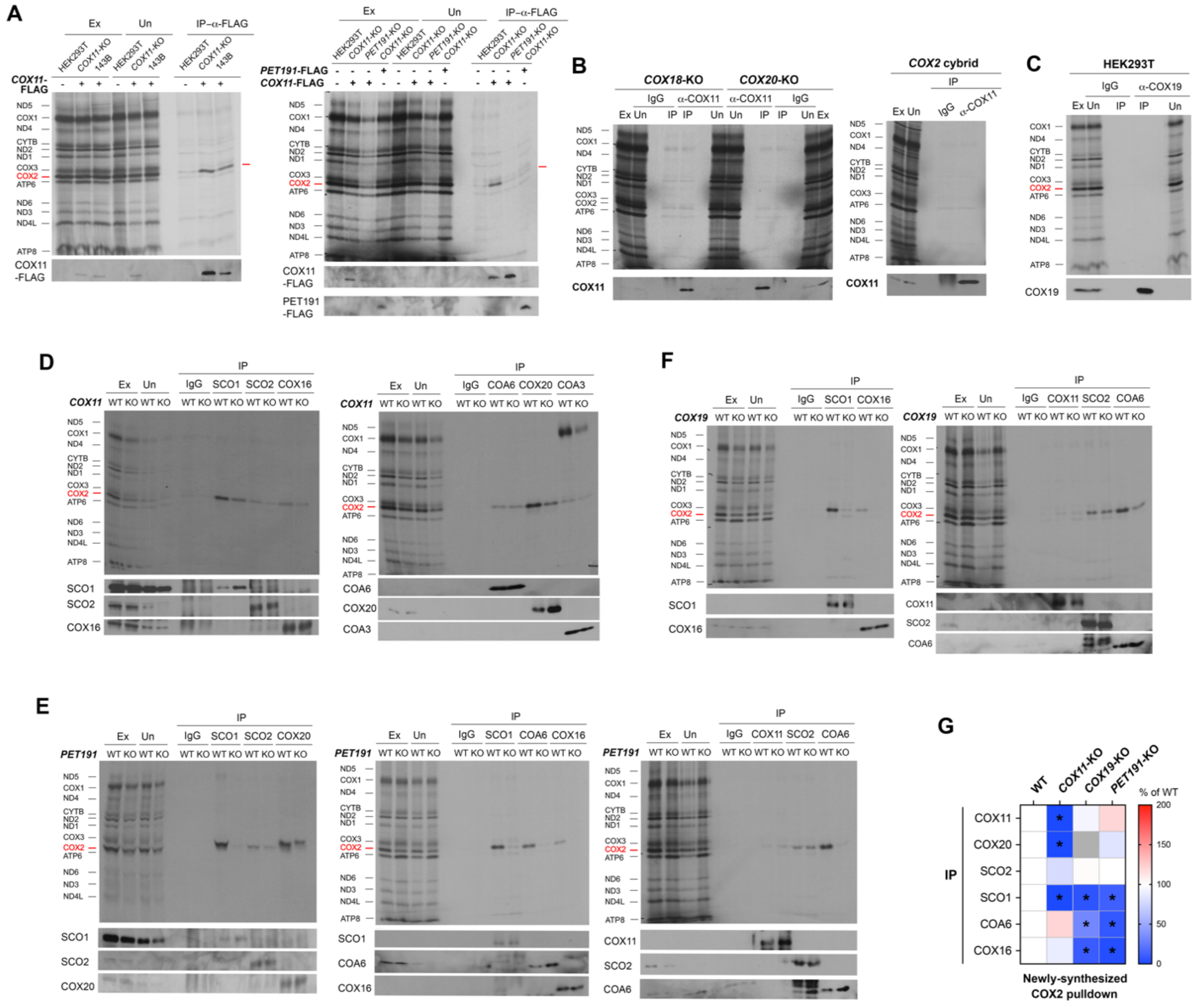

Supplementary Fig. 7: COX11 interacts with newly synthesized COX2 and accumulate in several partially overlapping high-mass complexes that partially overlap with those containing COX19 and PET191.

(**A**) Mitochondrial translation products were pulse-labeled with ^{35}S-methionine for 30 min in the presence of emetine to inhibit cytoplasmic protein synthesis in HEK293T or 143B cells (WT) and the indicated KO cell lines expressing FLAG-tagged version of COX11 or PET191. Whole-cell extracts were then used for immunoprecipitation (IP) using anti-FLAG-conjugated agarose beads. Equivalent amounts of total protein from extract (Ex), unbound (Un), and immunoprecipitate (IP) fractions were separated by SDS-PAGE on a 17.5% polyacrylamide gel, transferred to a nitrocellulose membrane, and analyzed by autoradiography. The lower panels are immunoblots of the same membrane probed with an anti-COX11 or anti-PET191 antibodies to detect the corresponding immunoprecipitated FLAG-tagged protein. (**B**) Similar experiment as in panel A but using HEK293T *COX18*-KO or *COX20*-KO cells, and 143B/COX2 cybrids. Whole-cell extracts were then used for immunoprecipitation using protein A-conjugated agarose beads and antibodies against the indicated proteins or IgG as a control. The lower panels are immunoblots of the same membrane probed with an anti-COX11 antibody to detect the immunoprecipitated COX11 protein. (**C**) Experiment performed as in panel B but using WT HEK293T cells. Whole-cell extracts were then used for immunoprecipitation using protein A-conjugated agarose beads and anti-COX19 antibody or IgG as a control. The lower panels are immunoblots of the same membrane probed with the anti-COX19 antibody to detect the immunoprecipitated COX19 protein. (**D**) Metabolic labeling with ^{35}S-methionine as in panel A in HEK293T and *COX11*-KO cells followed by IP with protein A-conjugated agarose beads and antibodies against the COX2 chaperones SCO1, SCO2, COX16, COA6, COX20, and the COX1 chaperone COA3. The lower panels are immunoblots of the same membrane probed with an anti-SCO1, anti-SCO2, anti-COX16, anti-COA6, anti-COX20, or anti-COA3 antibodies to detect the corresponding immunoprecipitated proteins. (**E**) As in panel C but using HEK293T and *PET191*-KO cells and antibodies against COX2 chaperones. The lower panels are immunoblots of the same membrane probed with an anti-SCO1, anti-SCO2, anti-COA6, anti-COX20, or anti-COX11 antibodies to detect the corresponding immunoprecipitated proteins. (**F**) As in

panel D but using HEK293T and *COX19*-KO cells. The lower panels are immunoblots of the same membrane probed with an anti-SCO1, anti-SCO2, anti-COX16, anti-COA6, anti-COX18, or anti-COX11 antibodies to detect the corresponding immunoprecipitated proteins. (**G**) The heat map shows the average quantification of immunoprecipitated COX2 relative to the levels of immunoprecipitated protein and expressed as percentage of wild-type, across the cell lines of interest, of three independent experiments. Grey squares indicate "not tested". The values were compared with the wild-type. Two-sided paired *t*-test. *$p<0.05$. Figures in panels A-F are representative of three independent repetitions with similar results. Source data for panels A-G are provided as a Source Data file.

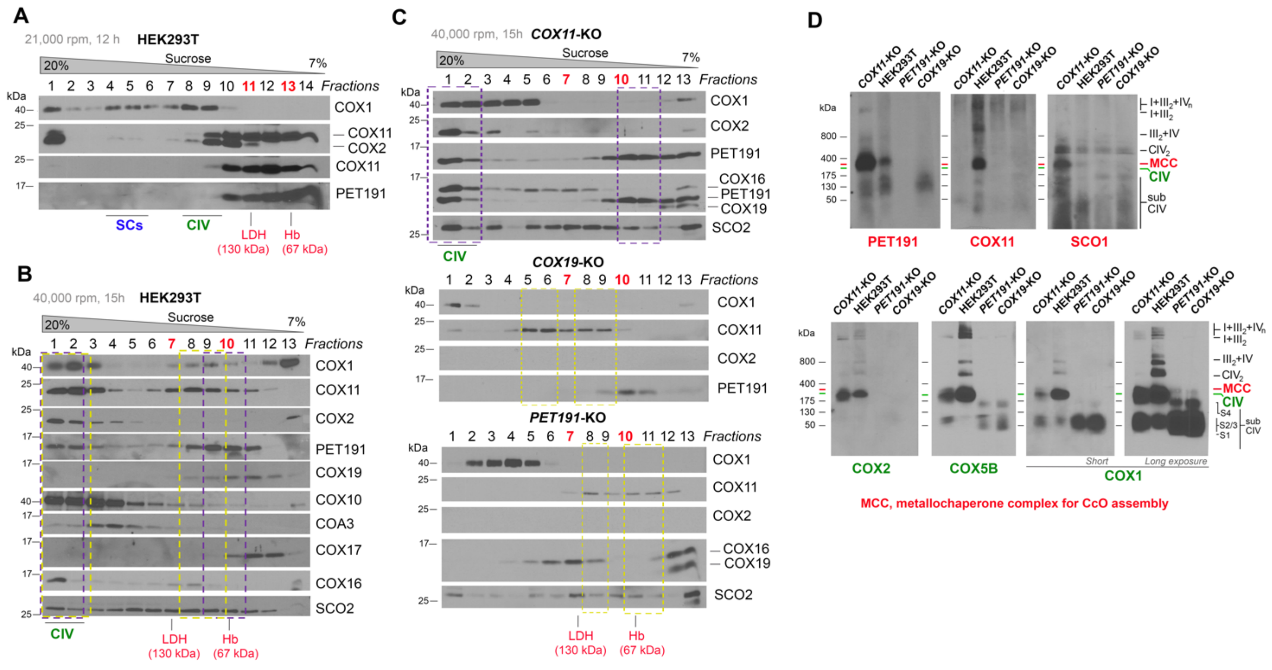

Supplementary Fig. 8: COX11, COX19 and PET191 accumulate in several partially overlapping high-mass complexes. (**A-C**) Sedimentation analyses of CcO metallochaperones in 7-20% linear sucrose gradients using mitochondrial extracts prepared in the presence of 1% digitonin. Two different centrifugation conditions on a 55 Ti swinging-bucket Beckman rotor were used: (**A**) 38,000 x *g* for 12 h or (**B-C**) 150,000 x *g* for 15 h. Lactate dehydrogenase (LDH) and hemoglobin (Hb) were used as molecular weight standards to calibrate the gradients. Following fractionation, the samples were analyzed by immunoblotting. Yellow and purple lines indicated the fractions in which COX11 or PET191 peak, respectively. SCs, respiratory chain supercomplexes; CIV, complex IV. (**D**) Mitochondrial extracts prepared as in panels B-C were analyzed by BN-PAGE and immunoblotting with antibodies against the indicated proteins. Figures in panels A-D are representative of three independent repetitions with similar results. Source data for panels A-C are provided as a Source Data file.

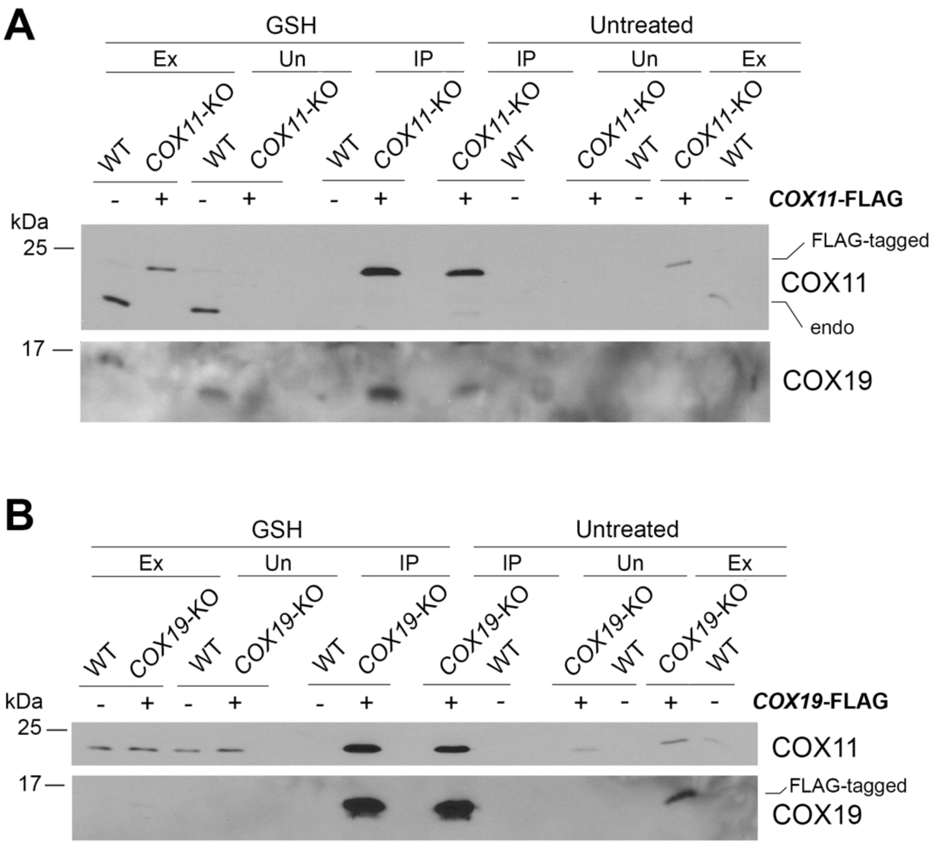

Supplementary Fig. 9: The interaction COX11-COX19 does not depend on the redox environment. Immunoblot analyses of reciprocal immunoprecipitations of COX11 and COX19 in mitochondria isolated from (**A**) *COX11*-KO cells reconstituted with COX11-FLAG or (**B**) *COX19*-KO cells expressing COX19-FLAG. The proteins were extracted from mitochondria treated or not with 5mM reduced glutathione (GSH), and the proteins immunoprecipitated with anti-FLAG-conjugated sepharose beads. Ex, extract; Un, unbound; IP, immunoprecipitate. Figures in panels A-B are representative of three independent repetitions with similar results. Source data for panels A, and B are provided as a Source Data file.

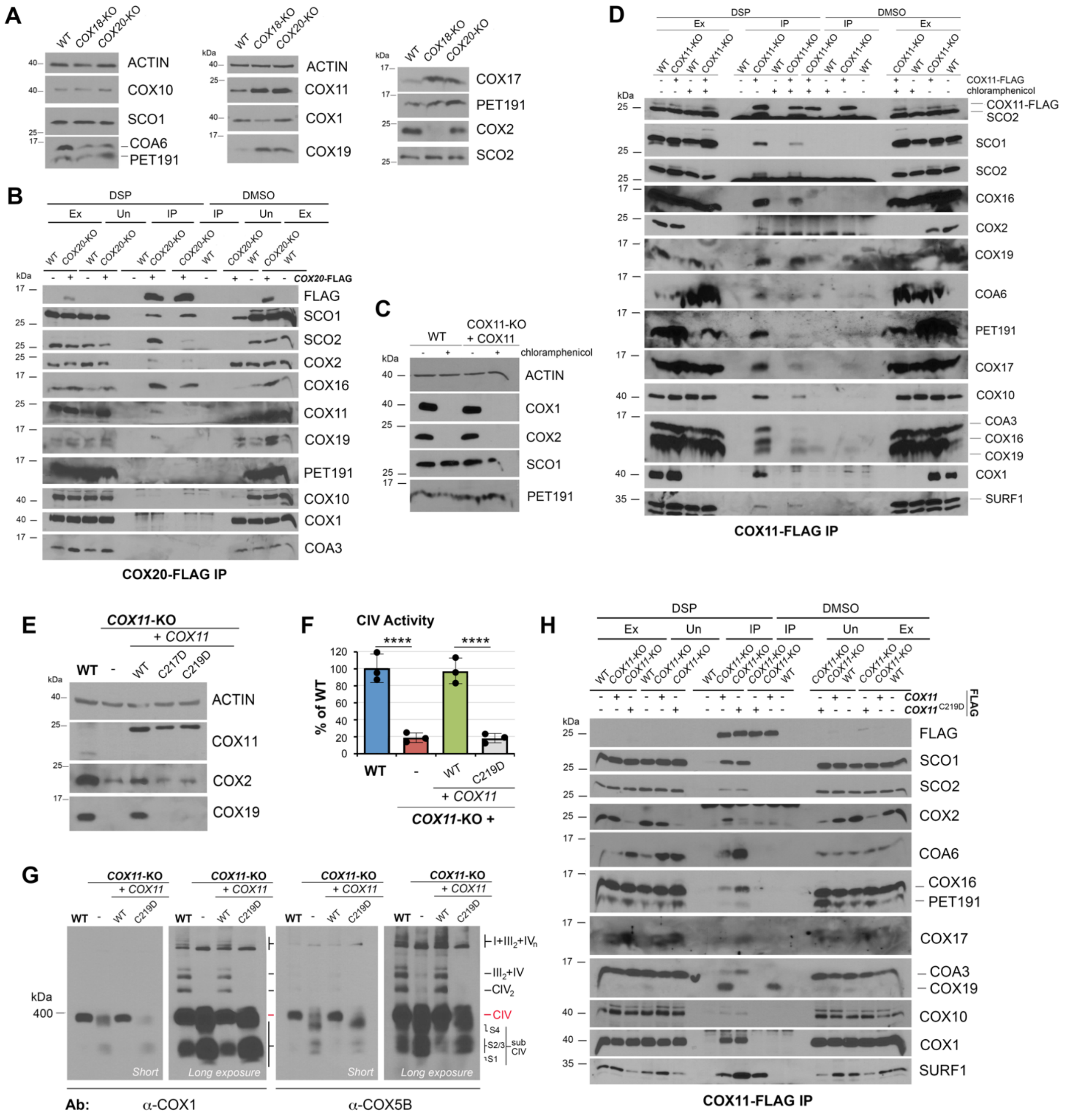

Supplementary Fig. 10: Details of the interaction network of COX11.

(**A-B**) COX20 interacts with COX11 but it does not interact with COX1, COA3, COX10, or PET191. (**A**) Steady-state levels of the mitochondrial respiratory chain complex subunits and assembly factors in the indicated cell lines, assessed by immunoblotting with respective antibodies. ACTIN served as the loading control. (**B**) Immunoprecipitation of COX20-FLAG in the indicated cellular backgrounds analyzed as described in Figure S4. Ex, extract; Un, unbound; IP, immunoprecipitate. (**C-D**) COX11 interacts with CcO metallochaperones and other complex IV assembly factors in the absence of COX1 and COX2. (**C**) Steady-state levels of mitochondrial respiratory chain complex subunits COX1 and COX2 and the indicated assembly factors in cells incubated with or without 200 µg/ml chloramphenicol for 10 days. ACTIN was used as the loading control. (**D**) Immunoprecipitation of COX11-FLAG in the indicated cellular backgrounds analyzed as described in panel B. (**E-H**) Cysteine mutant (C217D or C219D) variants of COX11 do not support CcO assembly and fail to interact with COX19. (**E**) Steady-state levels of COX11, COX19, and CcO subunit COX2 in the indicated cell lines, assessed by immunoblotting with respective antibodies. ACTIN served as the loading control. (**F**) CcO specific activity measured spectrophotometrically in frozen-thawed cells following the oxidation of exogenously reduced cytochrome *c*. The bars are the average ± S.D. of three independent replicates. Black dots represent individual data points. Two-sided unpaired *t*-test, ****p<0.0001. (**G**) Steady-state levels of complex IV subassemblies, holocomplex, and supercomplexes in the indicated cell lines, detected by BN-PAGE using an antibody against COX1. (**H**)

Immunoprecipitation of COX11-FLAG or COX11^{C219D}-FLAG in the indicated cellular backgrounds analyzed as described in panel B. Figures in panels A-E and G-H are representative of three independent repetitions with similar results. Source data for panels A-F, and H are provided as a Source Data file.

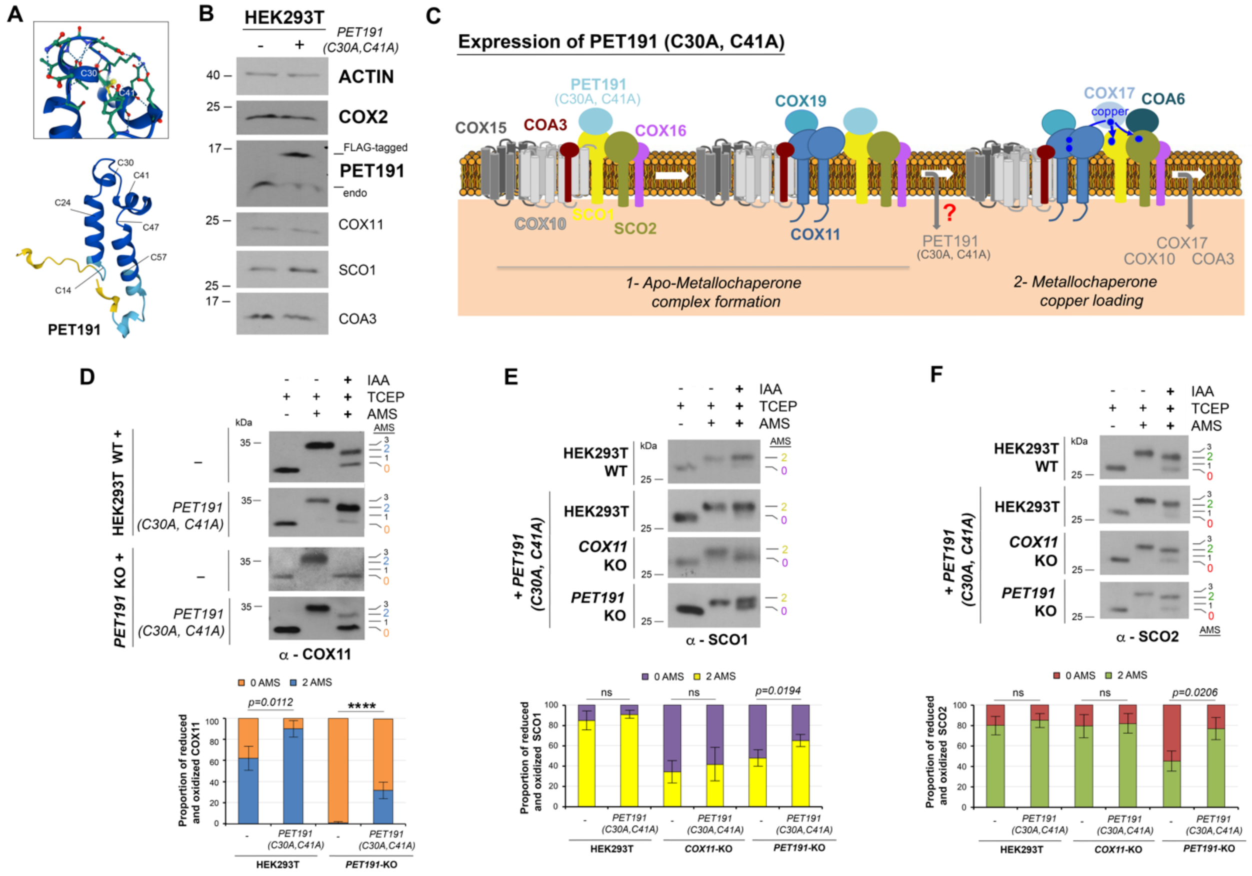

Supplementary Fig. 11: Effect of mutant *PET191* overexpression on the redox state of COX11, SCO1 and SCO2.

(**A**) Alpha-fold structure of human PET191 protein. Cysteine residues are indicated. (**B**) Steady-state levels of COX10, COX11, PET191, SCO1, COX16, and CcO subunit COX2 in the indicated cell lines upon overexpression of the PET191 (C30A, C41A) mutant variant, assessed by immunoblotting with the respective antibodies. ACTIN served as the loading control. (**C**) Cartoons depicting the consequences of overexpression of the PET191 (C30A, C41A) mutant variant in the stability of early apo-metallochaperone complexes. (**D-F**) Reverse COX11, SCO1 and SCO2 thiol trapping in the indicated cell lines performed as in **Fig. 2** and **Fig. 4** upon overexpression of the PET191 (C30A, C41A) mutant variant. The graphs show the quantification (mean ± SD) of three (panel D) or five (panels E and F) independent experiments. Two-sided unpaired *t*-test, ****p<0.0001. Figures in panel B are representative of three independent repetitions with similar results. Source data for panels B, D, E, and F are provided as a Source Data file.

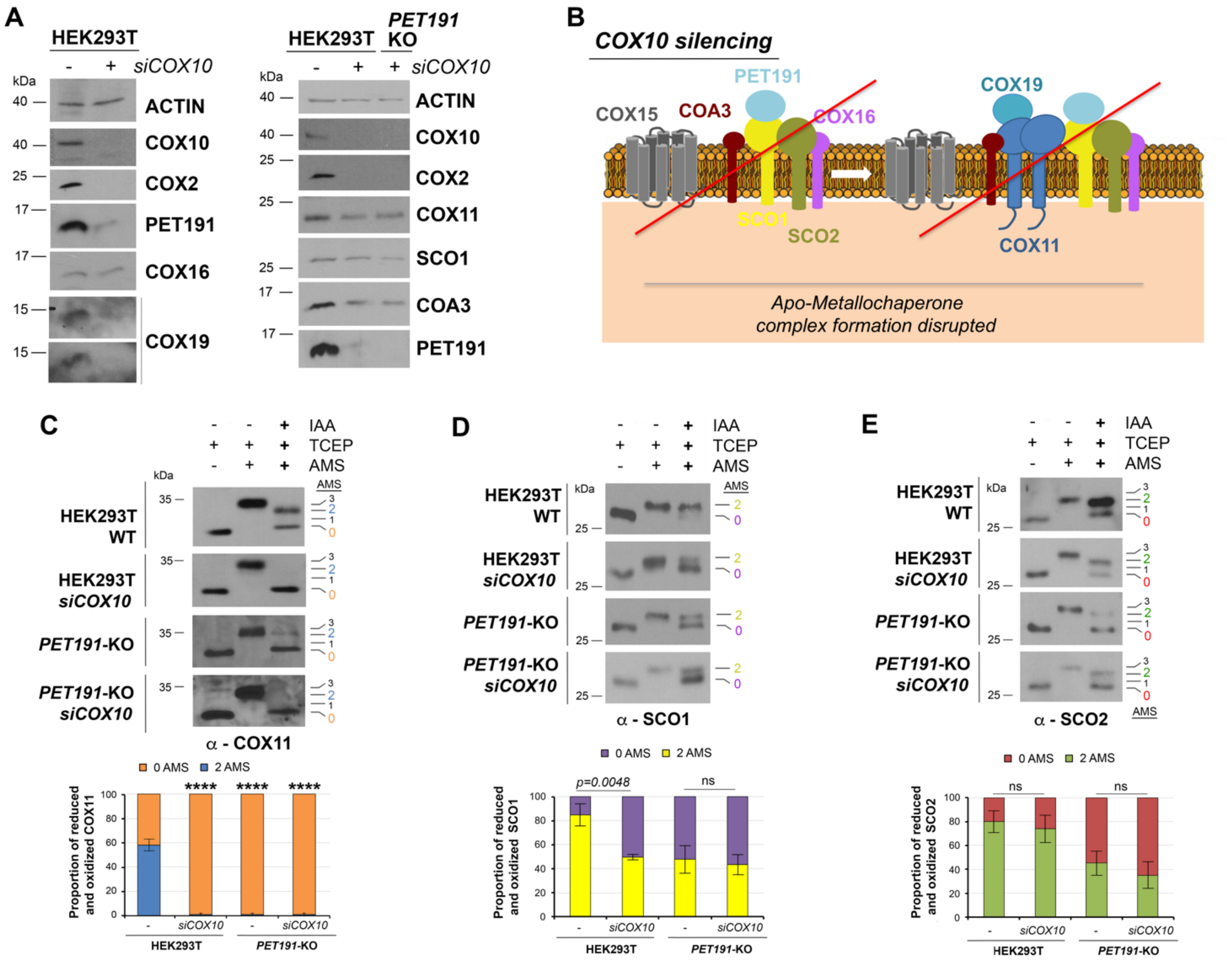

Supplementary Fig. 12: Effect of *COX10* silencing on the redox state of COX11, SCO1 and SCO2. (**A**) Steady-state levels of COX10, COX11, PET191, SCO1, COX16, COX19 (two independent blots are presented), and CcO subunit COX2 in the indicated cell lines upon *COX10* silencing, assessed by immunoblotting with respective antibodies. ACTIN served as the loading control. (**B**) Cartoons depicting the consequences of COX10 silencing in the stability of early apo-metallochaperone complexes. (**C-E**) Reverse COX11, SCO1 and SCO2 thiol trapping in the indicated cell lines performed as in **Fig. 2** and **Fig. 4** upon *COX10* silencing. The graphs show the quantification (mean ± SD) of three (panel C) or four (panels D and E) independent experiments. Two-sided unpaired *t*-test, ****p<0.0001. Figures in panel A are representative of three independent repetitions with similar results. Source data for panels A, C, B, D, and E are provided as a Source Data file.

Supplementary Tables

Supplementary Table 1: CcO assembly factors identified in humans

Protein	Function	Refs.
**Expression, and membrane insertion of core subunits**		
**TACO1**	Translational activator of *COX1* mRNA	3
**LRPPRC**	Stability of mitochondrial mRNAs, particularly *COX1/2/3*	4-6
**COA3**<br>MITRAC12<br>COX25	COX1 stabilization factor proposed to regulate COX1 synthesis	7, 8
**COX14**<br>C12orf62	COX1 stabilization factor proposed to regulate COX1 synthesis	9, 10
**OXA1L**	COX1/2/3 inner membrane insertase	11, 12
**COX18**	Translocates the COX2 C-terminal tail into the IMS	13
**COX20**	COX2 chaperone. Presents COX2 to SCO1/2 for copper insertion	14
**Heme A biosynthesis and assembly of heme *a* and hem a_3 sites**		
**COX10**	Farnesylation of heme B to generate heme O	15, 16
**COX15**	Hydroxylation of heme O to form heme A	17-19
**PET117**	Proposed to couple heme A synthesis and assembly	20, 21
**FDX2**	Cooperates with COX15 in heme O oxidation	22
**ADR**	Cooperates with COX15 in heme O oxidation	22
**SURF1**	Proposed to participate in heme a_3 center assembly or stabilization	23, 24
**Copper trafficking and assembly of Cu_A and Cu_B sites**		
**SLC25A3**	Carrier that imports copper and inorganic phosphate into mitochondria	25
**COX17**	Twin CX_9C protein that binds Cu^{1+} in the IMS for its delivery to SCO1/2 and COX11	26, 27
**SCO1**	Receives Cu^{1+} from COX17 and delivers it to COX2	28, 29
**SCO2**	Receives Cu^{1+} from COX17 and uses it to reduce the Cu_A coordinating cysteines in COX2	29, 30
**COA6**	Cooperates with SCO2 in copper delivery to COX2	31-33
**COA7**	Redox regulation of SCO1 and SCO2	34
**COX11**	Receives Cu^{1+} from COX17 and delivers it to COX1	35, 36<br>This study
**COX19**	Interact s with and protects COX11 from overoxidation	37<br>This study
**PET191**<br>COA5	Twin CX_9C protein that stabilizes complexes containing apo forms of COX1 and COX2 copper chaperones, acting as a place holder for COX17. Promotes Cu_B assembly in the absence of COX11	38, 39<br>This study
**CMC1**	Twin CX_9C protein that forms a complex with newly synthesized COX1, COX14 and COA3 prior to Cu_B assembly	1
**CMC2**	Twin CX_9C protein of unknown specific function	40
**COX23**	Twin CX_9C protein of unknown specific function	41
**Assembly chaperones**		
**COA1**<br>MITRAC15	Interacts with SURF1-containing CcO subassemblies	8, 42
**MITRAC7**	Stabilizes newly synthesized COX1 in the COX1-COX5A-COX4 -containing module	43
**MR-1S**	Interacts PET100 and PET117 chaperones	20
**TMEM177**	Associates with newly synthesized COX2, COX20, and SCO2.	44
**COX16**	Coordinates the merging of COX1 and COX2 modules	45, 46
**HIGD1A**	Complexes with the COX4-COX5A module to promote assembly with COX1	2, 20
**HIGD2A**	Promotes the assembly of the COX3 module	2, 47
**COA4**	Twin CX_9C protein of unknown specific function	48
**PET100**	Fuels the late stages of CcO assembly	20

Supplementary Table 2: List of oligonucleotides used in this study

Gene	Oligonucleotides
**COX11_F**	AAGCGATCGCATGGGAGGGCTCTGGCGT
**COX11_R**	AAACGCGTATTATATCCTGGAACTGGCA
**SCO2_F**	AAAGCGATCGCATGCTGCTGCTGACTCGGA
**SCO2_R**	AAAACGCGTAGACAGGACACTGCGGAAA
**SCO1_F**	AAGCGATCGCCATGGCGATGCTGGTCCTA
**SCO1_R**	AAACGCGT GCTCTTTTTTCTGTATGG
**talenPET191_F**	GTTGTTGCCGCTGGCTTCCGGT
**talenPET191_R**	CCTGGACCACACAGTCCGA
**CRISPR/CAS9Cox11_F**	TTCAGAGGGTTATGGGAGGGCT
**CRISPR/CAS9Cox11_R**	TGGCAATAGAGCCGATA
**CRISPR/CAS9Cox19_F**	GTGCTCGGCGCTCCCGGGGT
**CRISPR/CAS9Cox19_R**	TCACCTAAGTGATCCA
**PET191_mut_C30A_F**	GCAGTCGGACGCGGTGGTCCAGGAAGGAAAATCACCTC
**PET191_mut_C30A_R**	AGCAGACACGCGCCCAGG
**PET191_mut_C41A_F**	ACCTCGGCAGGCGTTGAAGGAAGGATAC
**PET191_mut_C41A_R**	GATTTTCCTTCCTGGACC
**COX11_mut_C217A_F**	TAAAATACAGGACTTCTGTTTTGAAGAAC
**COX11_mut_C217A_R**	TTGAAATACTGTCCAGCTTC
**COX11_mut_C219A_F**	ACAGTGCTTCGACTTTGAAGAACAAAGG
**COX11_mut_C219A_R**	ATTTTATTGAAATACTGTCCAG
**ScCOX11_BamH1_F**	CCGGGGATCCTGTTTGCTGAC
**ScCOX11_Hind3_R**	CCGGAAGCTTCAATTAATTTGAGTTGTCTTTCCTTG
**ScCOX11_Sgf1_F**	AAGCGATCGCATGATAAGAATATGTCCCAT
**ScCOX11_Mlu1_R**	AAACGCGTATTGAGTTGTCTTTCCTT
**HsCOX11_Kpn1_F**	AAGGTACCATGGAGGGCTCTGGCGTCCT
**HsCOX11_Sal1_R**	AAGTCGACTCATTATATCCTGGAACT

Supplementary Table 3. Yeast strains used in this study

Strain	Genotype	Reference/Source
W303-1B	*MATα ade2-1 can1-100 his3-11,15 leu2-3 trp1-1 ura3-1*	ATCC
cox11Δ	*MATa ade2-1 his3-11 leu2-3,112 ura3-1 can^r gal^r cox11::HIS3*	49
pet191Δ	*MATα ade2-1 can1-100 his3-11,15 leu2-3 trp1-1 ura3-1 pet191::URA3MX*	This study
pet117Δ	*MATα ade2-1 can1-100 his3-11,15 leu2-3 trp1-1 ura3-1 pet117::URA3MX*	21
coa3Δ	*MATa ade2-1 his3-11,15 leu2-3,112 trp1-1 ura3-1 coa3::KanMX*	50
cox11Δ coa3Δ	*MATa ade2-1 his3-11,15 leu2-3,112 trp1-1 ura3-1 coa3::KanMX cox11::HIS3*	50
cox11Δ pet191Δ	*MATa ade2-1 his3-11 leu2-3,112 ura3-1 can^r gal^r cox11::HIS3 pet191::URA3MX*	This study
cox11Δ pet117Δ	*MATa ade2-1 his3-11 leu2-3,112 ura3-1 can^r gal^r cox11::HIS3 pet117::KanMX4*	21

Supplementary References

1. Bourens, M. & Barrientos, A. A CMC1-Knockout reveals translation-independent control of human mitochondrial Complex IV biogenesis. *EMBO Rep.* **8**, 477-494 (2017).
2. Timón-Gómez, A., Garlich, J., Stuart, R.A., Ugalde, C. & Barrientos, A. Distinct roles of mitochondrial HIGD1A and HIGD2A in respiratory complex and supercomplex biogenesis. *Cell Rep.* **31**, 107607 (2020).
3. Weraarpachai, W. *et al.* Mutation in TACO1, encoding a translational activator of COX I, results in cytochrome *c* oxidase deficiency and late-onset Leigh syndrome. *Nat. Genet.* **41**, 833-837. (2009).
4. Mootha, V.K. *et al.* Identification of a gene causing human cytochrome *c* oxidase deficiency by integrative genomics. *Proc. Natl. Acad. Sci. U. S. A.* **100**, 605-610. (2003).
5. Ruzzenente, B. *et al.* LRPPRC is necessary for polyadenylation and coordination of translation of mitochondrial mRNAs. *EMBO J.* **31**, 443-456 (2012).
6. Sasarman, F., Brunel-Guitton, C., Antonicka, H., Wai, T. & Shoubridge, E.A. LRPPRC and SLIRP interact in a ribonucleoprotein complex that regulates posttranscriptional gene expression in mitochondria. *Mol. Biol. Cell* **21**, 1315-1323. (2010).
7. Clemente, P. *et al.* hCOA3 stabilizes cytochrome *c* oxidase 1 (COX1) and promotes cytochrome *c* oxidase assembly in human mitochondria. *J. Biol .Chem.* **288**, 8321-8331 (2013).
8. Mick, D.U. *et al.* MITRAC links mitochondrial protein translocation to respiratory-chain assembly and translational regulation. *Cell* **151**, 1528-1541 (2012).
9. Weraarpachai, W. *et al.* Mutations in C12orf62, a factor that couples COX I synthesis with cytochrome *c* oxidase assembly, cause fatal neonatal lactic acidosis. *Am. J. Hum. Genet.* **90**, 142-151 (2012).
10. Richter-Dennerlein, R. *et al.* Mitochondrial protein synthesis adapts to influx of nuclear-encoded protein. *Cell* **167**, 471-483 (2016).
11. Thompson, K. *et al.* OXA1L mutations cause mitochondrial encephalopathy and a combined oxidative phosphorylation defect. *EMBO Mol. Med.* **10** (2018).
12. Itoh, Y. *et al.* Mechanism of membrane-tethered mitochondrial protein synthesis. *Science* **371**, 846-849 (2021).
13. Bourens, M. & Barrientos, A. Human mitochondrial cytochrome c oxidase assembly factor COX18 acts transiently as a membrane insertase within the subunit 2 maturation module. *J. Biol. Chem.* **292**, 7774-7783 (2017).
14. Bourens, M., Boulet, A., Leary, S.C. & Barrientos, A. Human COX20 cooperates with SCO1 and SCO2 to mature COX2 and promote the assembly of cytochrome *c* oxidase. *Hum. Mol. Genet.* **23**, 2901-2913 (2014).

15. Valnot, I. *et al.* A mutation in the human heme A:farnesyltransferase gene (*COX10*) causes cytochrome *c* oxidase deficiency. *Hum. Mol. Genet.* **9**, 1245-1249. (2000).
16. Khalimonchuk, O., Kim, H., Watts, T., Perez-Martinez, X. & Winge, D.R. Oligomerization of heme o synthase in cytochrome oxidase biogenesis is mediated by cytochrome oxidase assembly factor Coa2. *J. Biol. Chem.* **287**, 26715-26726 (2012).
17. Antonicka, H. *et al.* Mutations in *COX15* produce a defect in the mitochondrial heme biosynthetic pathway, causing early-onset fatal hypertrophic cardiomyopathy. *Am. J. Hum. Genet.* **72**, 101-114. (2003).
18. Bareth, B. *et al.* The heme a synthase Cox15 associates with cytochrome c oxidase assembly intermediates during Cox1 maturation. *Mol. Cell. Biol.*, Aug 26 [Epub ahead of print] (2013).
19. Barros, M.H., Carlson, C.G., Glerum, D.M. & Tzagoloff, A. Involvement of mitochondrial ferredoxin and Cox15p in hydroxylation of heme O. *FEBS Lett.* **492**, 133-138 (2001).
20. Vidoni, S. *et al.* MR-1S interacts with PET100 and PET117 in module-based assembly of human cytochrome *c* oxidase. *Cell Rep.* **18**, 1727-1738 (2017).
21. Taylor, N.G. *et al.* The assembly factor Pet117 couples heme A synthase activity to cytochrome oxidase assembly. *J. Biol. Chem.* **292**, 1815-1825 (2017).
22. Barros, M.H., Nobrega, F.G. & Tzagoloff, A. Mitochondrial ferredoxin is required for heme A synthesis in *Saccharomyces cerevisiae*. *J. Biol. Chem.* **277**, 9997-10002 (2002).
23. Zhu, Z. *et al. SURF1*, encoding a factor involved in the biogenesis of cytochrome *c* oxidase, is mutated in Leigh syndrome. *Nat. Genet.* **20**, 337-343. (1998).
24. Tiranti, V. *et al.* Mutations of *SURF-1* in Leigh disease associated with cytochrome *c* oxidase deficiency. *Am. J. Hum. Genet.* **63**, 1609-1621. (1998).
25. Boulet, A. *et al.* The mammalian phosphate carrier SLC25A3 is a mitochondrial copper transporter required for cytochrome *c* oxidase biogenesis. *J. Biol. Chem.* **293**, 1887-1896 (2018).
26. Glerum, D.M., Shtanko, A. & Tzagoloff, A. Characterization of *COX17*, a yeast gene involved in copper metabolism and assembly of cytochrome oxidase. *J. Biol. Chem.* **271**, 14504-14509 (1996).
27. Banci, L. *et al.* Mitochondrial copper(I) transfer from Cox17 to Sco1 is coupled to electron transfer. *Proc. Natl. Acad. Sci. U. S. A.* **105**, 6803-6808 (2008).
28. Glerum, D.M., Shtanko, A. & Tzagoloff, A. *SCO1* and *SCO2* act as high copy suppressors of a mitochondrial copper recruitment defect in *Saccharomyces cerevisiae*. *J. Biol. Chem.* **271**, 20531-20535 (1996).
29. Leary, S.C. *et al.* Human SCO1 and SCO2 have independent, cooperative functions in copper delivery to cytochrome *c* oxidase. *Hum. Mol. Genet.* **13**, 1839-1848 (2004).
30. Banci, L. *et al.* A structural characterization of human *SCO2*. *Structure* **15**, 1132-1140 (2007).
31. Soma, S. *et al.* COA6 is structurally tuned to function as a thiol-disulfide oxidoreductase in copper delivery to mitochondrial cytochrome c oxidase. *Cell Rep.* **29**, 4114-4126.e4115 (2019).
32. Stroud, D.A. *et al.* COA6 is a mitochondrial complex IV assembly factor critical for biogenesis of mtDNA-encoded COX2. *Hum. Mol. Genet.* **24**, 5404-5415 (2015).
33. Pacheu-Grau, D. *et al.* COA6 facilitates cytochrome c oxidase biogenesis as thiol-reductase for copper metallochaperones in mitochondria. *J. Mol. Biol.* **432**, 2067-2079 (2020).
34. Formosa, L.E. *et al.* Mitochondrial COA7 is a heme-binding protein with disulfide reductase activity, which acts in the early stages of complex IV assembly. *Proc. Natl. Acad. Sci. U. S. A.* **119** (2022).
35. Hiser, L., Di Valentin, M., Hamer, A.G. & Hosler, J.P. Cox11p is required for stable formation of the Cu(B) and magnesium centers of cytochrome c oxidase. *J. Biol. Chem.* **275**, 619-623 (2000).
36. Horng, Y.C., Cobine, P.A., Maxfield, A.B., Carr, H.S. & Winge, D.R. Specific copper transfer from the Cox17 metallochaperone to both Sco1 and Cox11 in the assembly of yeast cytochrome *c* oxidase. *J. Biol. Chem.* **279**, 35334-35340 (2004).
37. Bode, M. *et al.* Redox-regulated dynamic interplay between Cox19 and the copper-binding protein Cox11 in the intermembrane space of mitochondria facilitates biogenesis of cytochrome c oxidase. *Mol. Biol. Cell.* **26**, 2385-2401 (2015).
38. Huigsloot, M. *et al.* A mutation in C2orf64 causes impaired cytochrome *c* oxidase assembly and mitochondrial cardiomyopathy. *Am. J. Hum. Genet.* **88**, 488-493. (2011).
39. Khalimonchuk, O. *et al.* Pet191 is a cytochrome *c* oxidase assembly factor in *Saccharomyces cerevisiae*. *Eukaryotic Cell* **7**, 1427-1431 (2008).
40. Horn, D. *et al.* The conserved mitochondrial twin CX_9C protein Cmc2 is a Cmc1 homologue essential for cytochrome *c* oxidase biogenesis. *J. Biol. Chem.* **285**, 15088-15099 (2010).
41. Barros, M.H., Johnson, A. & Tzagoloff, A. *COX23*, a homologue of *COX17*, is required for cytochrome oxidase assembly. *J. Biol. Chem.* **279**, 31943-31947 (2004).

42. Pierrel, F. *et al.* Coa1 links the Mss51 post-translational function to Cox1 cofactor insertion in cytochrome *c* oxidase assembly. *EMBO J.* **26**, 4335-4346 (2007).
43. Dennerlein, S. *et al.* MITRAC7 acts as a COX1-specific chaperone and reveals a checkpoint during cytochrome c oxidase assembly. *Cell Rep.* **12**, 1644-1655 (2015).
44. Lorenzi, I. *et al.* The mitochondrial TMEM177 associates with COX20 during COX2 biogenesis. *Biochim. Biophys. Acta Mol. Cell Res.* **1865**, 323-333 (2018).
45. Aich, A. *et al.* COX16 promotes COX2 metallation and assembly during respiratory complex IV biogenesis. *Elife* **7** (2018).
46. Cerqua, C. *et al.* COX16 is required for assembly of cytochrome c oxidase in human cells and is involved in copper delivery to COX2. *Biochim. Biophys. Acta Bioenerg.* **1859**, 244-252 (2018).
47. Hock, D.H. *et al.* HIGD2A is required for assembly of the COX3 module of human mitochondrial complex IV. *Mol. Cell Proteomics* **19**, 1145-1160 (2020).
48. Bestwick, M., Jeong, M.Y., Khalimonchuk, O., Kim, H. & Winge, D.R. Analysis of Leigh syndrome mutations in the yeast SURF1 homolog reveals a new member of the cytochrome oxidase assembly factor family. *Mol. Cell Biol.* **30**, 4480-4491 (2010).
49. Carr, H.S., George, G.N. & Winge, D.R. Yeast Cox11, a protein essential for cytochrome *c* oxidase assembly, is a Cu(I)-binding protein. *J. Biol. Chem.* **277**, 31237-31242 (2002).
50. Fontanesi, F., Clemente, P. & Barrientos, A. Cox25 teams up with Mss51, Ssc1, and Cox14 to regulate mitochondrial cytochrome *c* oxidase subunit 1 expression and assembly in *Saccharomyces cerevisiae*. *J. Biol. Chem.* **286**, 555-566 (2011).

Supplementary Source Data for

Coordination of metal center biogenesis in human cytochrome *c* oxidase

Eva Nývltová, Jonathan V. Dietz, Javier Seravalli, Oleh Khalimonchuk, and Antoni Barrientos

Correspondence to: abarrientos@med.miami.edu

This PDF file includes:

(1) Representative source data for mitochondrial cytochrome spectra analyses. Related to Fig. 1. Mitochondria were extracted at a protein concentration of 5 mg/ml with potassium deoxycholate under conditions that quantitatively solubilize all the cytochromes (see Methods). Difference spectra of the reduced (with sodium dithionite) versus oxidized (with potassium ferricyanide) extracts were recorded at room temperature. The α absorption bands corresponding to cytochrome *b* (*b*) and for cytochrome *c* and c_1 (*c*) are 560 and 550 nm, respectively. The maxima for cytochromes *a* and a_3 extracted from the wild-type HEK293T cell line is at 603 nm (*a*). Deviations in the maxima for cytochromes *a* and a_3 in the mutant cell lines are indicated.

(2) Representative source data panels of the stable and transient protein interactomes analyses of CcO assembly chaperones: (2.1) COX11, (2.2) COX19, (2.3) PET191, (2.4) COX17, (2.5) COX10, (2.6) COA6, (2.7) SCO1, (2.8) SCO2, (2.9) COX16. The images correspond to the immunoprecipitation analyses of the indicated C-terminus FLAGged proteins in several cellular backgrounds. Mitochondria were purified from each strain, treated with DSP or the vehicle DMSO, extracted with 0.4% n-dodecyl β-D-maltoside (DDM), incubated with anti-FLAG-conjugated protein A beads, and processed for immunoblot analyses to detect the indicated proteins with respective antibodies. HEK293T cells without FLAG (WT) were used as negative control. Ex, extract; Un, unbound; IP, immunoprecipitate. The experiments were performed at least in biological triplicates, the images were digitalized, used for densitometry analyses, and the quantification of the data condensed in the heat maps presented in **Figure 3C-D**.

(3) Uncropped and unprocessed images of all immunoblots in the supplementary figures.

(1) Representative raw data for mitochondrial cytochrome spectra.

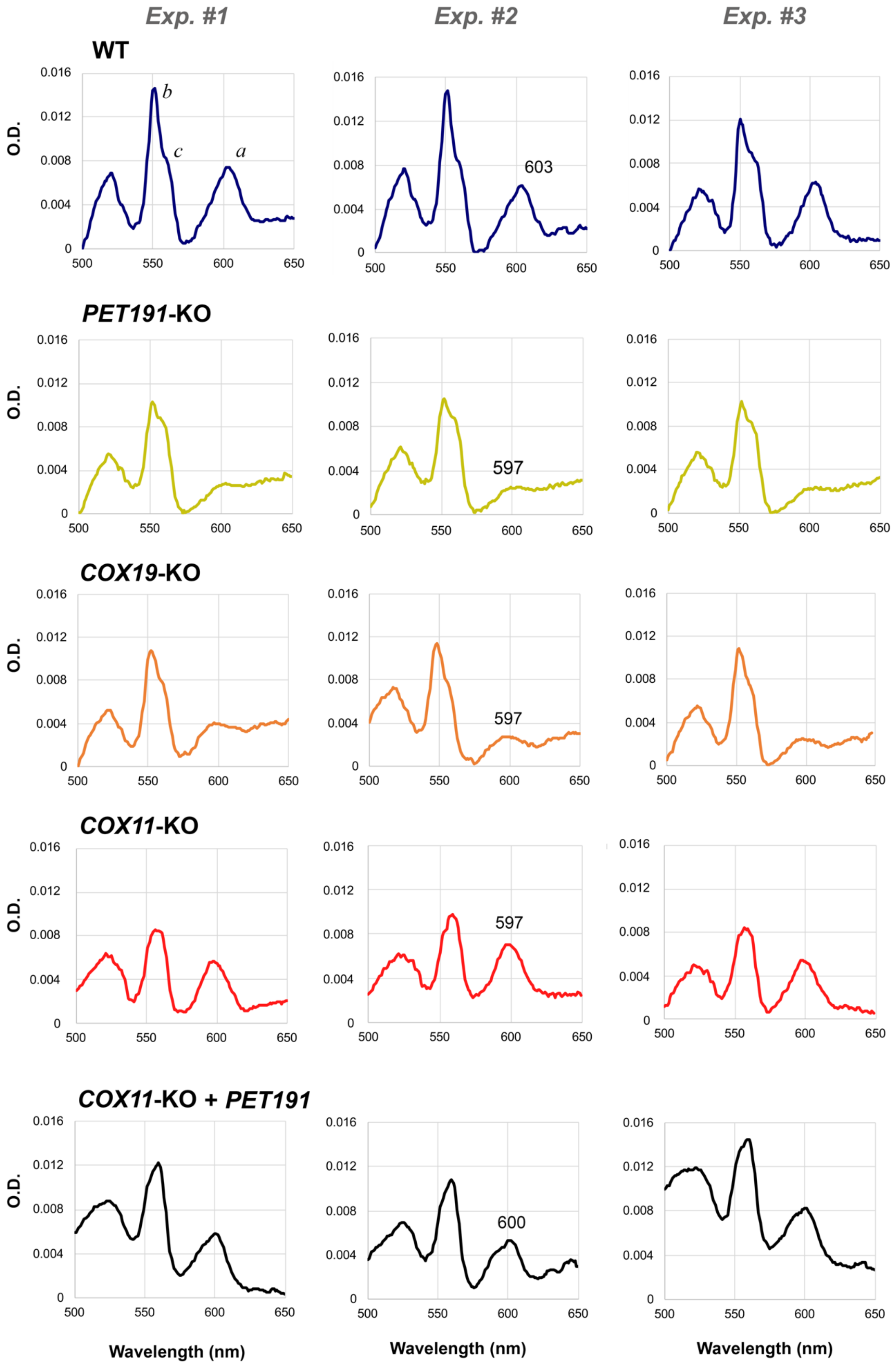

(2.1) The stable and transient protein interactomes of COX11

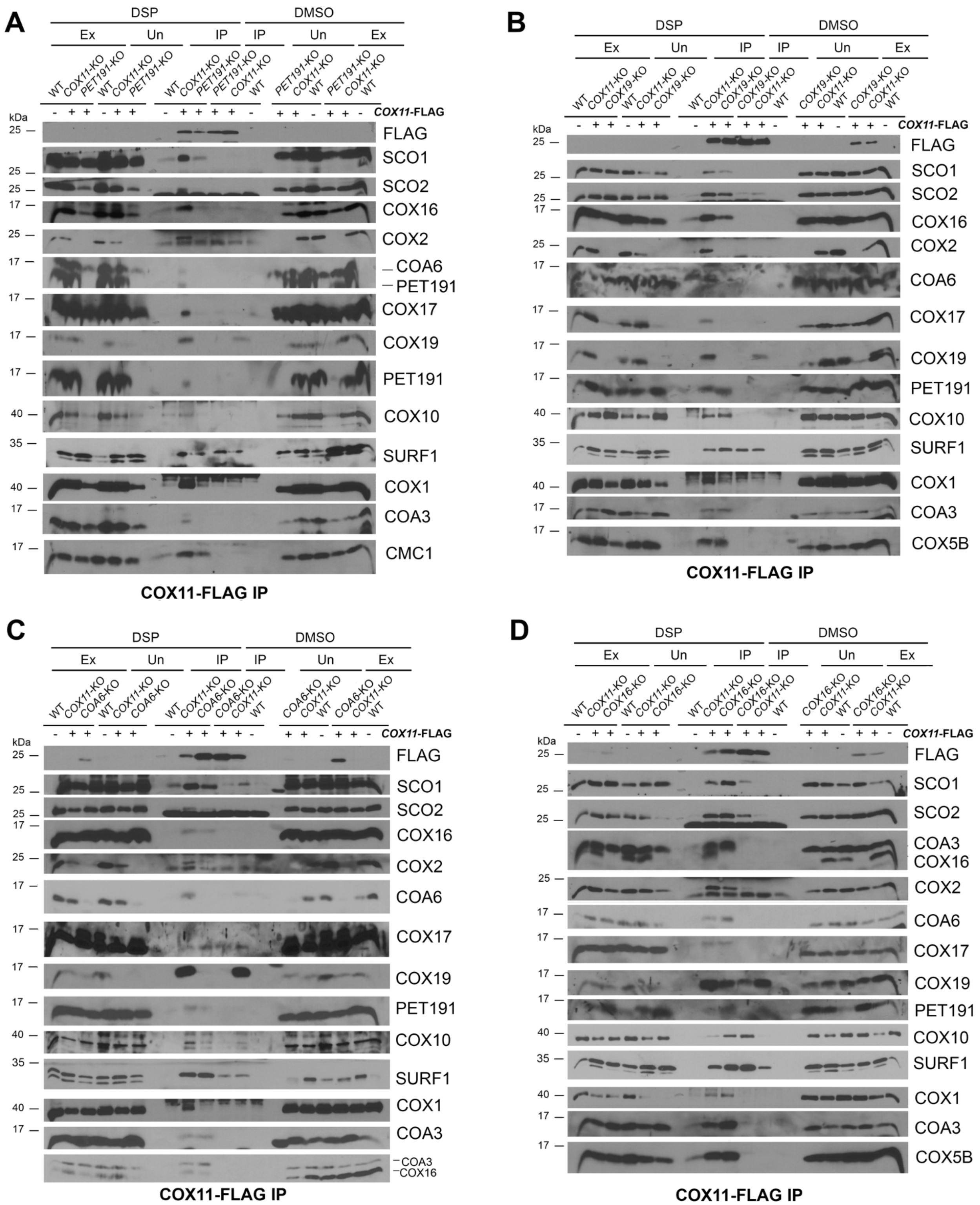

(2.2) The stable and transient protein interactomes of COX19

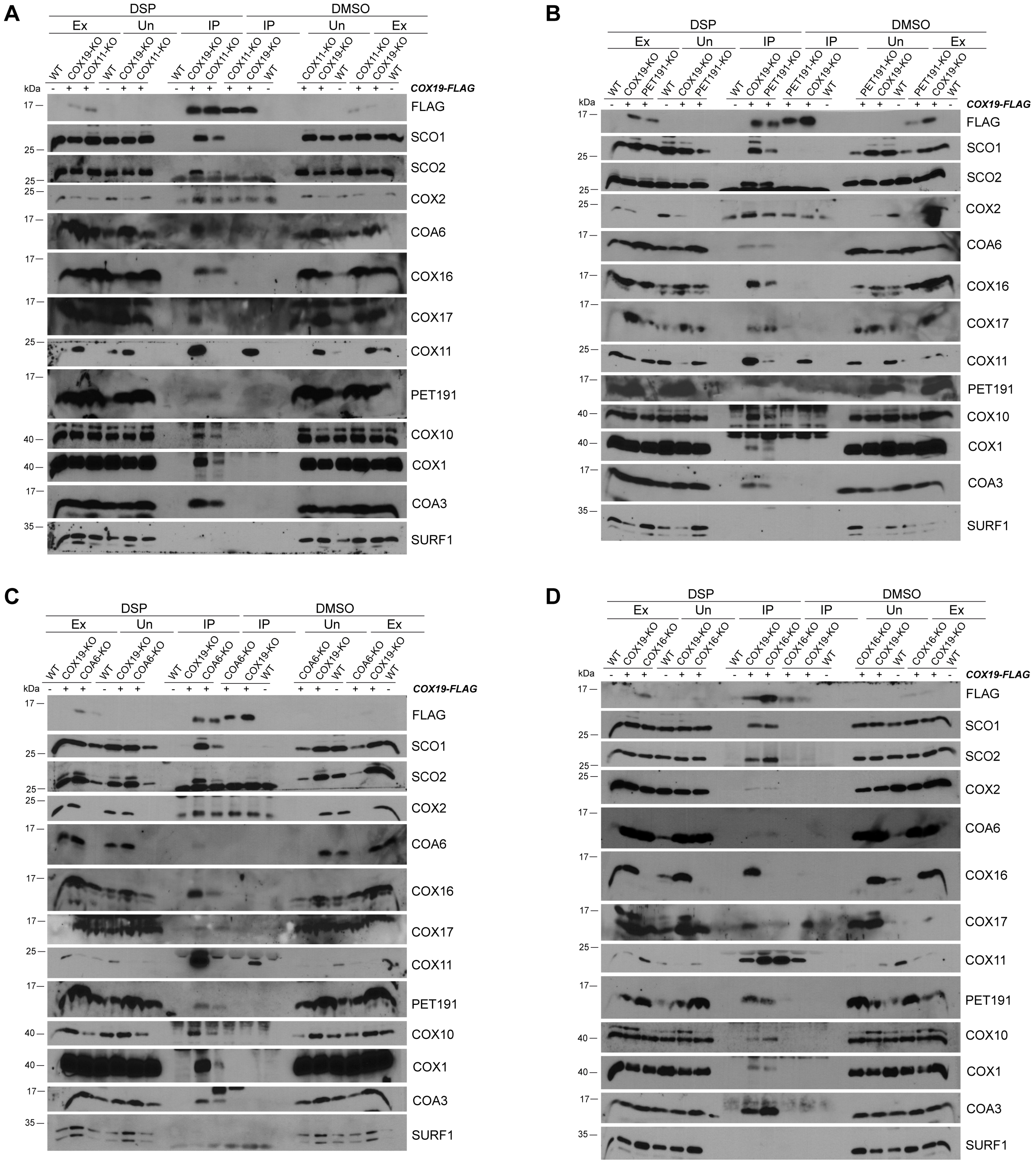

(2.3) The stable and transient protein interactomes of PET191

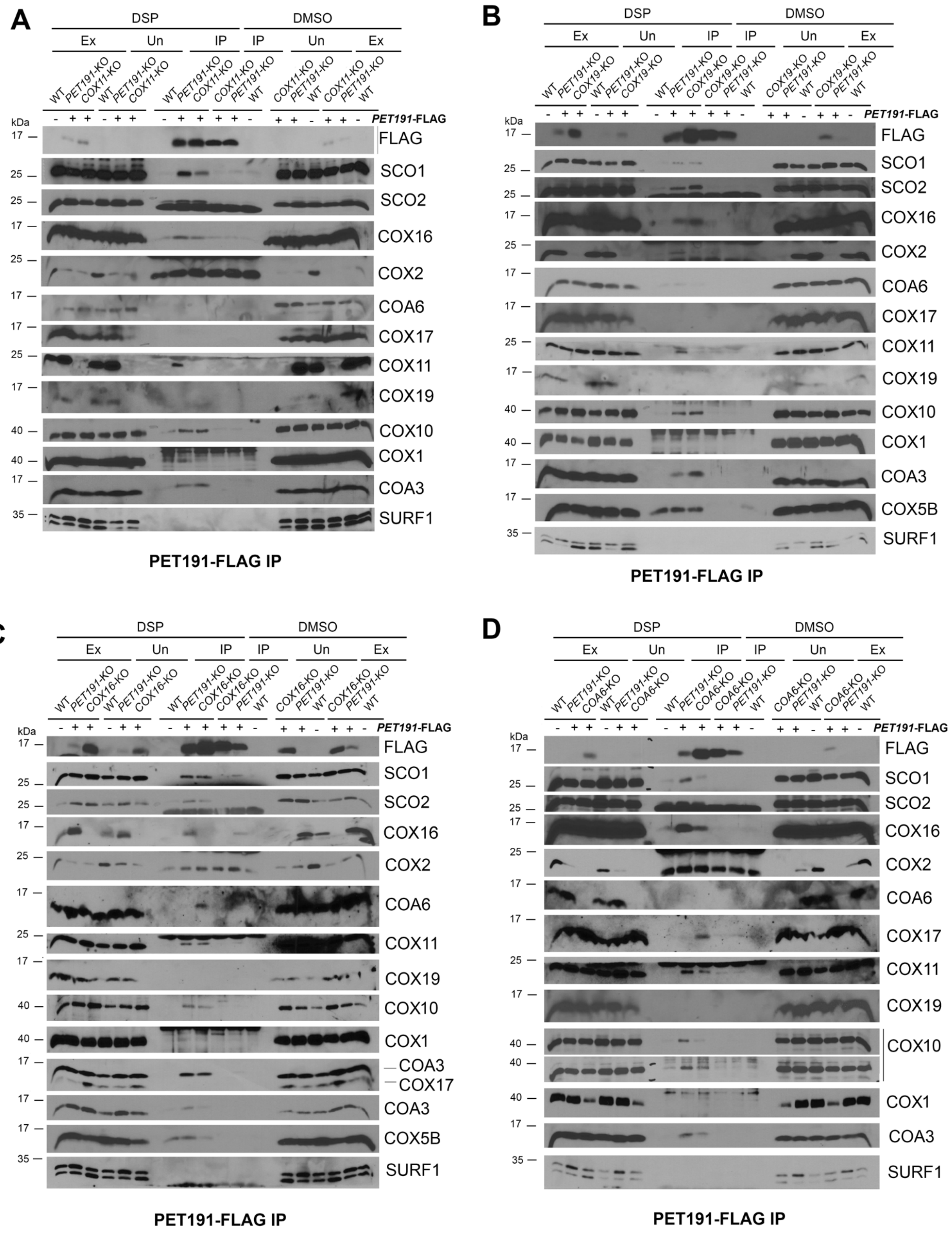

(2.4) The stable and transient protein interactomes of COX17

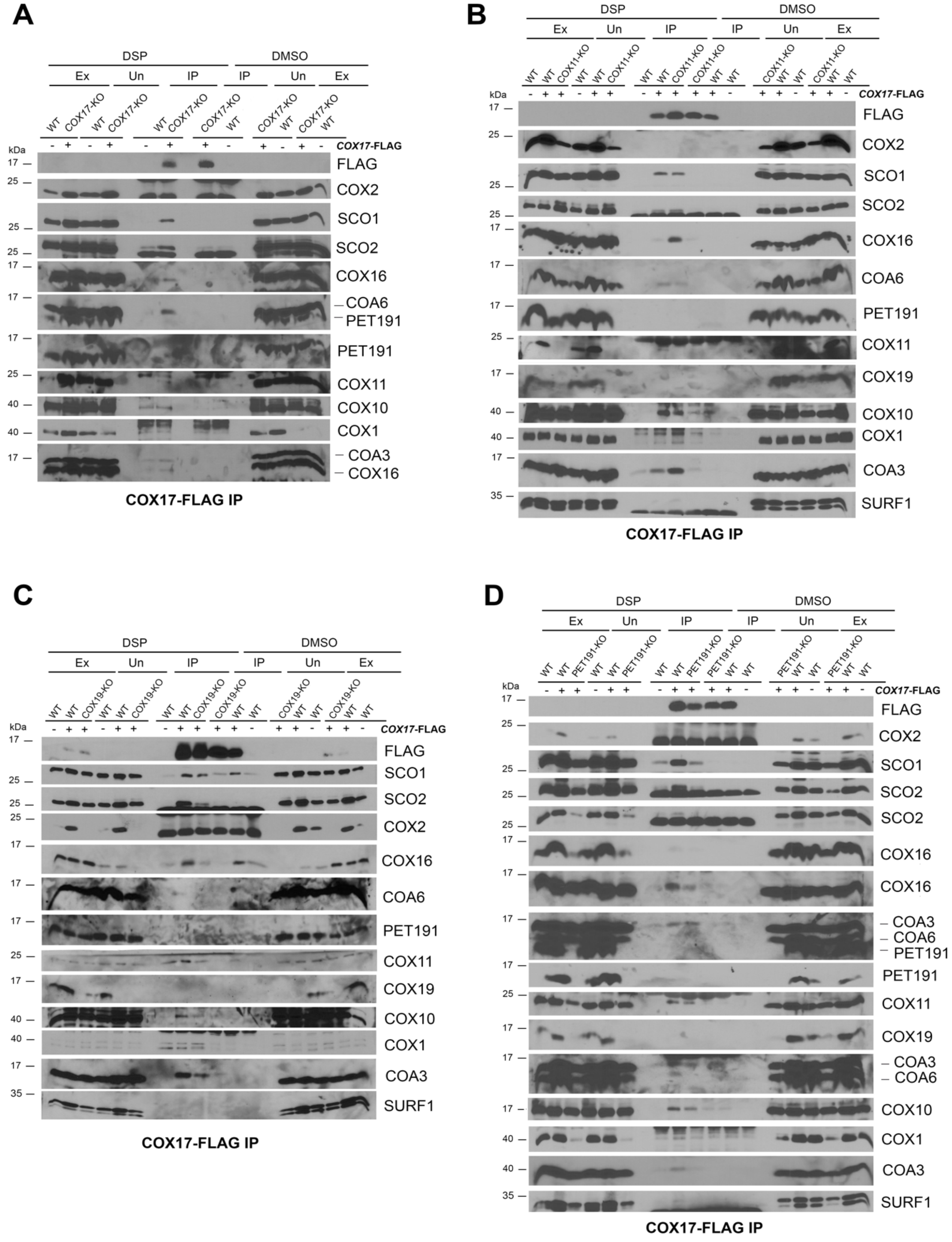

(2.5) The stable and transient protein interactomes of COX10

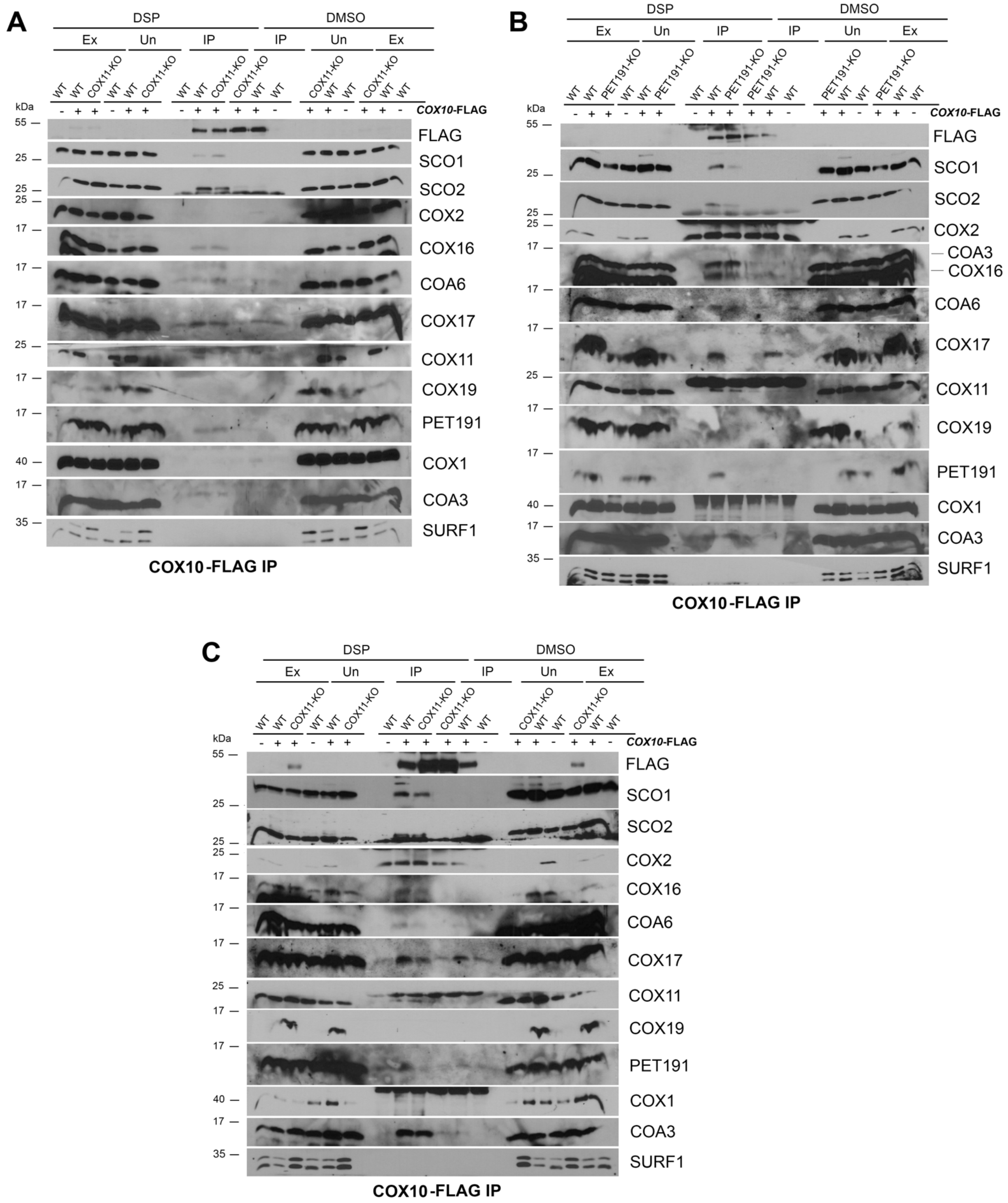

(2.6) The stable and transient protein interactomes of COA6

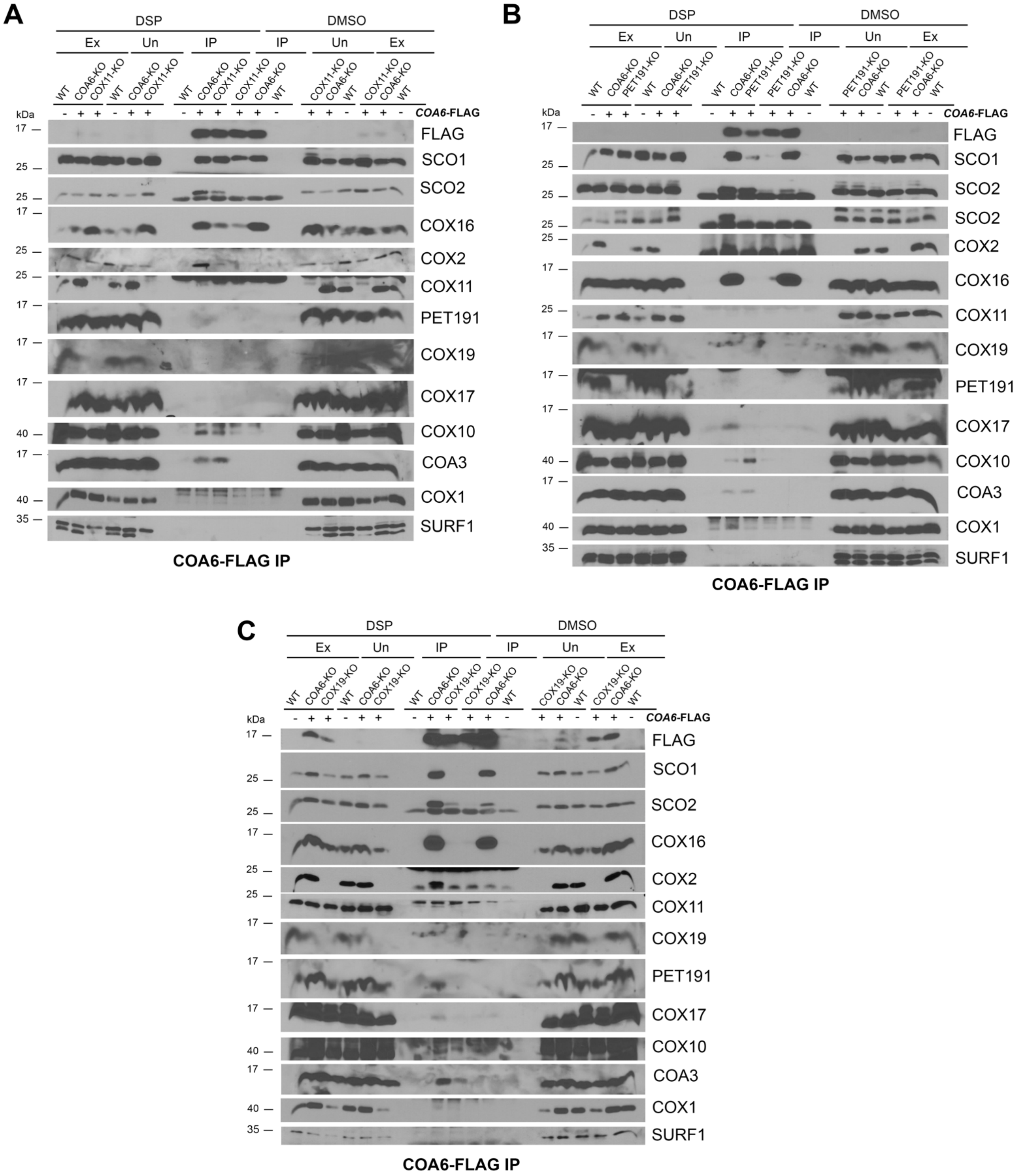

(2.7) The stable and transient protein interactomes of SCO1

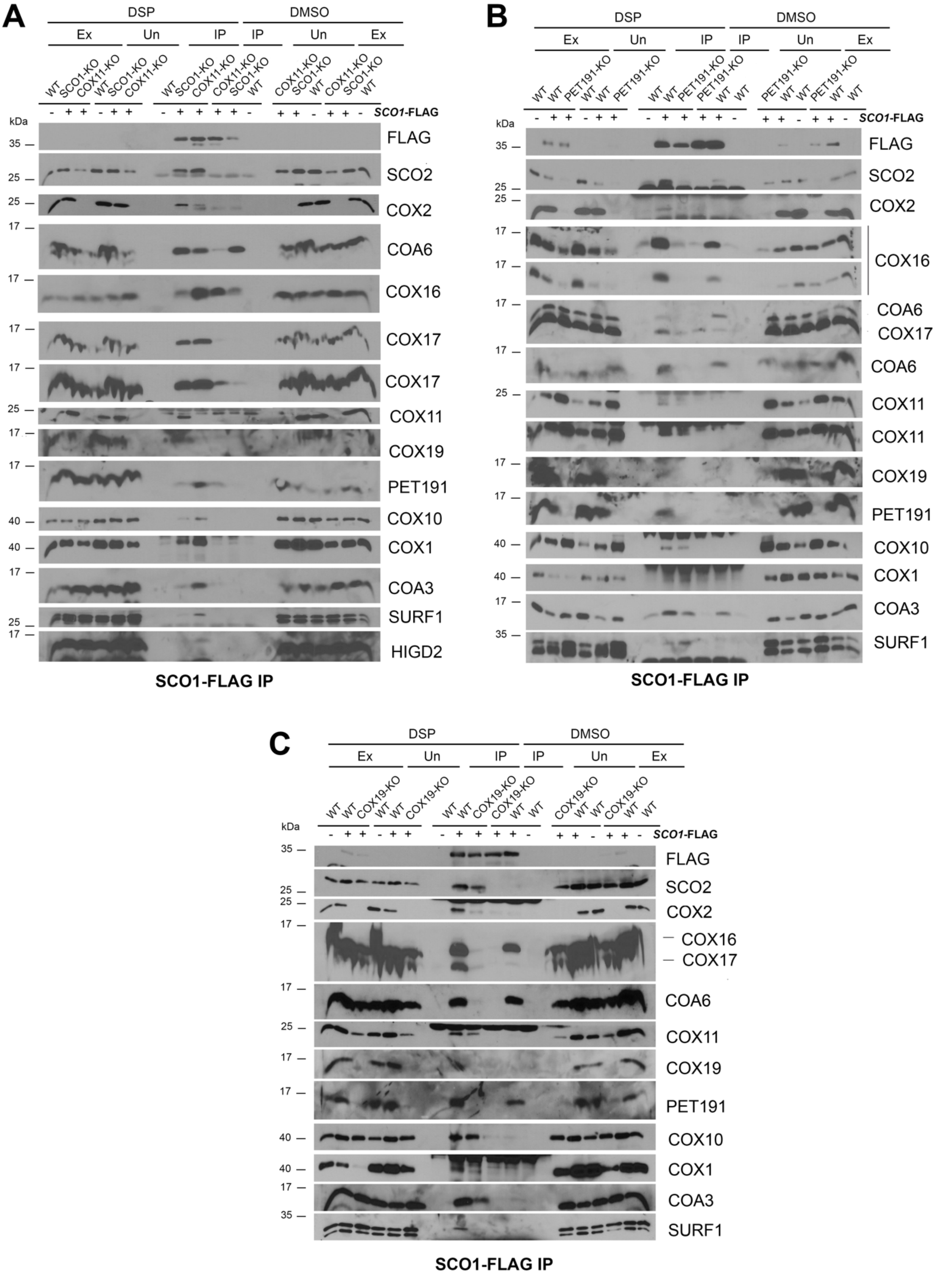

(2.8) The stable and transient protein interactomes of SCO2

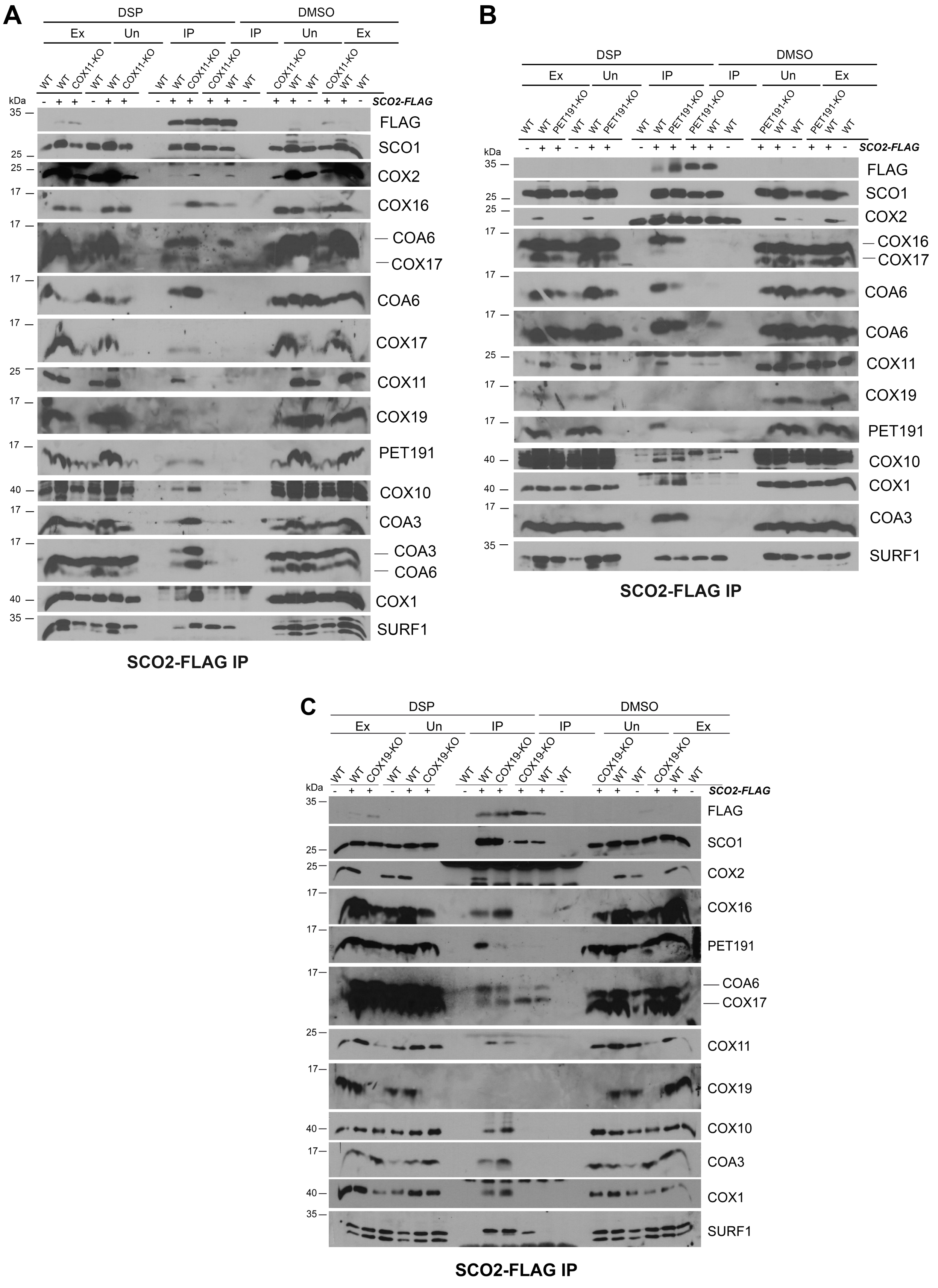

(2.9) The stable and transient COX16 protein interactomes of COX16.

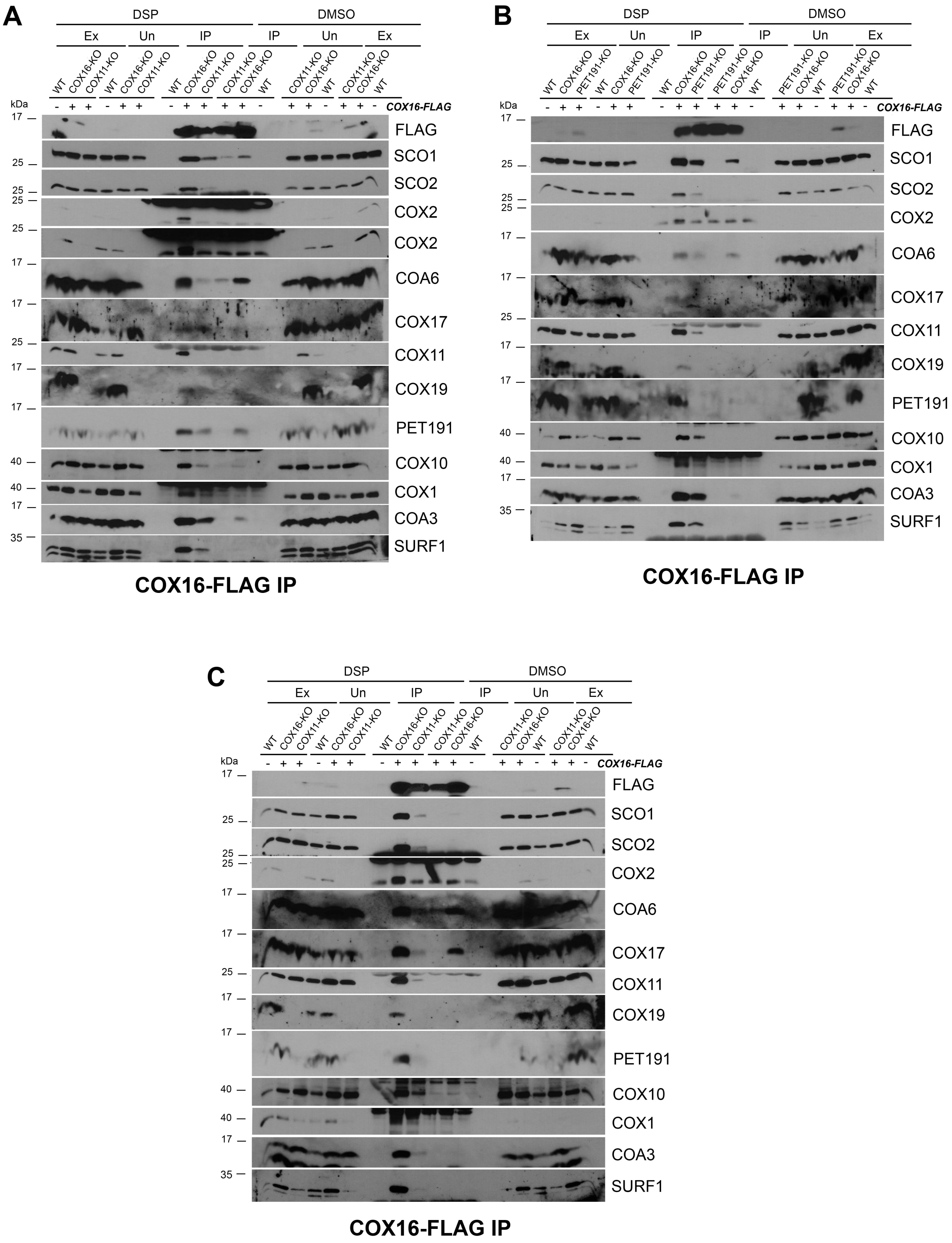

3. Uncropped and unprocessed images of all immunoblots in Supplementary figures

Supplementary Fig. 1B

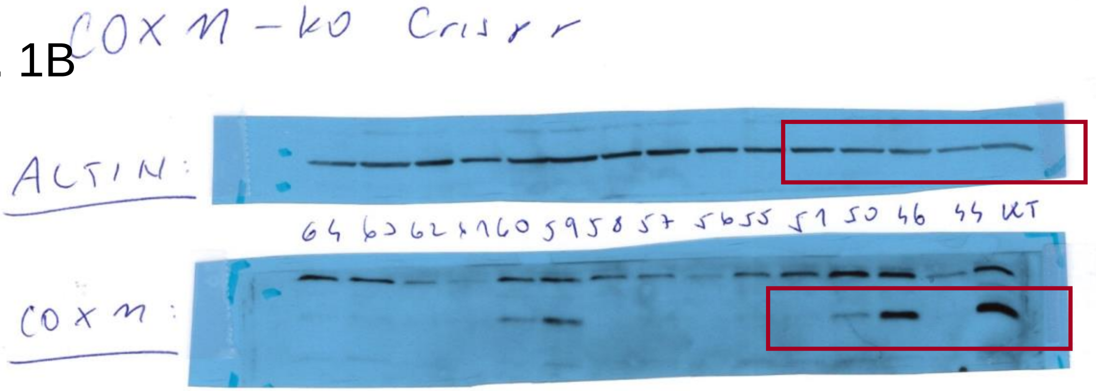

ACTIN

COX11

Supplementary Fig. 1F

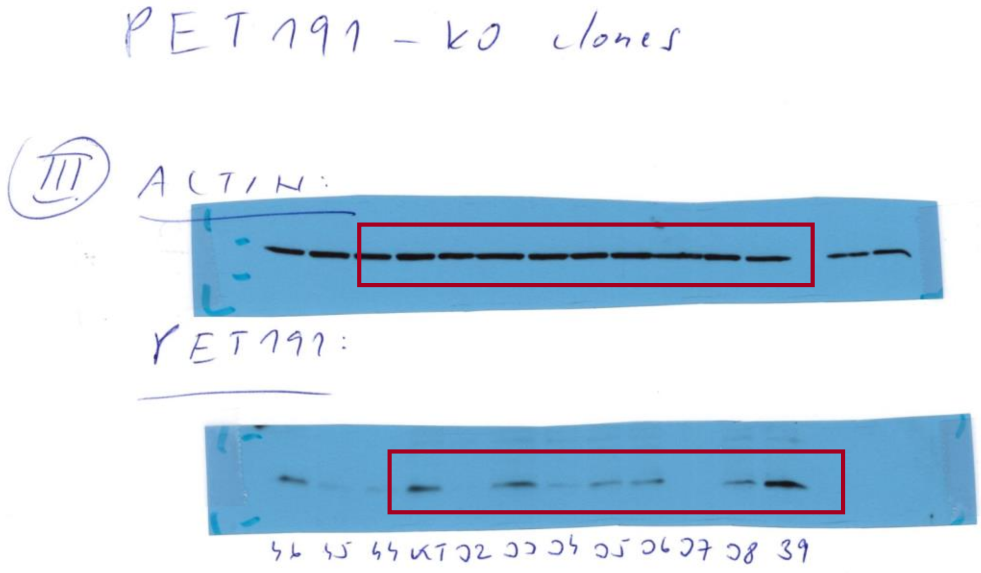

ACTIN

PET191

Supplementary Fig. 1E

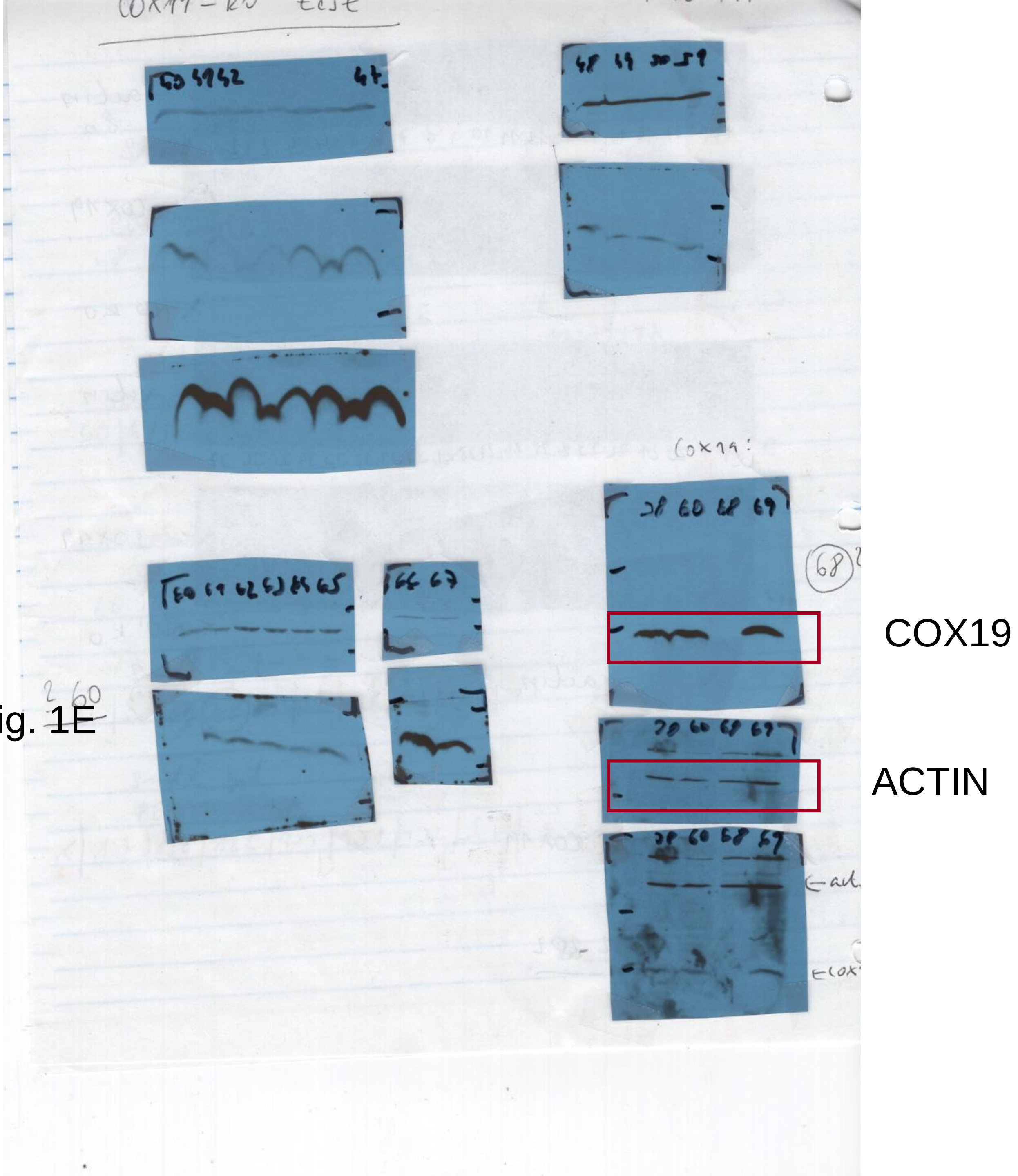

Supplementary Fig. 2A

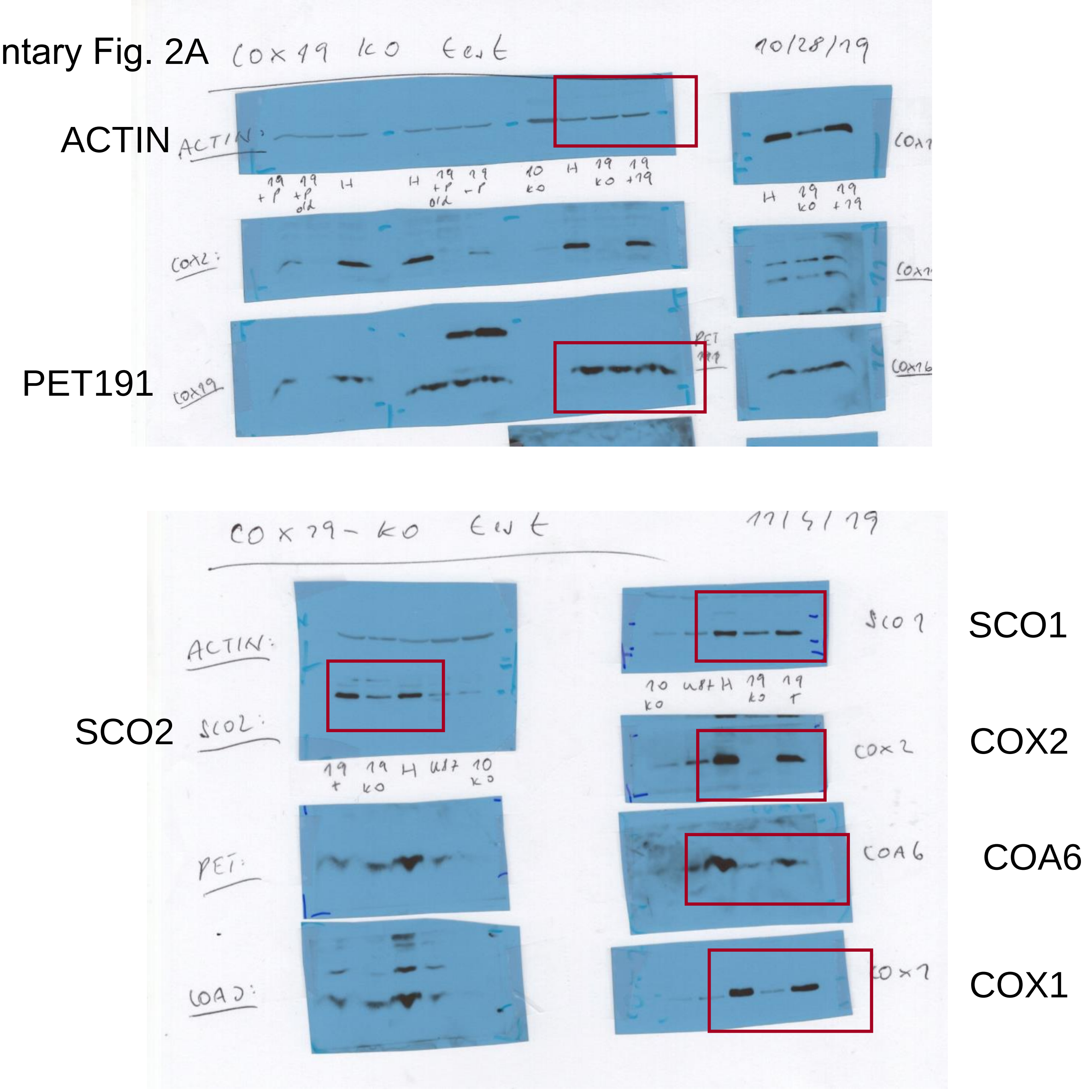

Supplementary Fig. 2A

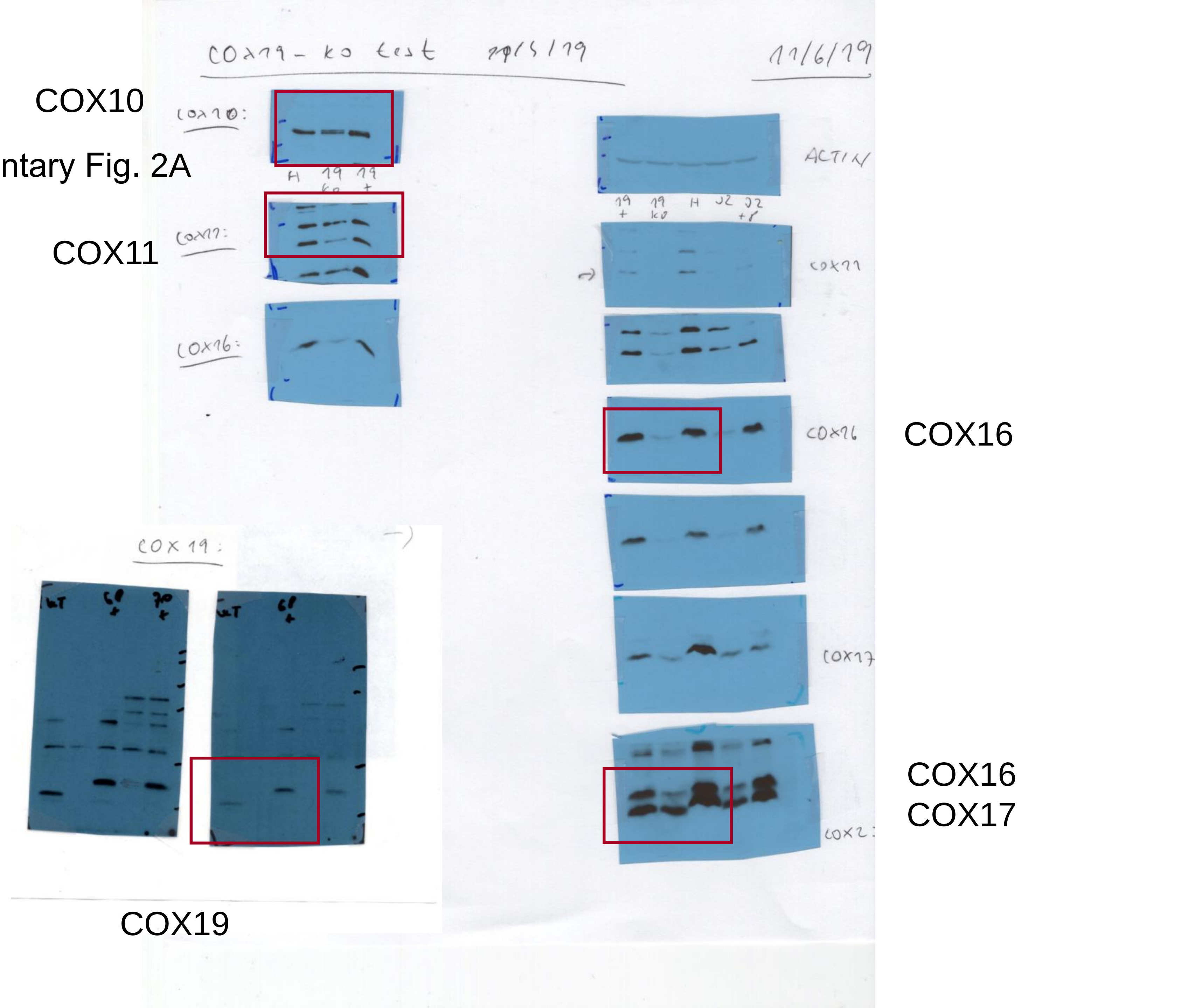

Supplementary Fig. 2A

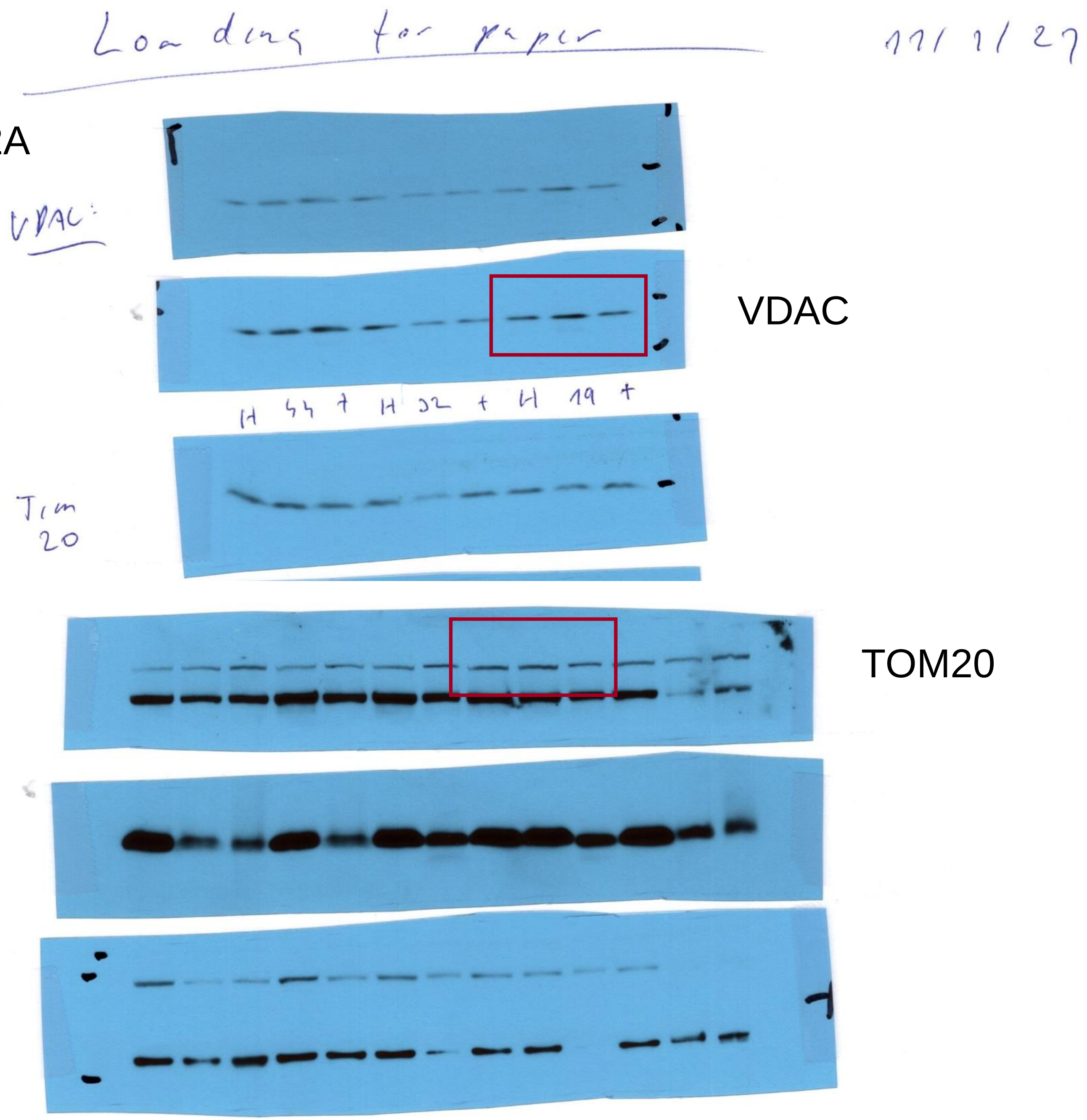

Supplementary Fig. 2B

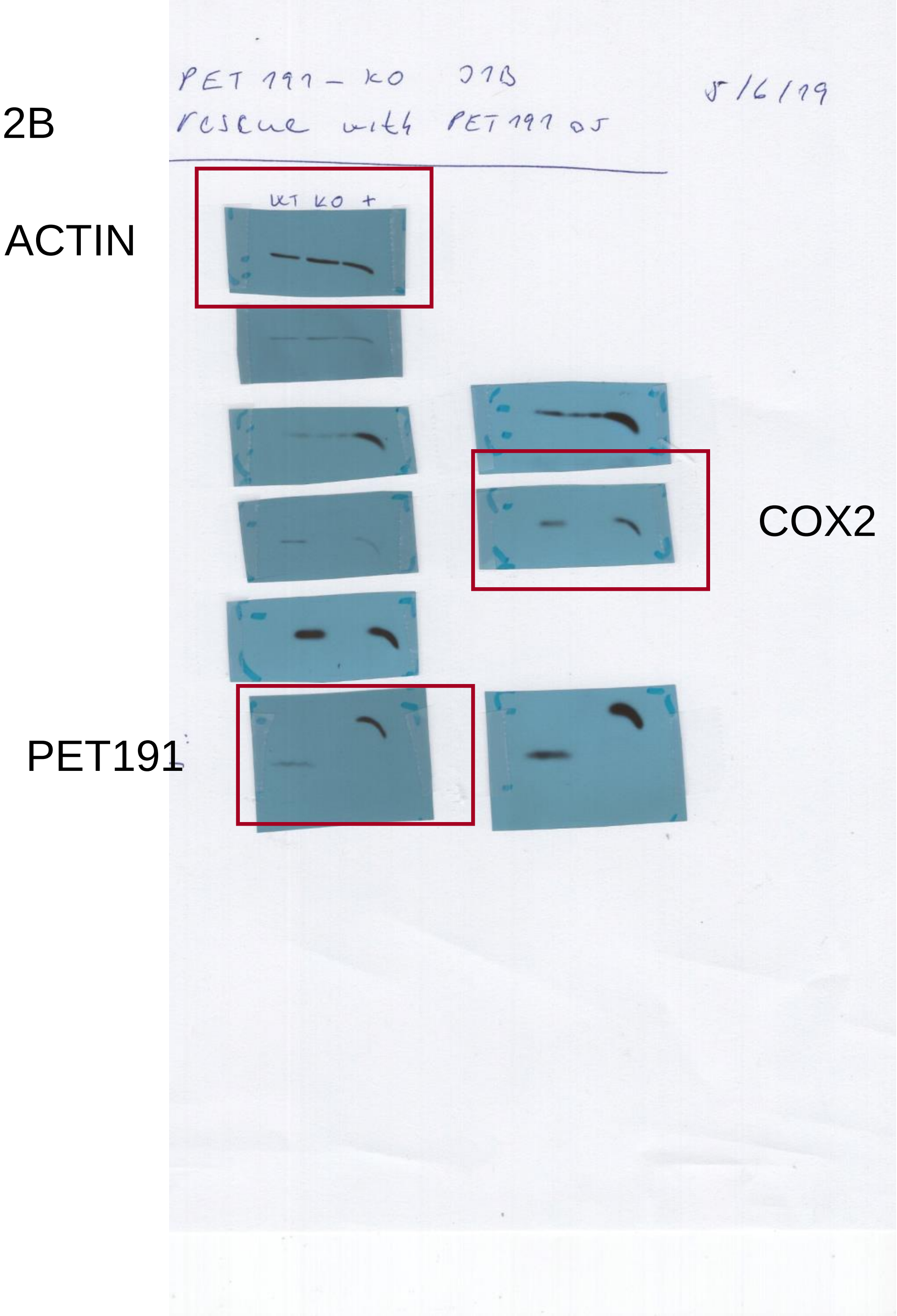

Supplementary Fig. 2B

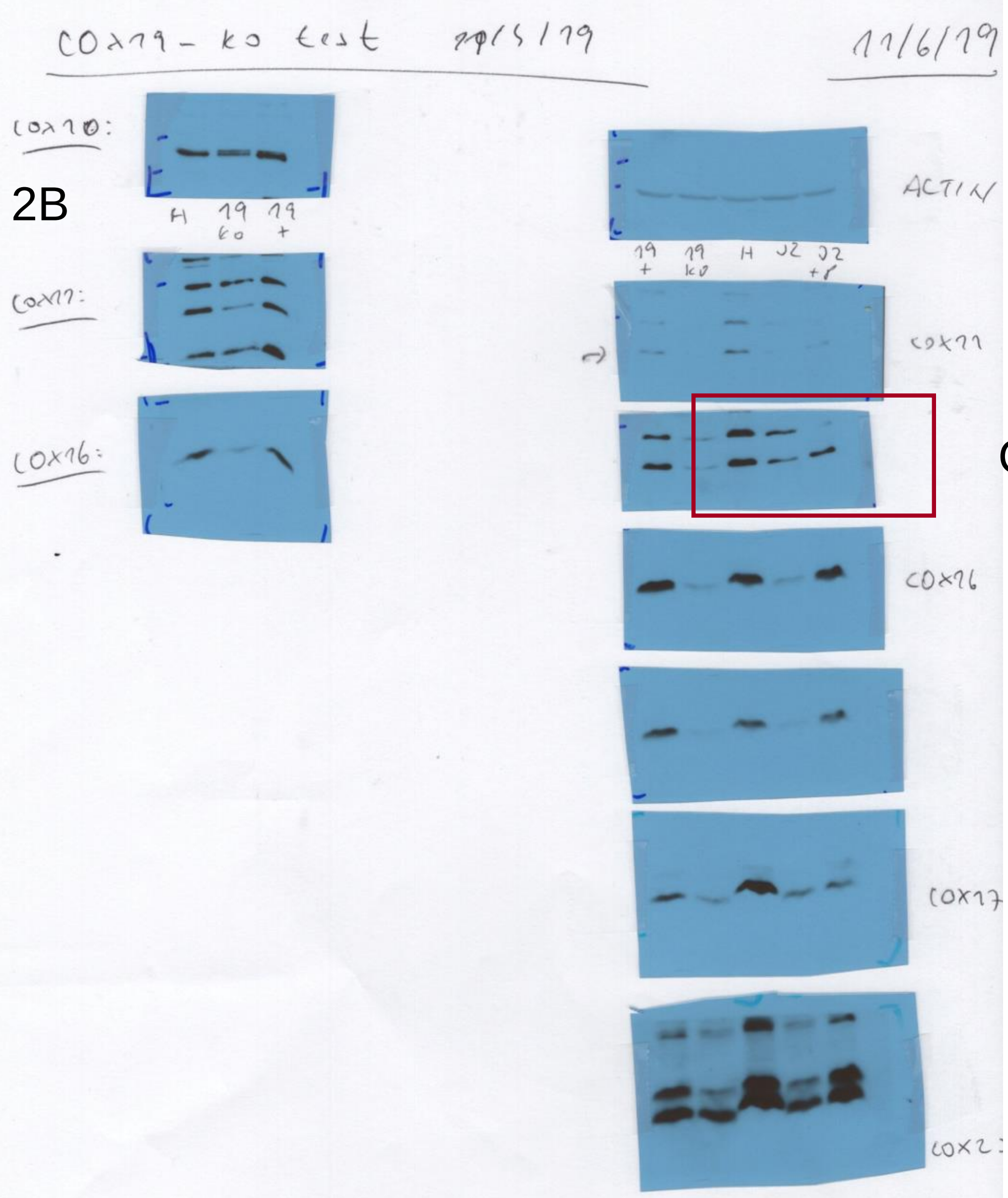

COX11

Supplementary Fig. 2B

COX1

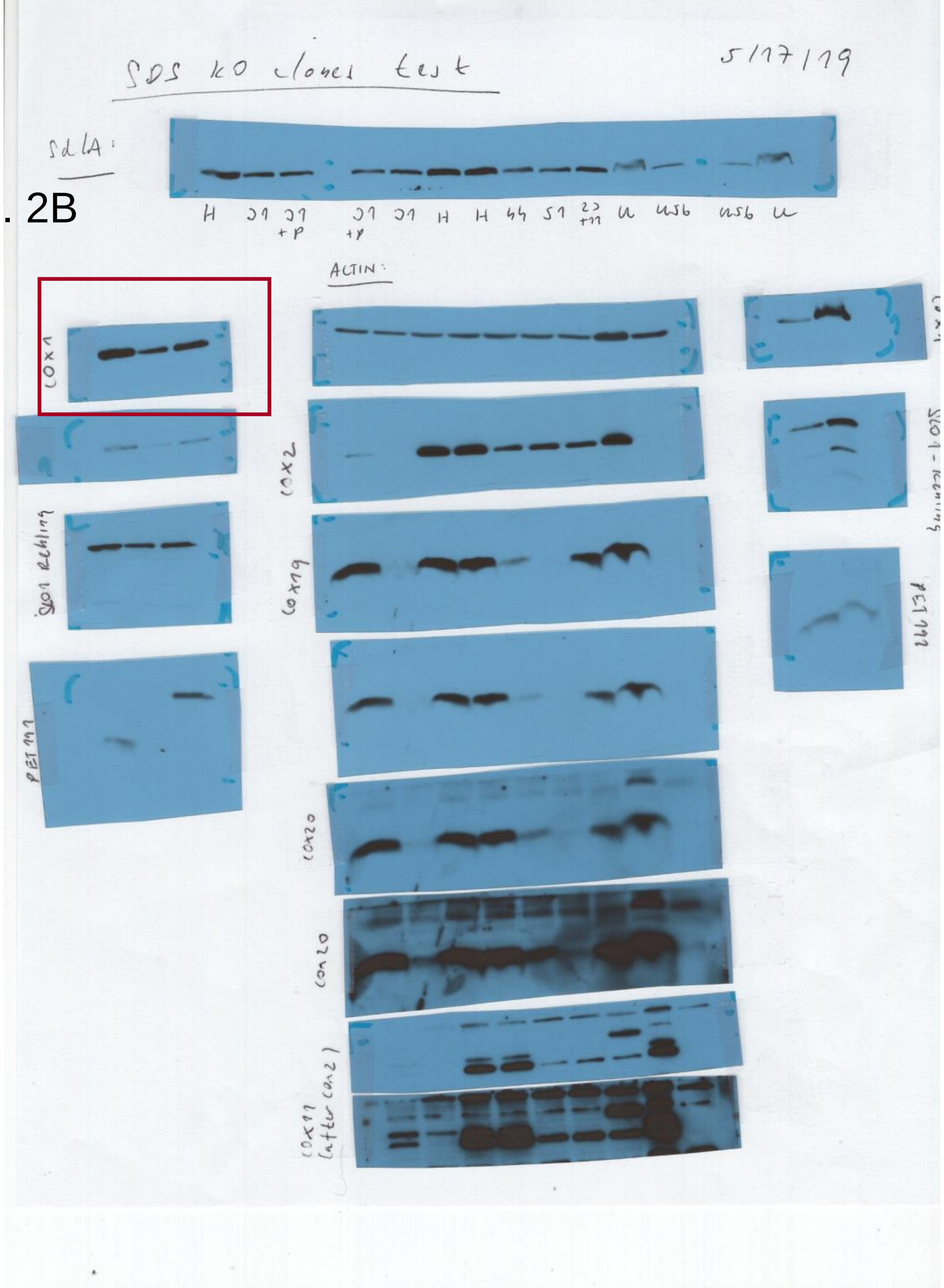

Supplementary Fig. 2B

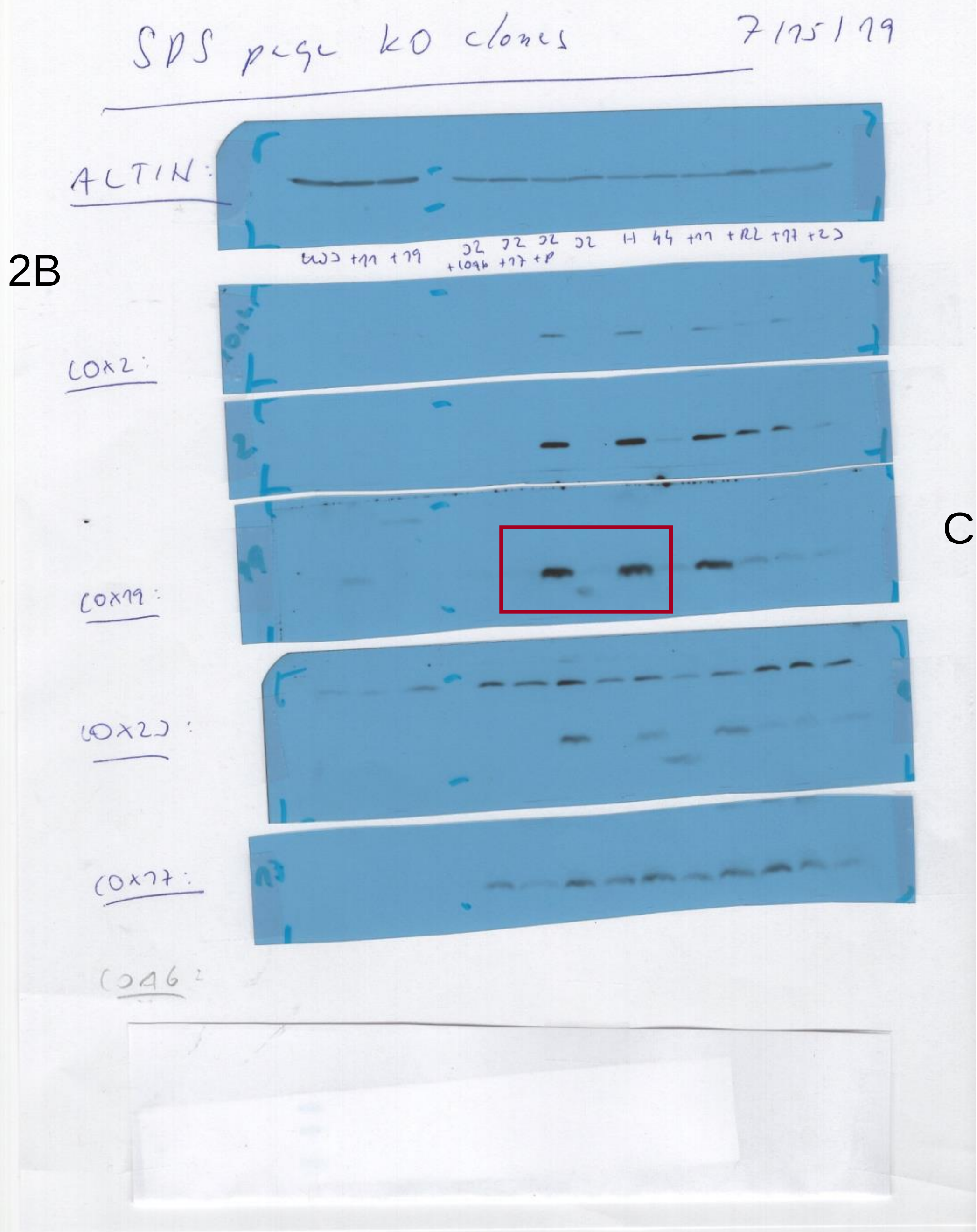

COX19

Supplementary Fig. 2B

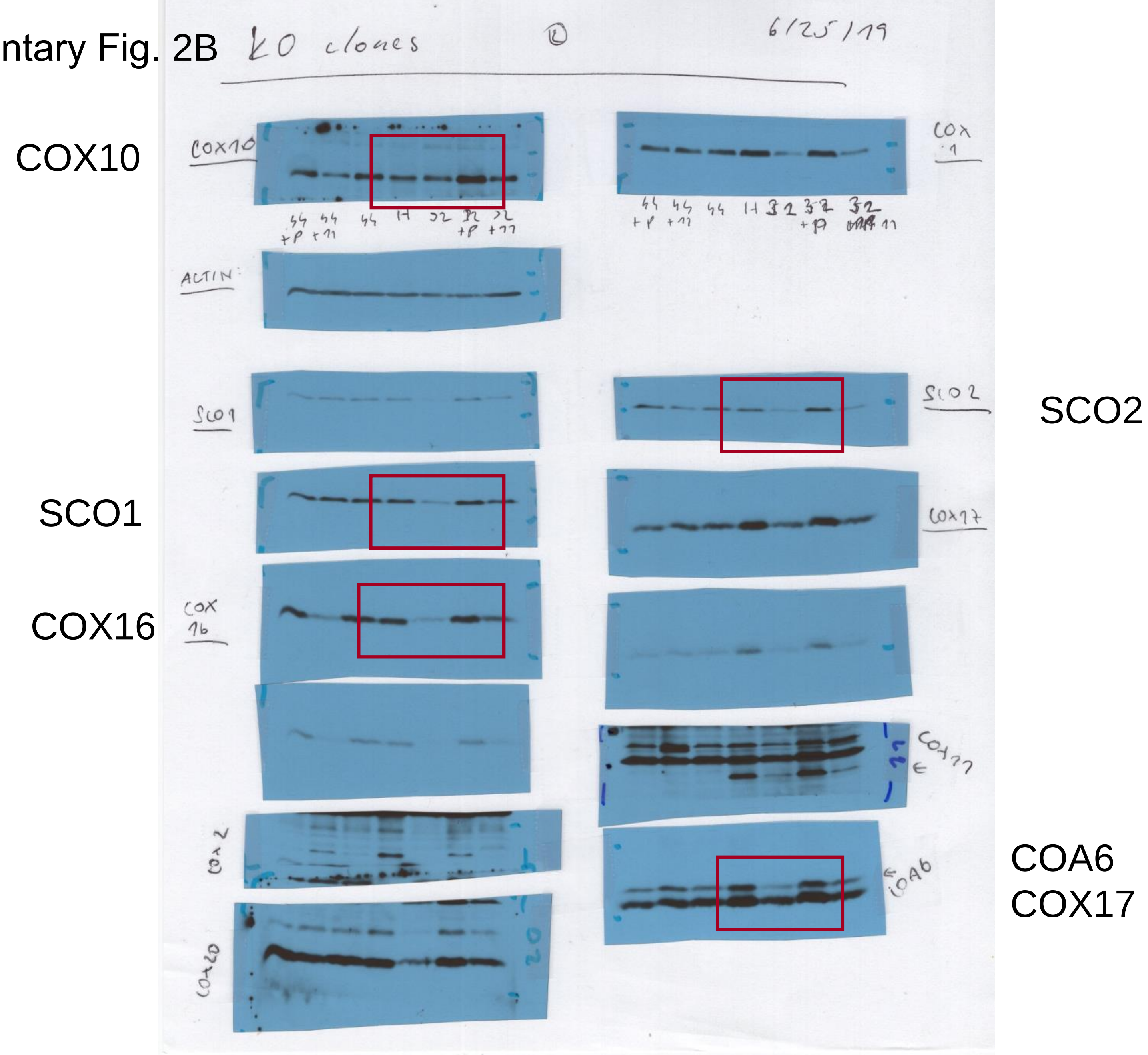

Supplementary Fig. 2B

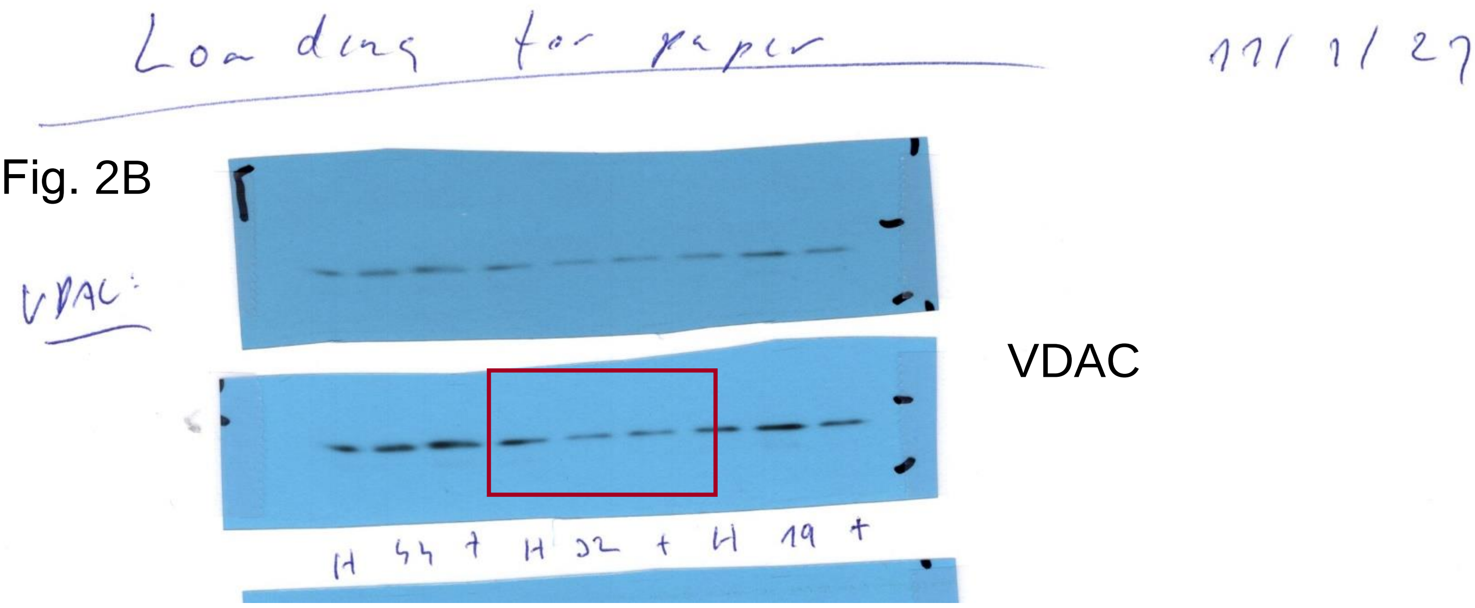

VDAC

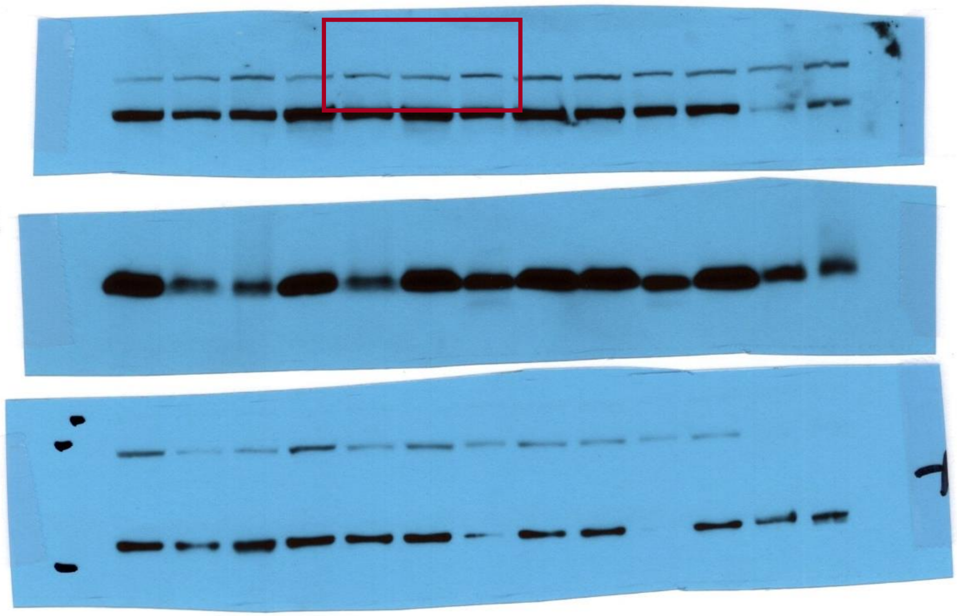

TOM20

Supplementary Fig. 2D

FLAG

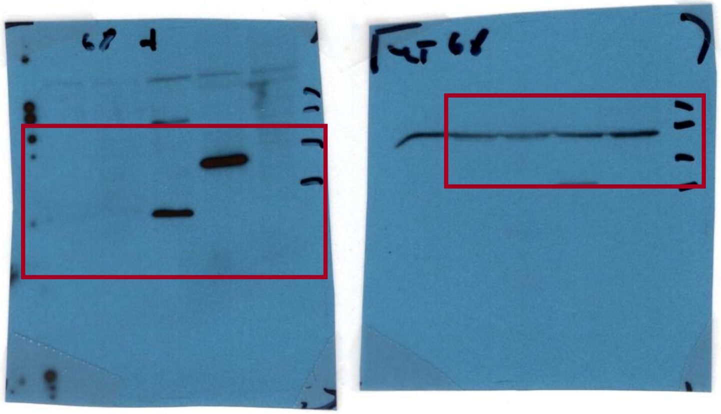

ACTIN

+11 +19 ko HEK

COX2

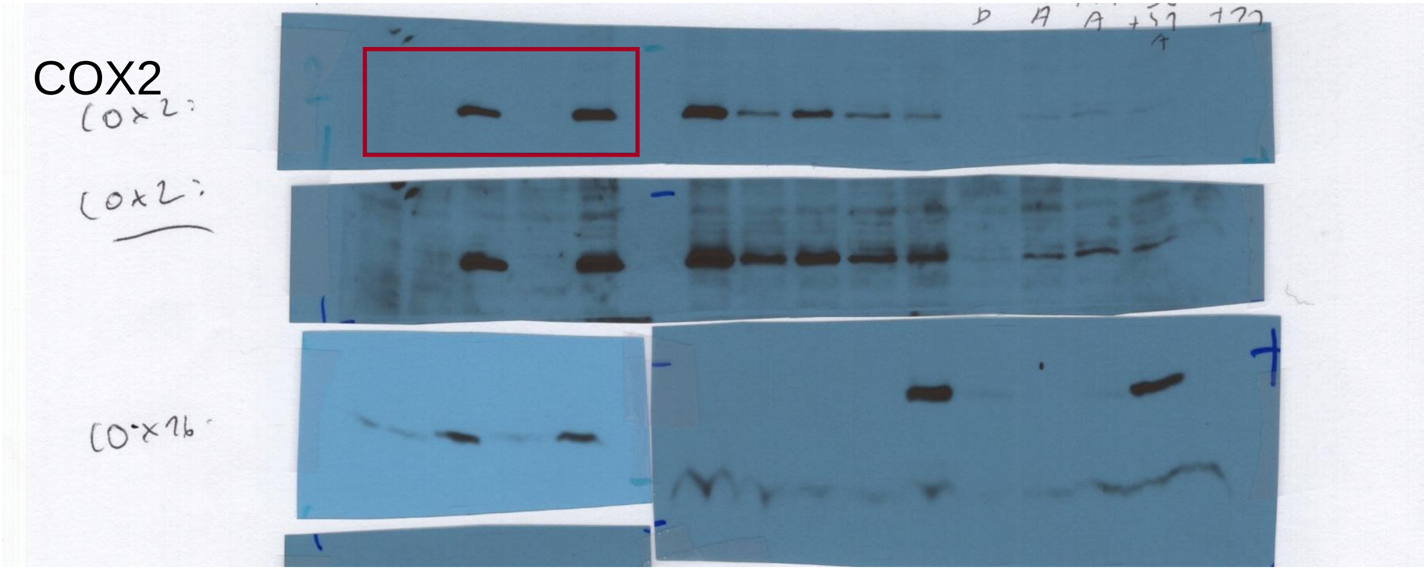

Supplementary Fig. 2D

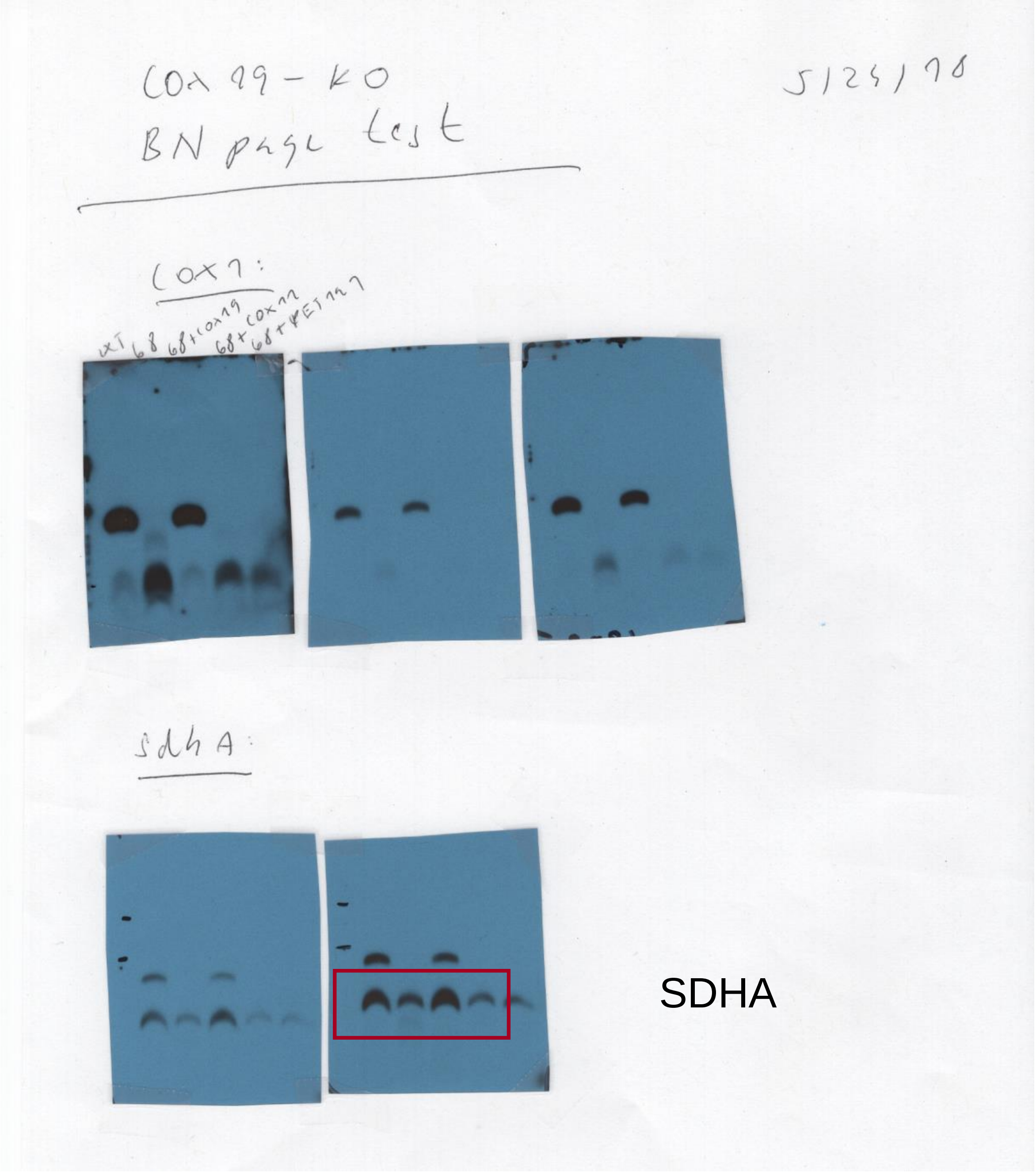

SDHA

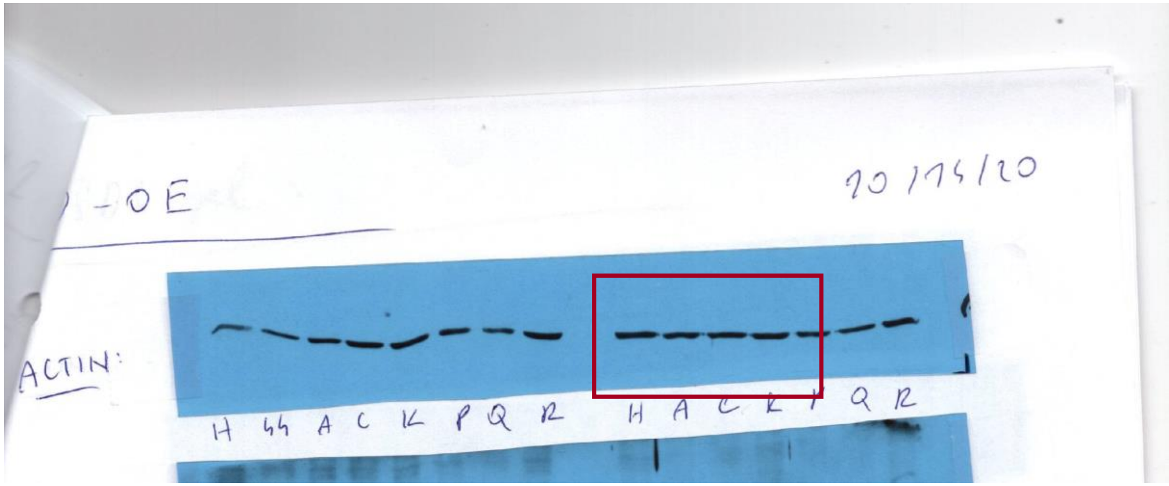

ACTIN

Supplementary Fig. 2F

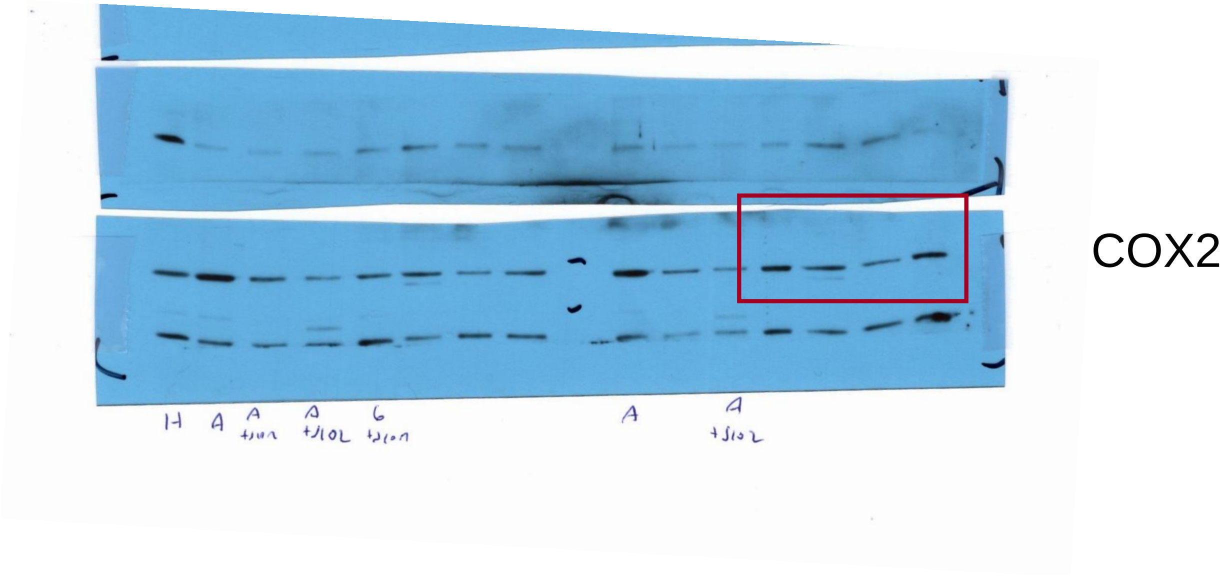

COX2

Supplementary Fig. 2F

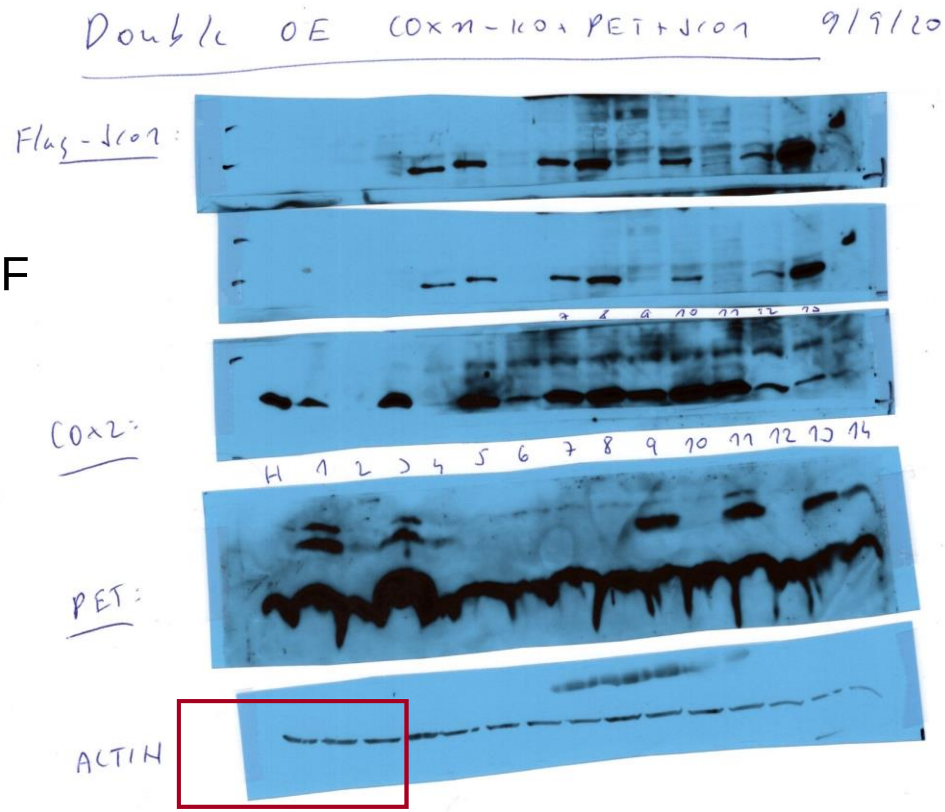

ACTIN

Supplementary Fig. 3B
=Supplementary Fig. 5G

HA

COX11

Porin

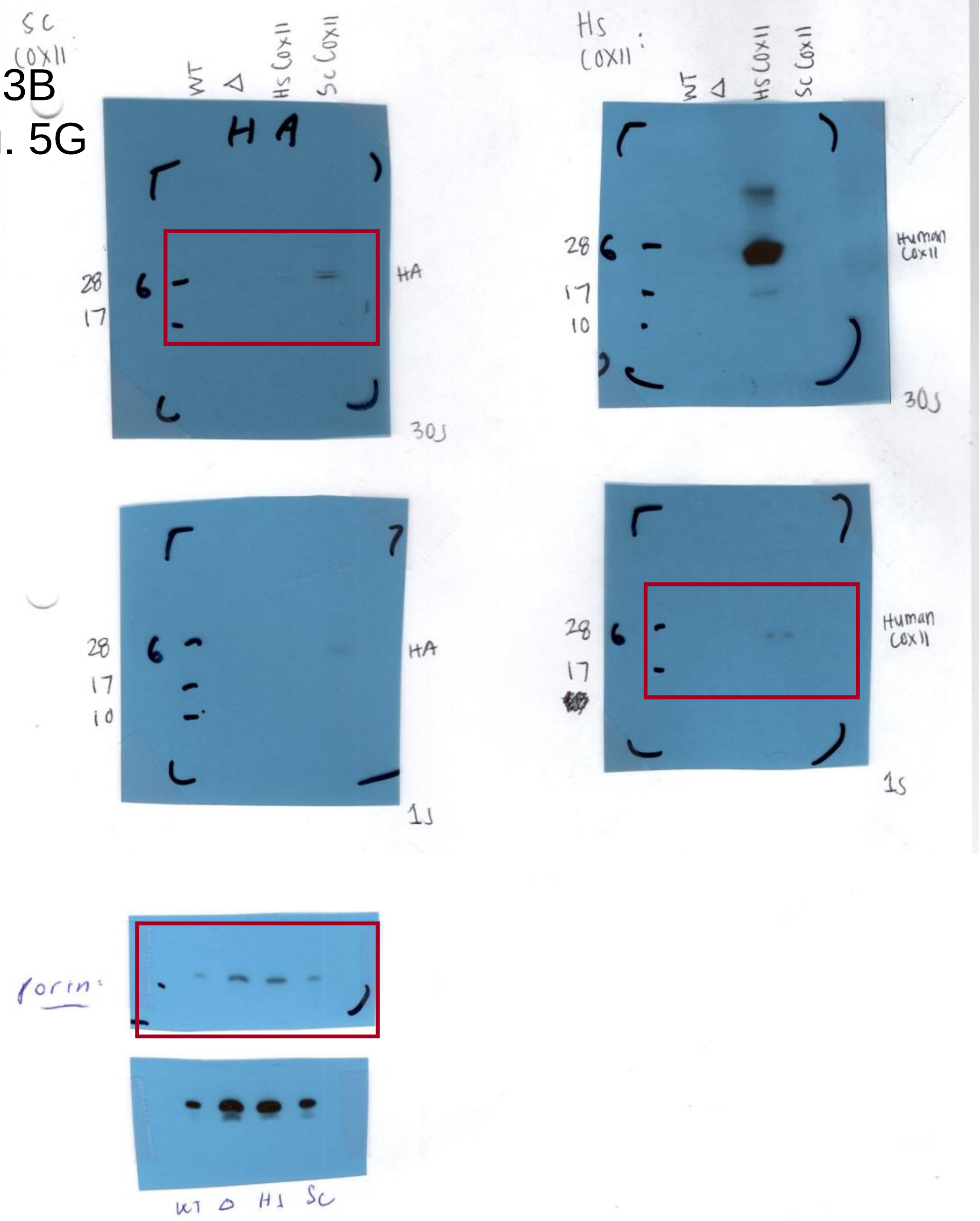

Supplementary Fig. 3C = Supplementary Fig. 5H

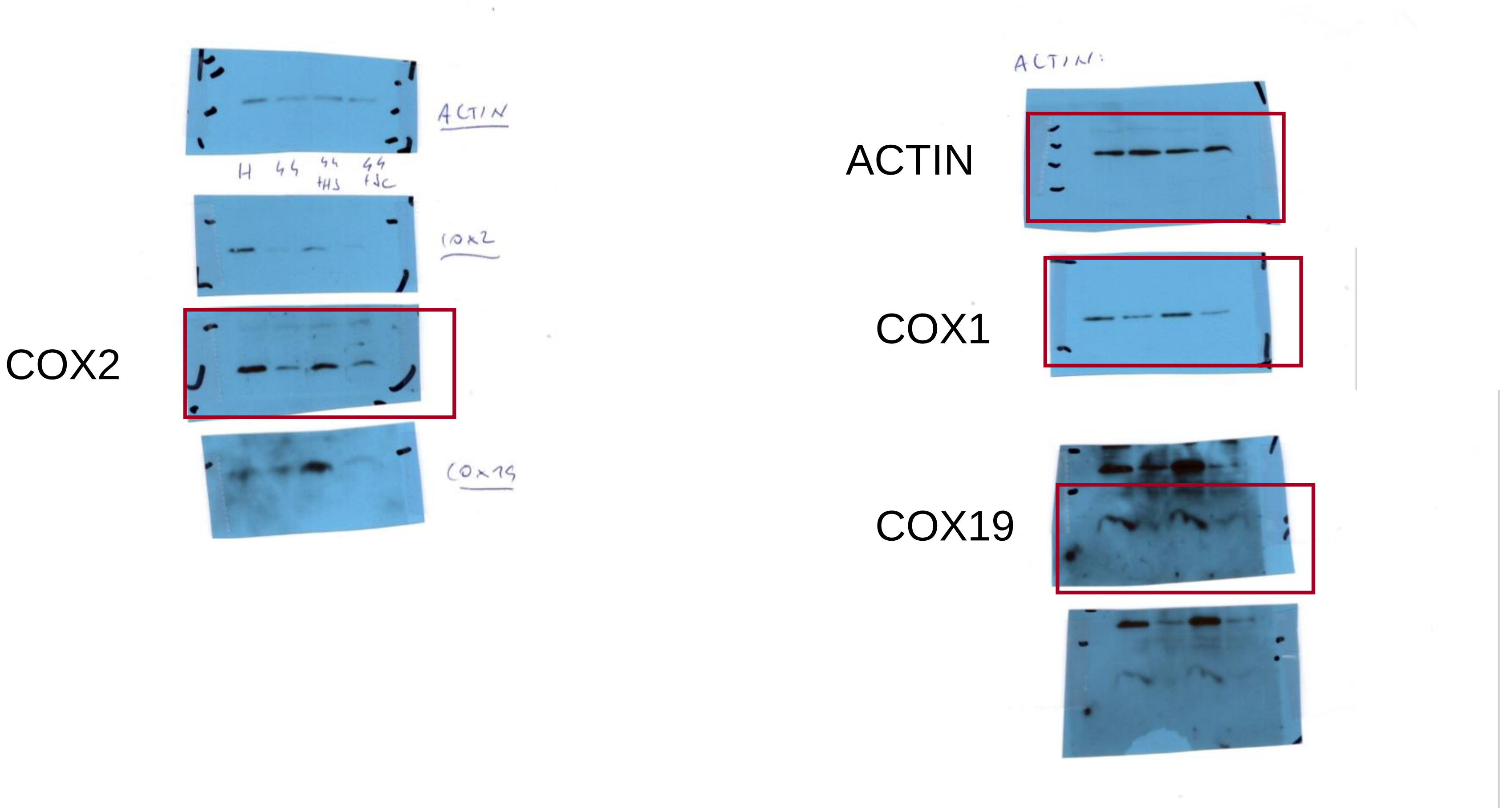

Supplementary Fig. 4A

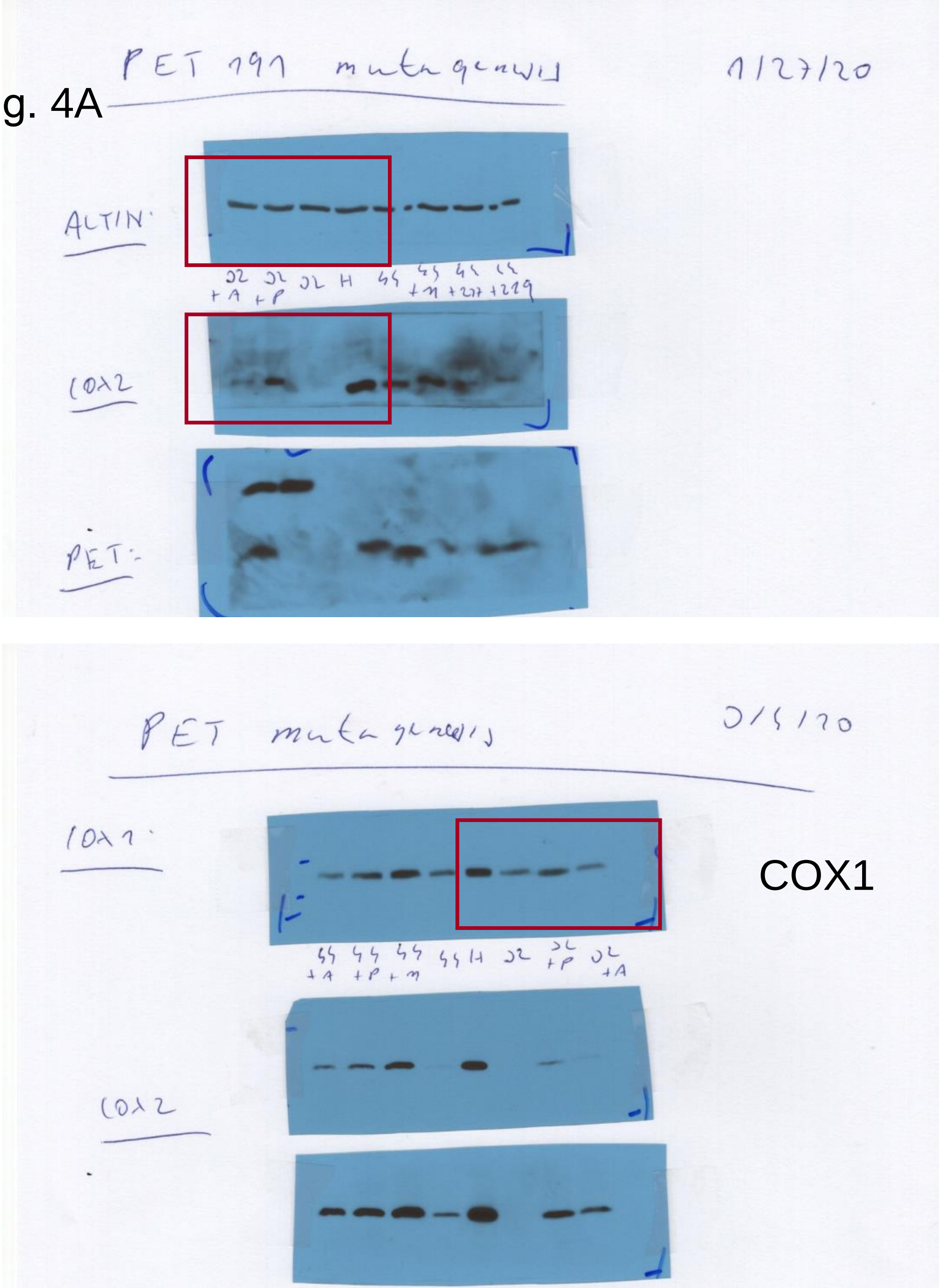

Supplementary Fig. 4A

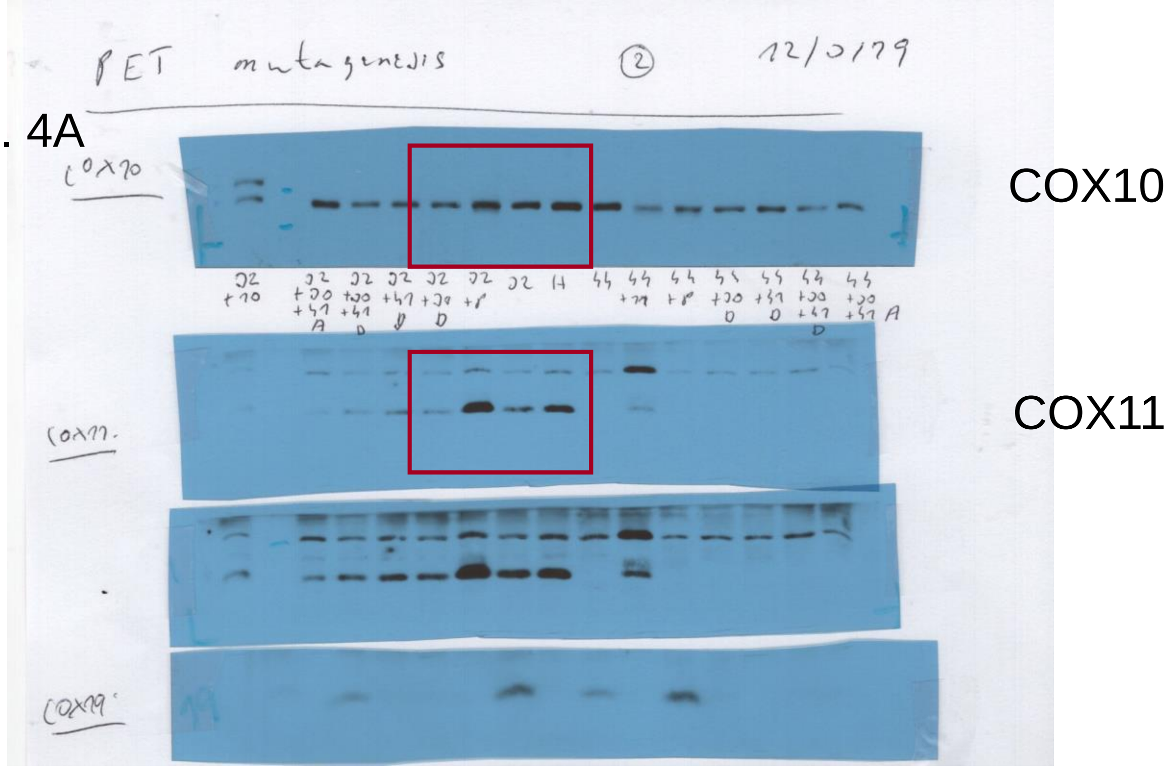

Supplementary Fig. 4D

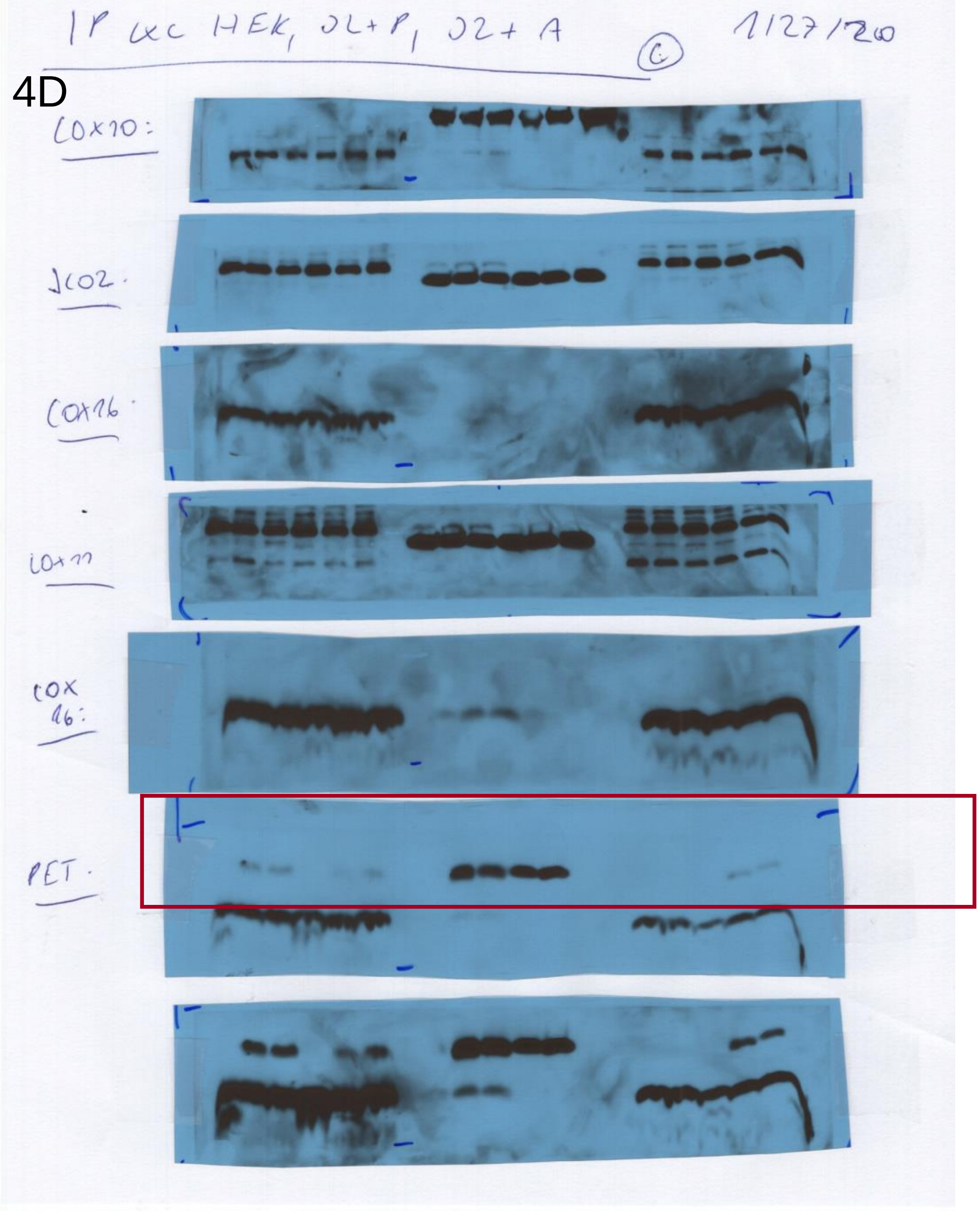

Supplementary Fig. 4D

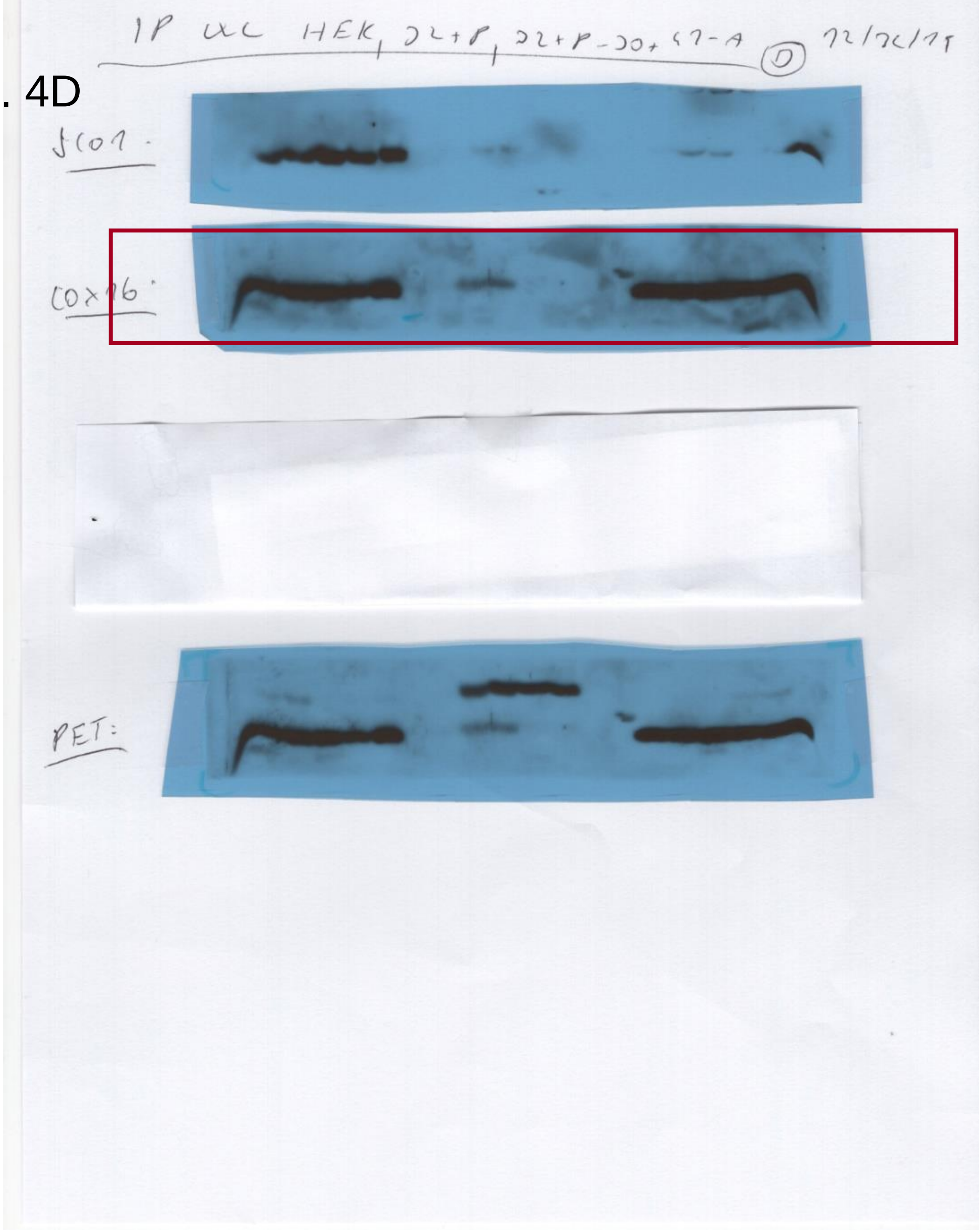

COX16

Supplementary Fig. 4D

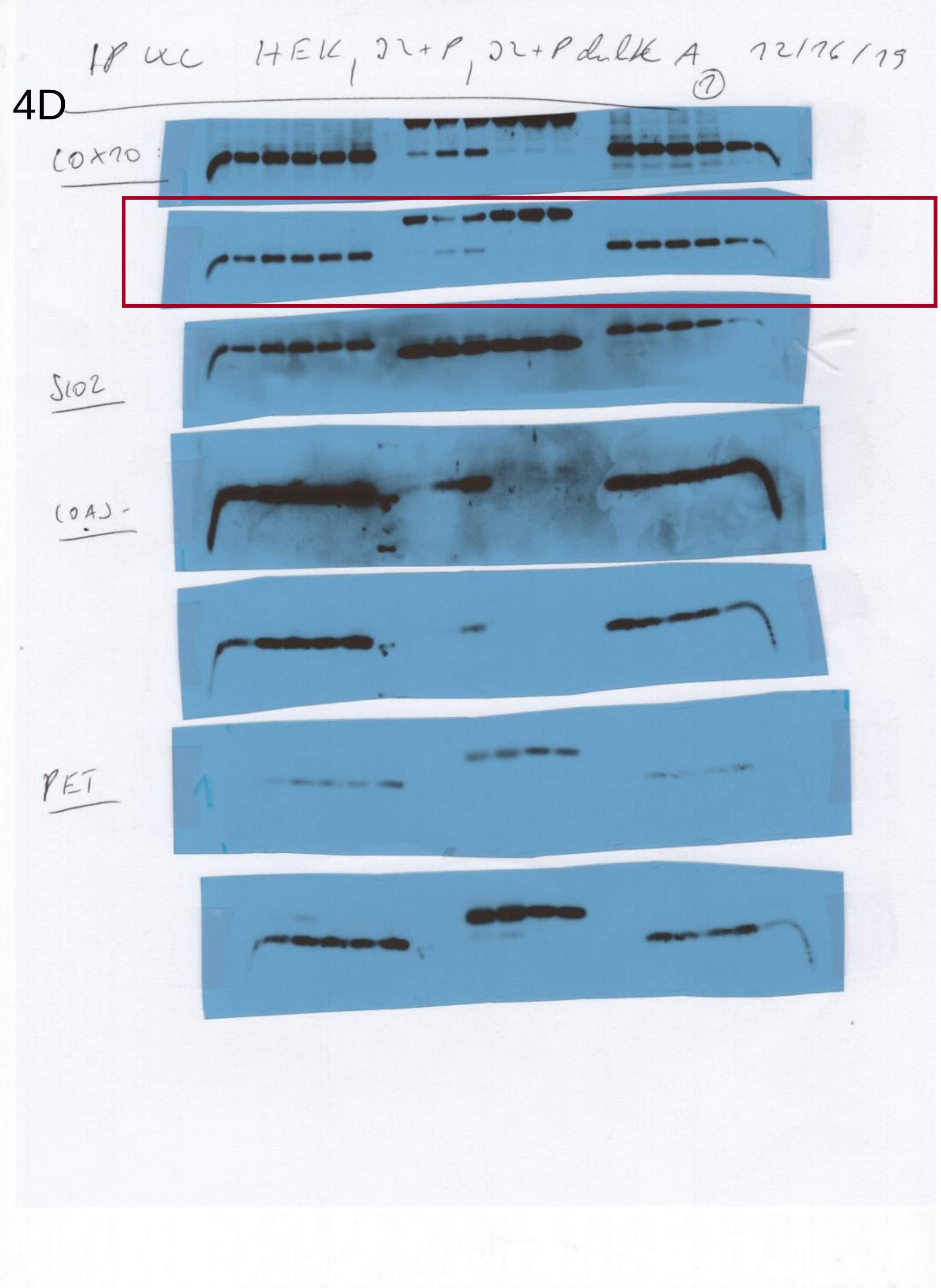

COX10

Supplementary Fig. 4D

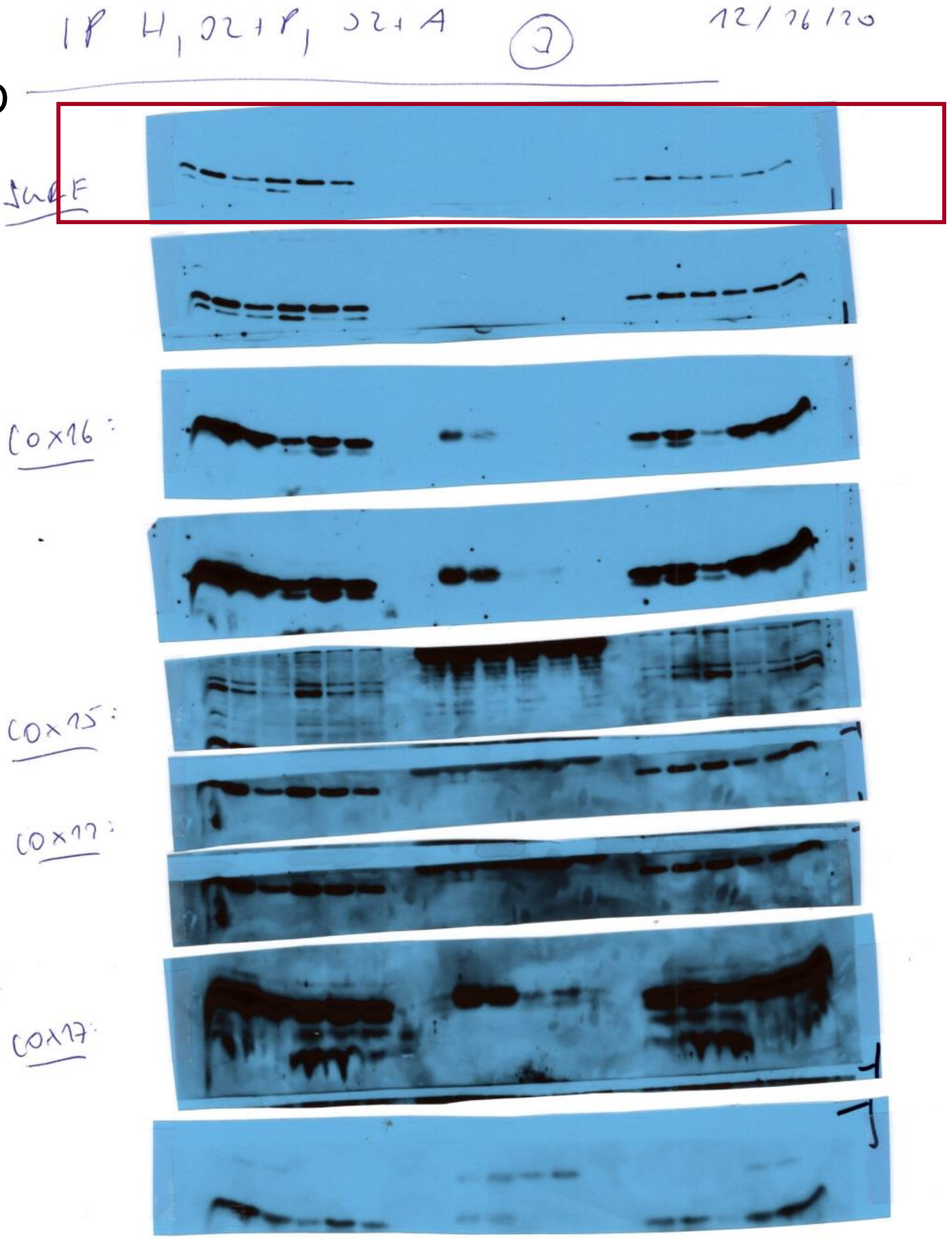

SURF1

Supplementary Fig. 4D

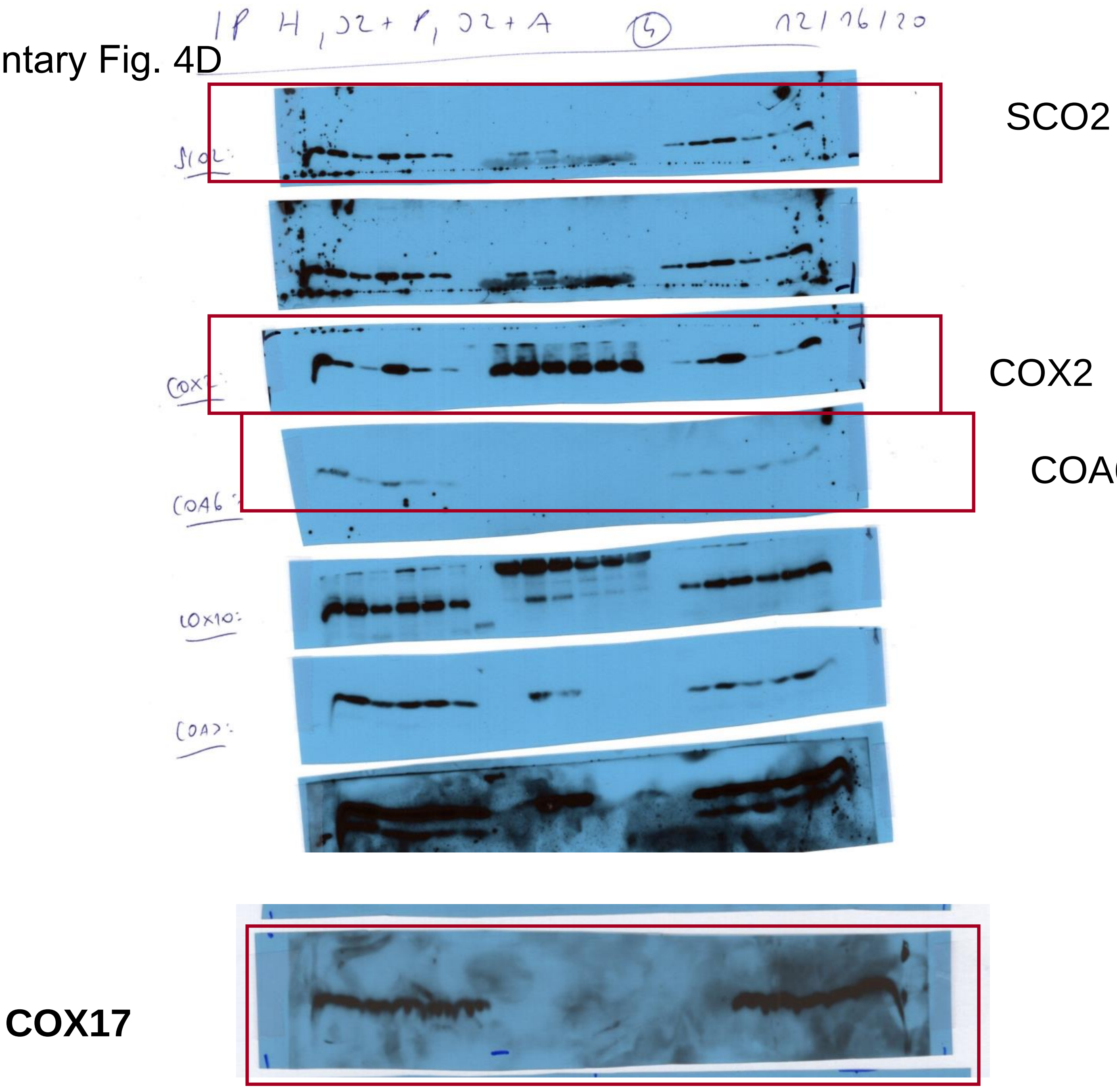

Supplementary Fig. 4D

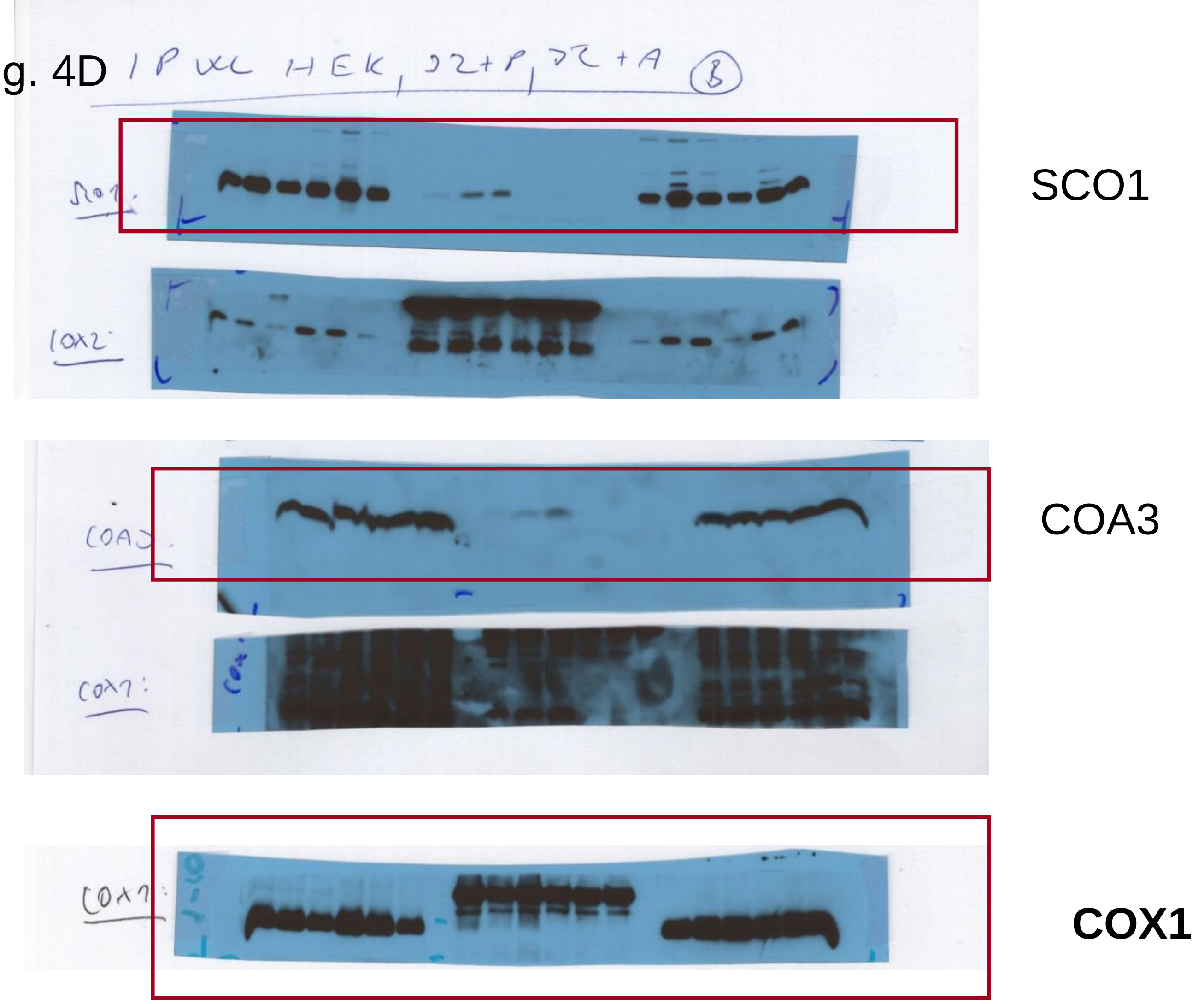

Supplementary Fig. 4D

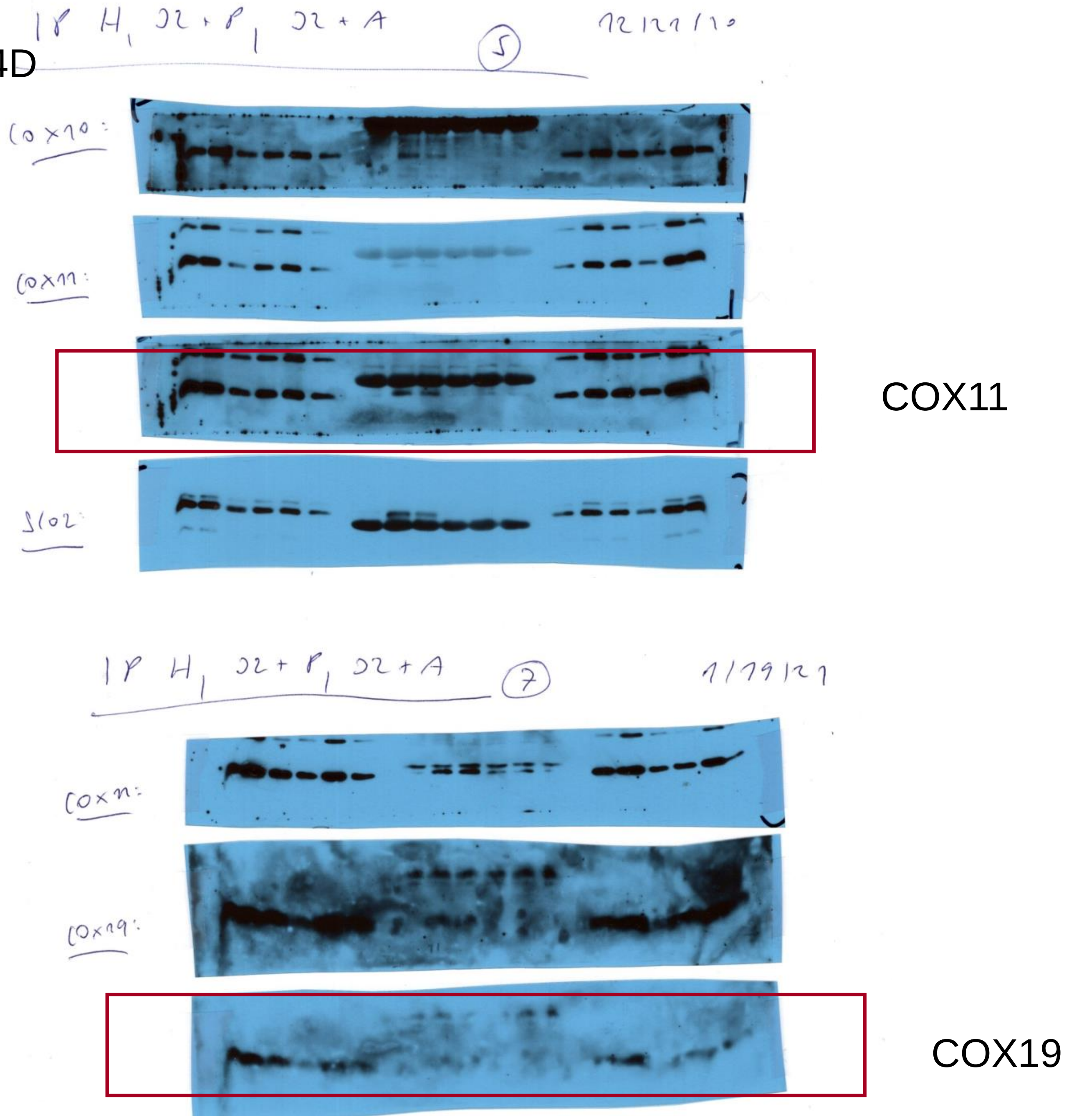

Supplementary Fig. 4E

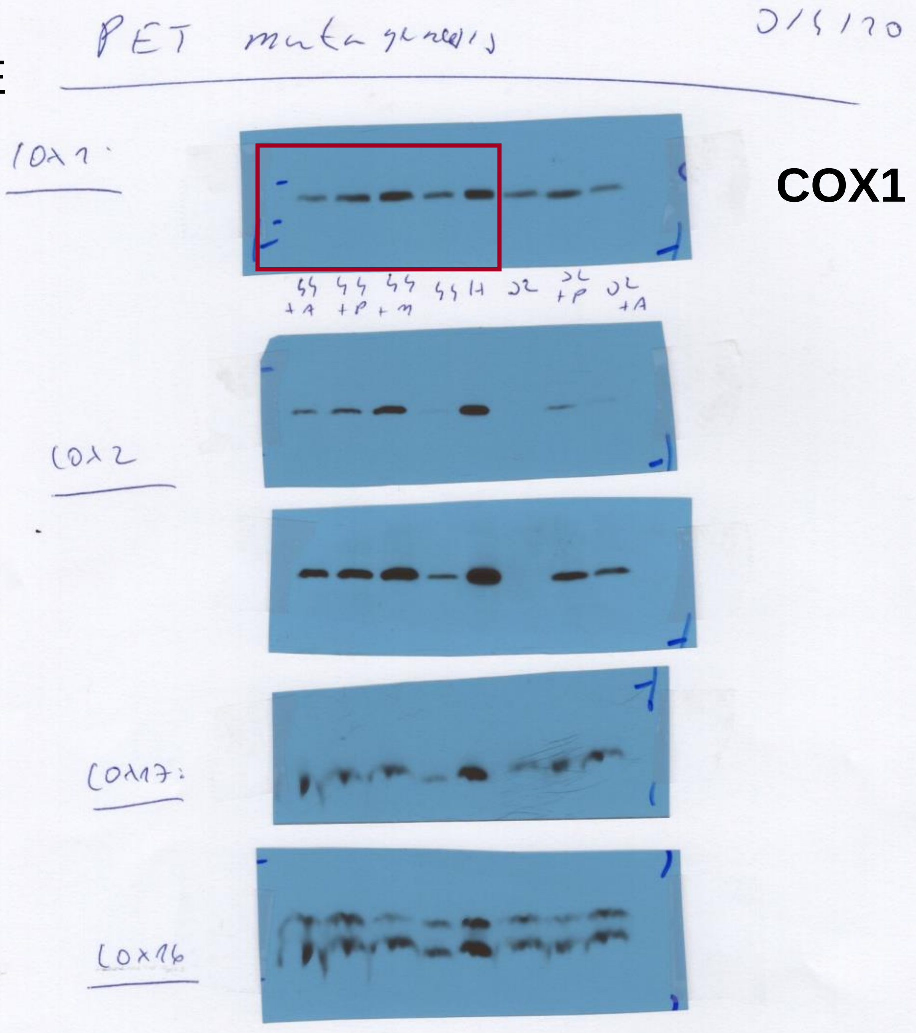

COX1

Supplementary Fig. 4E

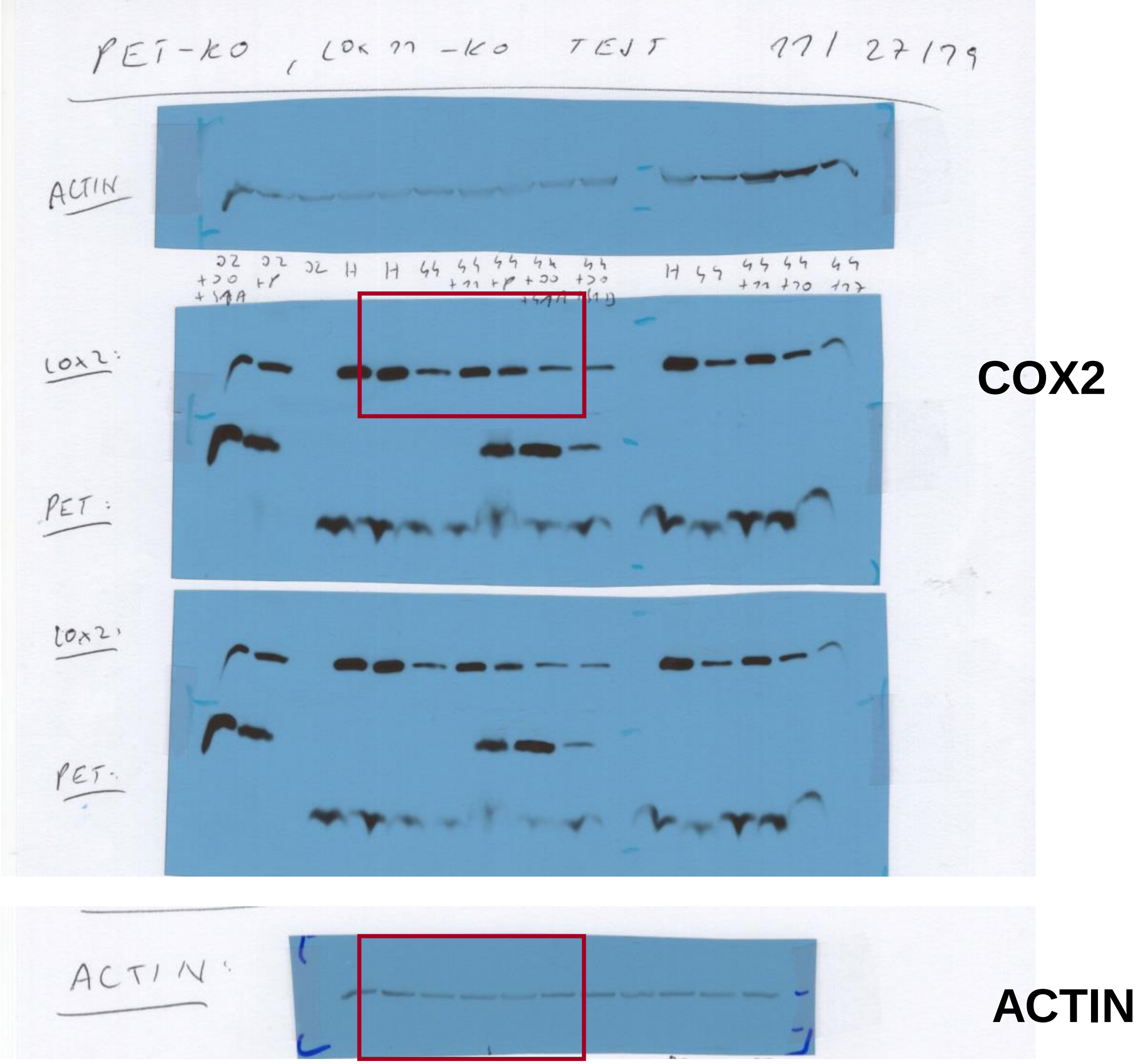

COX2

ACTIN

Supplementary Fig. 4F

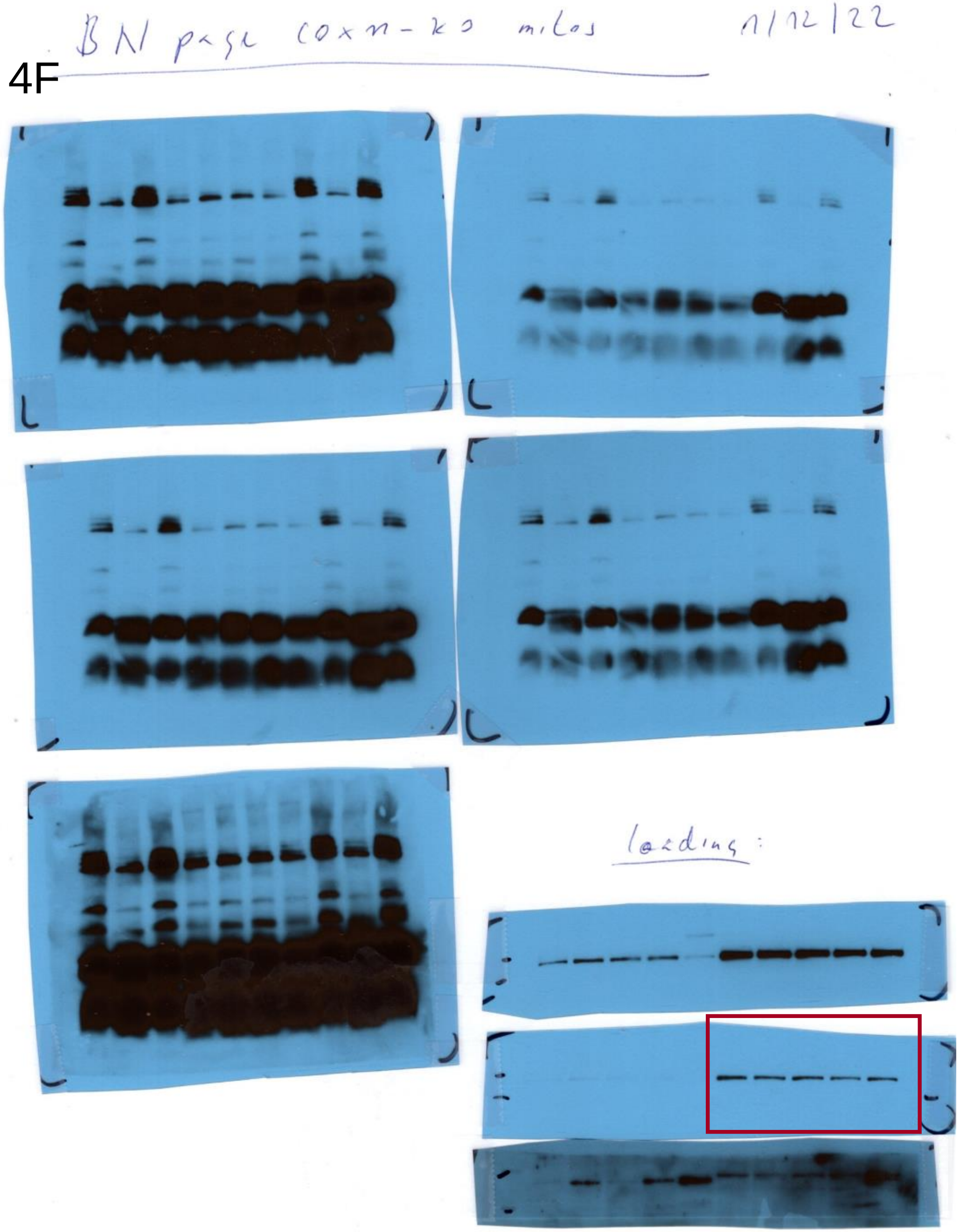

ACTIN

Supplementary Fig.4F

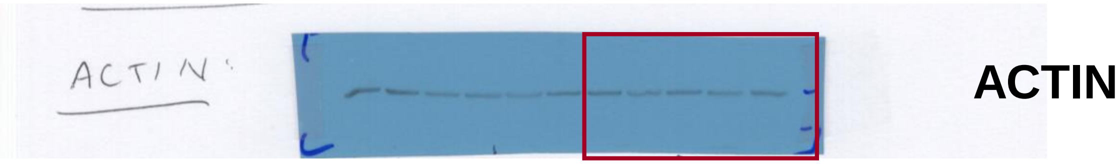

ACTIN

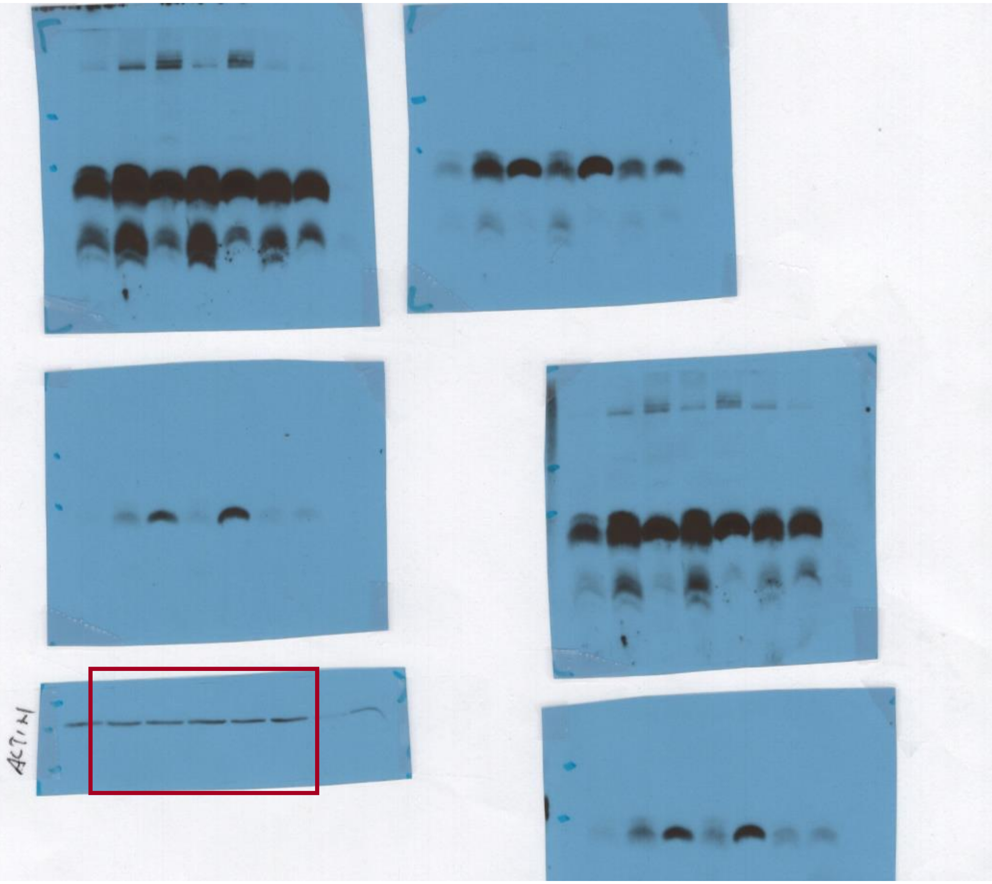

ACTIN

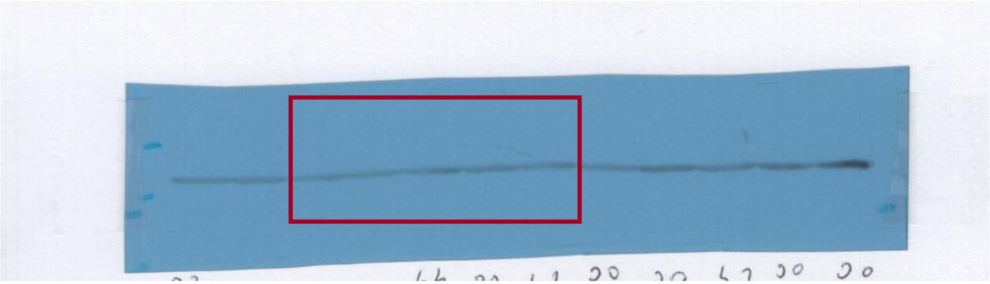

ACTIN

Supplementary Fig. 4H

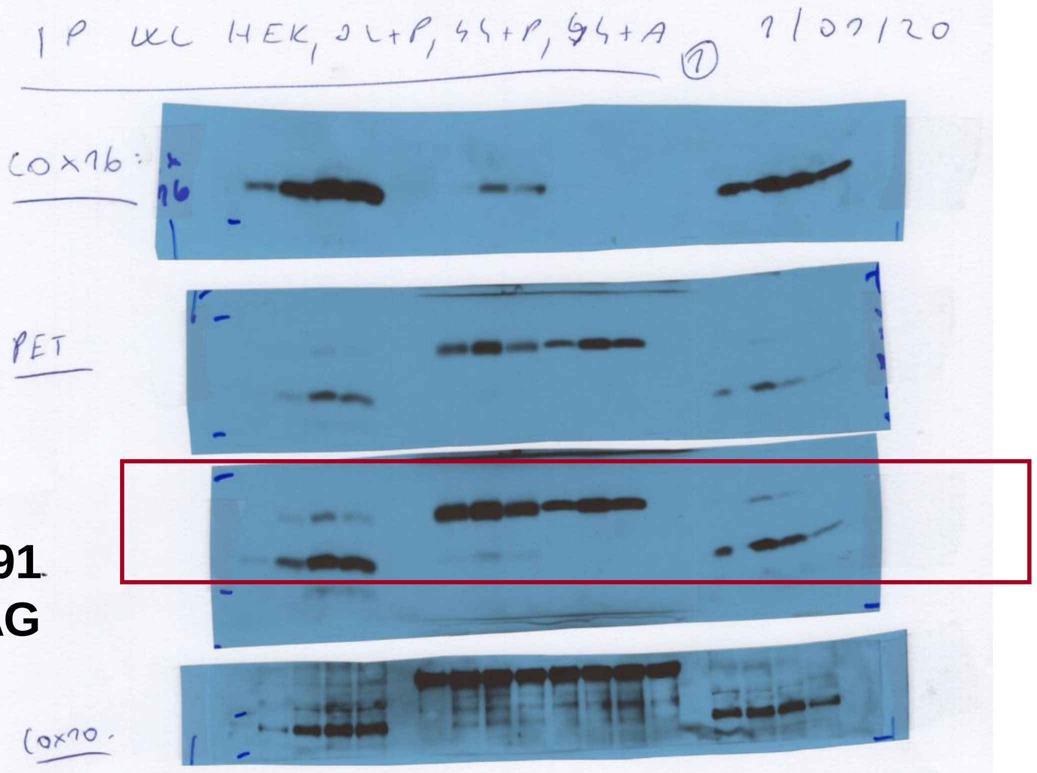

PET191 - FLAG

Supplementary Fig. 4H

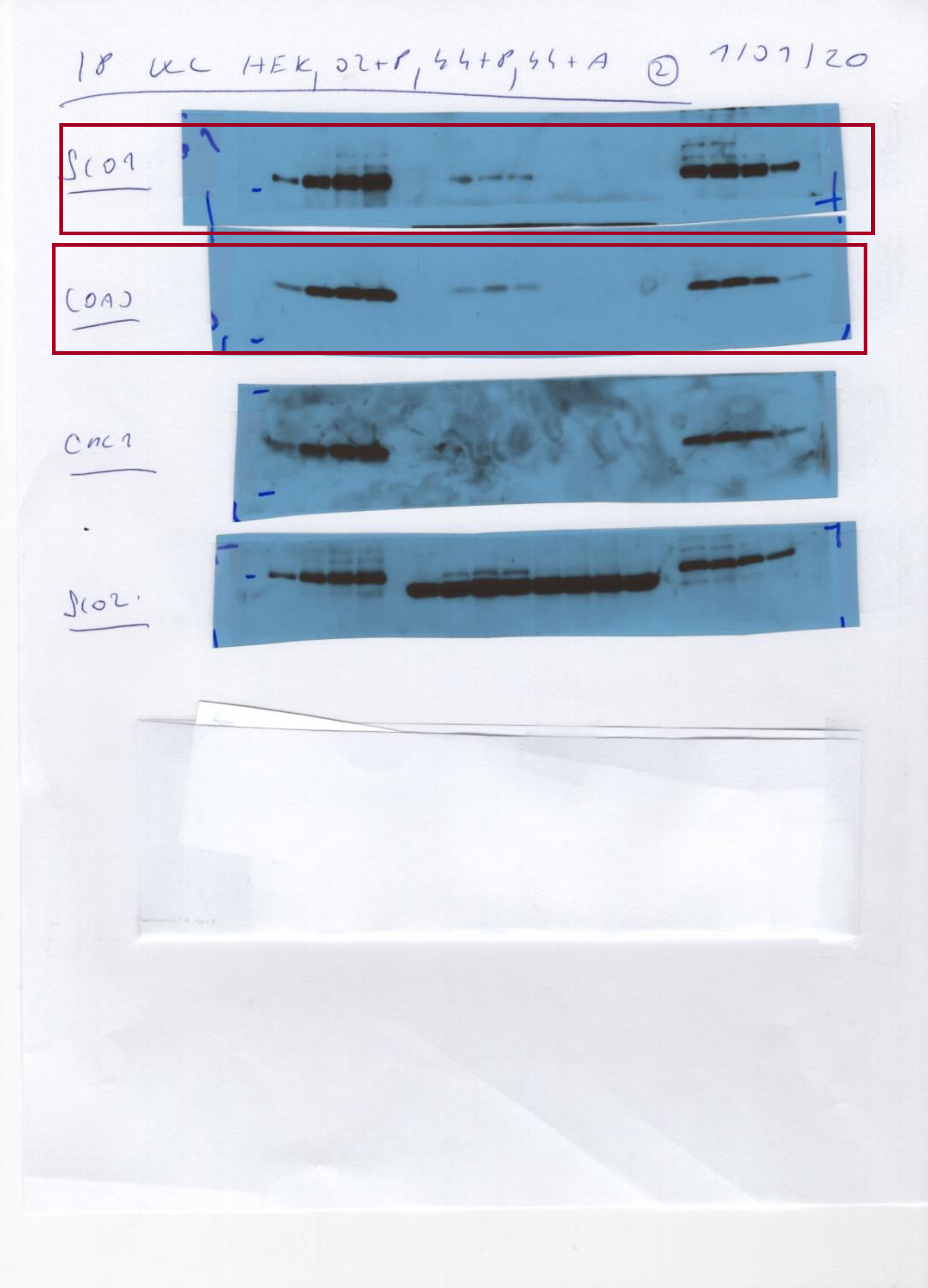

SCO1

COA3

Supplementary Fig. 4H

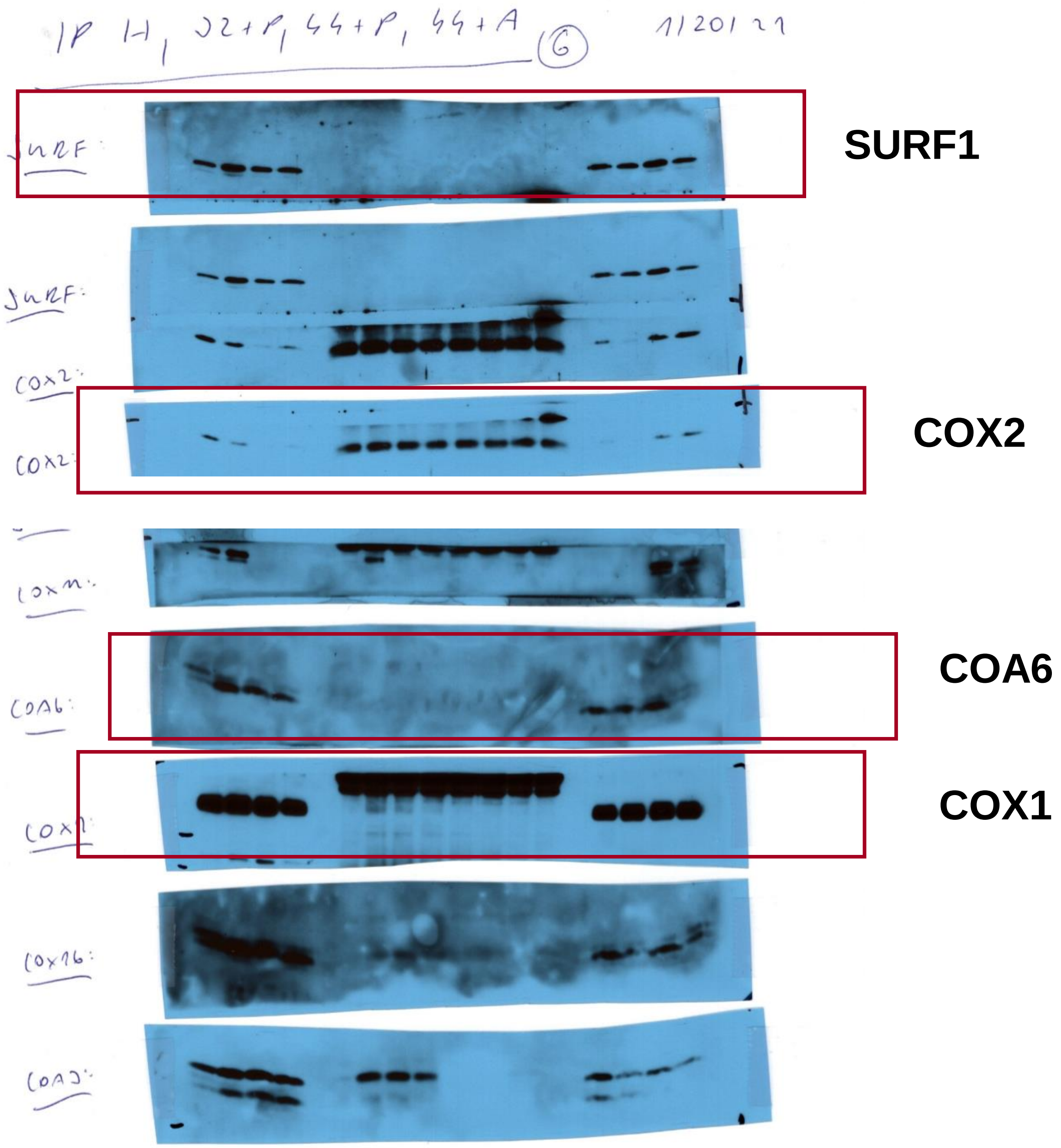

Supplementary Fig. 4H

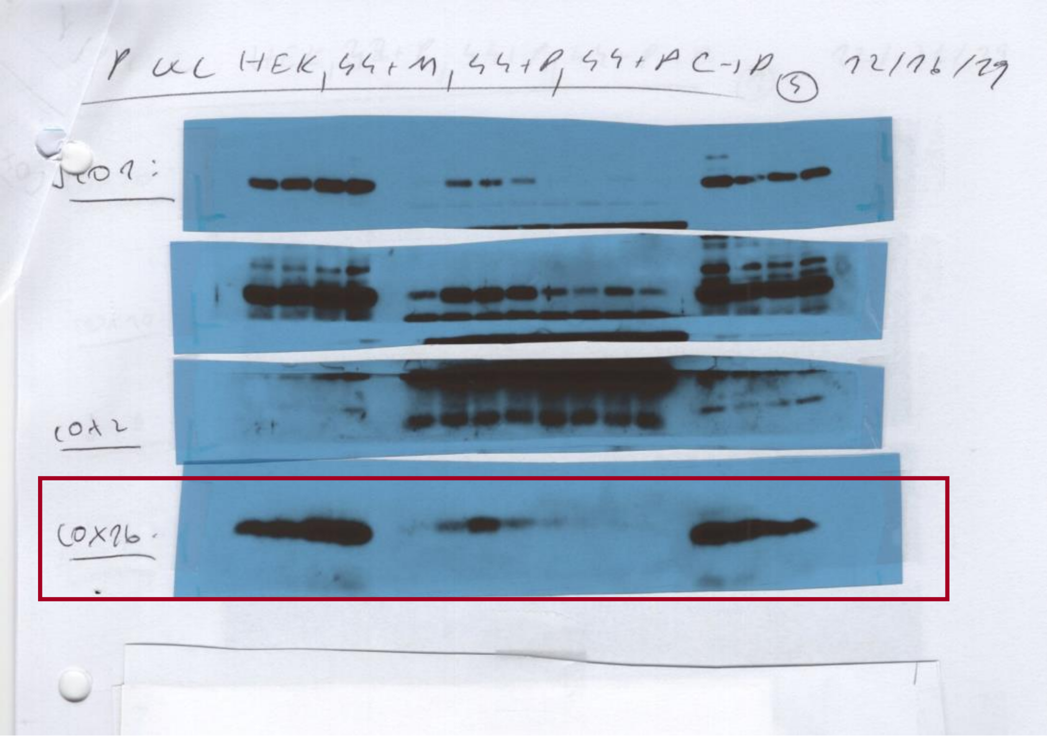

COX16

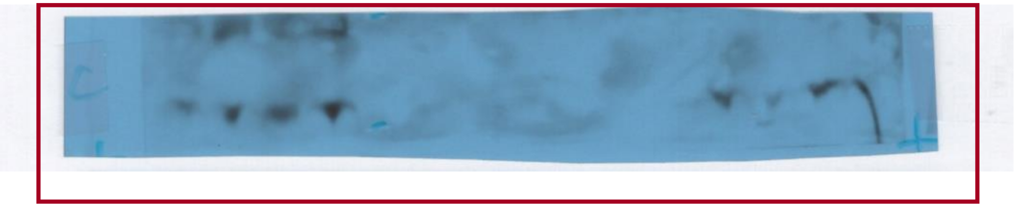

COX19

Supplementary Fig. 4H

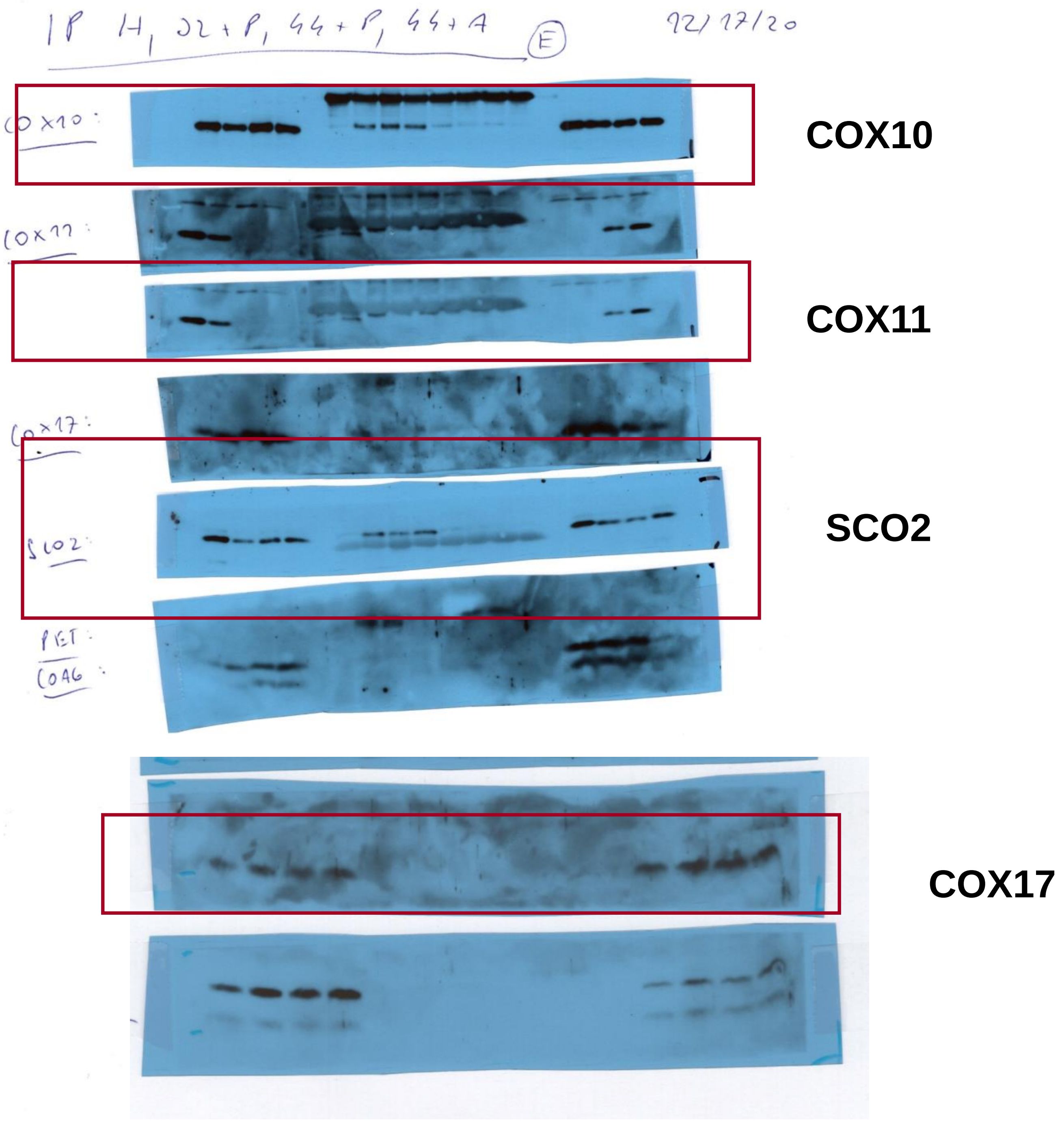

Supplementary Fig. 7A

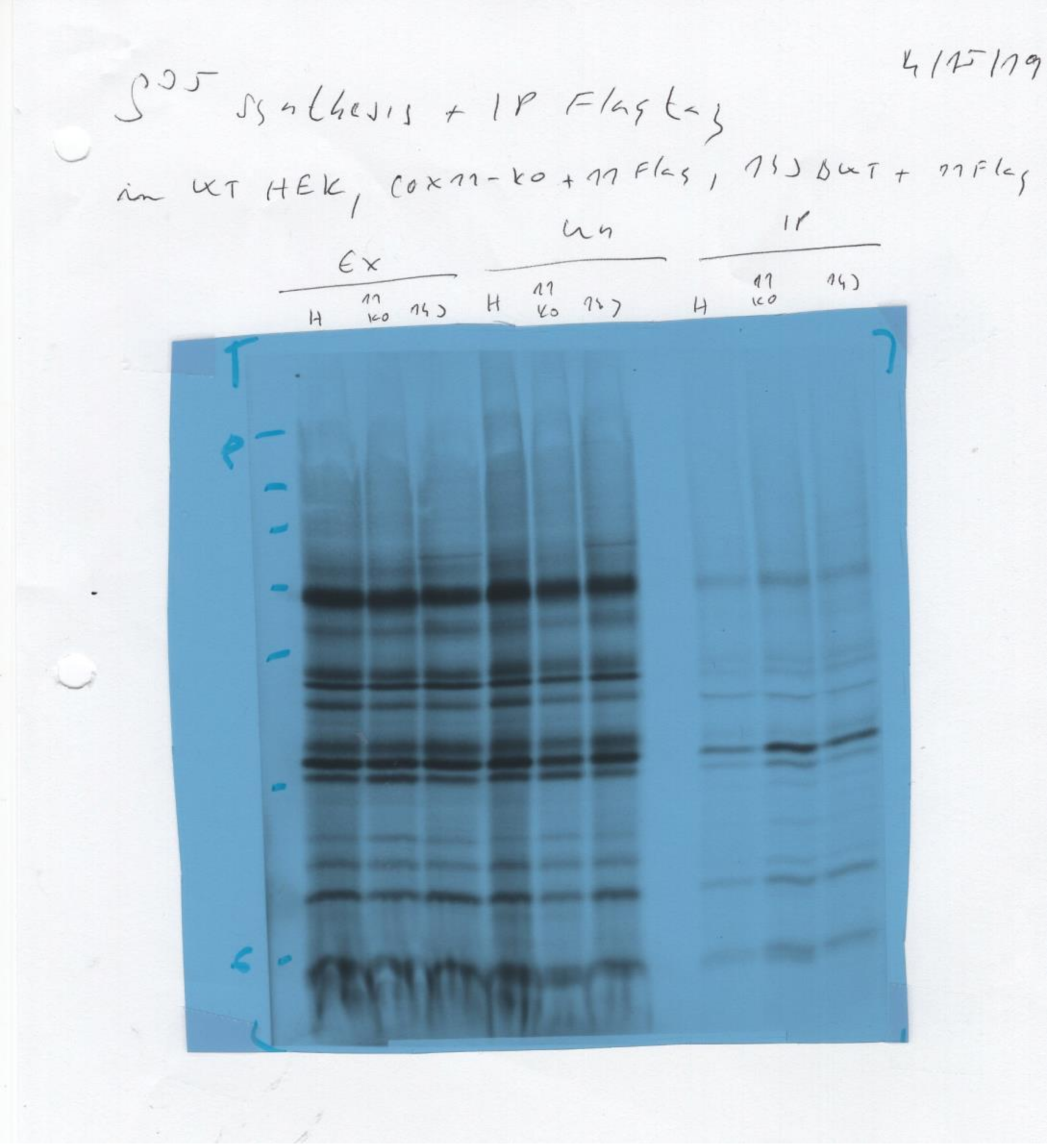

Supplementary Fig. 7A

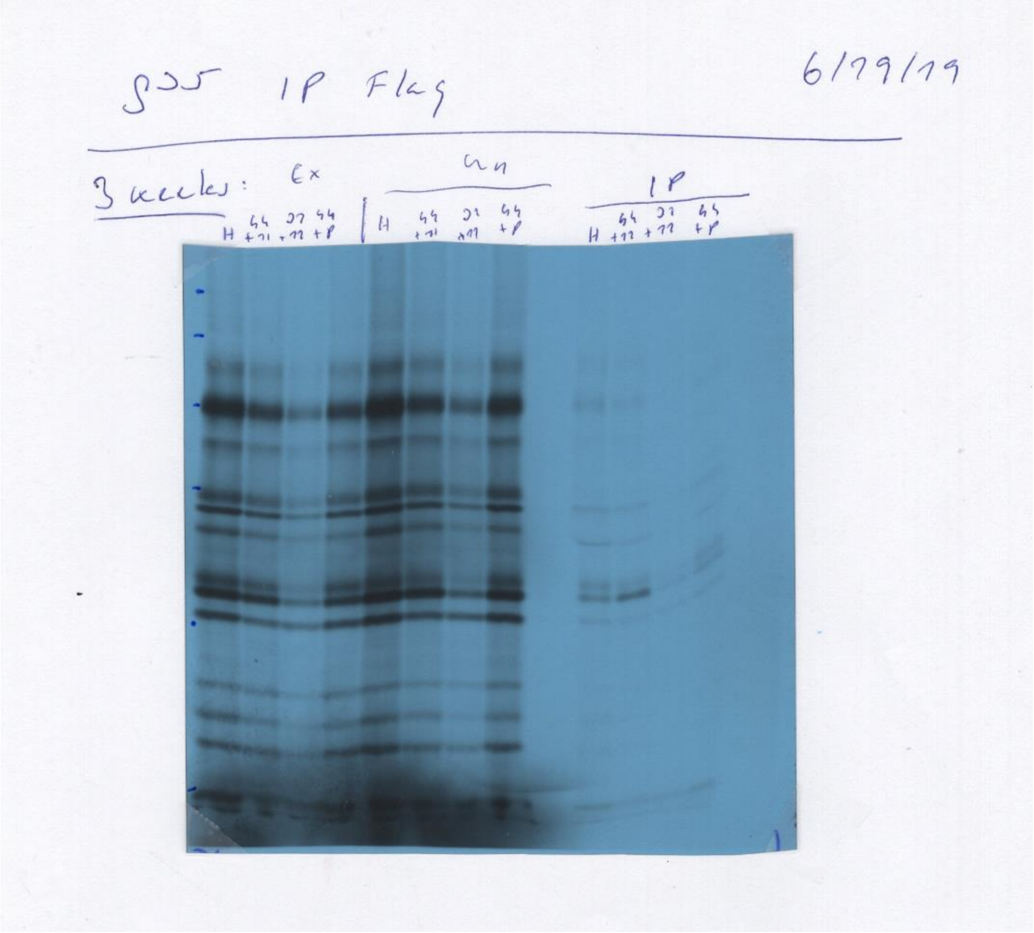

Supplementary Fig. 7A

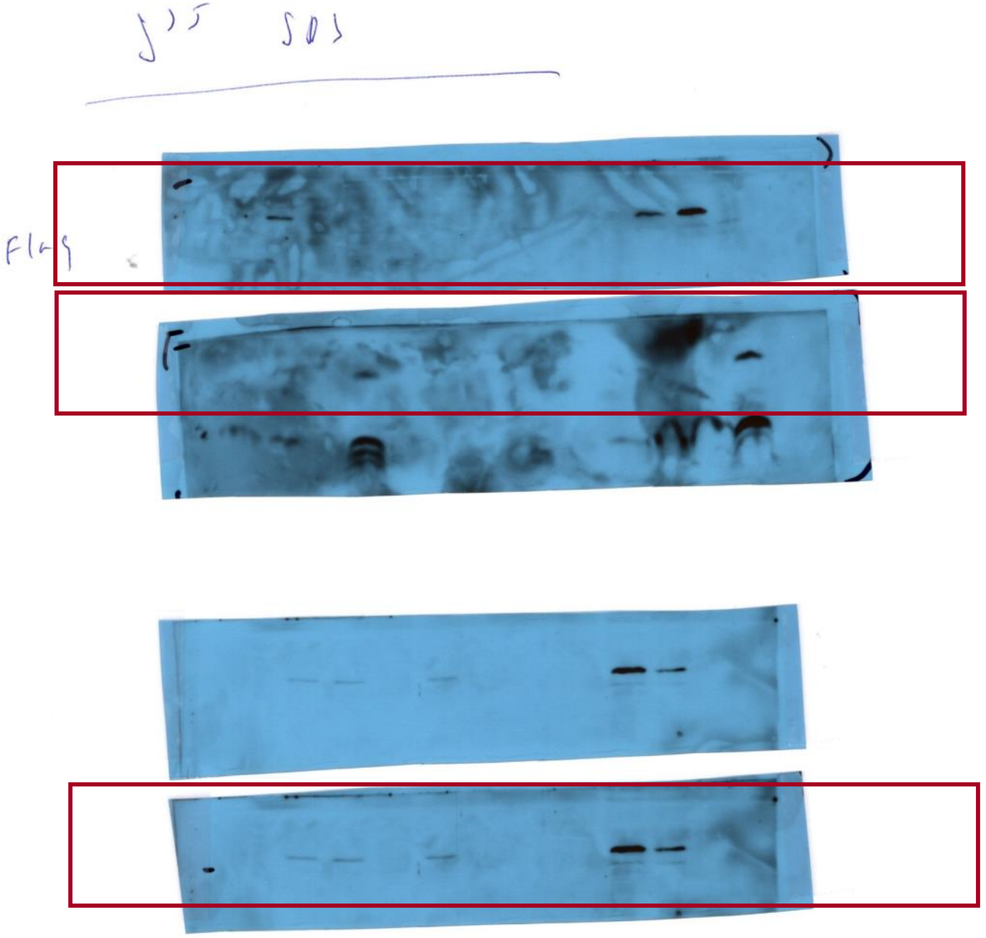

COX11-FLAG

PET191-FLAG

COX11-FLAG

Supplementary Fig. 7B

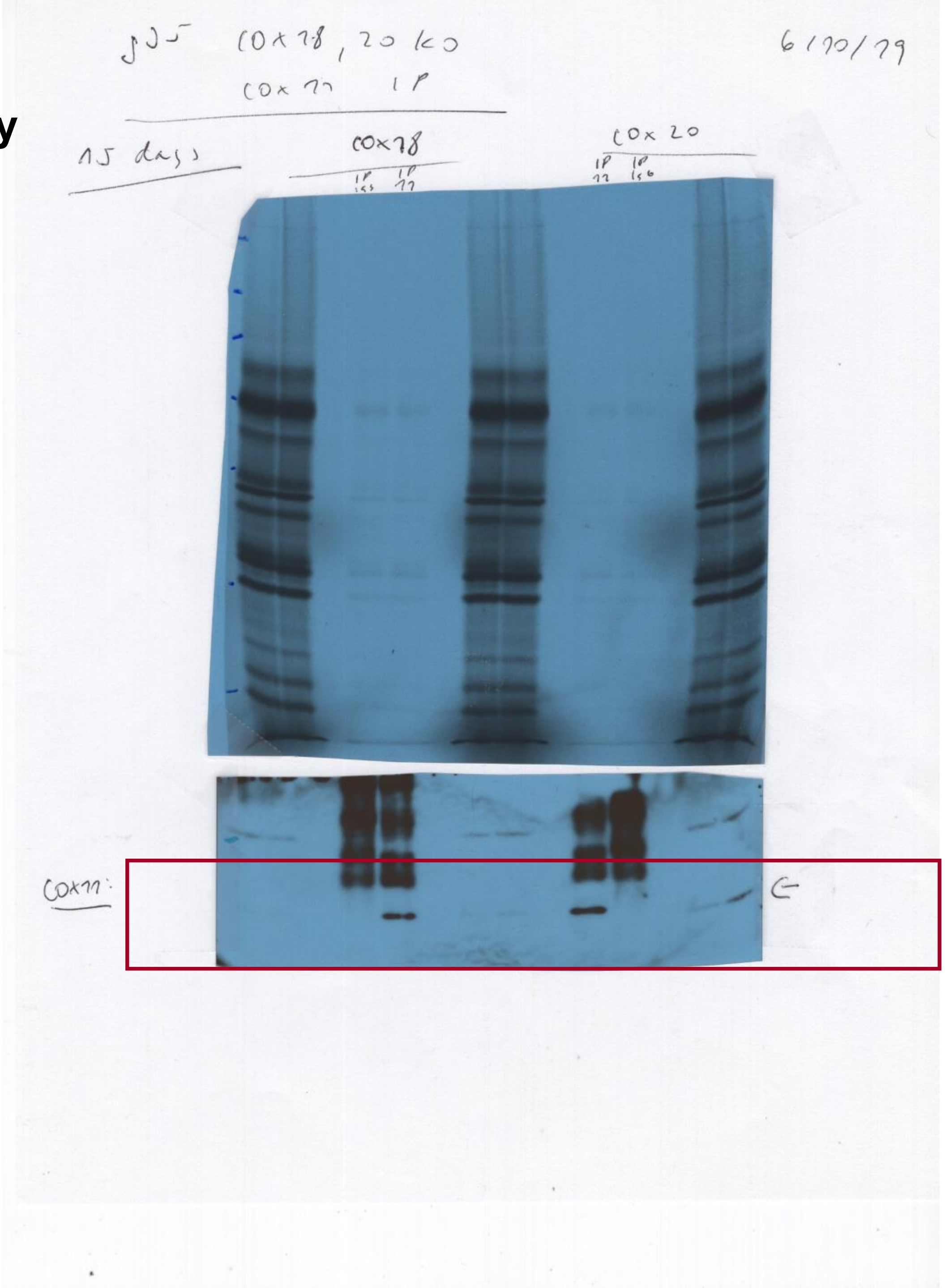

COX11

Supplementary Fig. 7B

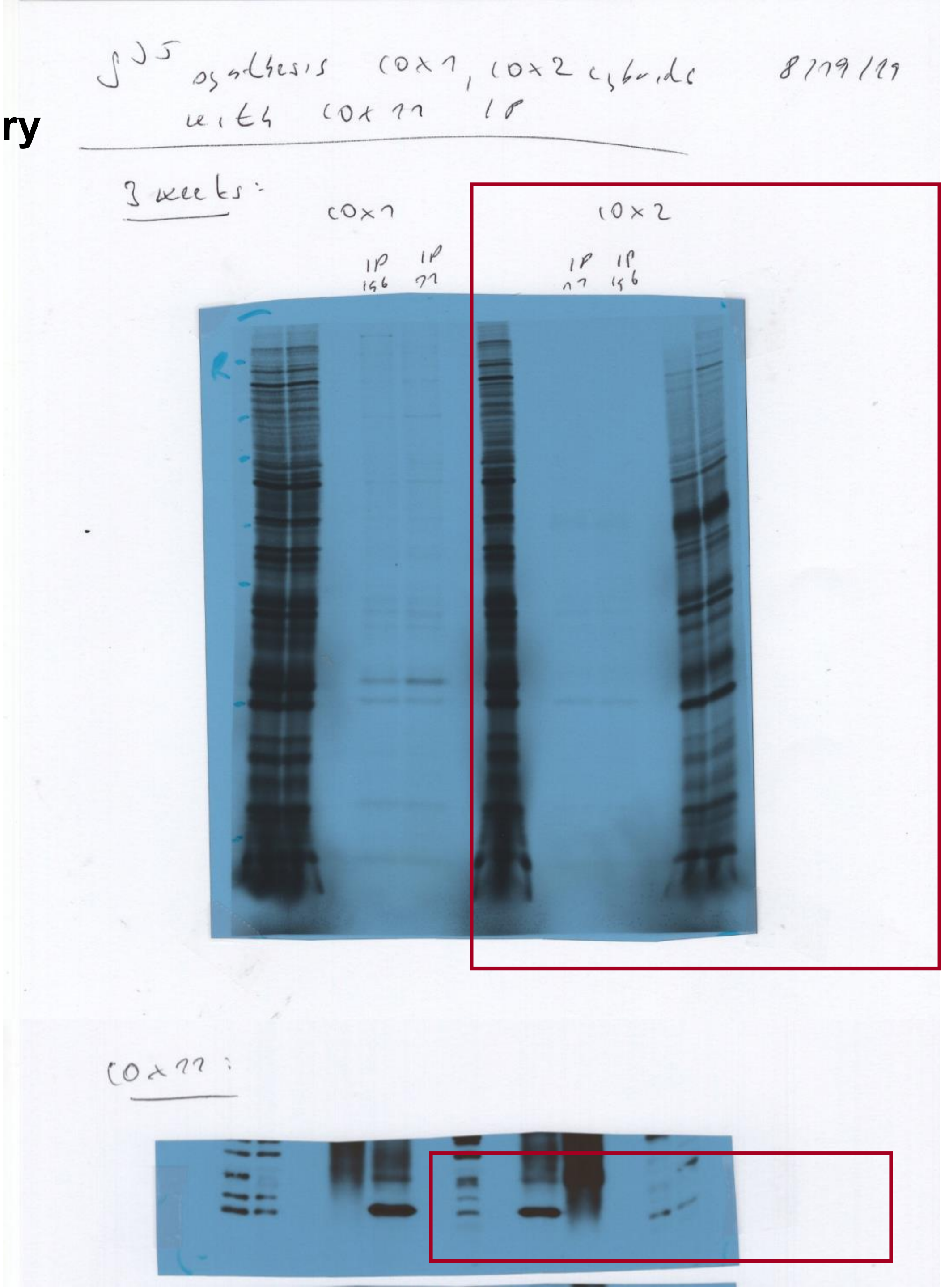

COX11

Supplementary Fig. 7C

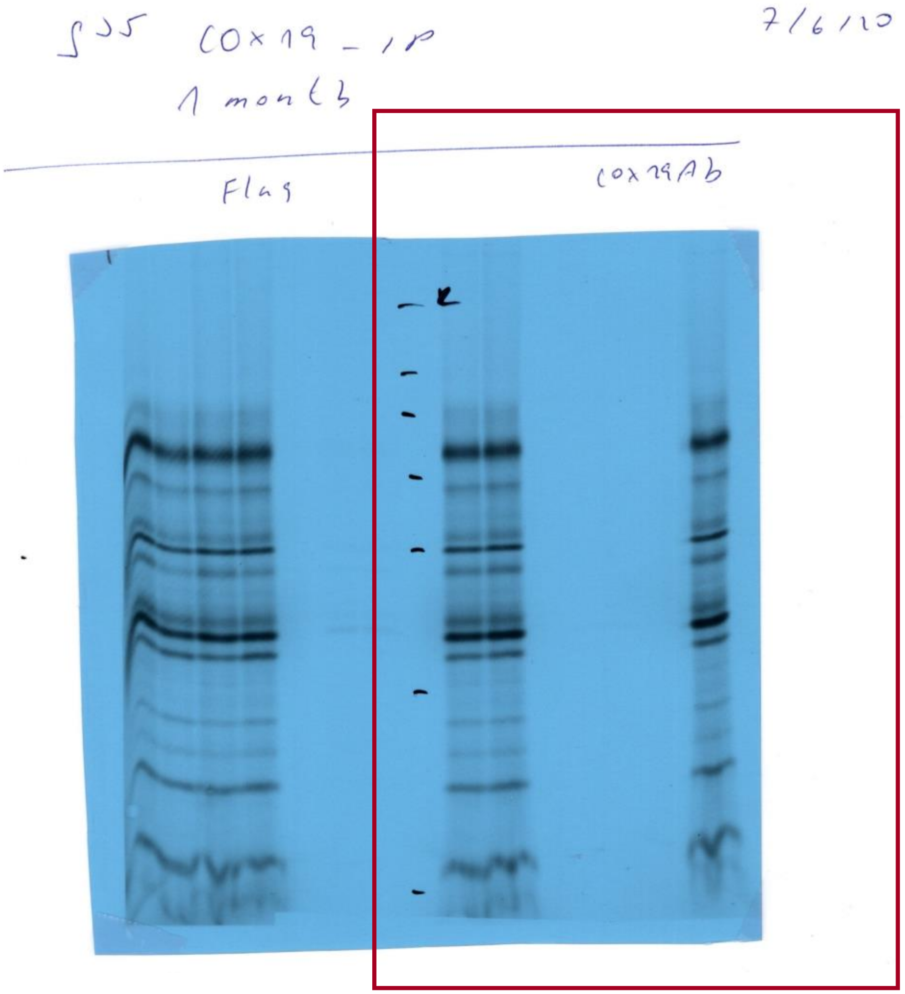

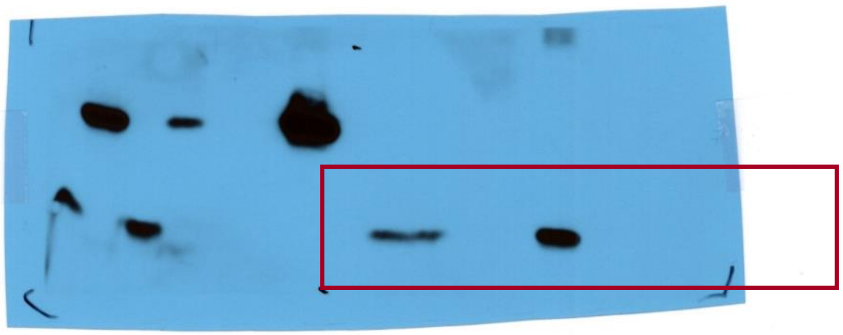

COX19

Supplementary Fig. 7D

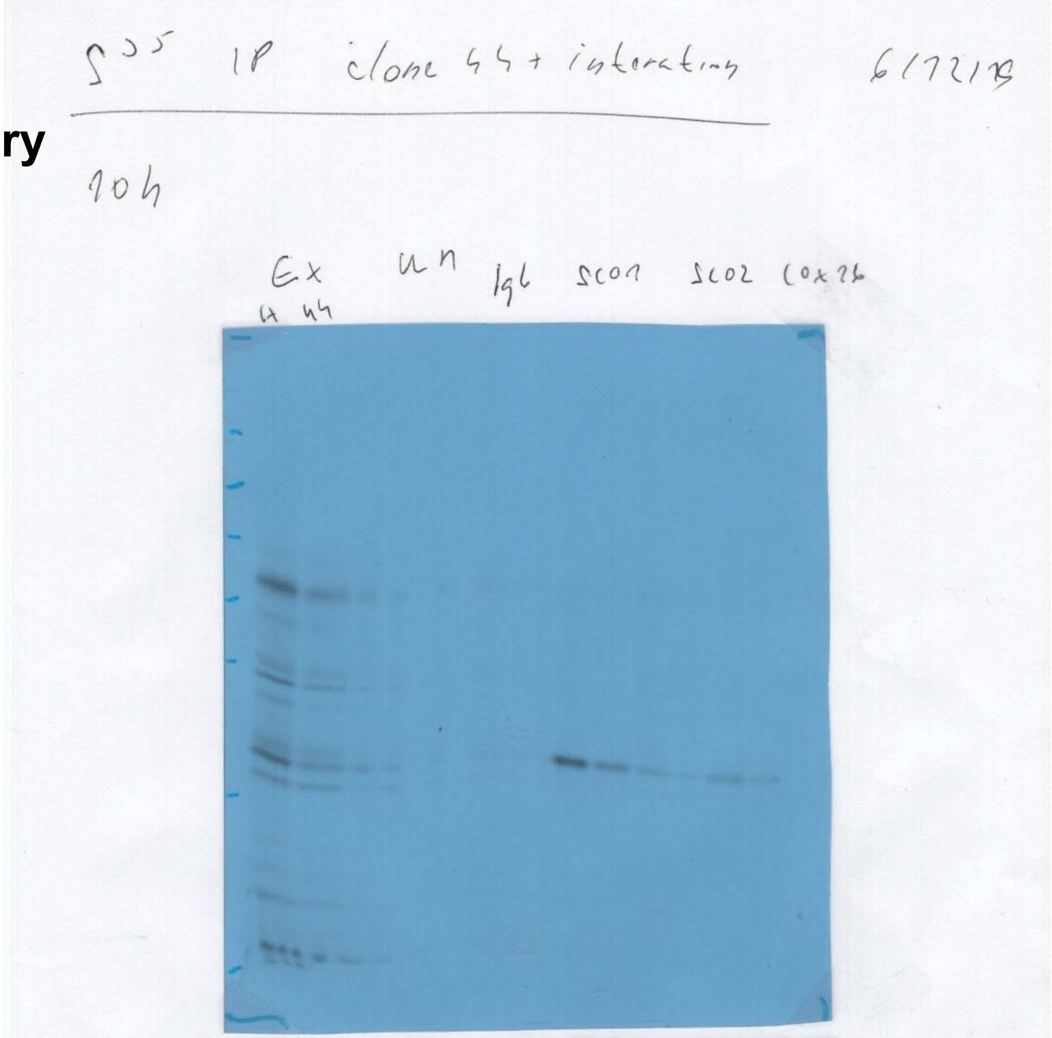

Supplementary Fig. 7D

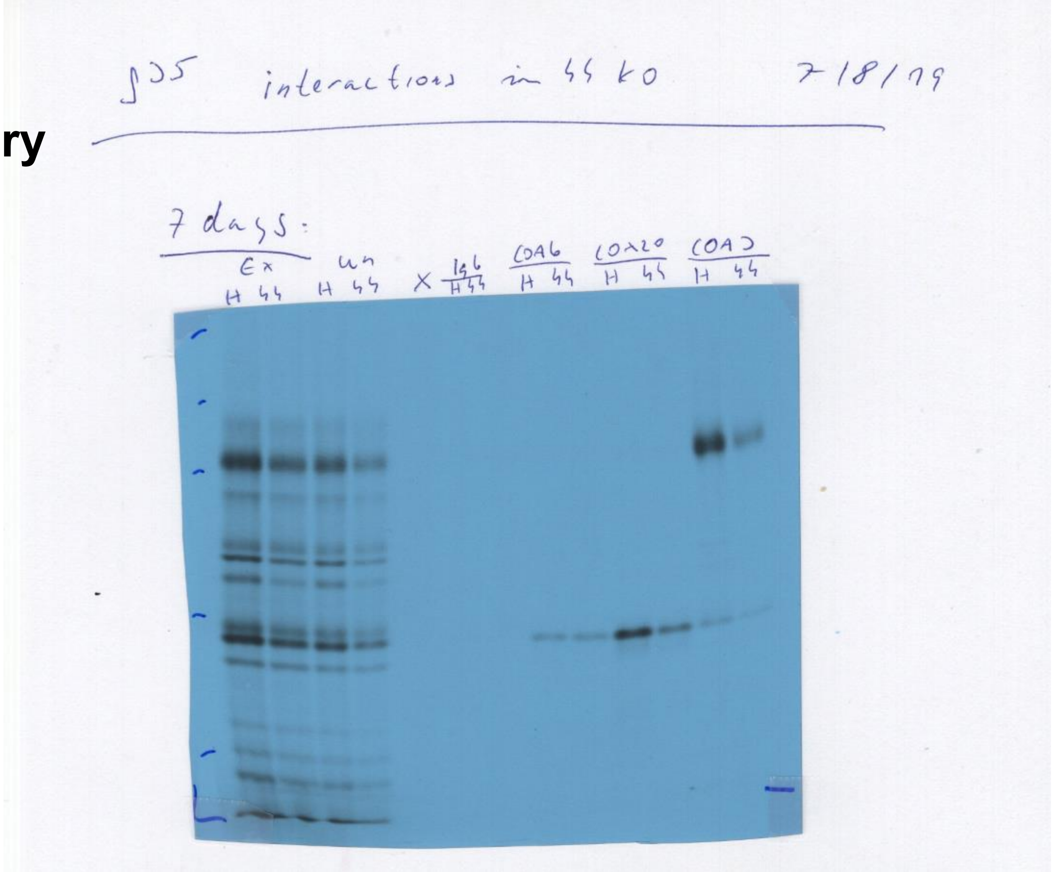

Supplementary Fig. 7D

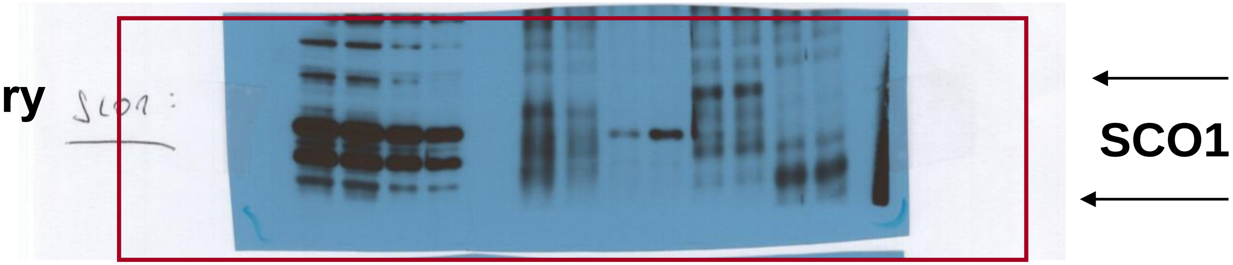

Supplementary Fig. 7E

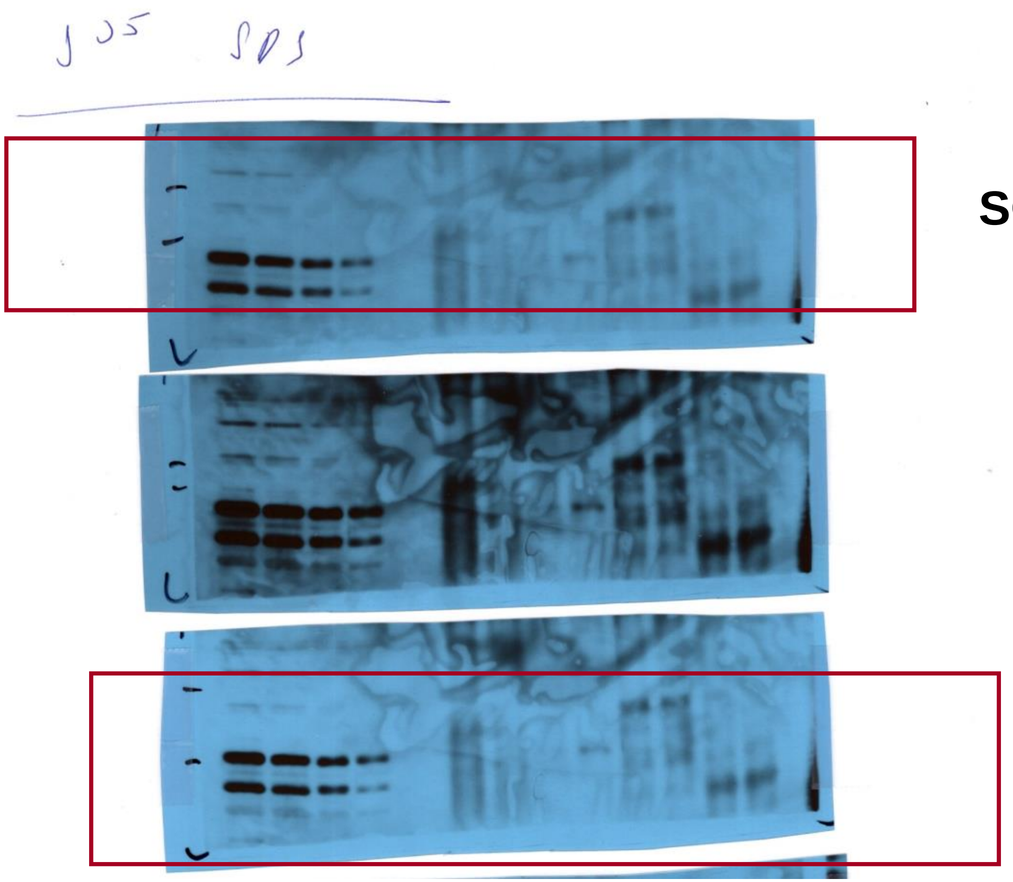

Supplementary Fig. 7D

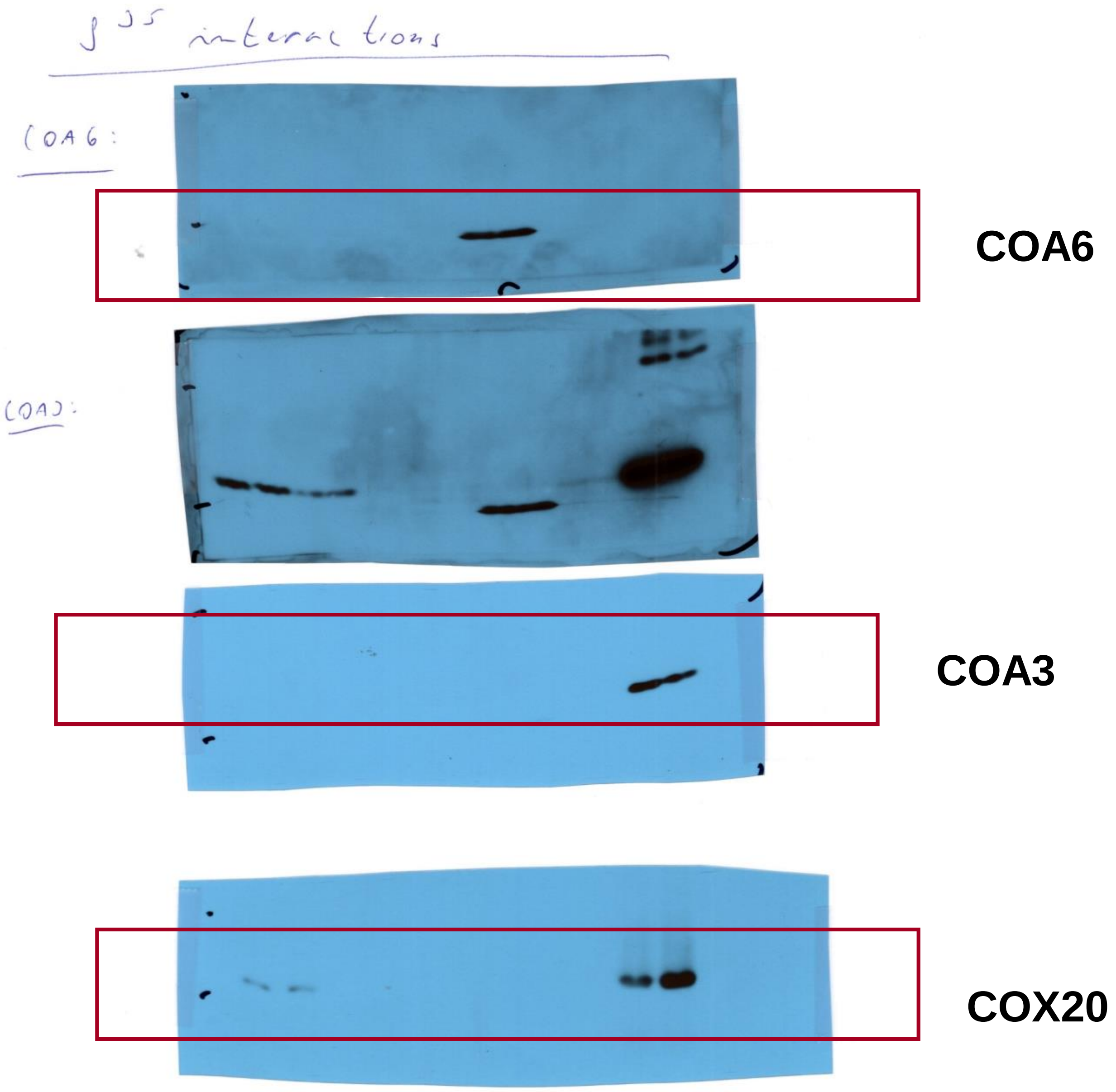

Supplementary Fig. 7E

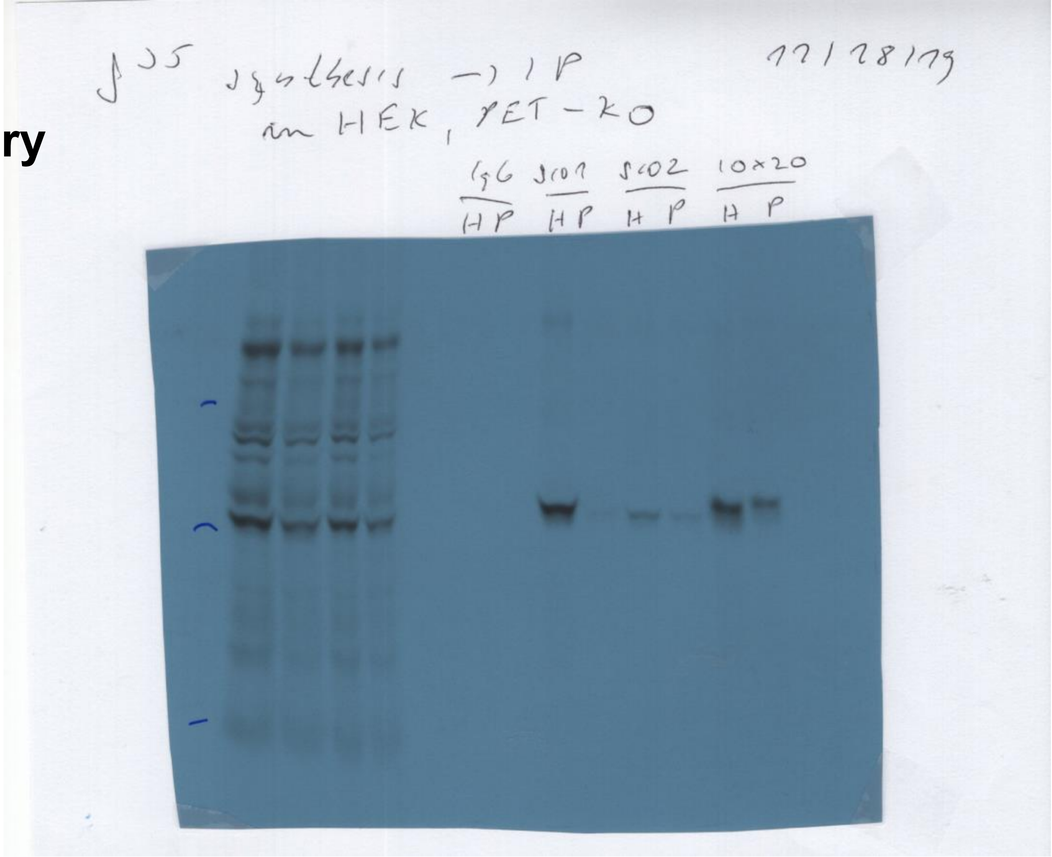

Supplementary Fig. 7E

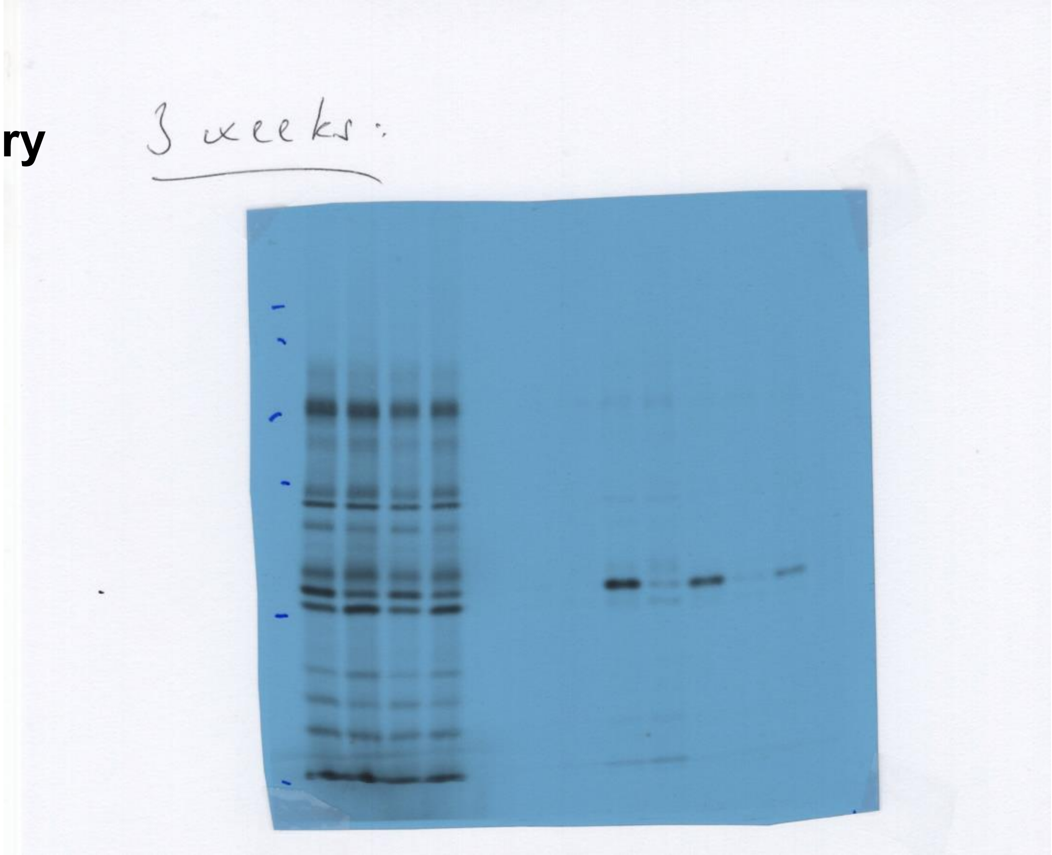

Supplementary Fig. 7E

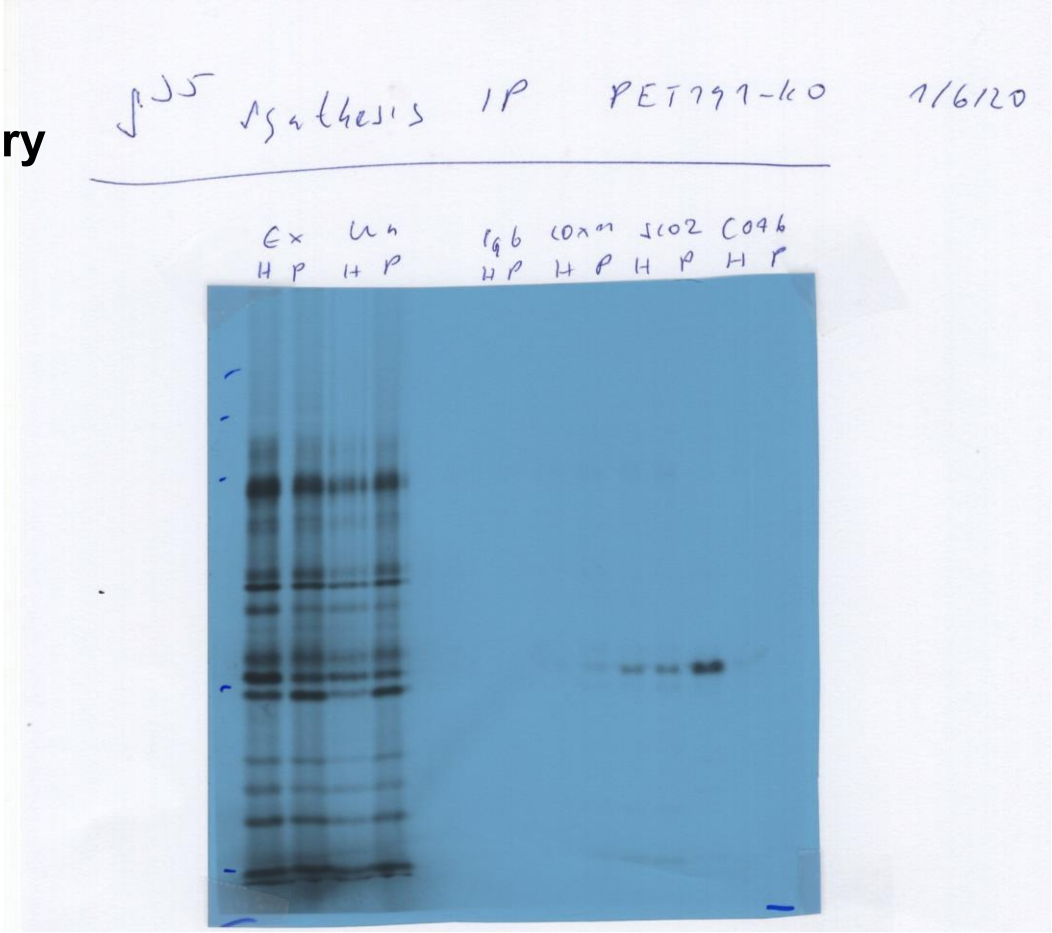

Supplementary Fig. 7E

SCO2

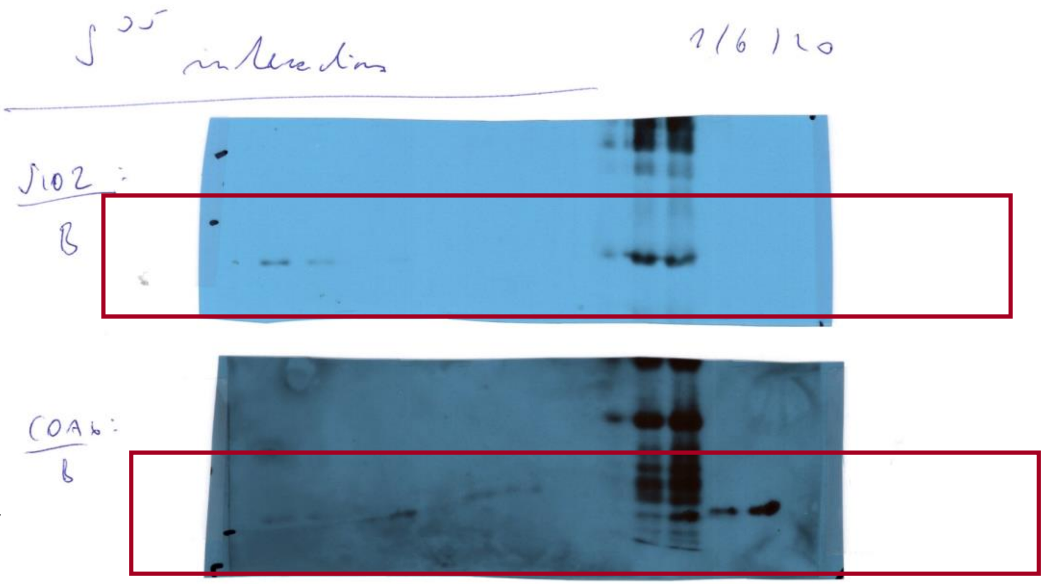

Supplementary Fig. 7E

COA6

Supplementary Fig. 7E

COX11

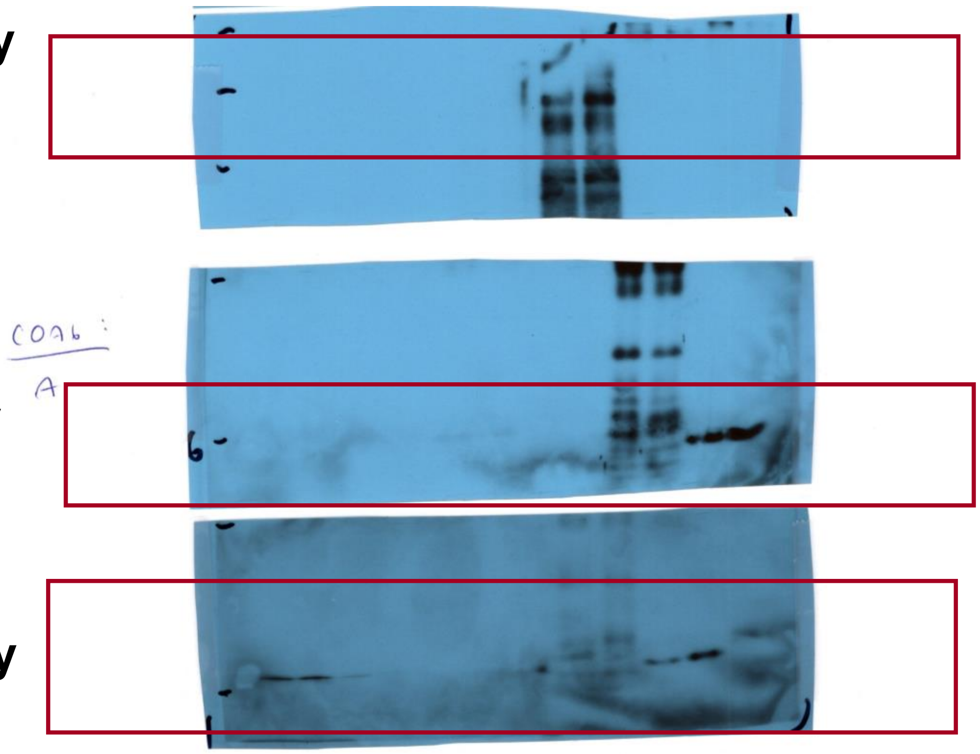

Supplementary Fig. 7F

COA6

Supplementary Fig. 7E

COA6

Supplementary Fig. 7F

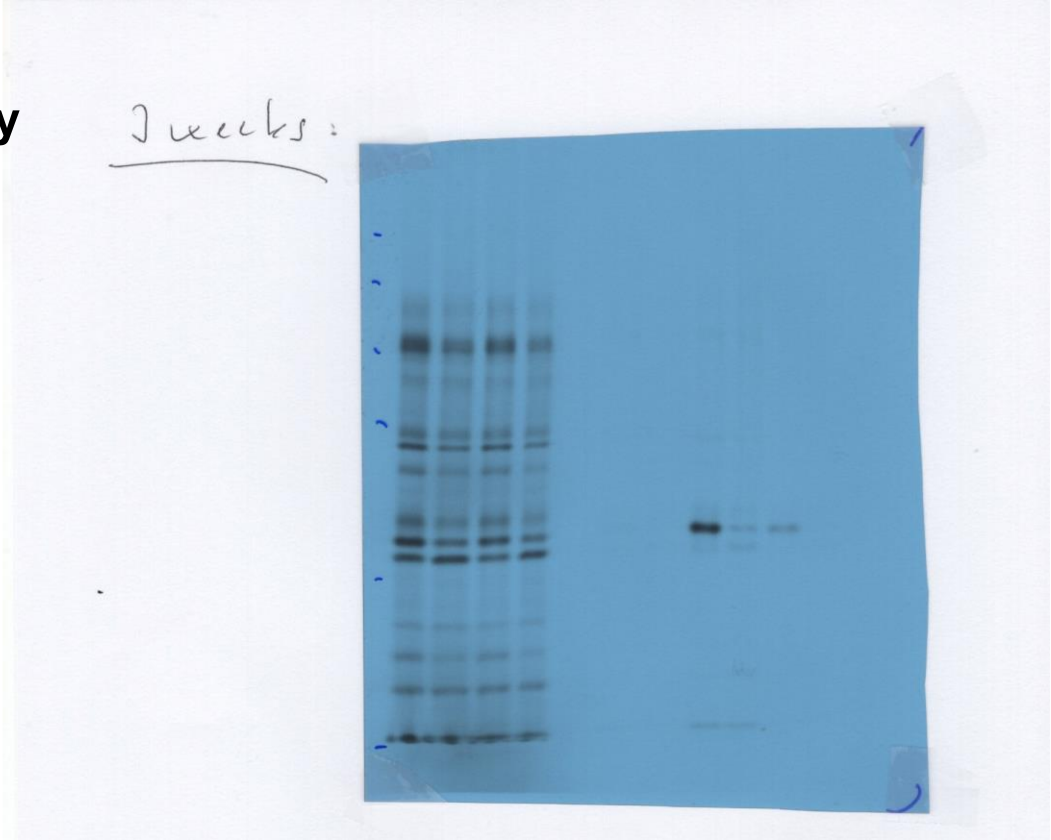

Supplementary Fig. 7F

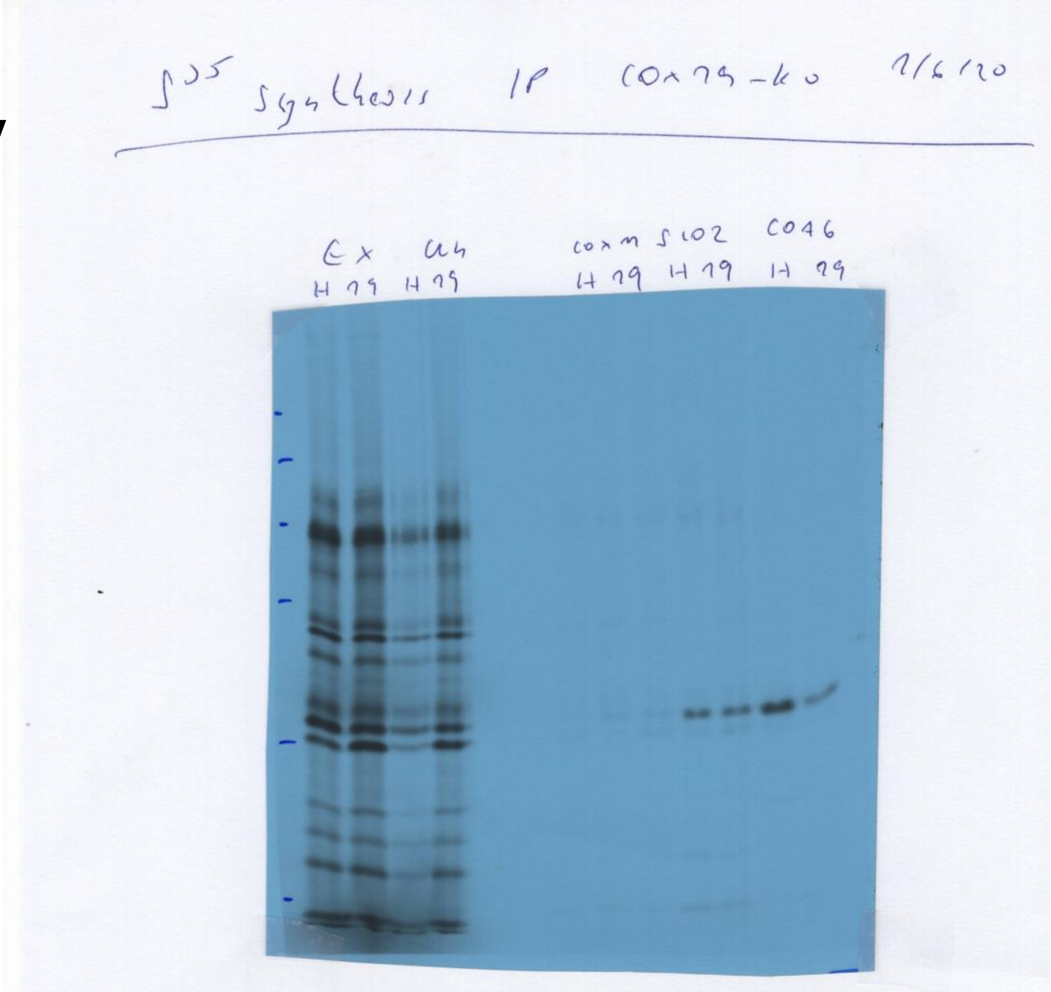

Supplementary Fig. 7E

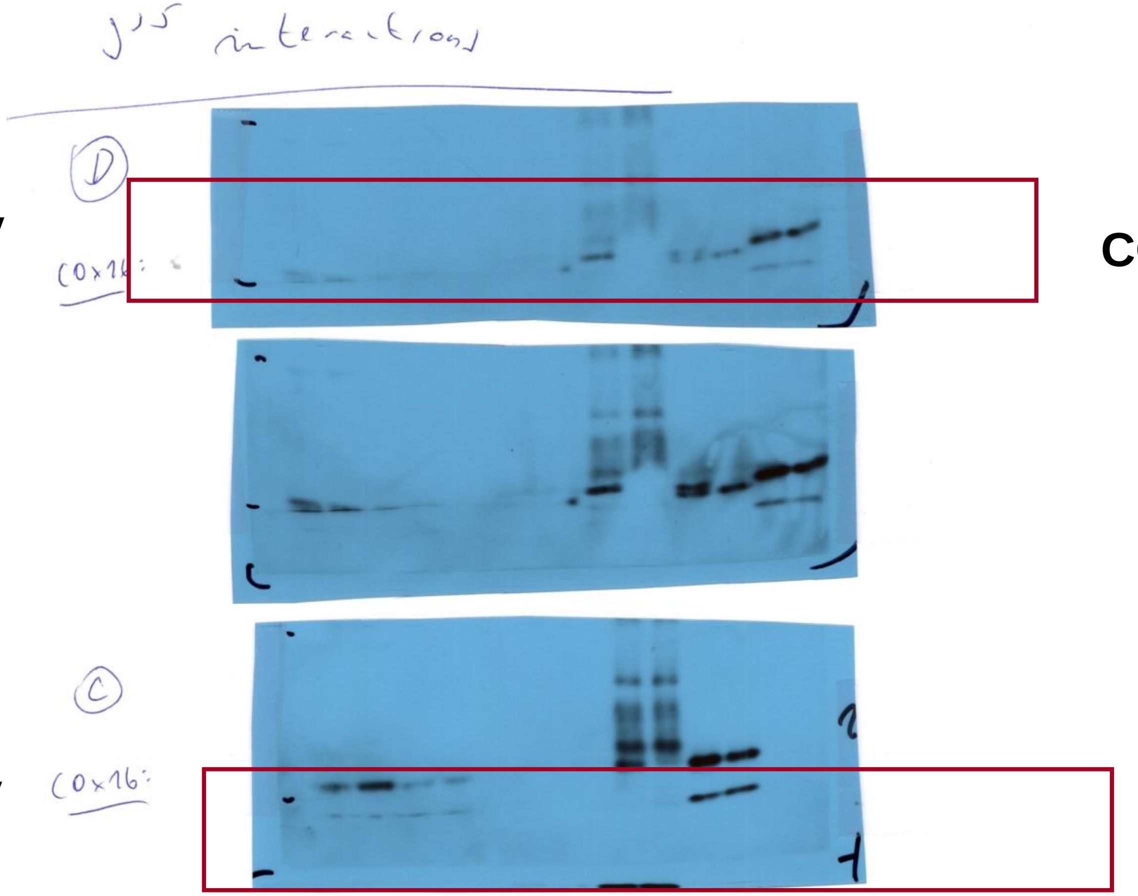

COX16

Supplementary Fig. 7F

COX16

Supplementary Fig. 7F

SCO1

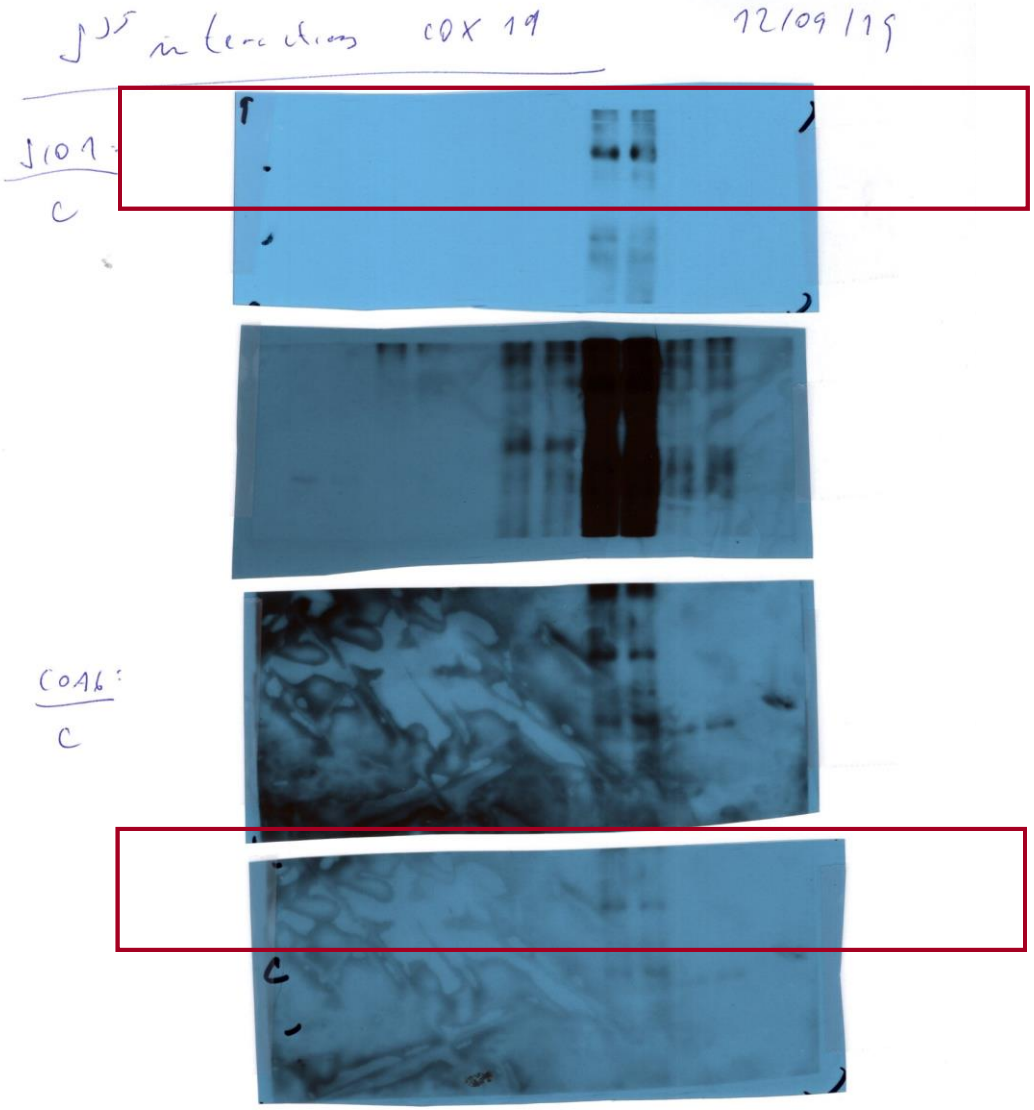

Supplementary Fig. 7E

SCO1

Supplementary Fig. 8A

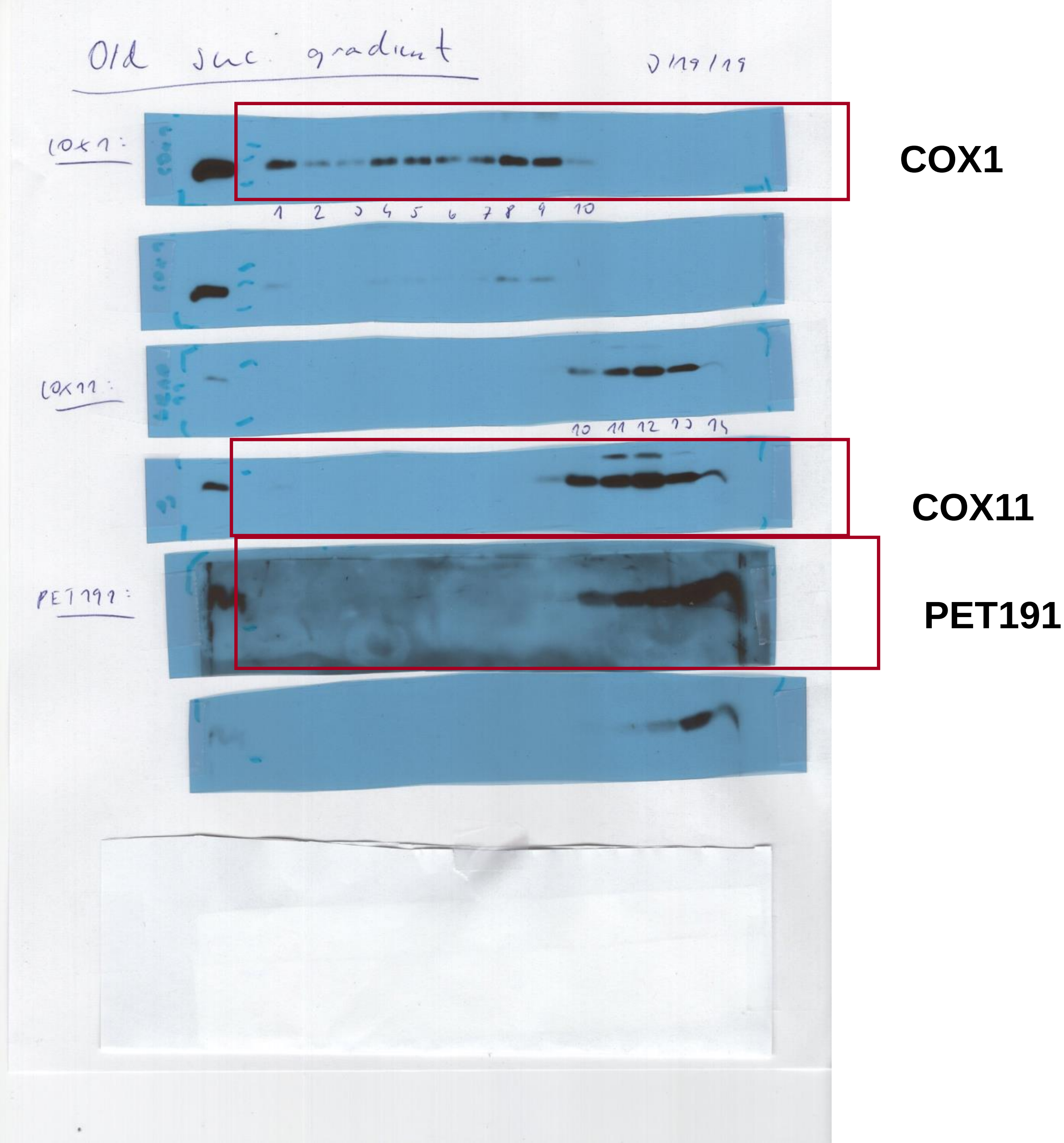

Supplementary Fig. 8A

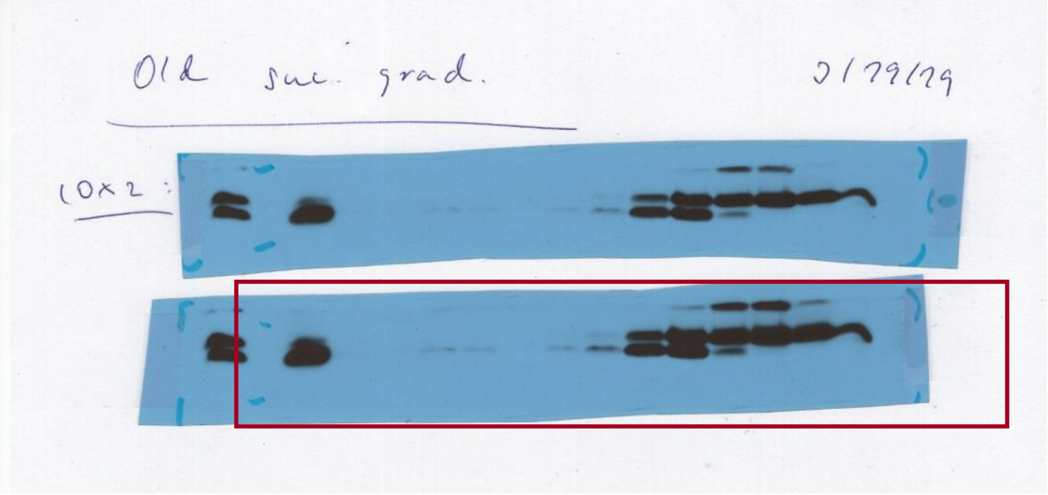

COX11
COX2

Supplementary Fig. 8B

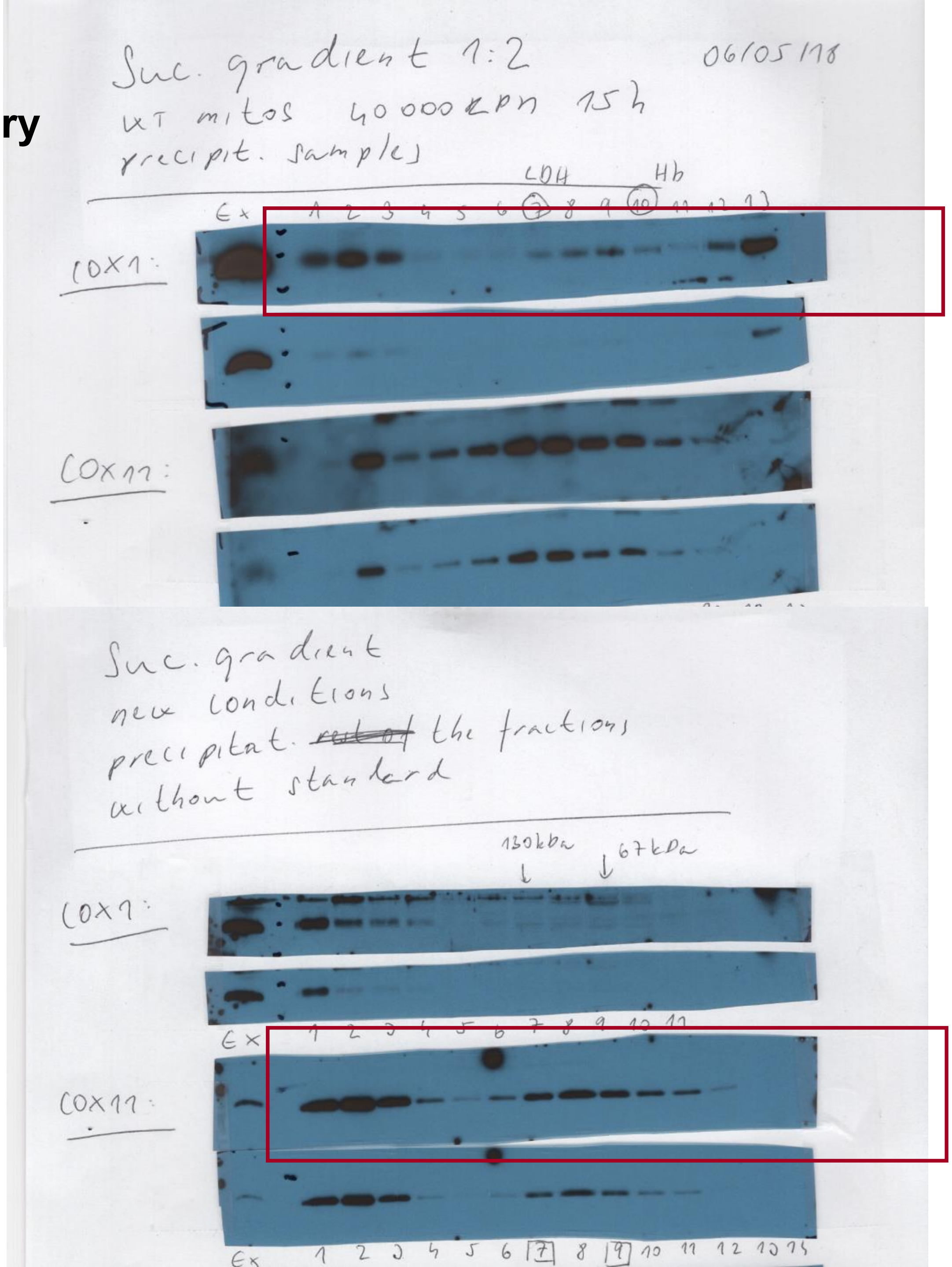

Supplementary Fig. 8B

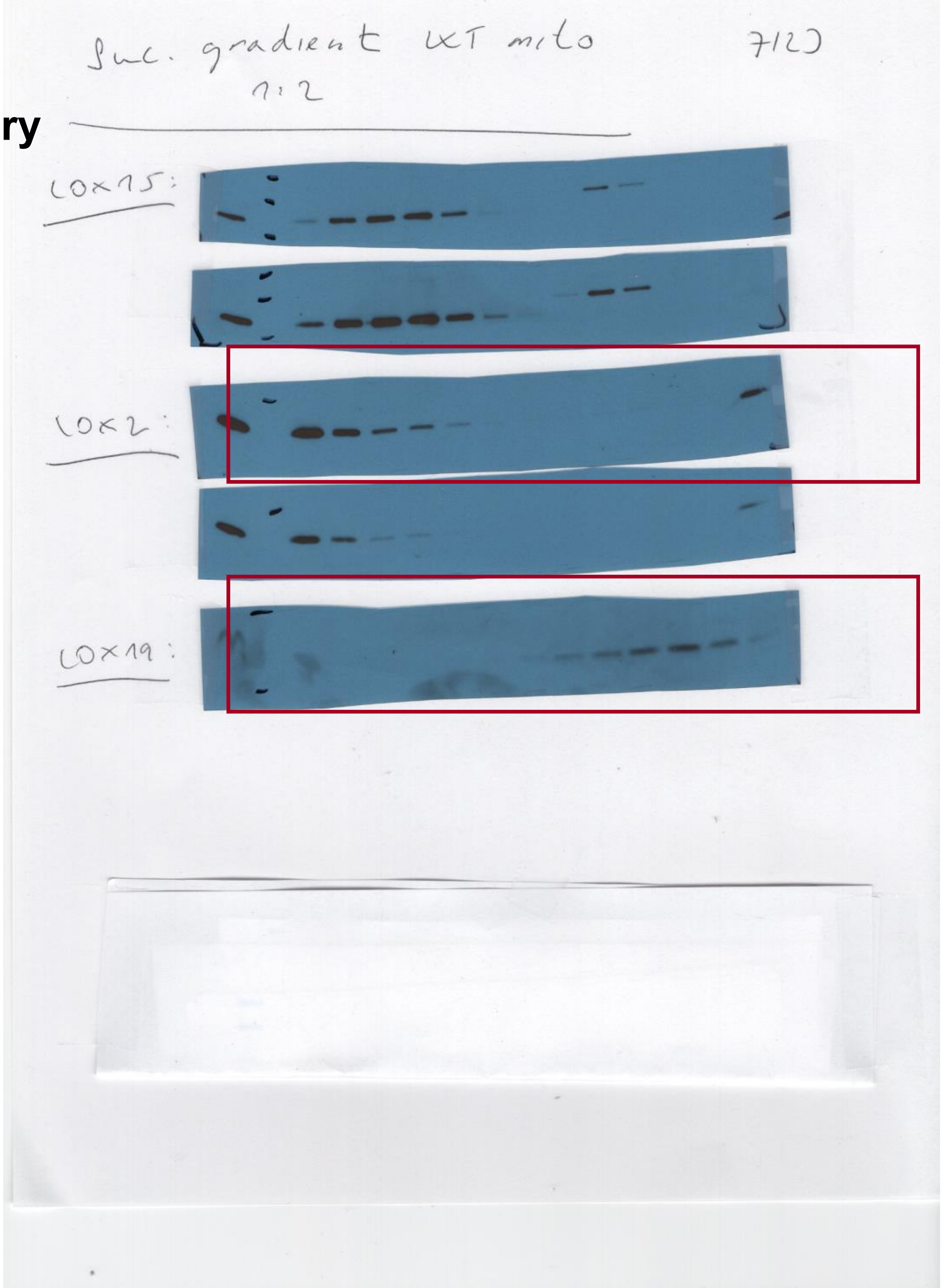

COX2

COX19

Supplementary Fig. 8B

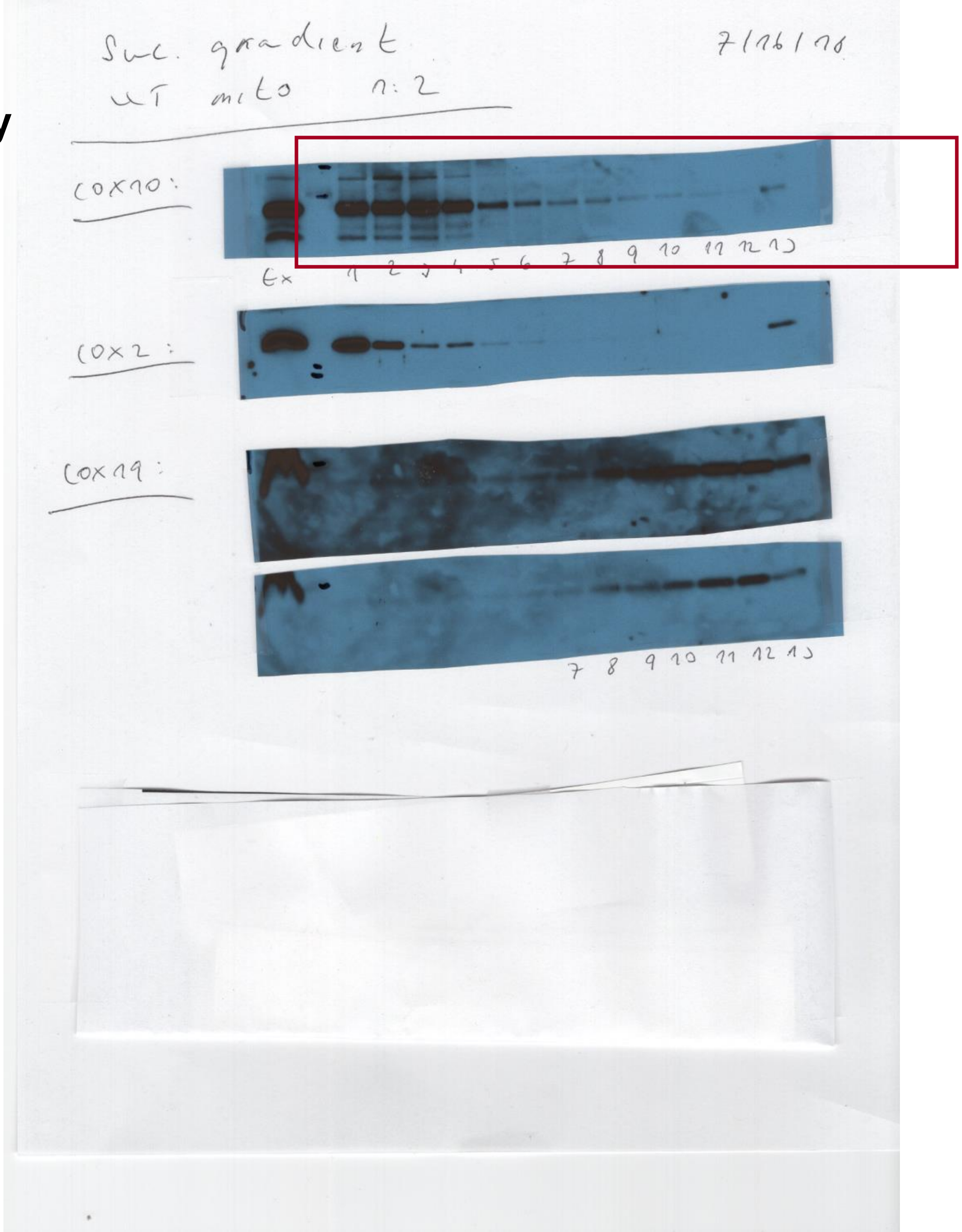

COX10

Supplementary Fig. 8B

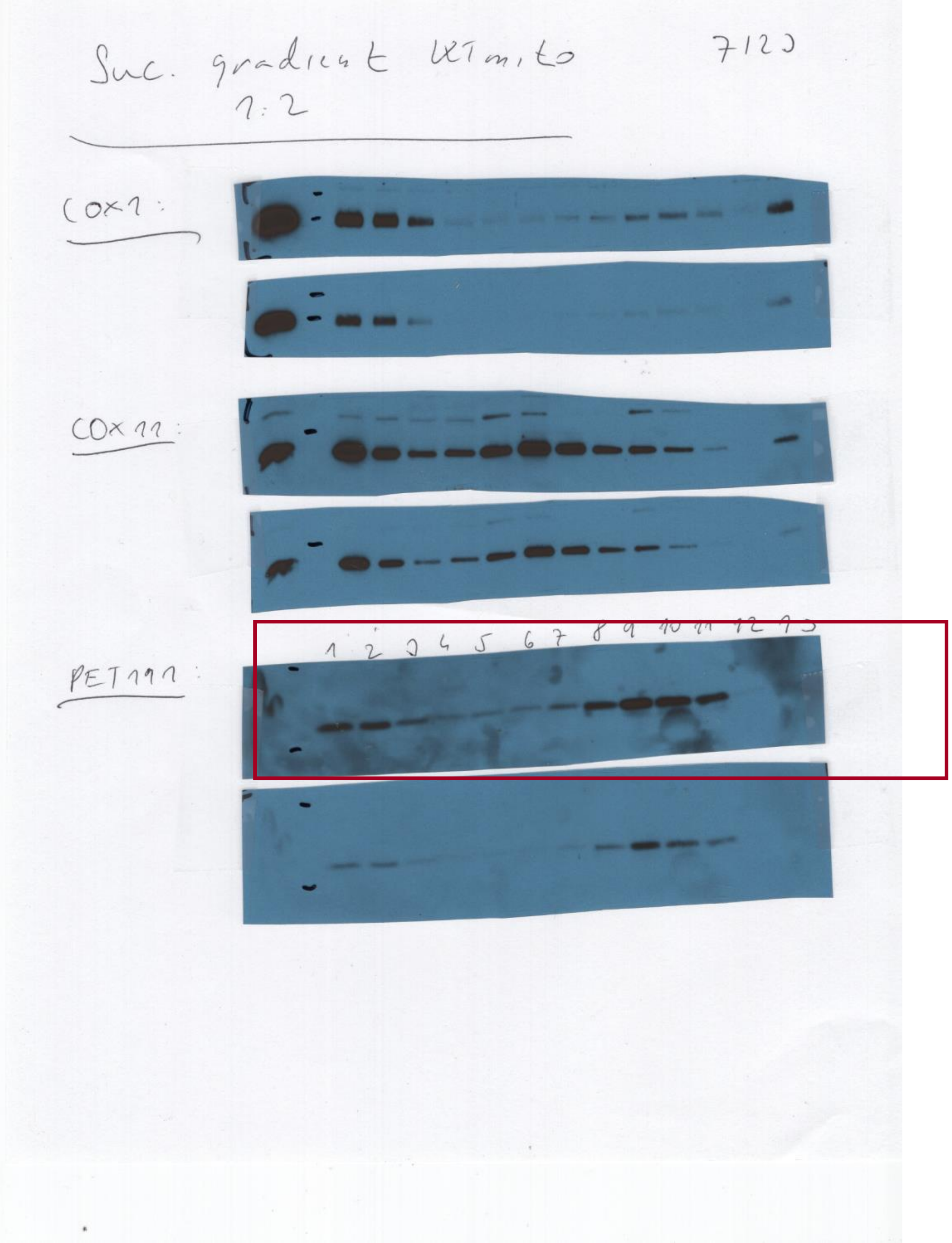

PET191

Supplementary Fig. 8B

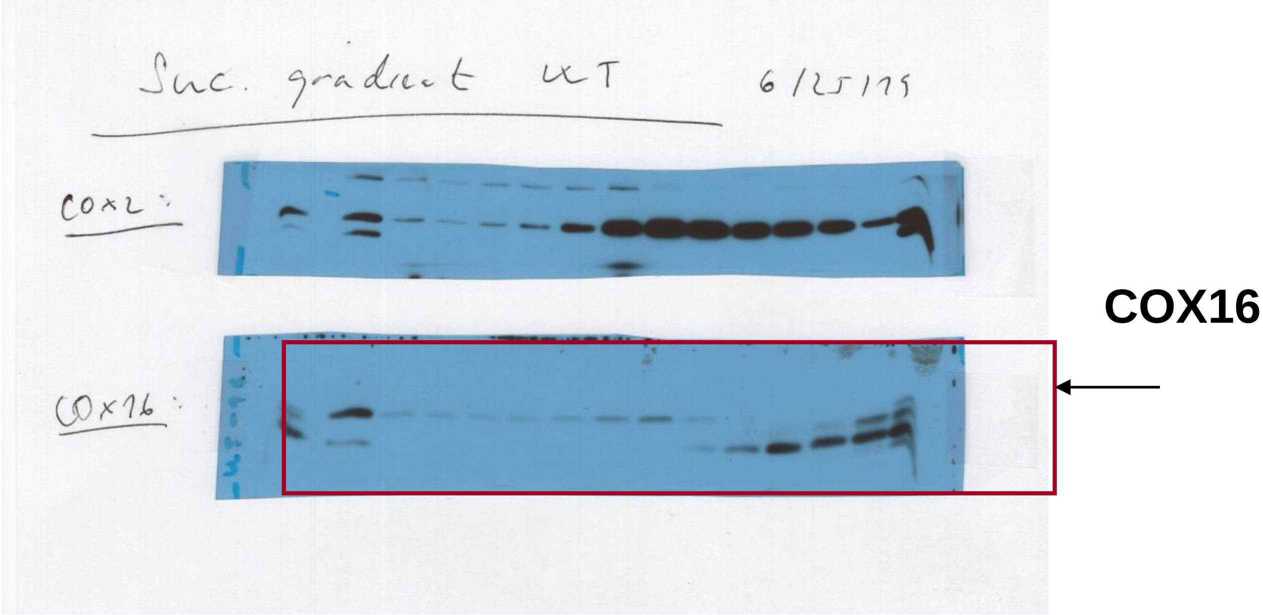

COX16

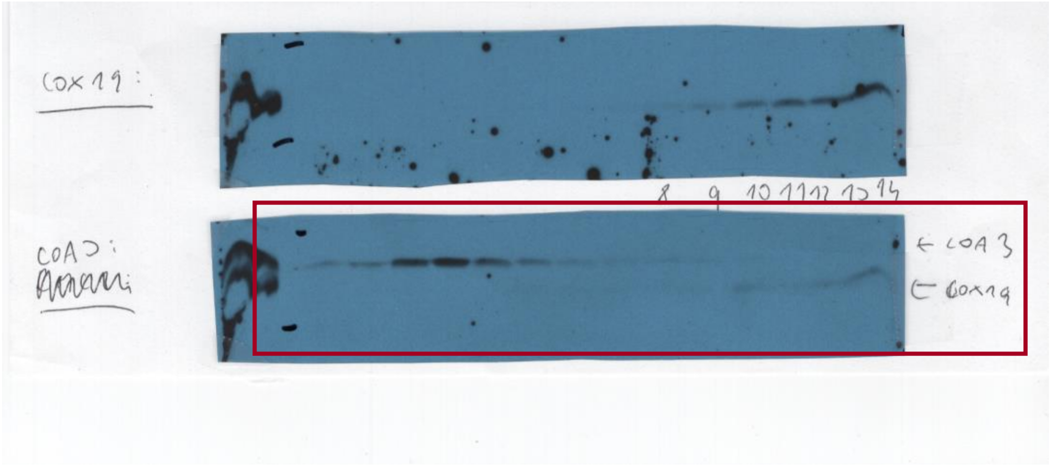

COA3

Supplementary Fig. 8B

COX11-KO

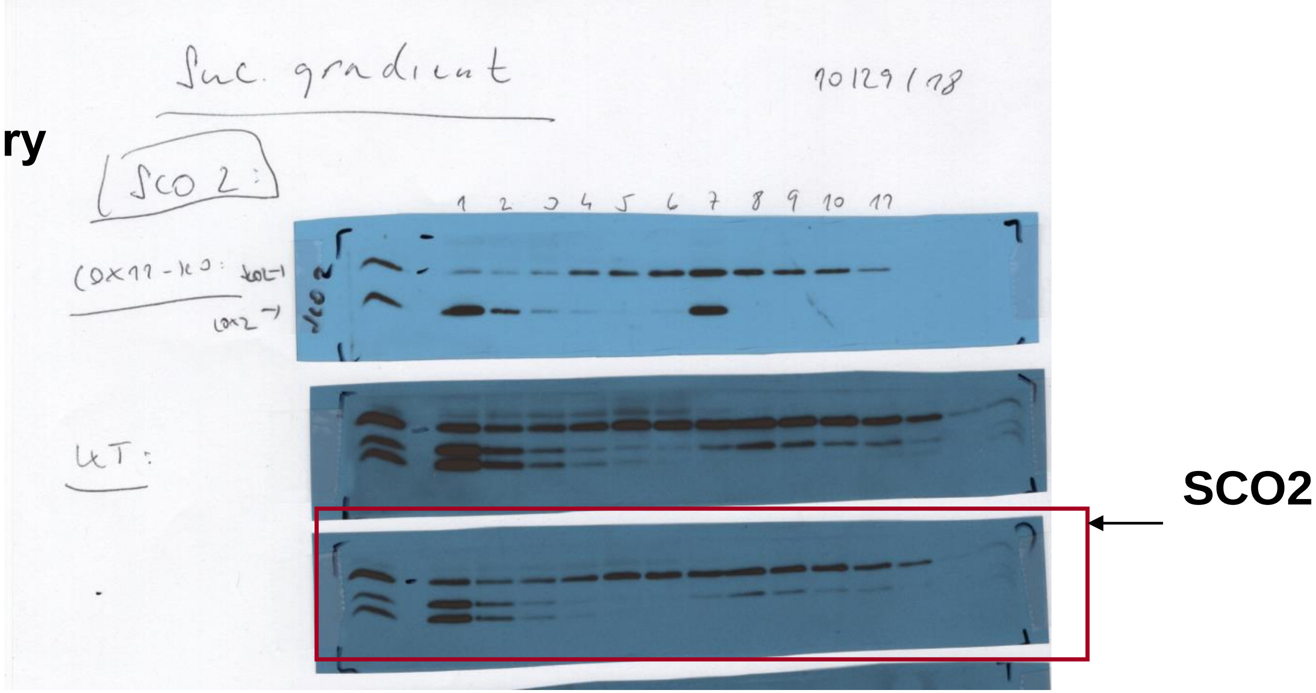

SCO2

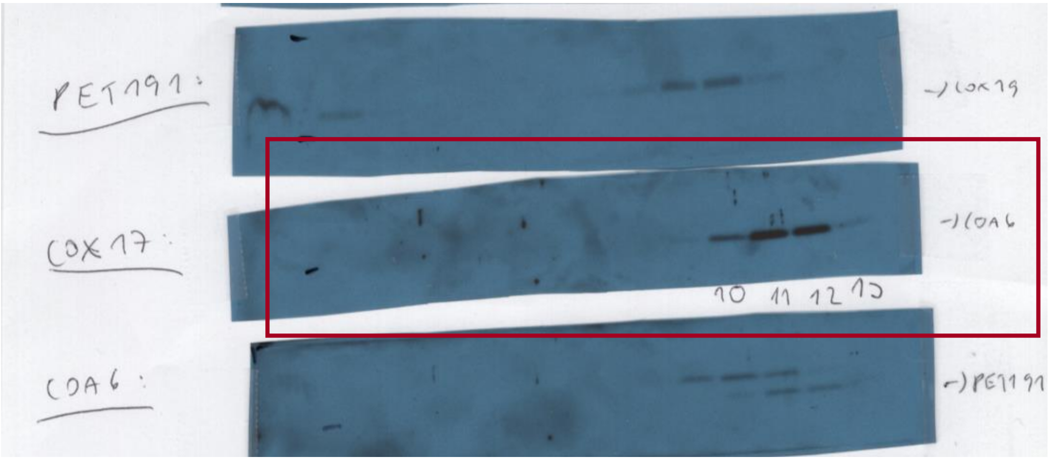

COX17

Supplementary Fig. 8B

COX11-KO

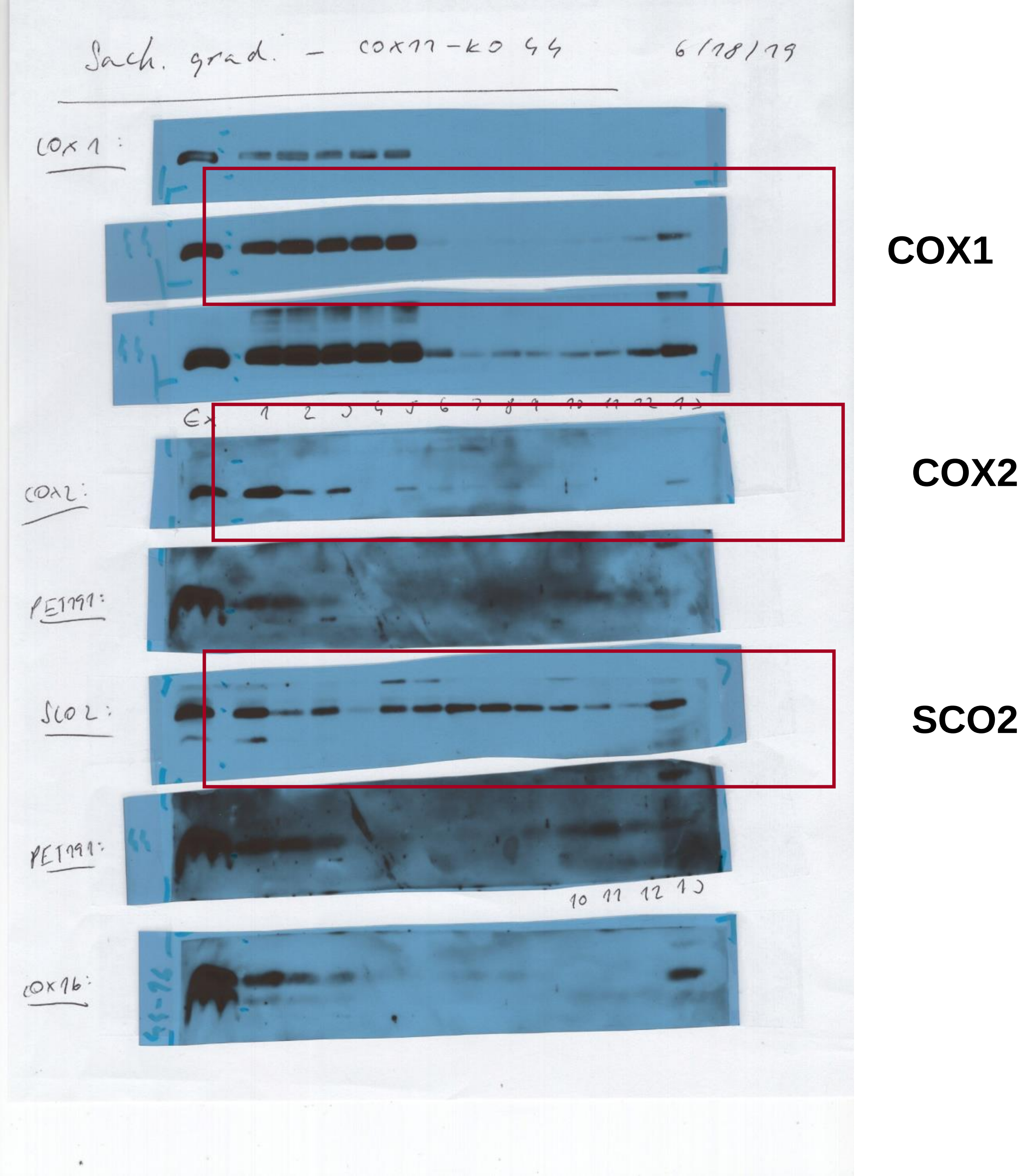

Supplementary Fig 8B

COX11-KO

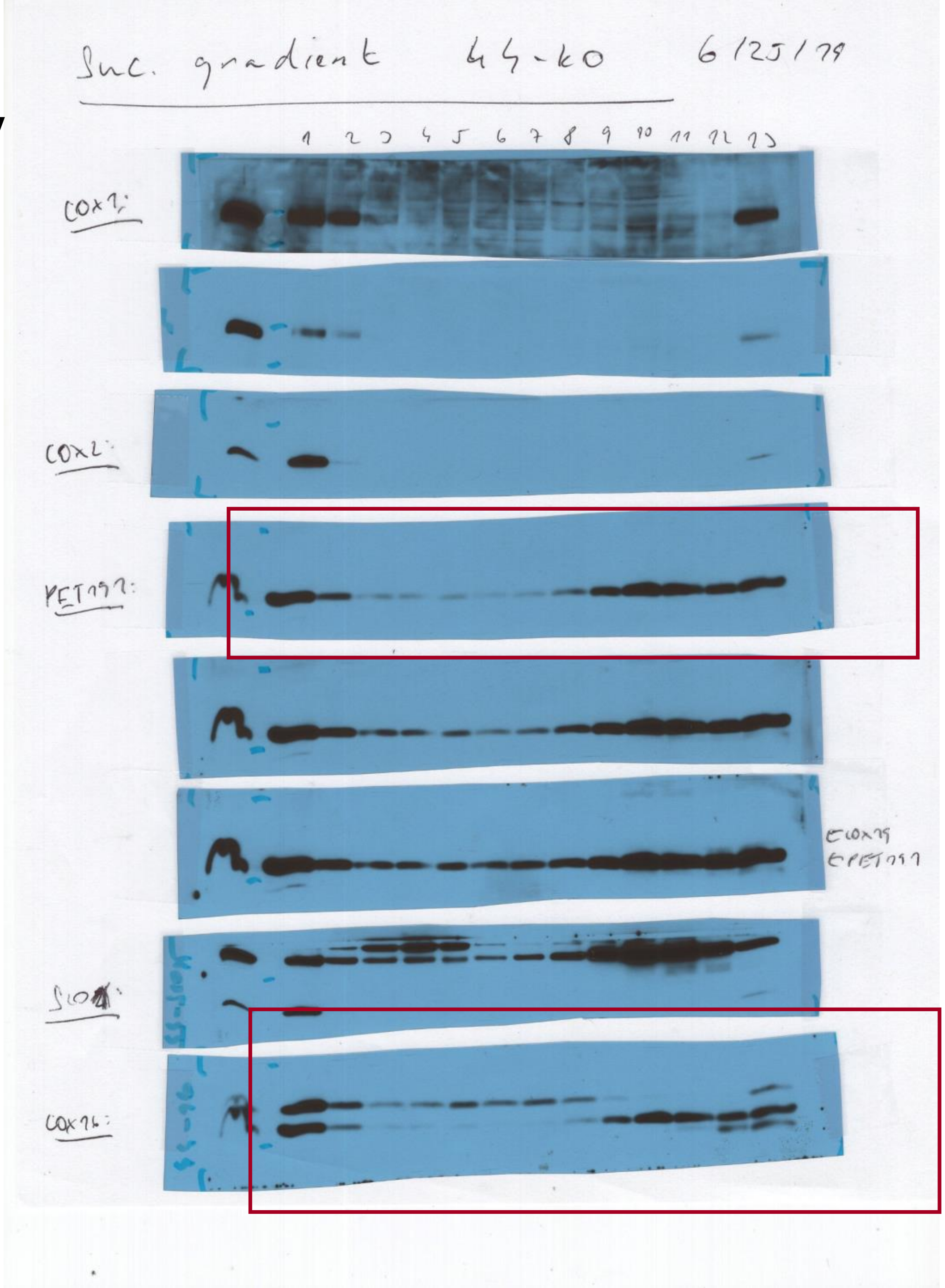

PET191

COX16
PET191
COX19

Supplementary Fig. 8C

COX19-KO

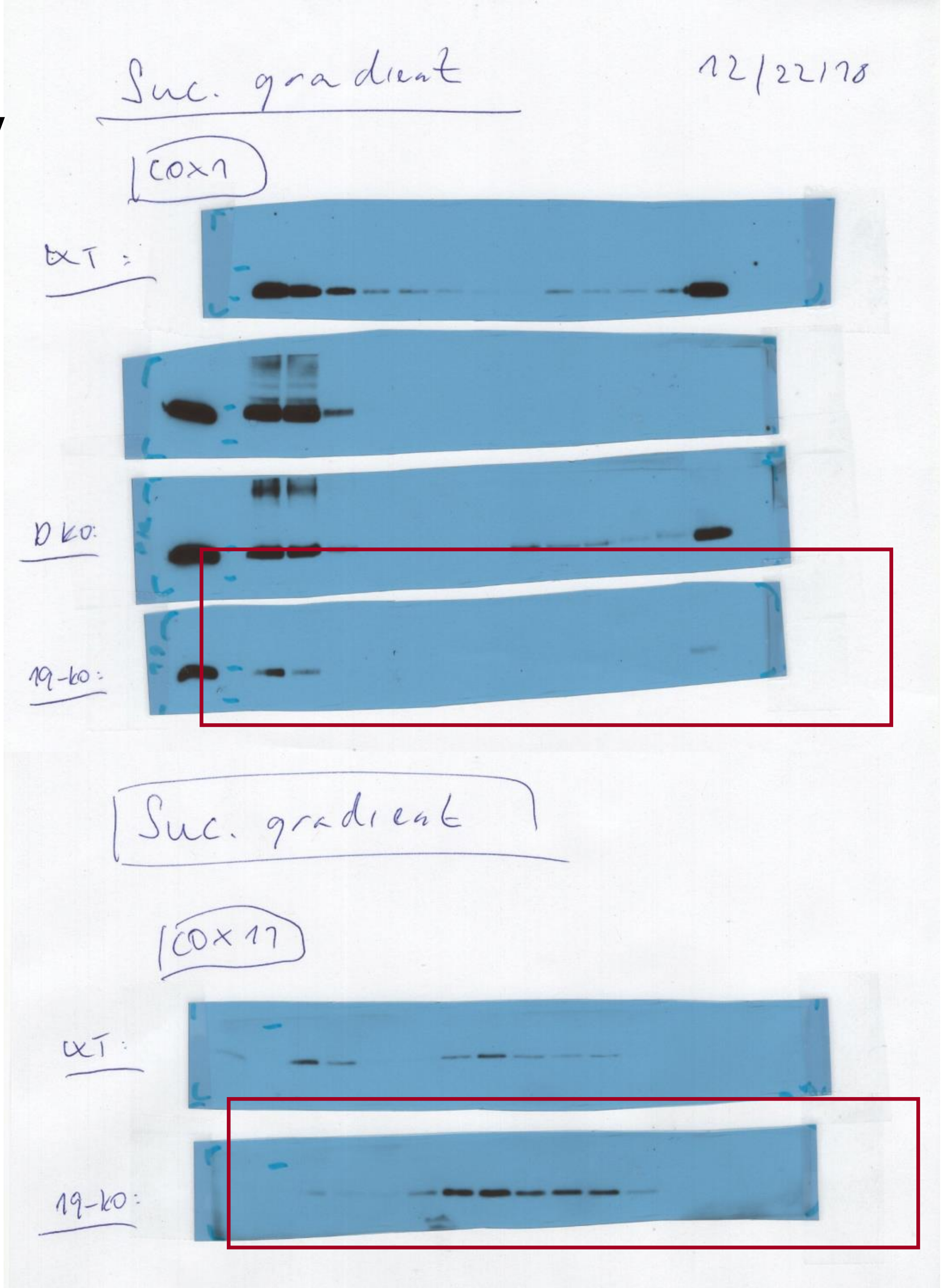

COX1

COX11

Supplementary Fig. 8C

COX19-KO

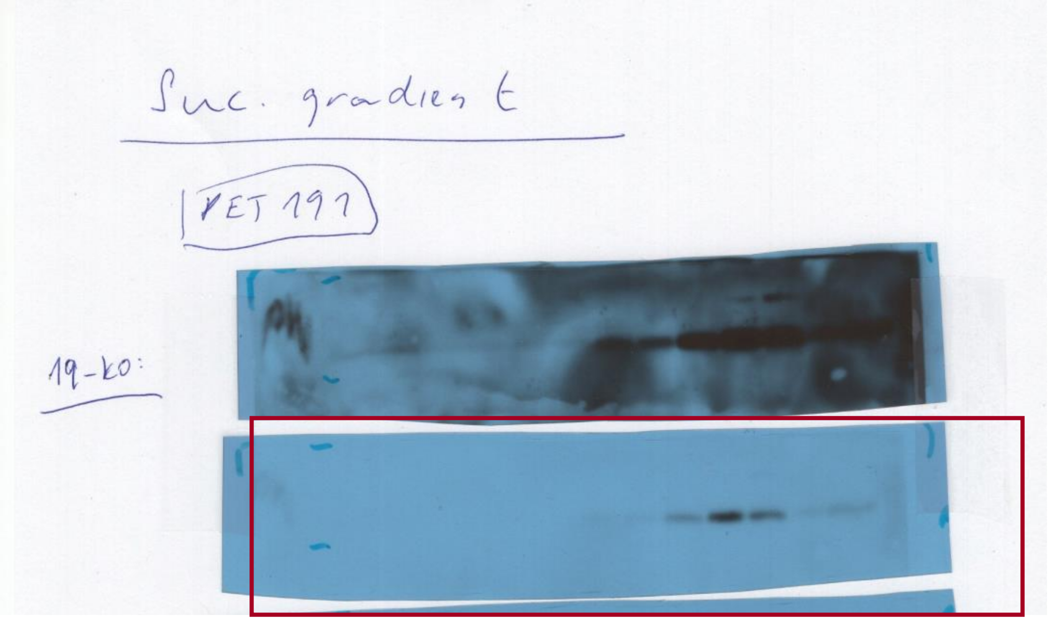

PET191

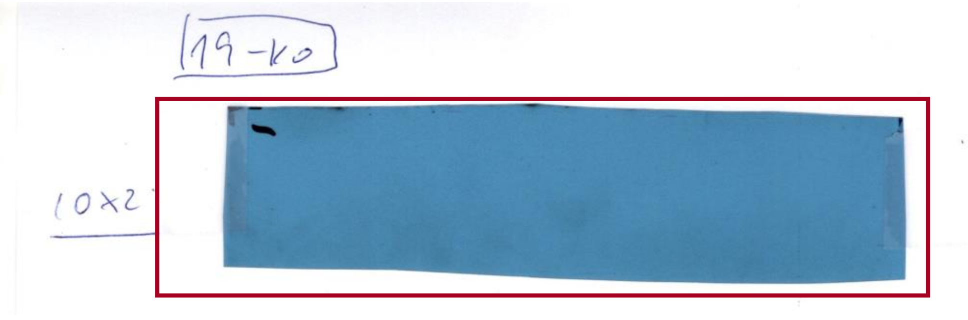

COX2

Supplementary Fig. 8C

PET191-KO

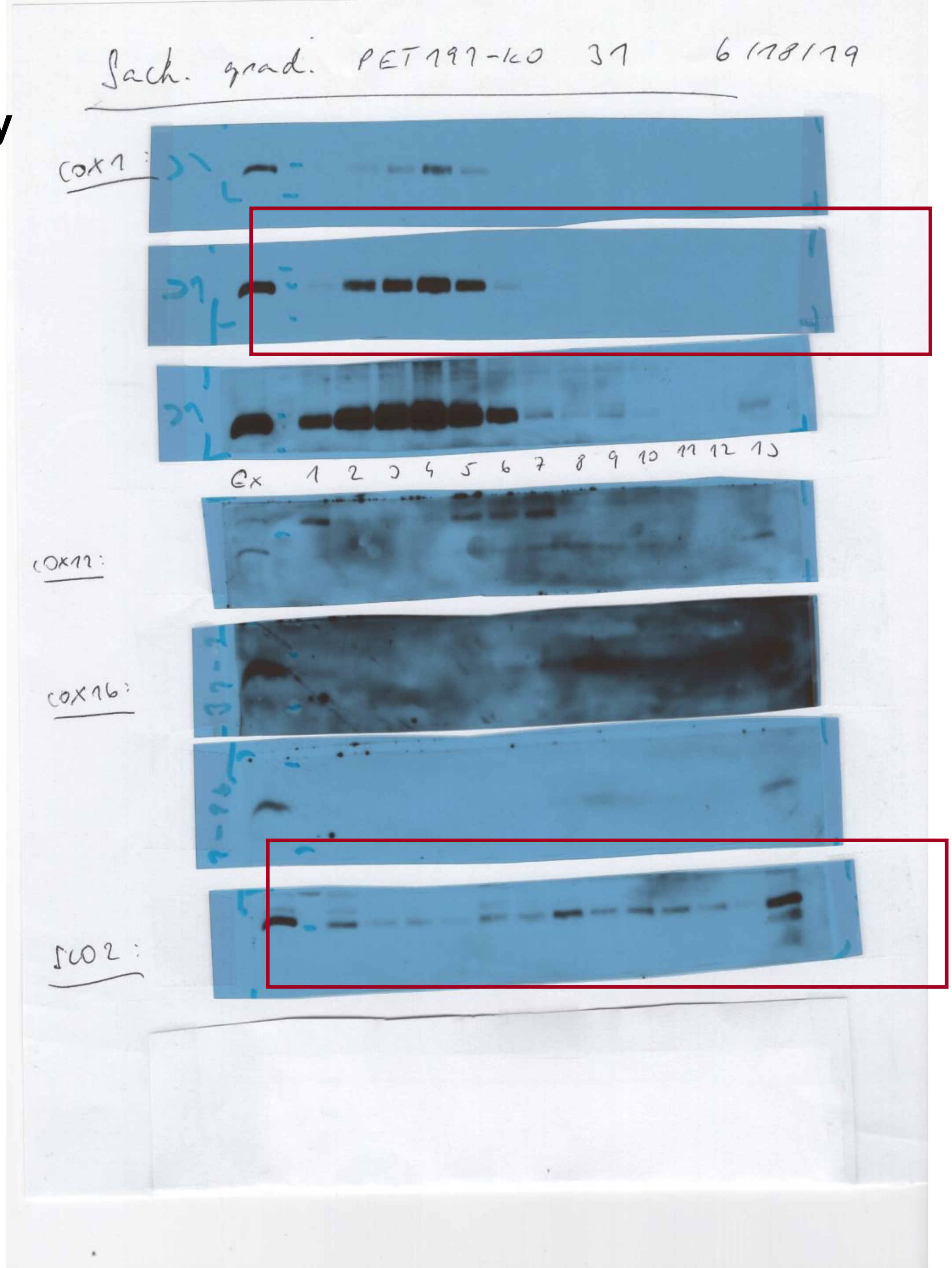

COX1

SCO2

Supplementary Fig. 8C

PET191-KO

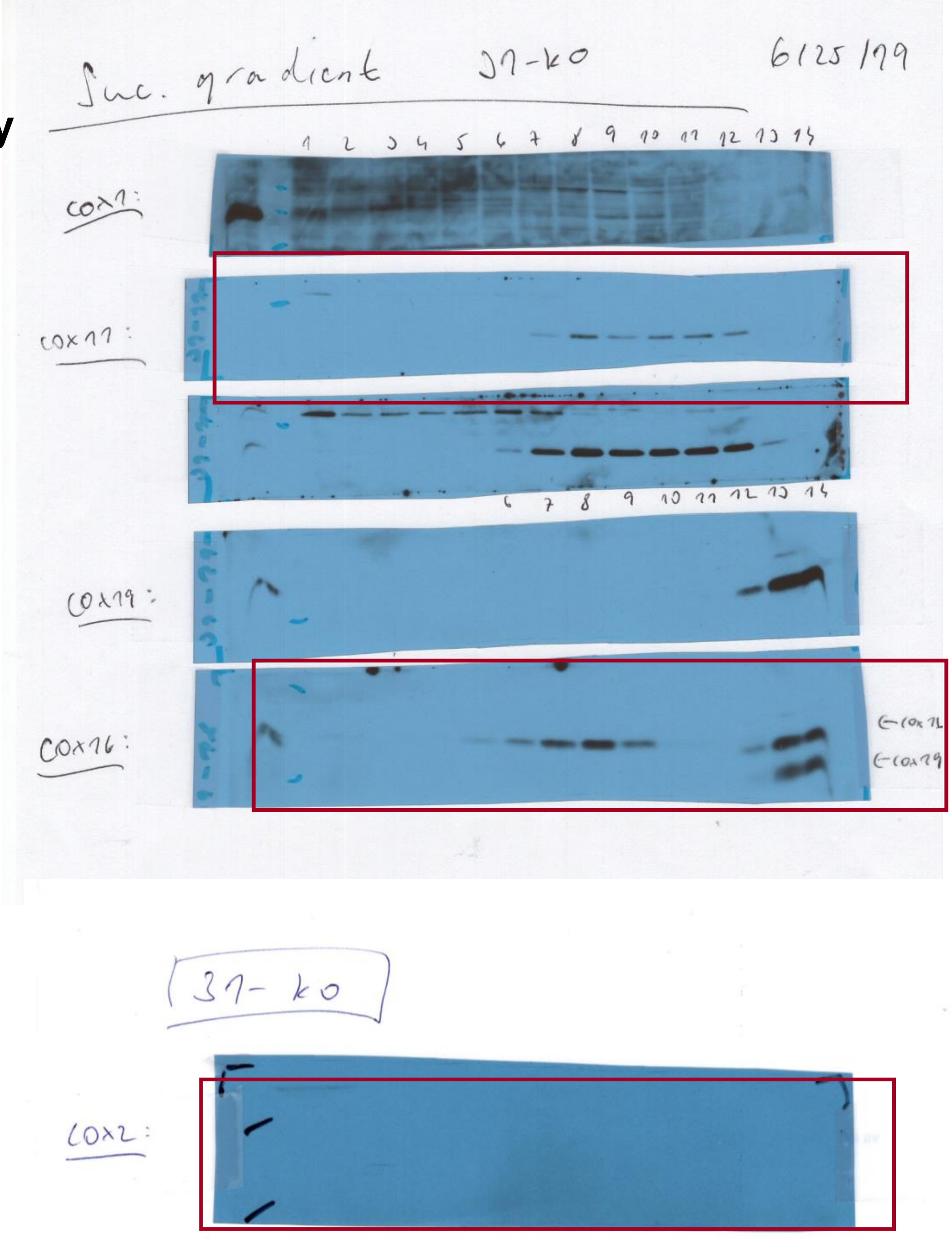

COX11

COX16
COX19

COX2

Supplementary Fig. 9B

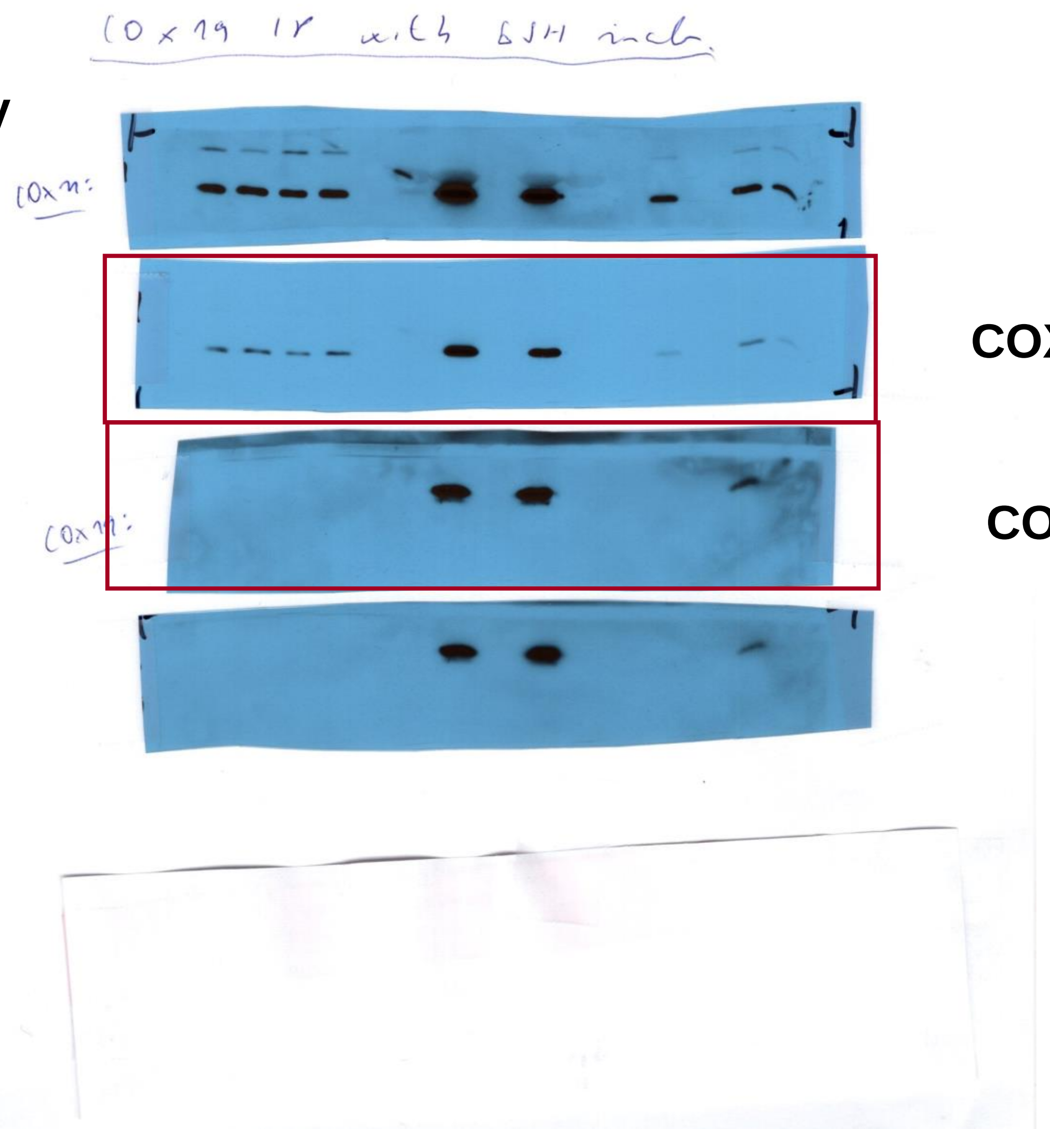

COX11

COX19

Supplementary Fig. 10A

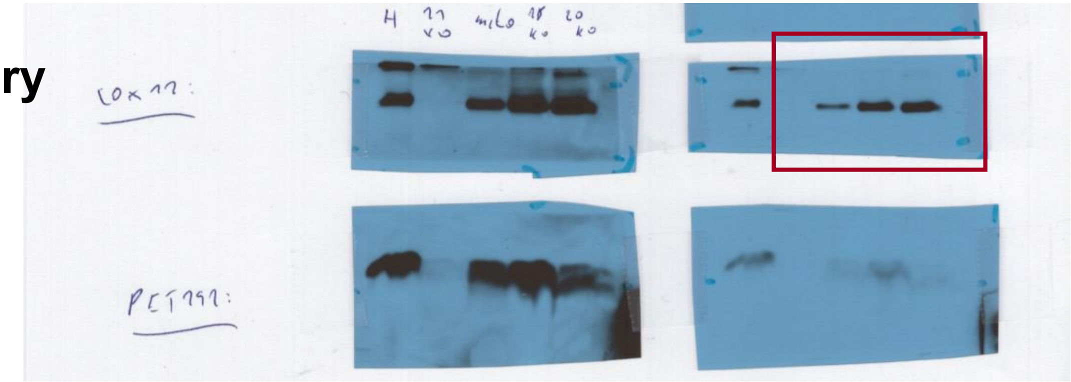

COX11

ACTIN

COX1

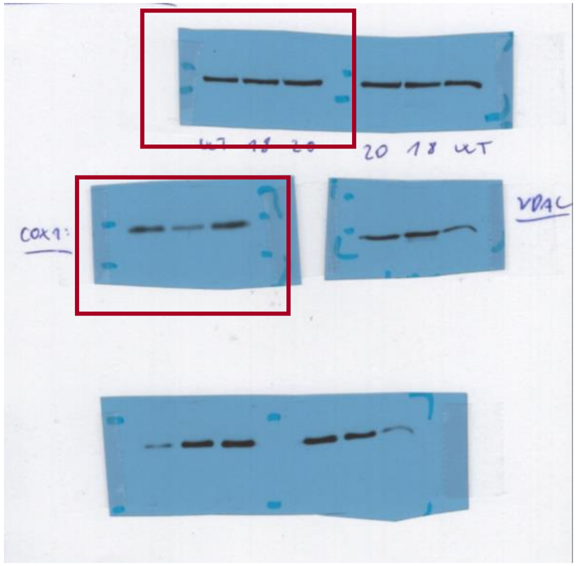

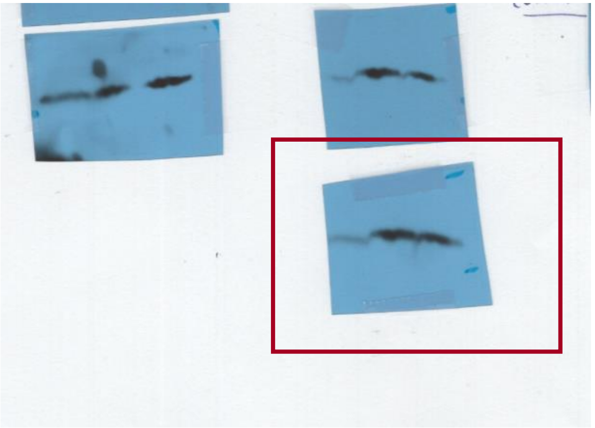

COX17

Supplementary Fig. 10A

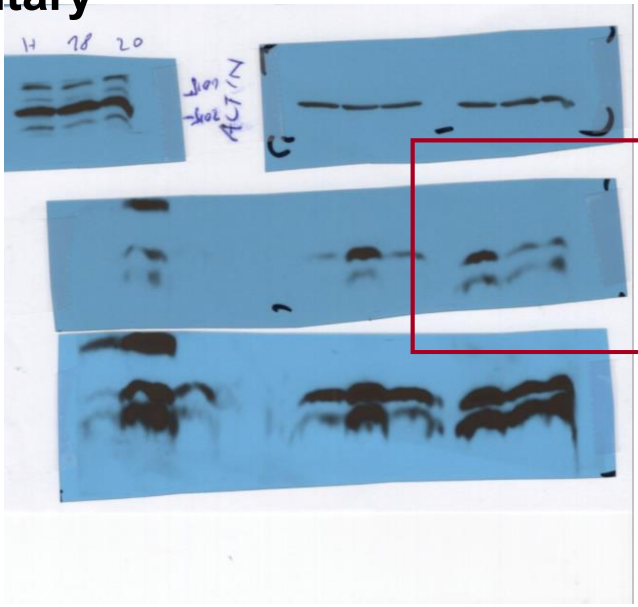

COA6
PET191

Missing actin, cox10, sco1

SCO1 →

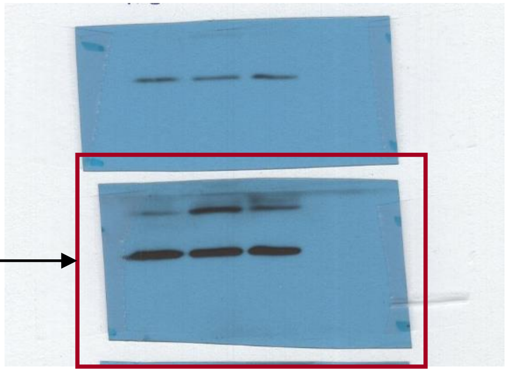

ACTIN

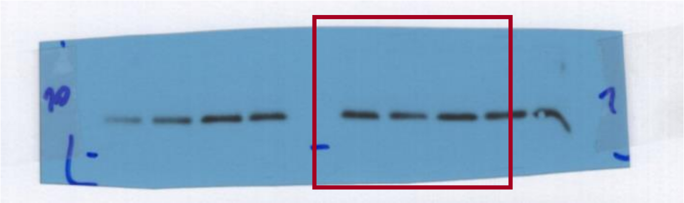

COX10

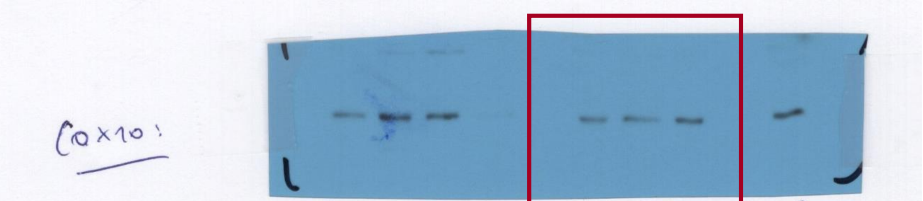

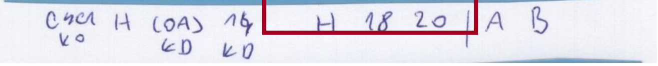

Supplementary Fig. 10A

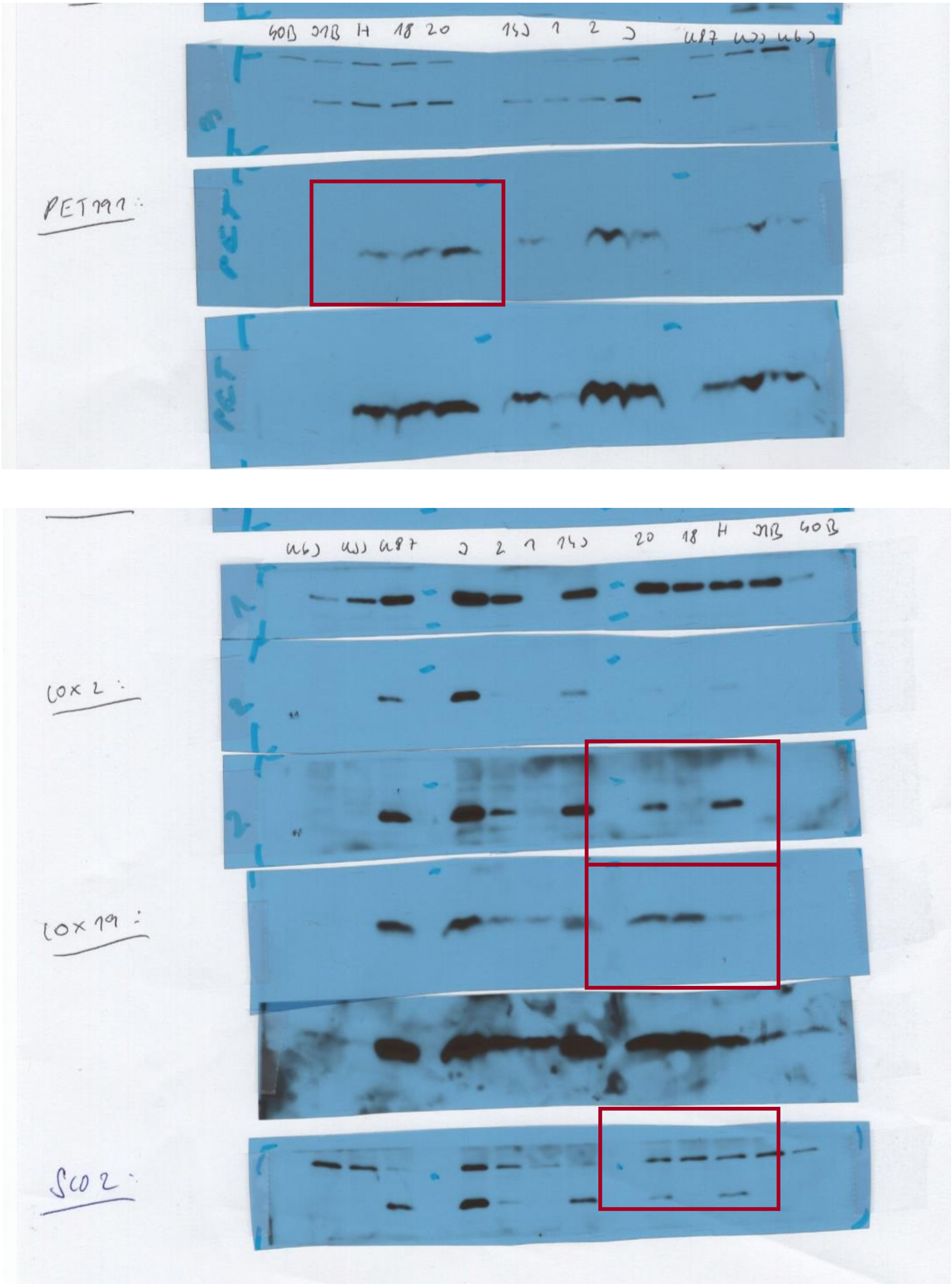

PET191

COX2

COX19

SCO2

Supplementary Fig. 10B

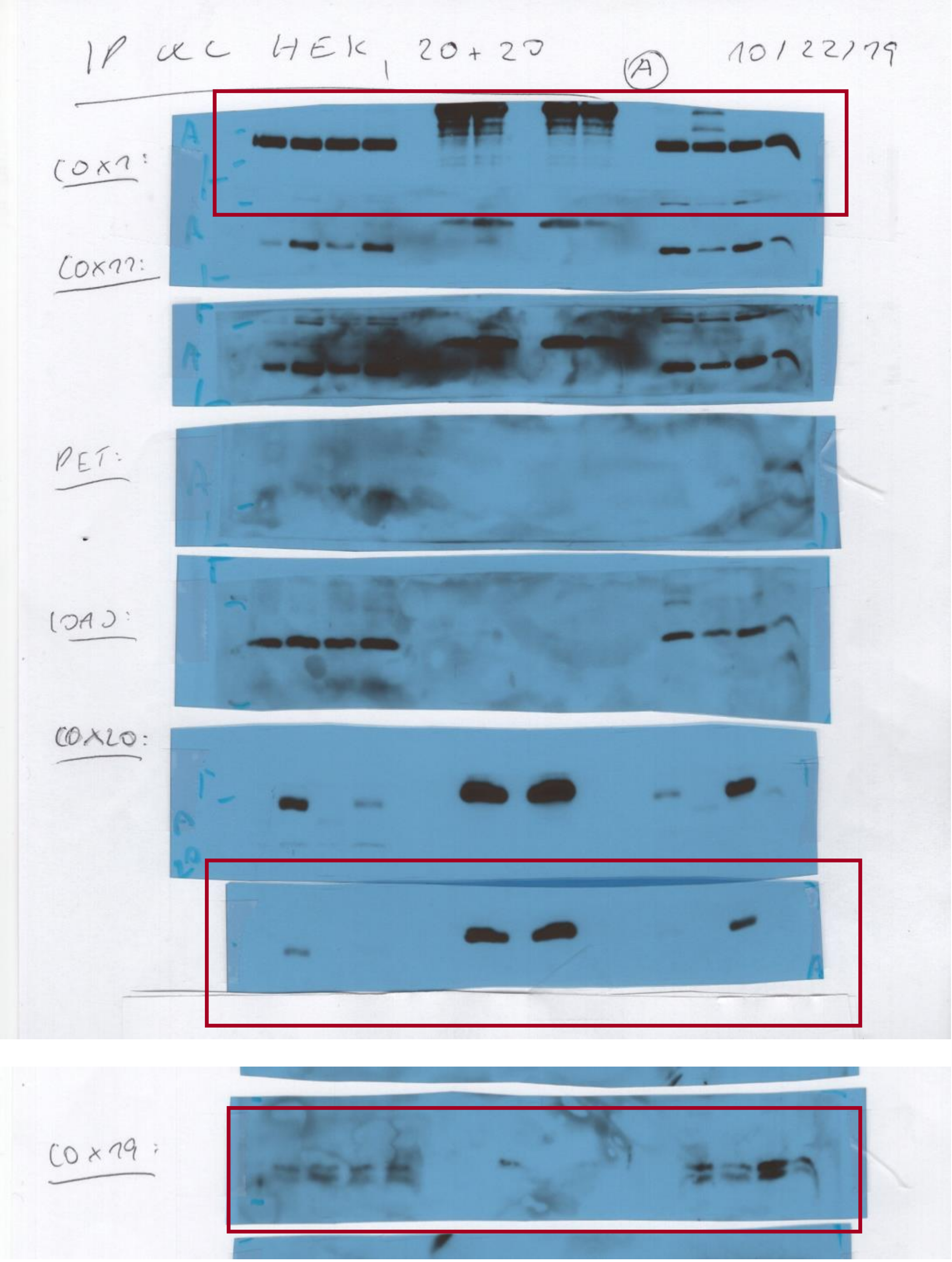

COX1

COX20-FLAG

COX19

Supplementary Fig. 10B

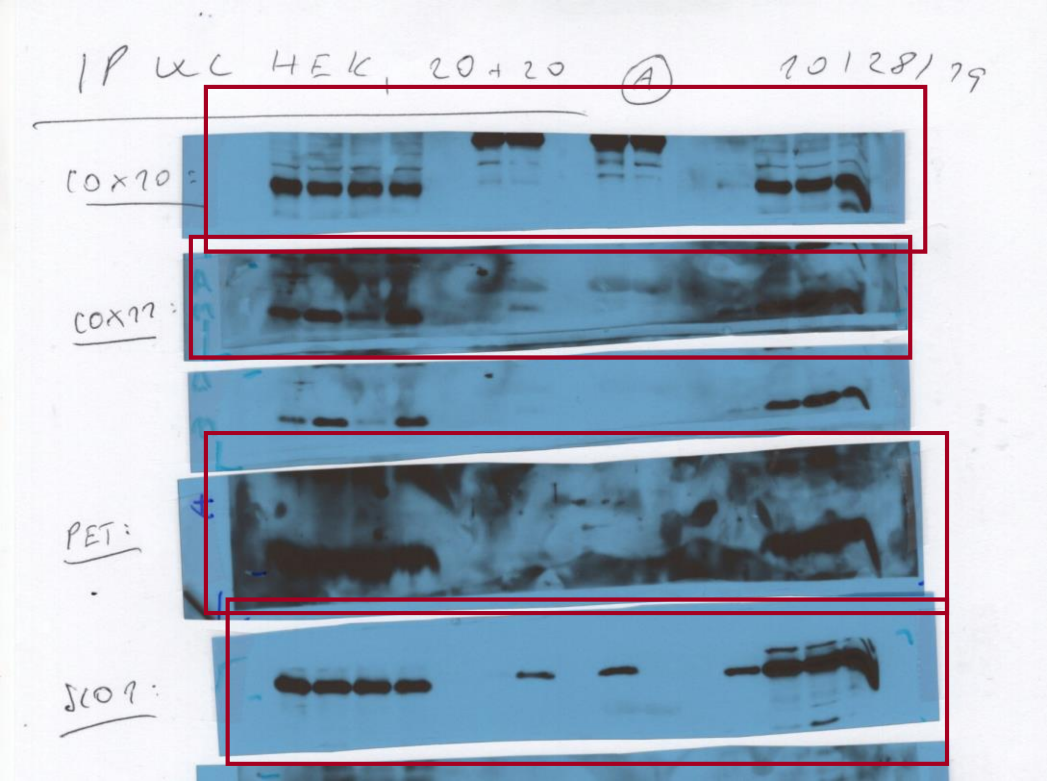

COX10

COX11

PET191

SCO1

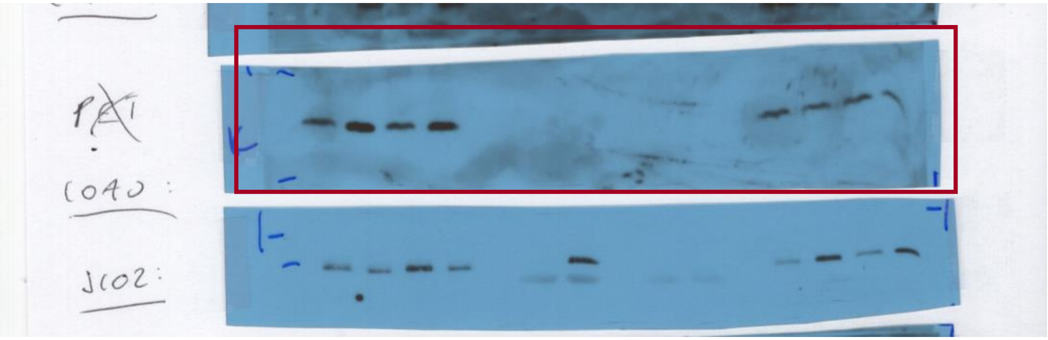

COA3

Supplementary Fig. 10B

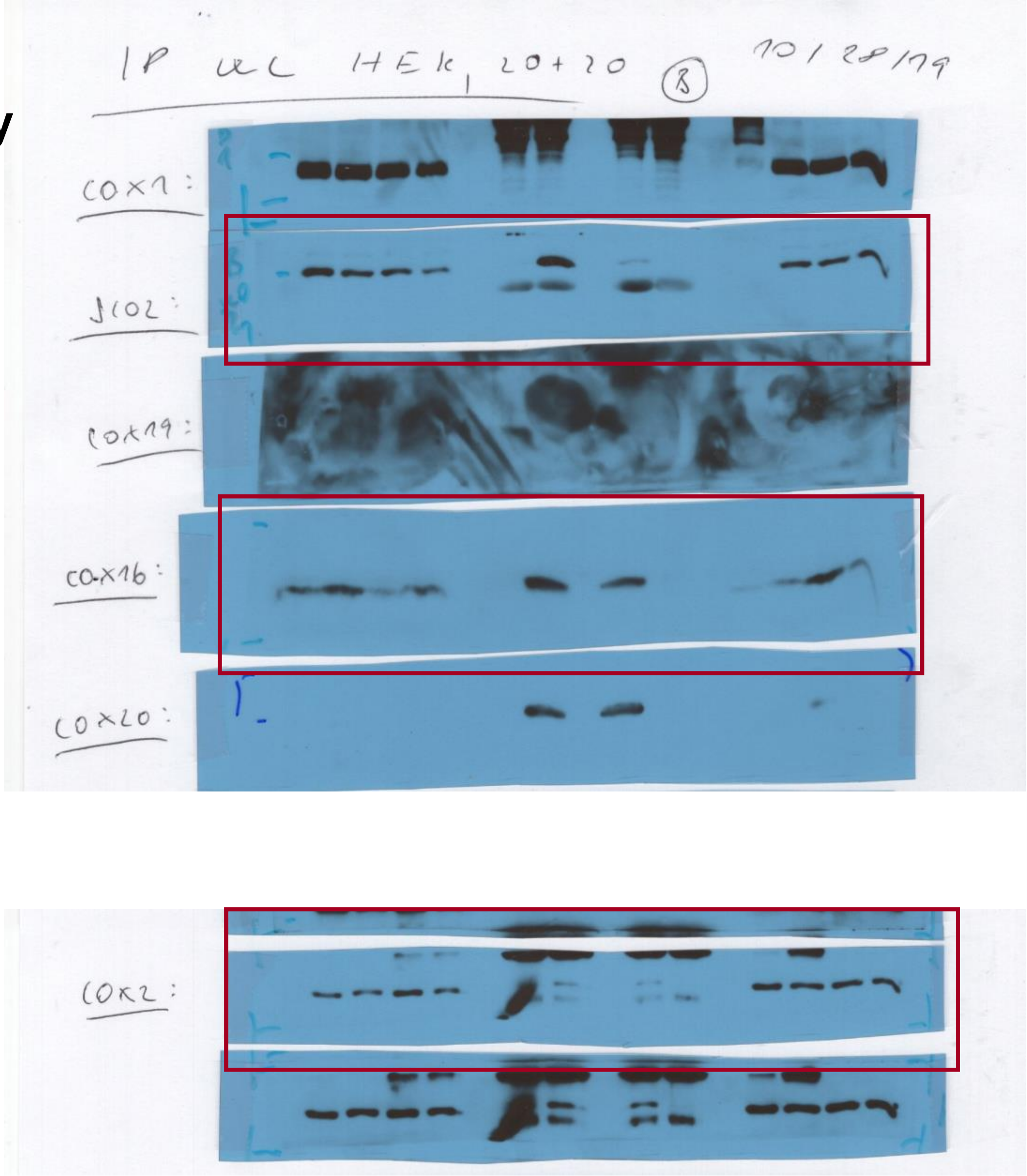

SCO2

COX16

COX2

Supplementary Fig. 10C

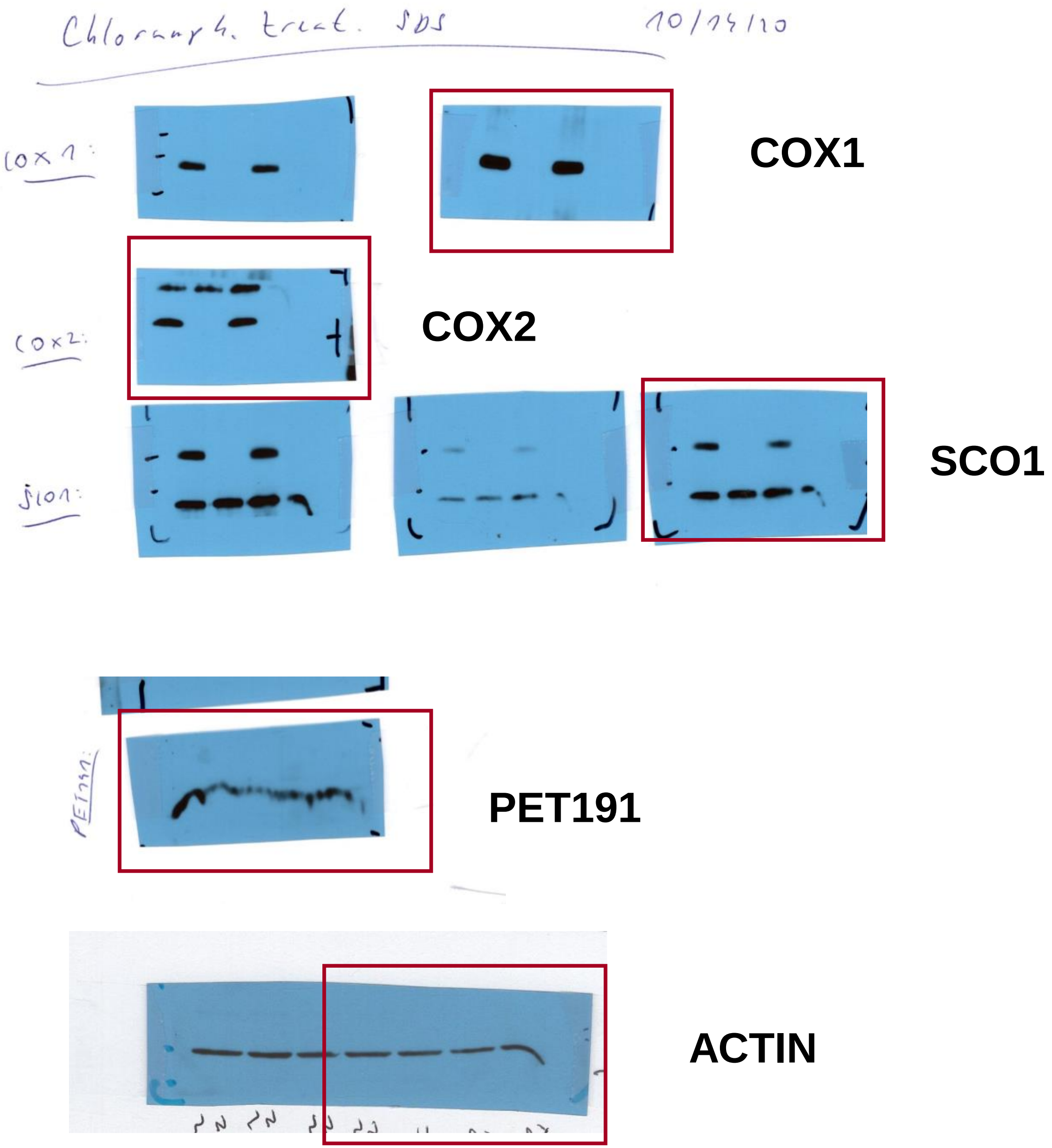

Supplementary Fig. 10D

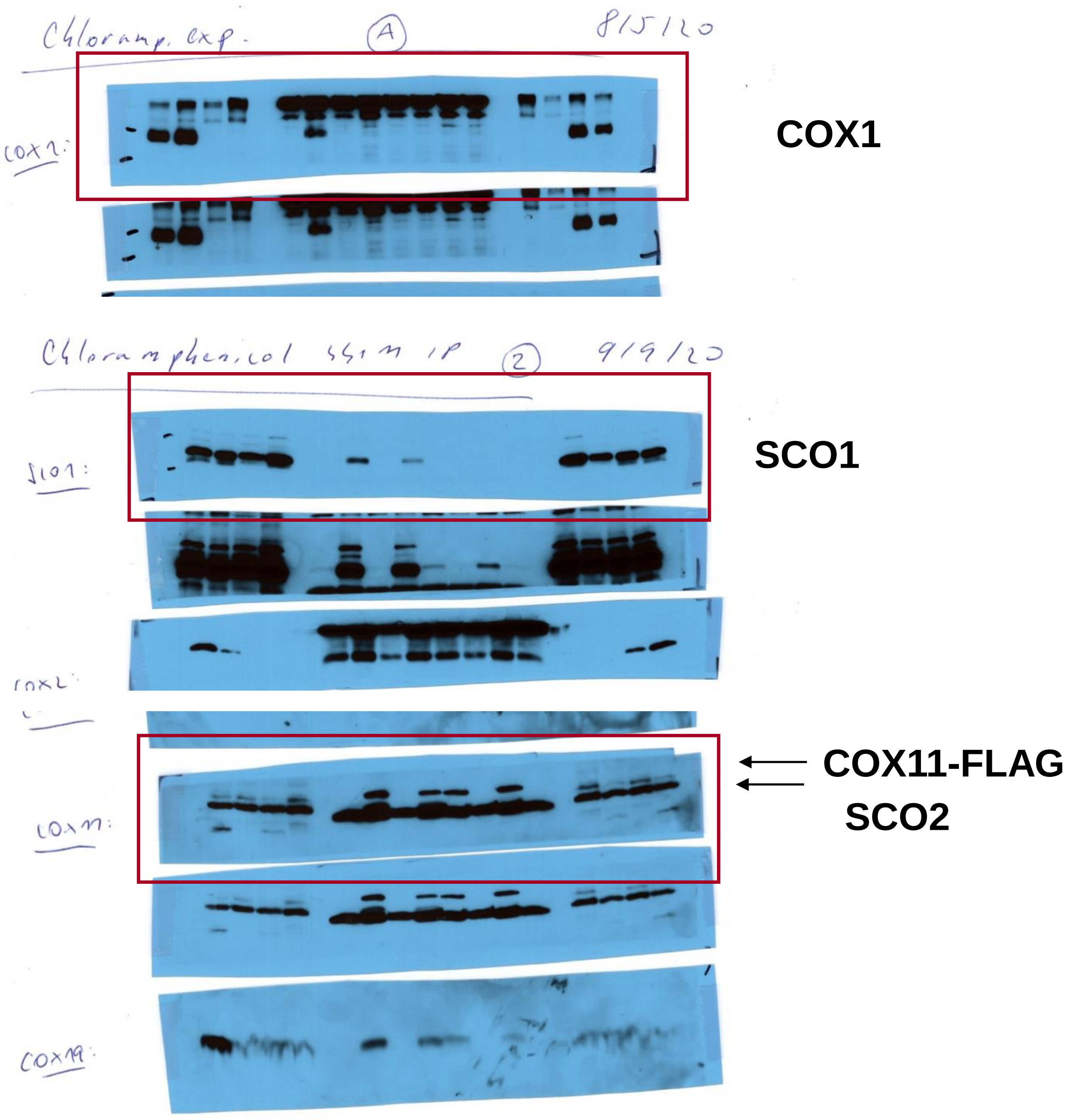

Supplementary Fig. 10D

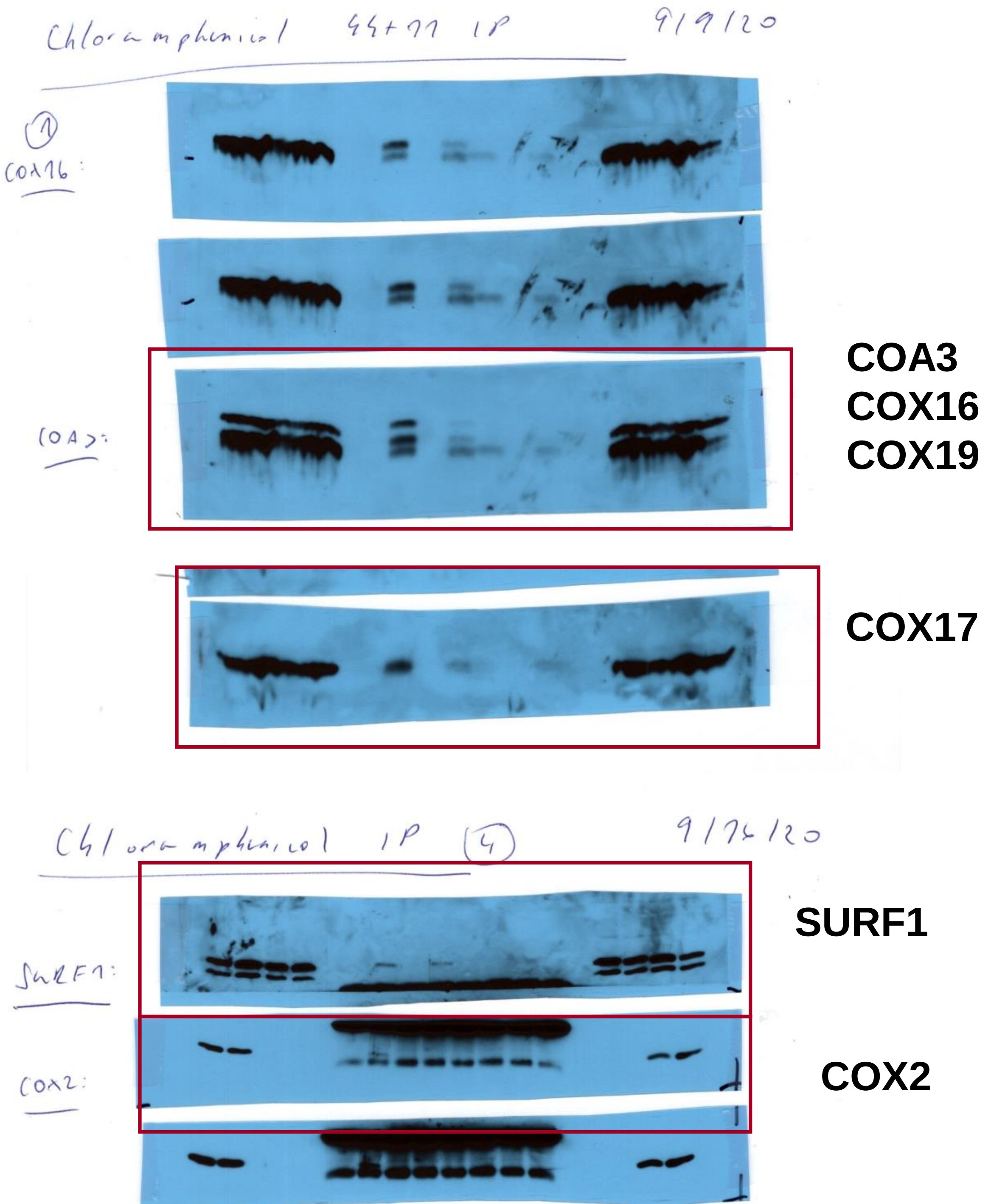

Supplementary Fig. 10D

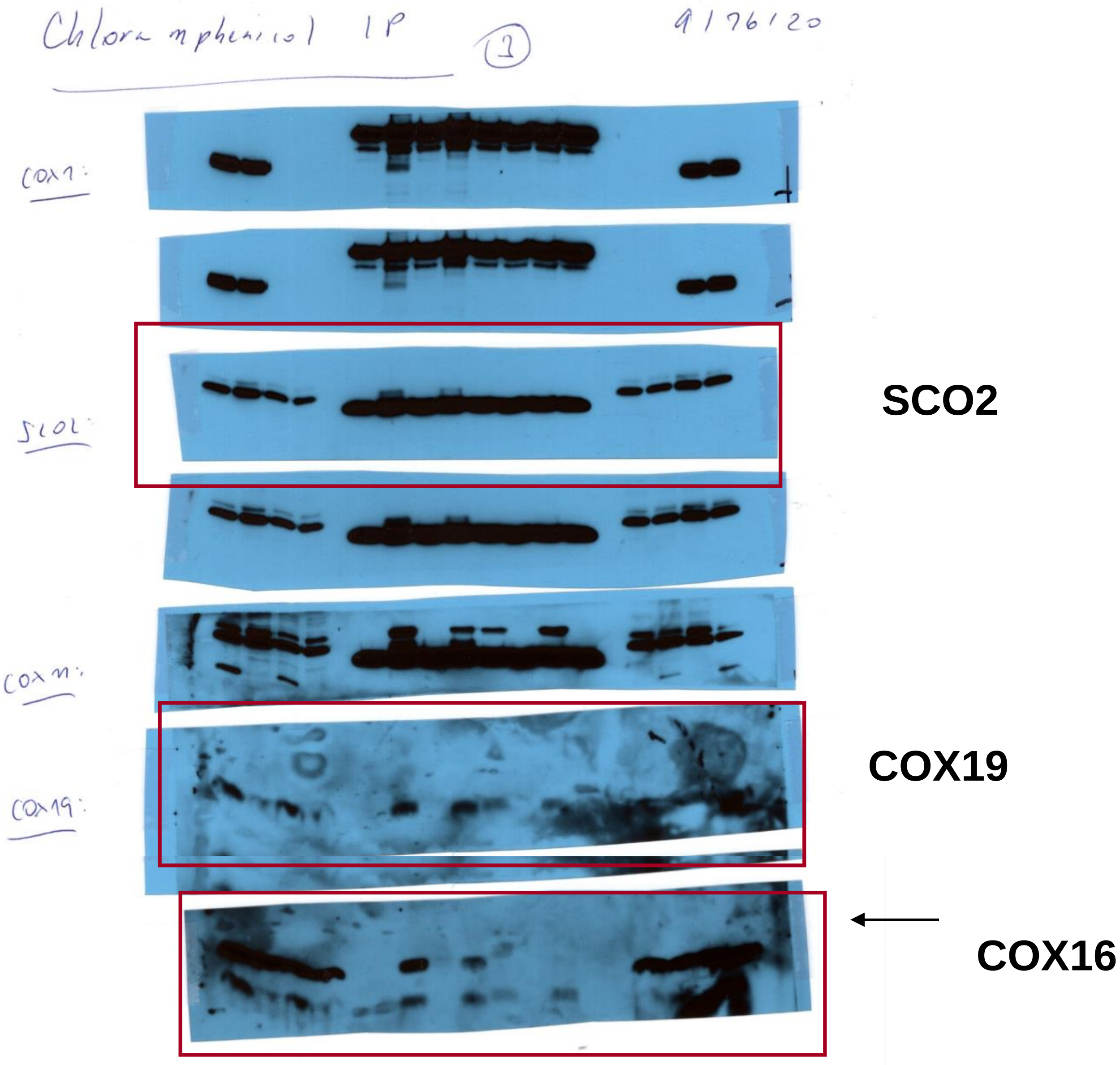

Supplementary Fig. 10D

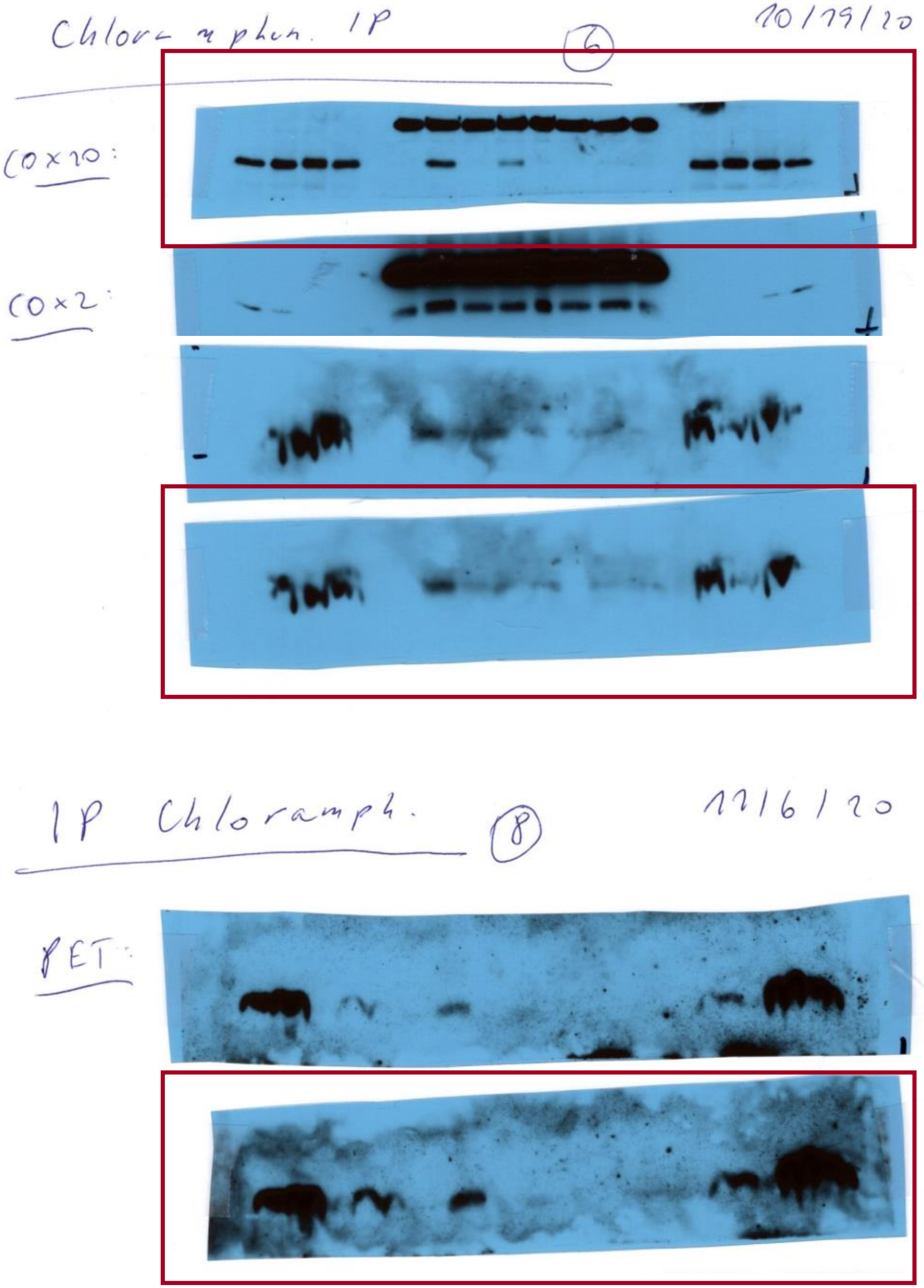

COX10

COA6

PET191

Supplementary Fig. 10E

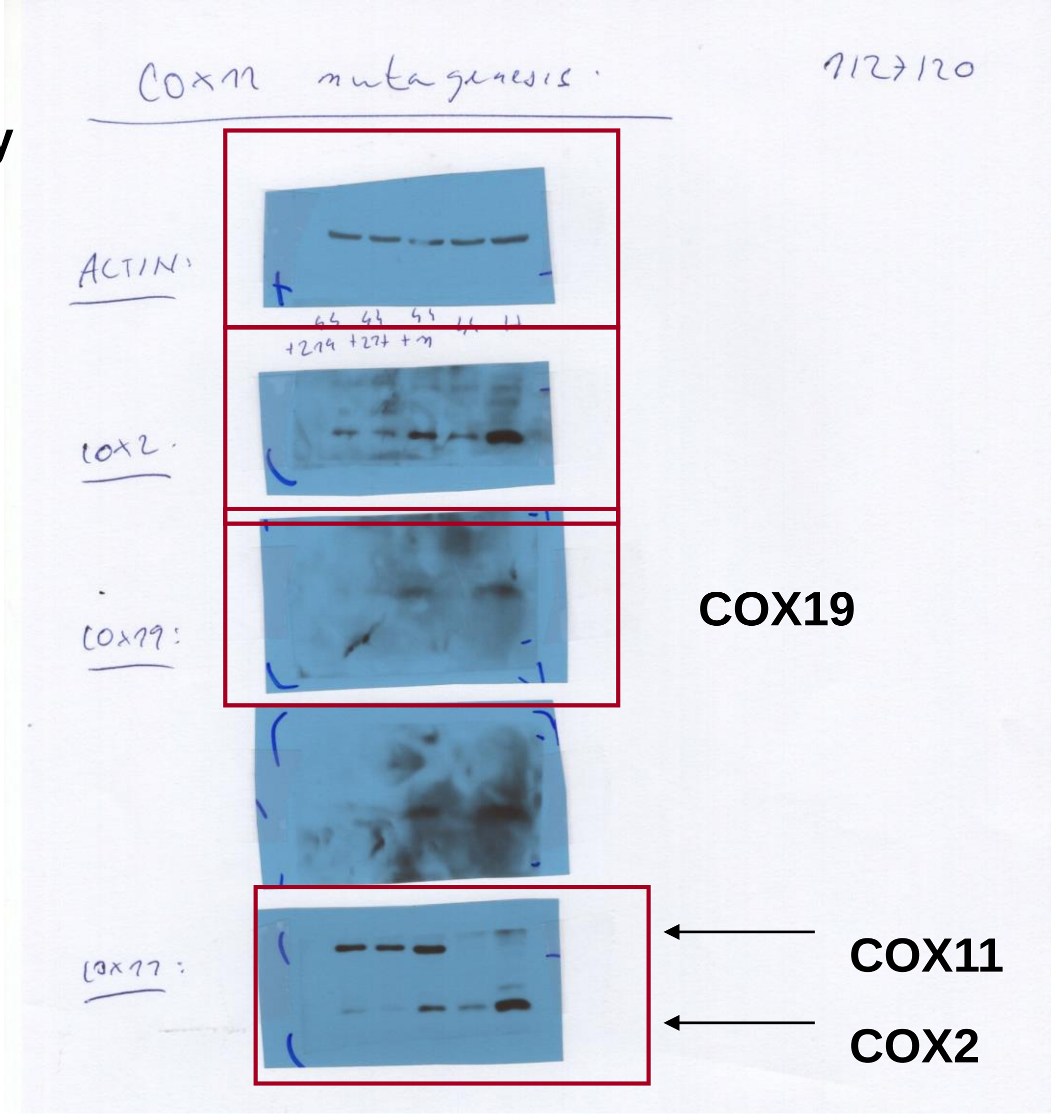

Different from pa

Supplementary Fig. 10G

COX1

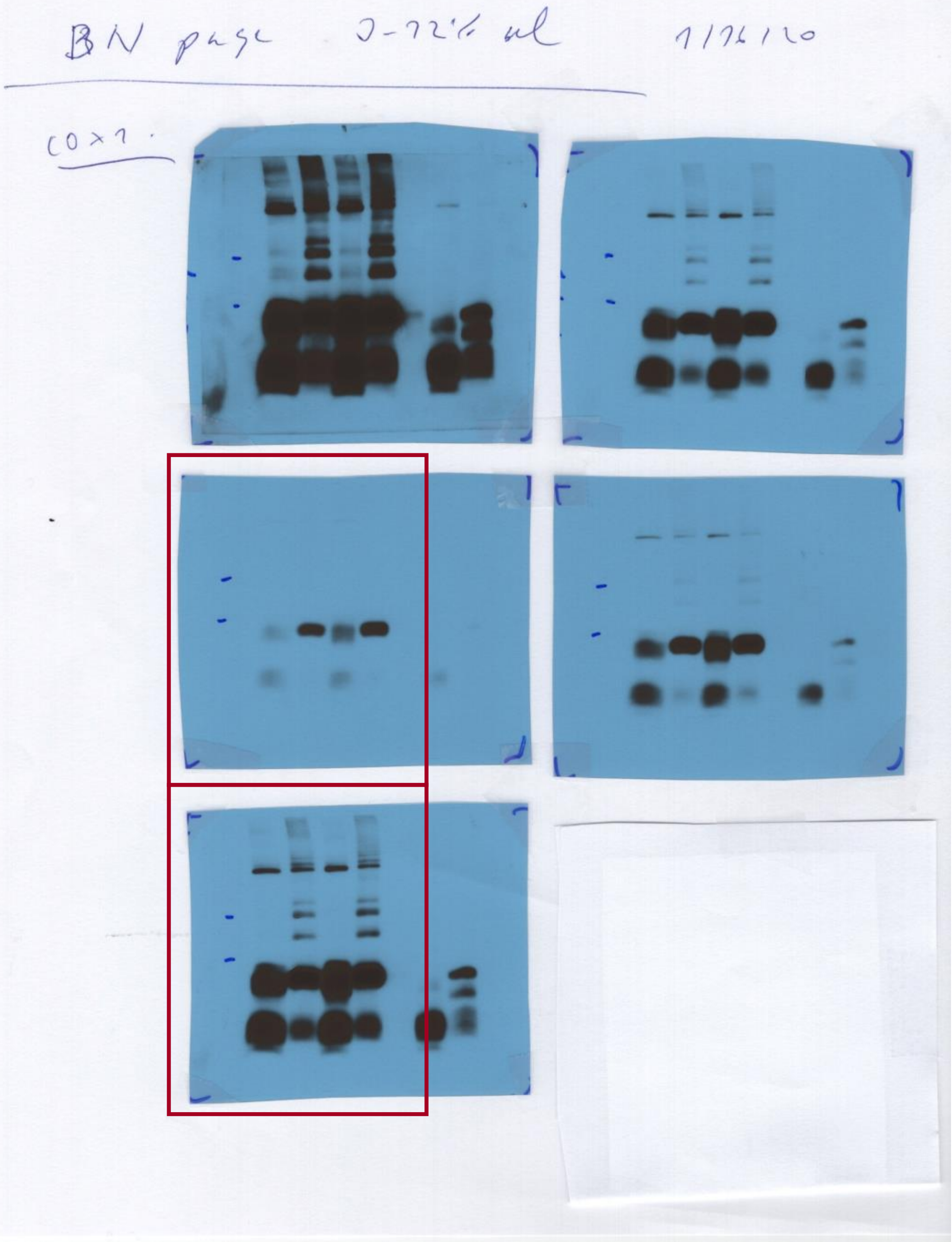

Supplementary Fig. 10G

COX5B

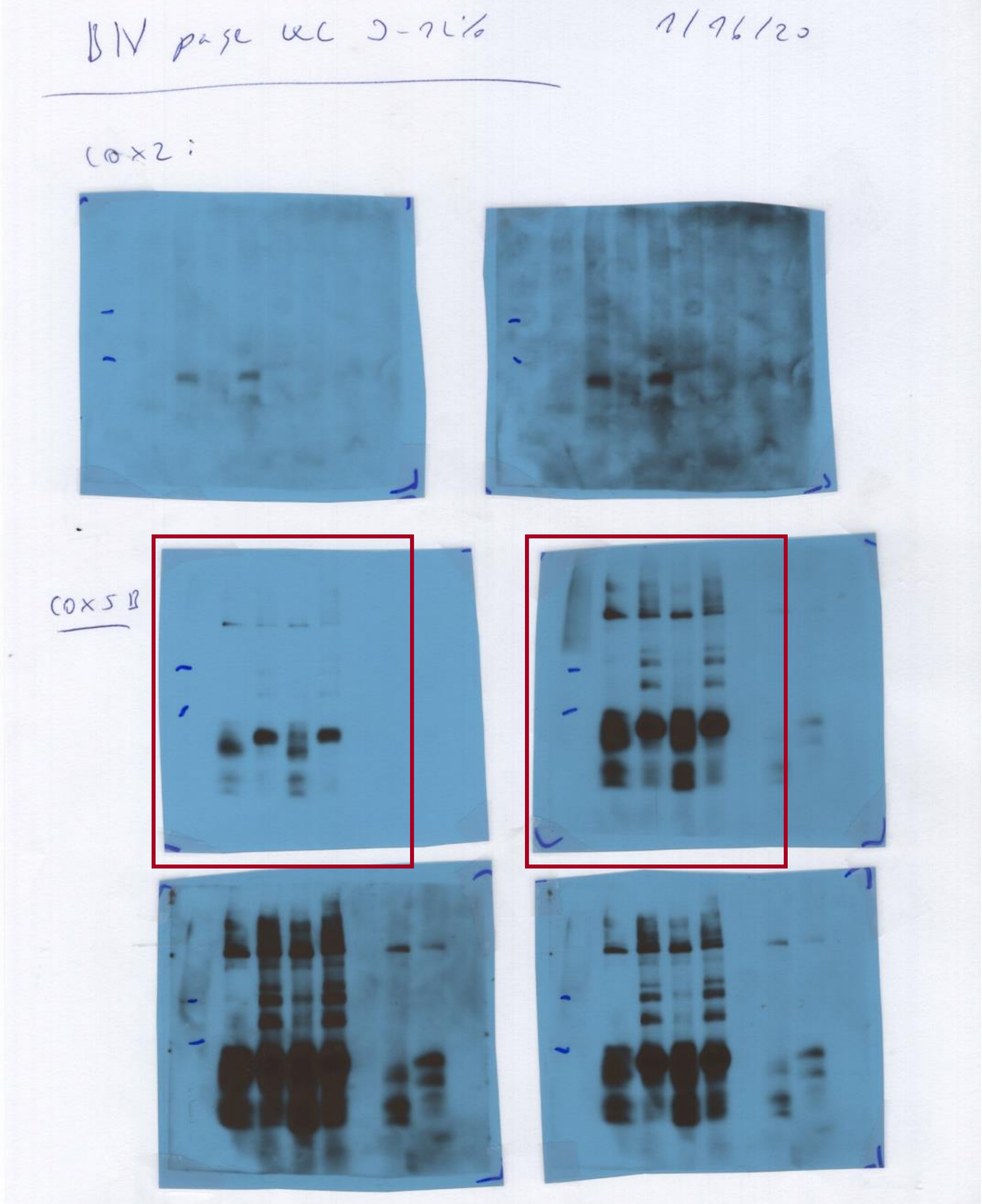

Supplementary Fig. 10H

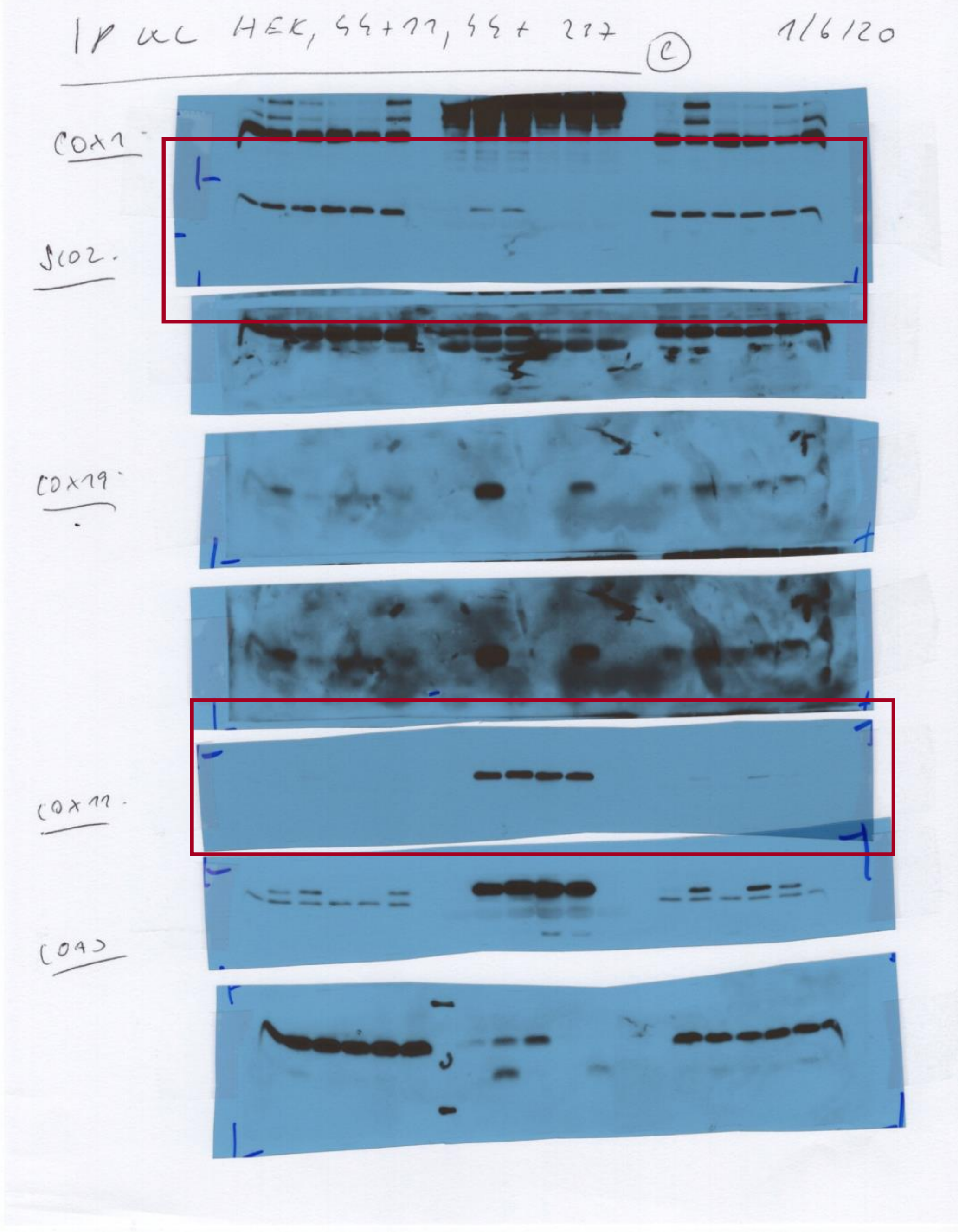

SCO2

COX11-FLAG

Supplementary Fig. 10H

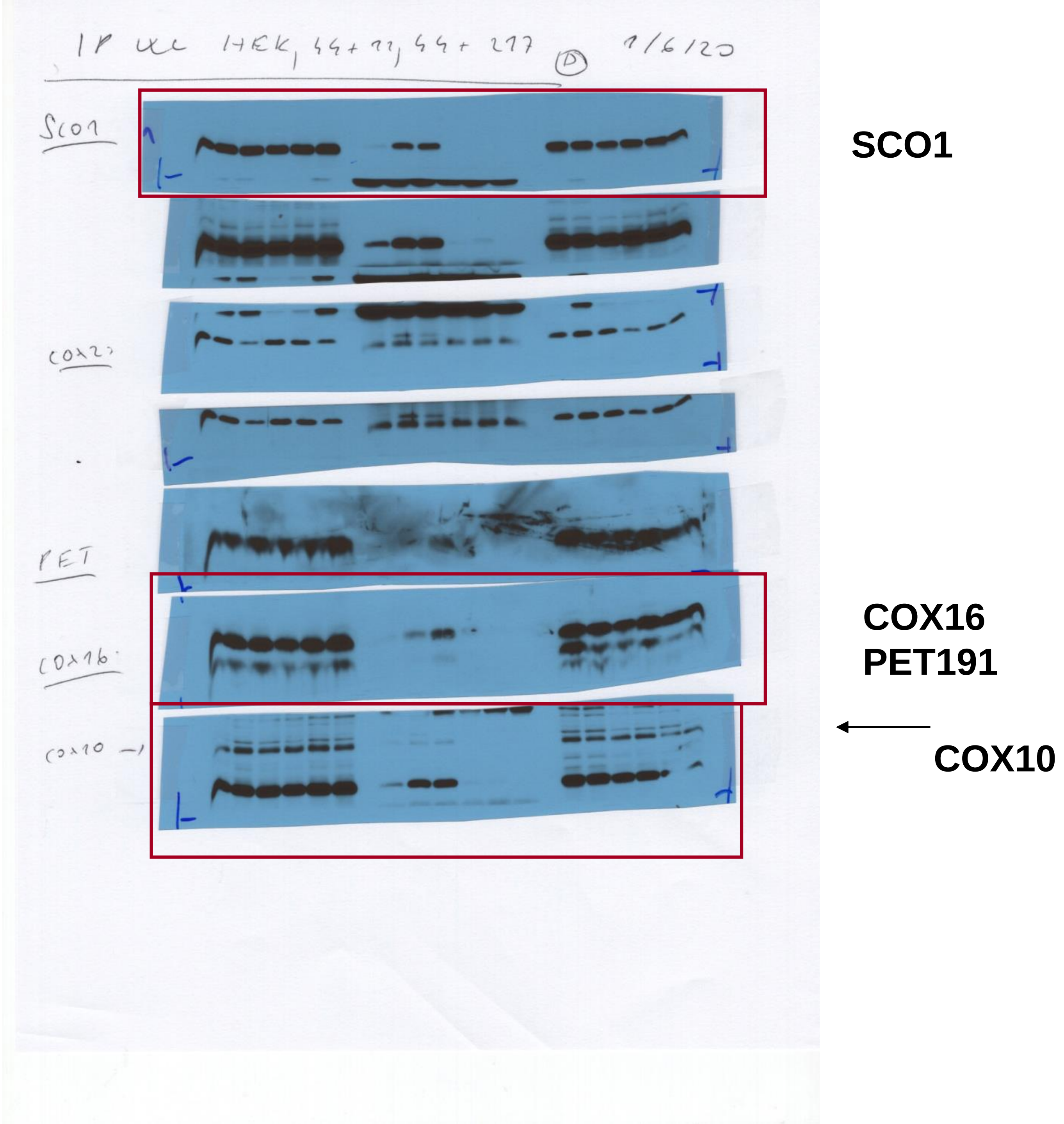

Supplementary Fig. 10H

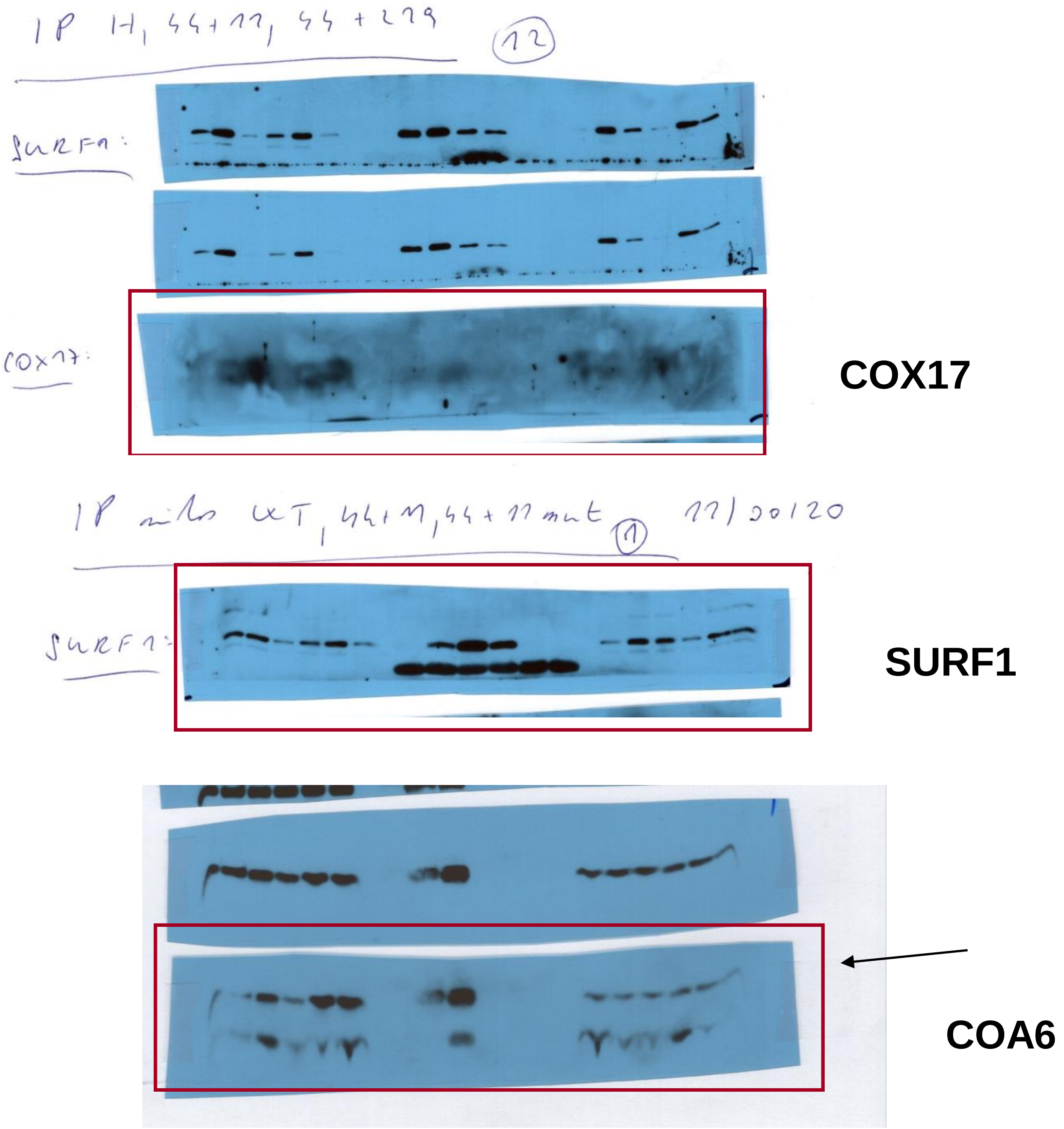

Supplementary Fig. 10H

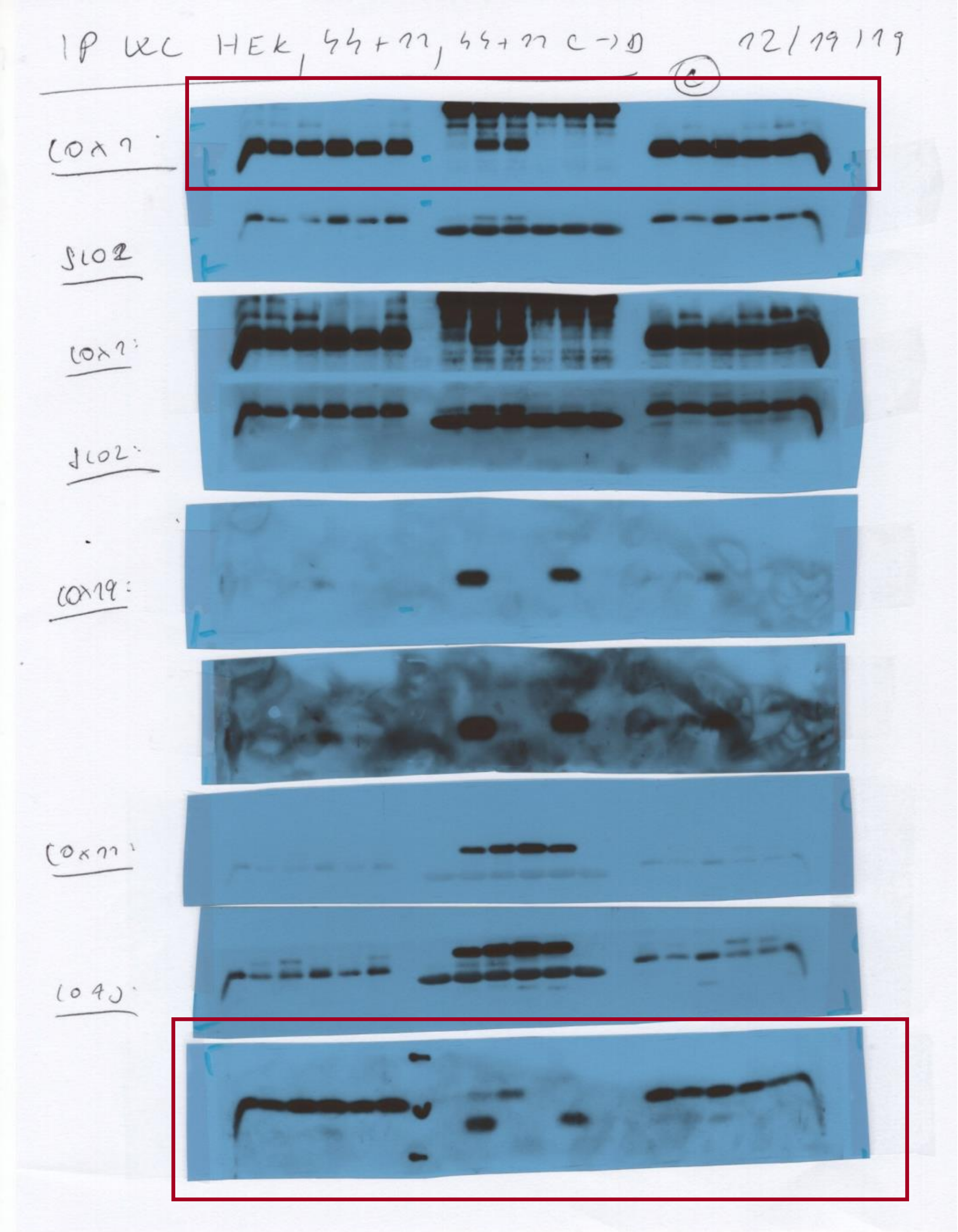

COA3
COX19

Supplementary Fig. 10H

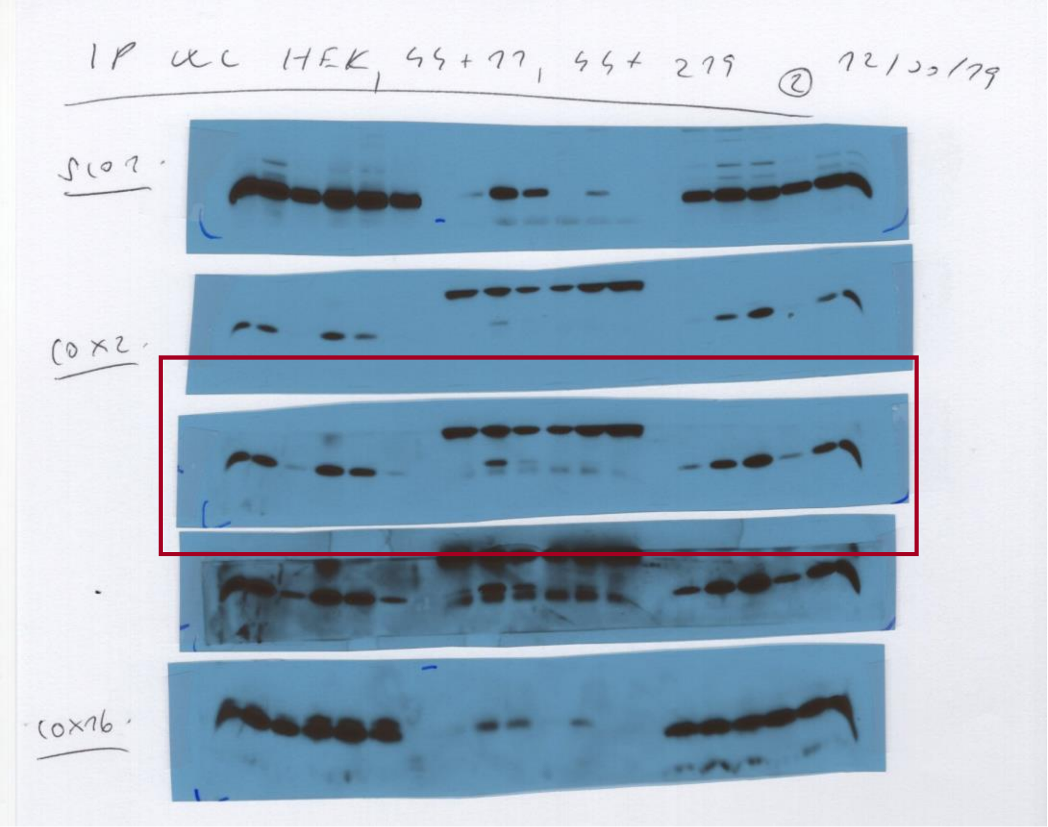

COX2

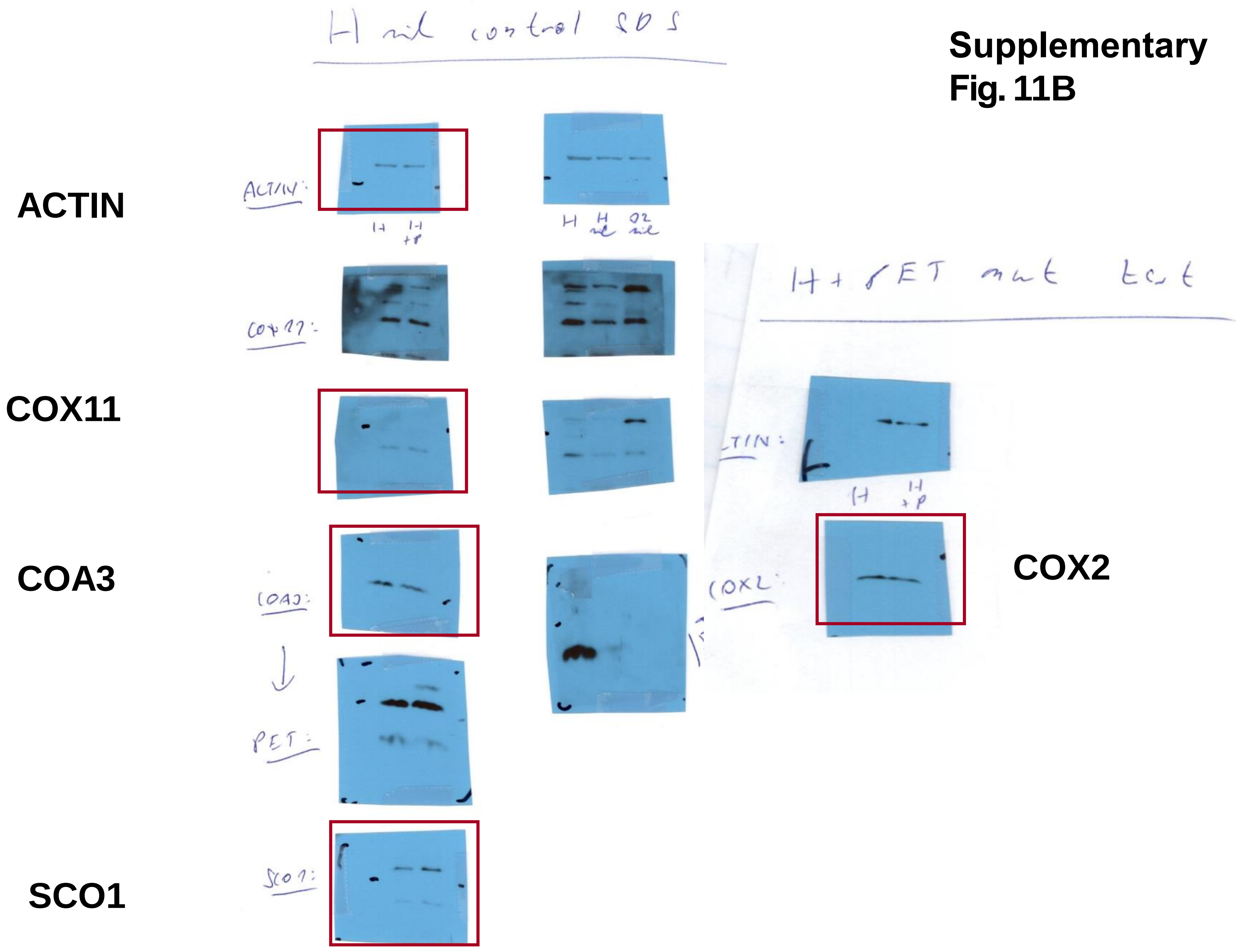

Supplementary Fig. 11B

Supplementary Fig. 12A

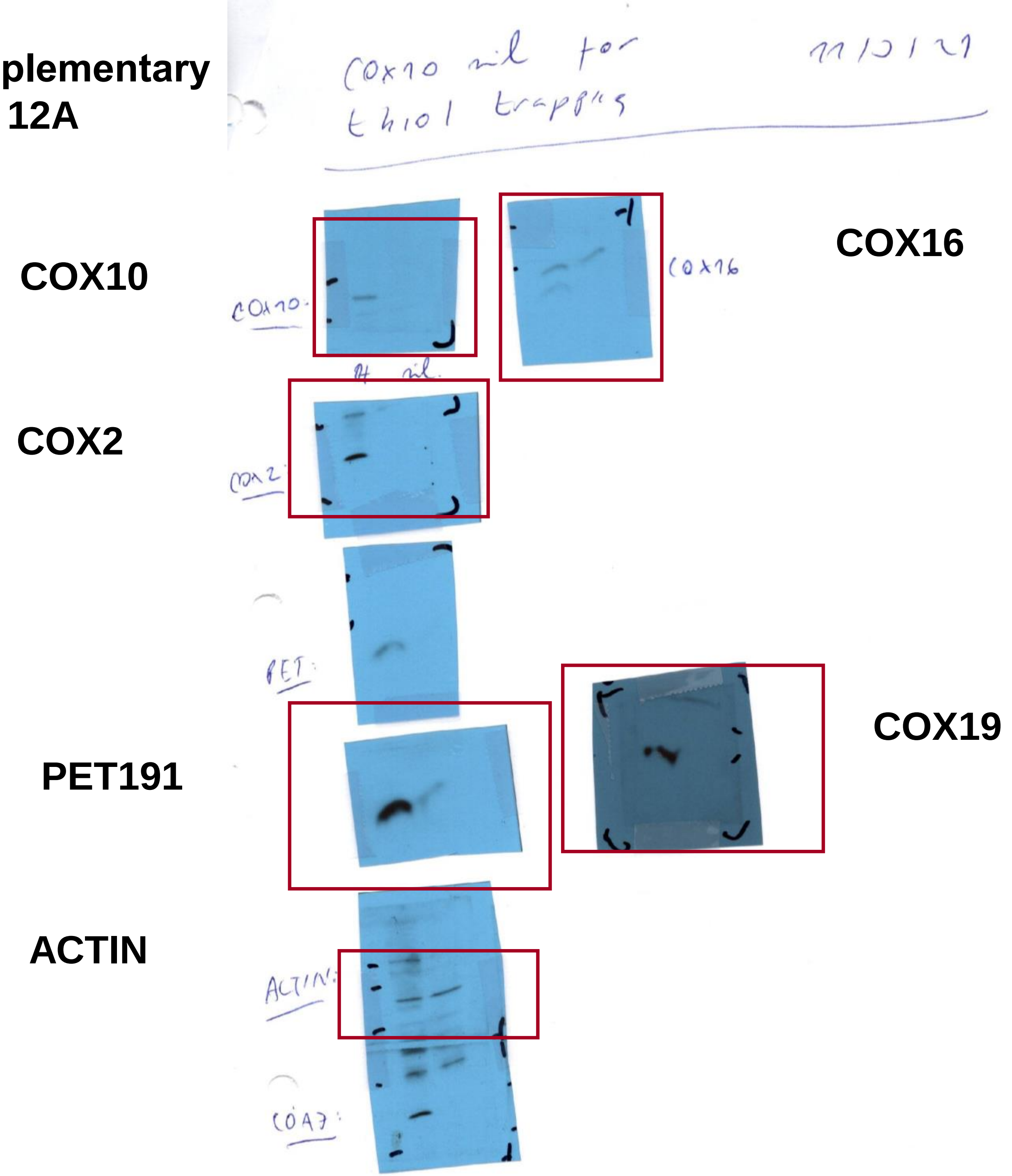

Supplementary Fig. 12A

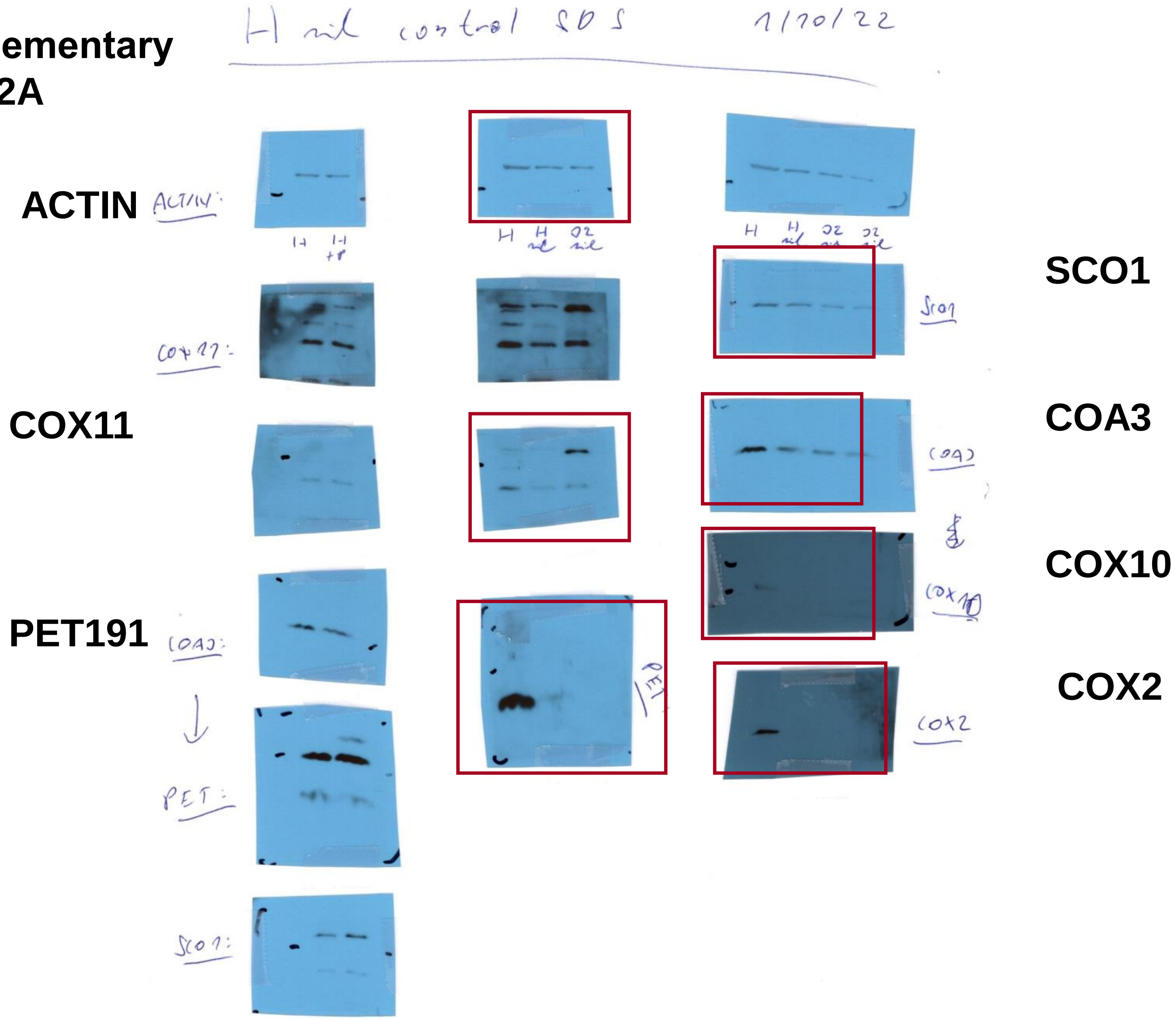

Supplement: Supplementary file 1 — Supplementary Information [file 41467_2022_31413_MOESM1_ESM.pdf]
