# Supplementary material for: Coordination of metal center biogenesis in human cytochrome c oxidase
Source: Nat Commun. 2022 Jun 24;13:3615. doi: 10.1038/s41467-022-31413-1 (PMC9232578; doi:10.1038/s41467-022-31413-1)
Supplement: Supplementary file 3 — Reporting Summary [file 41467_2022_31413_MOESM3_ESM.pdf]

nature portfolio

Corresponding author(s): Antoni Barrientos

Last updated by author(s): Jun 6, 2022

Reporting Summary

Nature Portfolio wishes to improve the reproducibility of the work that we publish. This form provides structure for consistency and transparency in reporting. For further information on Nature Portfolio policies, see our Editorial Policies and the Editorial Policy Checklist.

Statistics

For all statistical analyses, confirm that the following items are present in the figure legend, table legend, main text, or Methods section.

n/a	Confirmed	
☐	☒	The exact sample size (*n*) for each experimental group/condition, given as a discrete number and unit of measurement
☐	☒	A statement on whether measurements were taken from distinct samples or whether the same sample was measured repeatedly
☐	☒	The statistical test(s) used AND whether they are one- or two-sided<br>*Only common tests should be described solely by name; describe more complex techniques in the Methods section.*
☒	☐	A description of all covariates tested
☒	☐	A description of any assumptions or corrections, such as tests of normality and adjustment for multiple comparisons
☐	☒	A full description of the statistical parameters including central tendency (e.g. means) or other basic estimates (e.g. regression coefficient) AND variation (e.g. standard deviation) or associated estimates of uncertainty (e.g. confidence intervals)
☐	☒	For null hypothesis testing, the test statistic (e.g. *F*, *t*, *r*) with confidence intervals, effect sizes, degrees of freedom and *P* value noted<br>*Give P values as exact values whenever suitable.*
☒	☐	For Bayesian analysis, information on the choice of priors and Markov chain Monte Carlo settings
☒	☐	For hierarchical and complex designs, identification of the appropriate level for tests and full reporting of outcomes
☒	☐	Estimates of effect sizes (e.g. Cohen's *d*, Pearson's *r*), indicating how they were calculated

Our web collection on statistics for biologists contains articles on many of the points above.

Software and code

Policy information about availability of computer code

Data collection	ImageJ software v 1.53r , Adobe Photoshop, Scaffold v 5., GraphPad Prism Software v.9.3.1
Data analysis	Statistical analyses were preformed using GraphPad Prism v9.3.1, with either Anova or "Multiple t tests -- one per row".

For manuscripts utilizing custom algorithms or software that are central to the research but not yet described in published literature, software must be made available to editors and reviewers. We strongly encourage code deposition in a community repository (e.g. GitHub). See the Nature Portfolio guidelines for submitting code & software for further information.

Data

Policy information about availability of data

All manuscripts must include a data availability statement. This statement should provide the following information, where applicable:

- Accession codes, unique identifiers, or web links for publicly available datasets
- A description of any restrictions on data availability
- For clinical datasets or third party data, please ensure that the statement adheres to our policy

All unique/stable reagents generated in this study (plasmids and cell lines) are available from the corresponding author with a completed Materials Transfer Agreement. All raw data is either included in the manuscript or it will be provided upon request.

We have reported a Source Data file including representative source data for 1. mitochondrial cytochrome spectra analyses, 2. Representative data panels of the stable and transient protein interactomes analyses of CcO assembly chaperones: (1) COX11, (2) COX19, (3) PET191, (4) COX17, (5) COX10, (6) COA6, (7) SCO1, (8) SCO2, (9) COX16. The images correspond to the immunoprecipitation analyses of the indicated C-terminus FLAG-tagged proteins in several cellular backgrounds. The experiments were performed in at least biological triplicates, the images were digitalized, used for densitometry analyses, and the quantification of the data

condensed in the heat maps presented in Figure 3C-D. 3. uncropped and unprocessed images of all immunoblots, and 4. source data for all graphs are provided with this paper

The MS data has been uploaded to Mendeley Data and are available at: Barrientos, Antonio (2021), "Nyvltova et al. MS Data", Mendeley Data, V1, doi: 10.17632/7jsmsm3xrn.1
The ICP-MS data has been uploaded to Mendeley Data and are Nyvltova, Eva (2022), "Nyvltova et al. ICP-MS Data", Mendeley Data, V1, doi: 10.17632/w2yk8g568t.1

Field-specific reporting

Please select the one below that is the best fit for your research. If you are not sure, read the appropriate sections before making your selection.

☒ Life sciences ☐ Behavioural & social sciences ☐ Ecological, evolutionary & environmental sciences

For a reference copy of the document with all sections, see nature.com/documents/nr-reporting-summary-flat.pdf

Life sciences study design

All studies must disclose on these points even when the disclosure is negative.

Sample size

We used several cell lines to be analyzed for a few quantitative parameters. All experiments were performed at least in triplicate, which was considered sufficient when applying standard statistics. For experiments with higher variability such as redox state of SCO1 and SCO2 we performed six independent repetitions (Fig 4B, D, G, and I), for copper measurements we performed 9 repetitions (Fig 6D) or cell viability measurements we performed 9 repetitions (Fig 6B) No special algorithms were used to determine sample size.

Data exclusions

No data has been excluded

Replication

Three to nine repetitions were performed to verify repeatability, using independent biological samples. All results were successfully replicated.

Randomization

Randomization was in most cases not relevant to our study since we are studying specific cell lines for a few specific quantitative parameters. The cell lines are either WT or KO for specific genes. All experiments were performed with the same set of cellular clones.
However, in the few cases in which cell cultures were supplemented with either copper (Cu) or elesclomol (ES), the plates of cell cultures that will receive Cu, ES, Cu+ES, or none for each cell line were randomly selected without applying any specific randomization method.

Blinding

All experiments, data collection, and analysis were performed mostly by a single researcher. Blinding was not possible in most cases. For protein-protein interaction studies, EN prepared the samples and run the immunoblots; then EN and AB quantified the data separately and confirmed that they agreed.

Reporting for specific materials, systems and methods

We require information from authors about some types of materials, experimental systems and methods used in many studies. Here, indicate whether each material, system or method listed is relevant to your study. If you are not sure if a list item applies to your research, read the appropriate section before selecting a response.

Materials & experimental systems

n/a	Involved in the study
☐	☒ Antibodies
☐	☒ Eukaryotic cell lines
☒	☐ Palaeontology and archaeology
☒	☐ Animals and other organisms
☒	☐ Human research participants
☒	☐ Clinical data
☒	☐ Dual use research of concern

Methods

n/a	Involved in the study
☒	☐ ChIP-seq
☒	☐ Flow cytometry
☒	☐ MRI-based neuroimaging

Antibodies

Antibodies used

We used primary antibodies against the following human proteins: COX11 (dilution 1:1,000; OriGene Tech., TA323960), PET191 (dilution 1:500; Sigma; St. Louis, MO; HPA057768), COX19 (dilution 1:500; Sigma, HPA021226), β-ACTIN (dilution 1:2,000; Proteintech; Rosemont, IL; 60008-1-Ig), COX1 (dilution 1:2,000; Abcam; Cambridge, MA; ab14705), COX2 (dilution 1:1,000; Abcam, ab110258), COA6 (dilution 1:500; Sigma, HPA028588), COX17 (dilution 1:500; OriGene Tech., TA315013), COX16 (dilution 1:1,000; Proteintech, 19425-1-AP), COX10 (dilution 1:1,000; Sigma, HPA032005), COA3 (dilution 1:1,000; Sigma, HPA031966), SCO1 and SCO2 (PRAB4980 and PRAB4982 according to P. Rehling catalog; each at dilution 1:500; kind gift of P. Rehling who generated, validated, and cited them in PMID: 29381136), CMC1 (dilution 1:1,000; Sigma, HPA043333), COX5B (dilution 1:1,000; Santa Cruz Biotech.; Dallas, TX; sc-374417), SURF1 (dilution 1:1,000; Abcam, Ab155251), CORE2 (dilution 1:2,000; Abcam, ab14745), FLAG (dilution 1:1,000; Sigma, F3165), COX20 (dilution 1:1,000; Sigma, HPA045490), HIGD2A (dilution 1:1,000; Sigma, HPA042715), HA (dilution 1:1,000; Thermo Fisher Scientific, 71-5500), Porin (dilution 1:1,000; Abcam, ab110326). Horseradish peroxidase-conjugated anti-

mouse or anti-rabbit IgGs were used as secondary antibodies (dilution 1:10,000; Rockland; Limerick, PA).

The following primary antibodies were used for yeast studies: mouse anti-porin (459500, Thermo Scientific) and rabbit anti-Pet191 (kindly provided by Dr. A. Chacinska). All antibodies were tested for reliability using specific KO strains to ensure specificity of detection.

Validation

We are providing the RRI (Resource Identification number), supporting new guidelines for Rigor and Transparency in biomedical publications:
Abs with assigned RRI:
COX11 (OriGene, TA323960; RRID: AB_2678777), PET191 (Sigma, HPA057768; RRID: AB_2683521), COX19 (Sigma, HPA021226; RRID: AB_1847176), ACTIN (Proteintech, 60008-1-Ig), COX1 (Abcam, ab14705; RRID: AB_2084810), COX2 (Abcam, ab110258; RRID: AB_10887758), COA6 (Sigma, HPA028588; RRID: AB_10601123), COX16 (Proteintech, 19425-1-AP; RRID: AB_10666854), COX10 (Sigma, HPA032005; RRID: AB_10603560), COA3 (Sigma, HPA031966, RRID: AB_10602115), CMC1 (Sigma, HPA043333; RRID: AB_10797139), COX5B (Santa Cruz, sc-374417; RRID: AB_10988066), SURF1 (Abcam, Ab155251; RRID: AB_2196427), CORE2 (Abcam, ab14745; RRID: AB_2213640), FLAG (Sigma, F3165; RRID: AB_259529), COX20 (Sigma, HPA045490; RRID: AB_10962869), HIGD2A (Sigma, HPA042715; RRID: AB_2678127). Horseradish peroxidase-conjugated anti-mouse or anti-rabbit IgGs were used as secondary antibodies (Molecular Probes; RRID: AB_218457 and RRID: AB_219720). β-ACTIN (Abcam ab8227; RRID: AB_2305186). Anti yest Porin (459500, Thermo Scientific, RRID: AB_2532239)

Abs without assigned RRI:
COX17 (OriGene, TA812124). According to Origene, HEK293T cells were transfected with the pCMV6-ENTRY control or pCMV6-ENTRY COX17 cDNA for 48 hrs and lysed. Equivalent amounts of cell lysates (5 ug per lane) were separated by SDS-PAGE and immunoblotted with anti-COX17 (Cat# TA812124)(1:2000), giving a specific signal of the expected size.
SCO1 and SCO2 (kind gift of P. Rehling, were previously published and validated, and cited them in PMID: 29381136); rabbit anti-yeast-Pet191 (Dilution 1:500; kindly provided by Dr. A. Chacinska and cited in PMID, reported in PMID: 23508107); mouse anti-yeast Porin (Dilution 1:1,000; Thermo Scientific 459500; This Antibody was verified by Cell treatment to ensure that the antibody binds to the antigen stated); and chicken anti-yeast Cox11 (Dilution 1:5,000; gift from Dennis Winge (University of Utah) reported in PMID: 12063264).
All anti-yeast antibodies were tested for reliability using specific KO strains to ensure specificity of detection.

Eukaryotic cell lines

Policy information about cell lines

Cell line source(s)

Human HEK293T embryonic kidney cells (CRL-3216, RRID: CVCL-0063), HEK293 (CRL-1573, RRID: CVCL-0045), and 143B osteosarcoma cells (CRL-8303, RRID: CVCL-2270), were obtained from ATCC. Cybrid cell lines were constructed using enucleated control fibroblasts and the osteosarcoma 143B TK 206 rho zero cell lines (Ref 38). COX1 and COX2 mutant cybrid cells carry a homoplasmic G6930A or G7896A, respectively, mitochondrial mutation that generates a stop codon and a truncated version of the protein (Refs 28, 29). The HEK293T COX16 knock-out (KO) cell line was provided by Dr. Peter Rehling (University Medical Center Göttingen, Göttingen, Germany), and the COA6-KO cell line by Dr. Mike Ryan (Monash University, Melbourne, Australia). The HEK293T COX18-KO and COX20-KO cell lines were previously reported by our group (Refs 11, 39).

To create stable human PET191-KO lines in HEK293T cells, two gene-specific pairs of TALEN constructs were obtained from Thermo-Invitrogen. The pair of left and right TALENs was designed to target the corresponding exon 1. To create stable human COX19-KO and COX11-KO lines in HEK293T cells, we used CRISPR-CAS9 guide RNAs obtained from OriGene (KN210770 and KN203238, respectively) designed to bind the exon 1 region of the corresponding gene.

Yeast strains used in this work were of the W303 genetic background. The cox11Δ, pet191Δ, pet117Δ, coa3Δ, cox11Δcoa3Δ, cox11Δpet191Δ, and cox11Δpet117Δ strains were generated in vivo using homologous recombination of PCR-amplified gene-specific knock-out cassettes containing HIS3, URA3MX, KanMX, or KanMX4 selection markers flanked by DNA sequences with homology to the upstream and downstream chromosomal regions of the coding sequence for the deleted gene. All strains were validated by genotyping.

Authentication

None of the cell lines purchased from ATCC were authenticated in house. ATCC uses morphology, karyotyping, and PCR based approaches to confirm the identity of human cell lines and to rule out both intra- and interspecies contamination. These include an assay to detect species specific variants of the cytochrome c oxidase I gene (COI analysis) to rule out inter-species contamination and short tandem repeat (STR) profiling to distinguish between individual human cell lines and rule out intra-species contamination.

The three KO cell lines constructed here were validated in several ways. Single clones were grown and screened by immunoblotting against PET191, COX11, or COX19 antibodies, and by genotyping. each clone was reconstituted with C-terminal Myc-DDK-tagged wild-type version of the corresponding gene. Analysis of the phenotypes of reconstituted cell lines allowed us to discard of-target effects of the gene-editing approach used in each case.

Mycoplasma contamination

Cell lines are regularly tested for mycoplasma contamination using the Sigma "LookOut® Mycoplasma qPCR Detection Kit" and were confirmed mycoplasma free.

Commonly misidentified lines
(See ICLAC register)

No commonly misidentified cell lines were used in the study.
