# Supplementary material for: Coordination of metal center biogenesis in human cytochrome c oxidase
Source: Nat Commun. 2022 Jun 24;13:3615. doi: 10.1038/s41467-022-31413-1 (PMC9232578; doi:10.1038/s41467-022-31413-1)
Supplement: Supplementary file 4 — Source Data [file 41467_2022_31413_MOESM4_ESM.zip › Souce Data Files/Source Data-Figure Panels.pdf]

Fig 1A

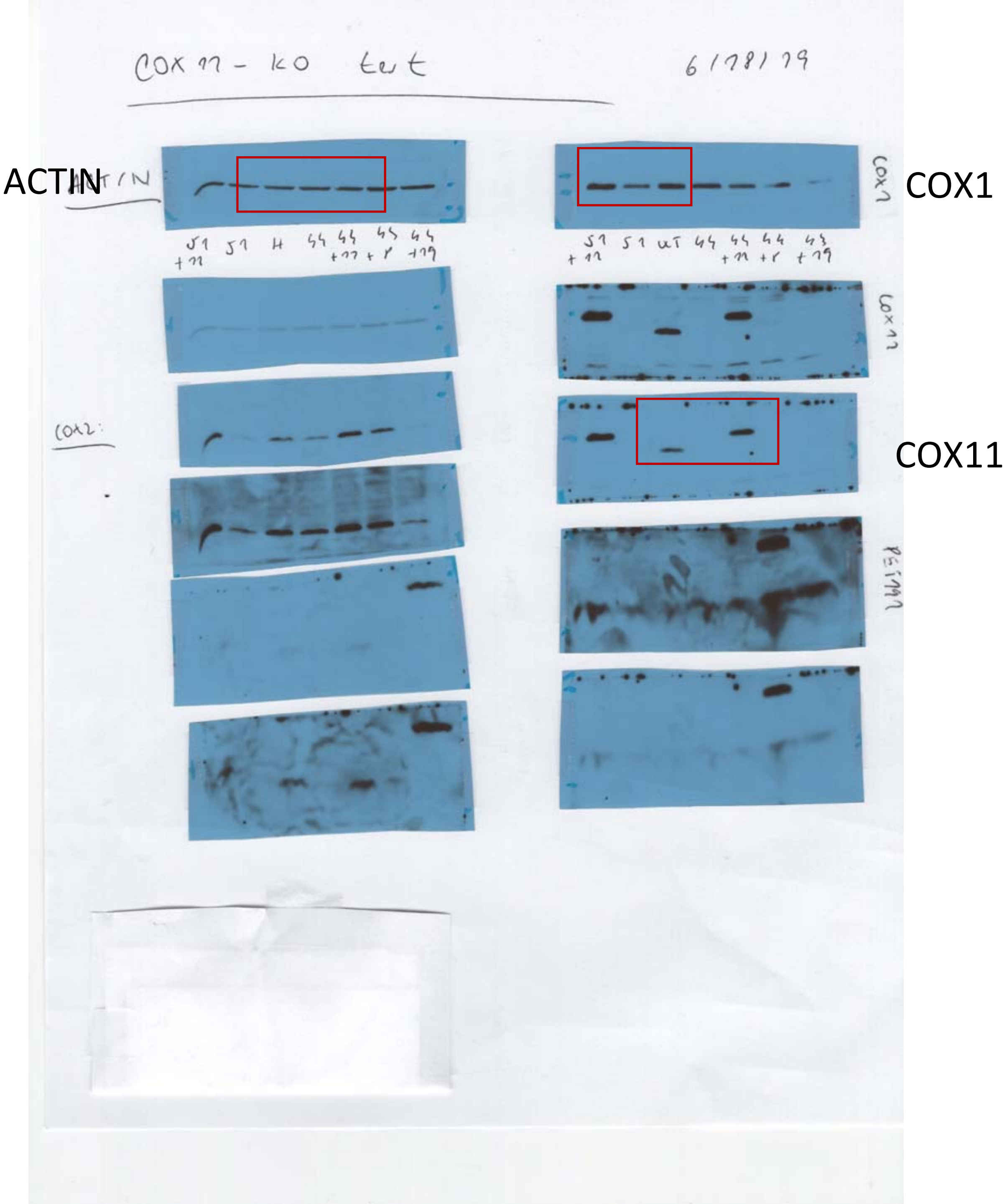

Fig 1A

COX2

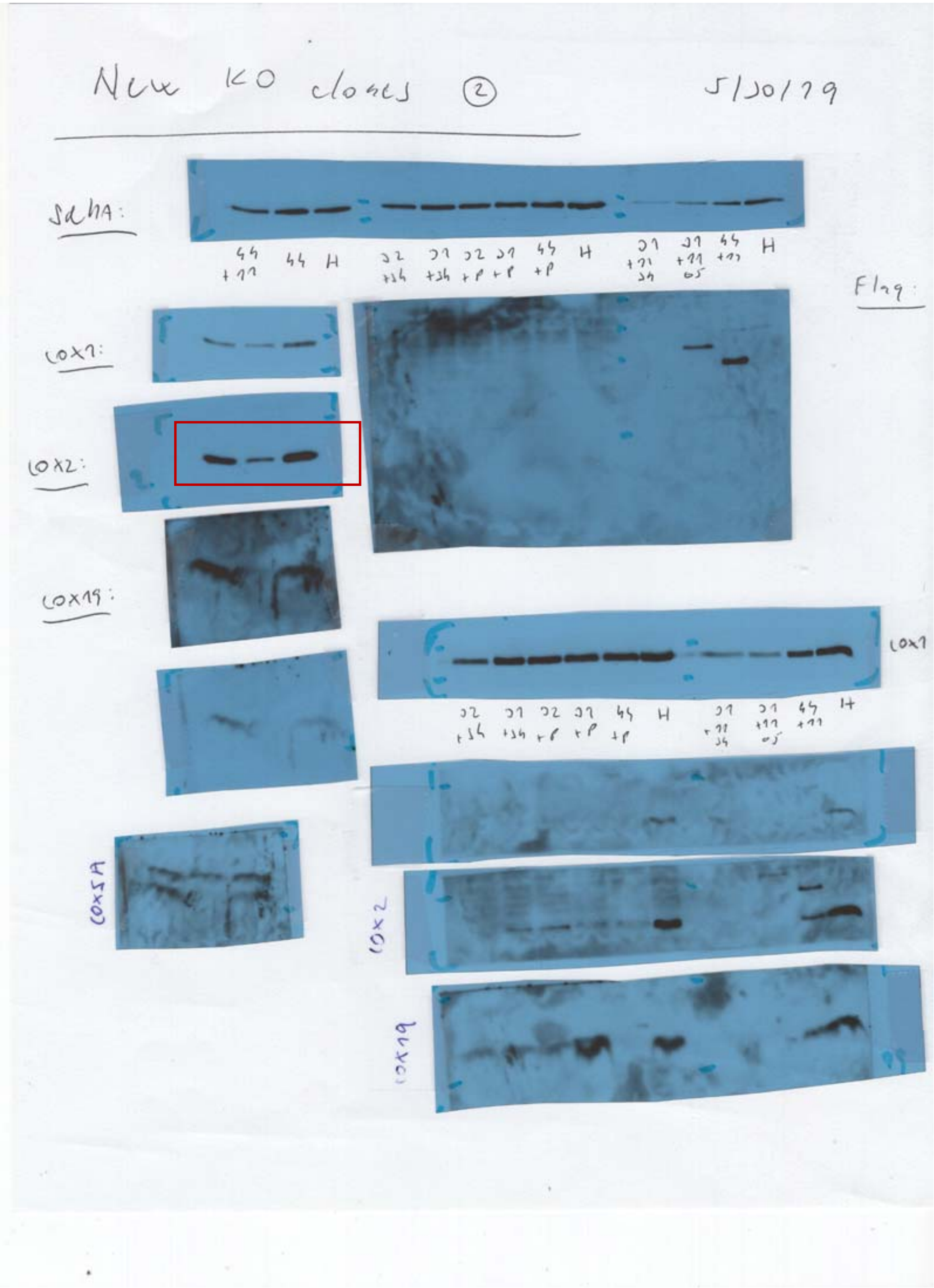
New KO clones ② 5/30/19
Sdha:
Flag:
COX1:
COX2:
COX19:
COX5A
COX2
COX19
COX1

Fig 1A

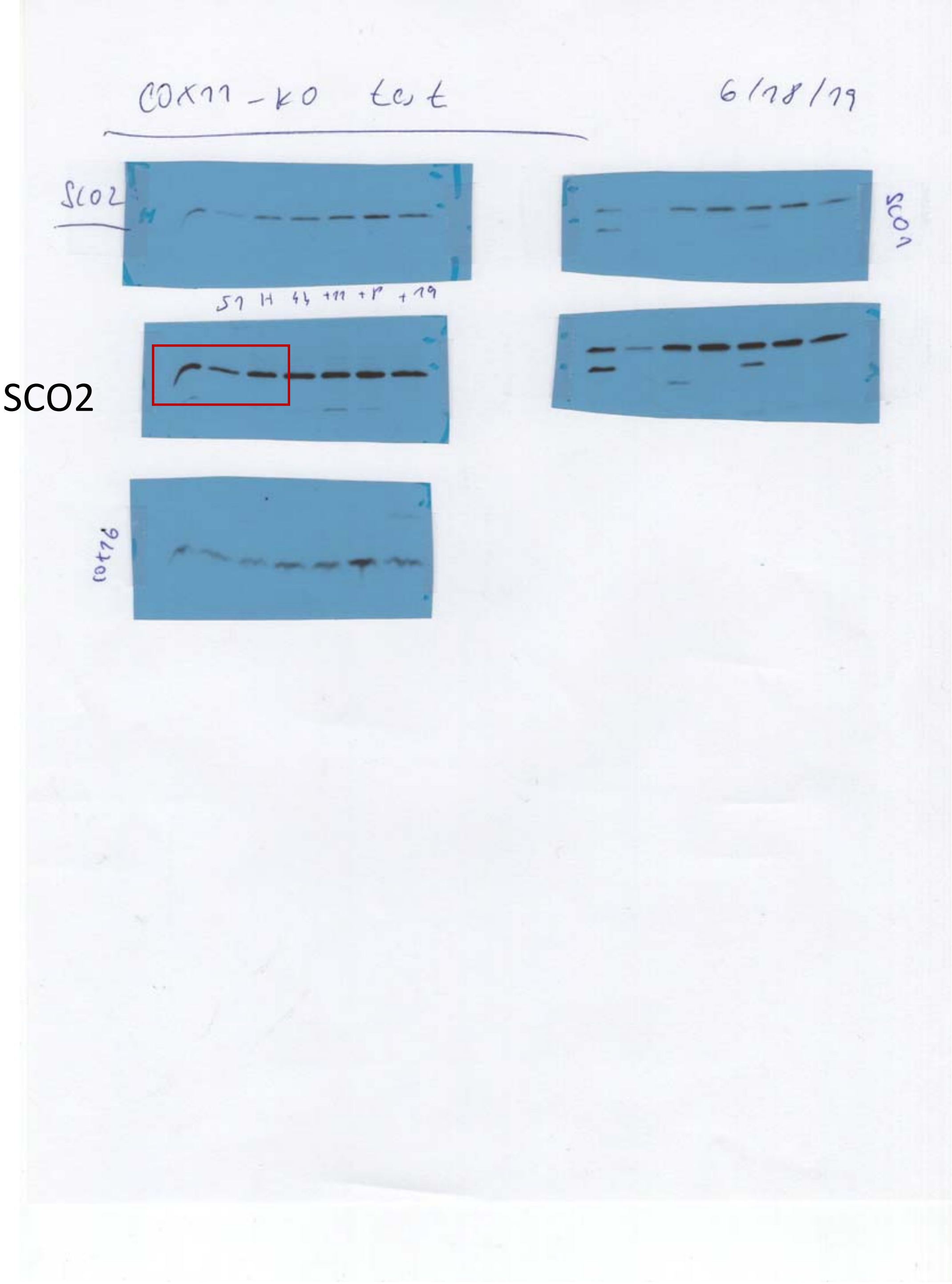

SCO2

Fig 1A

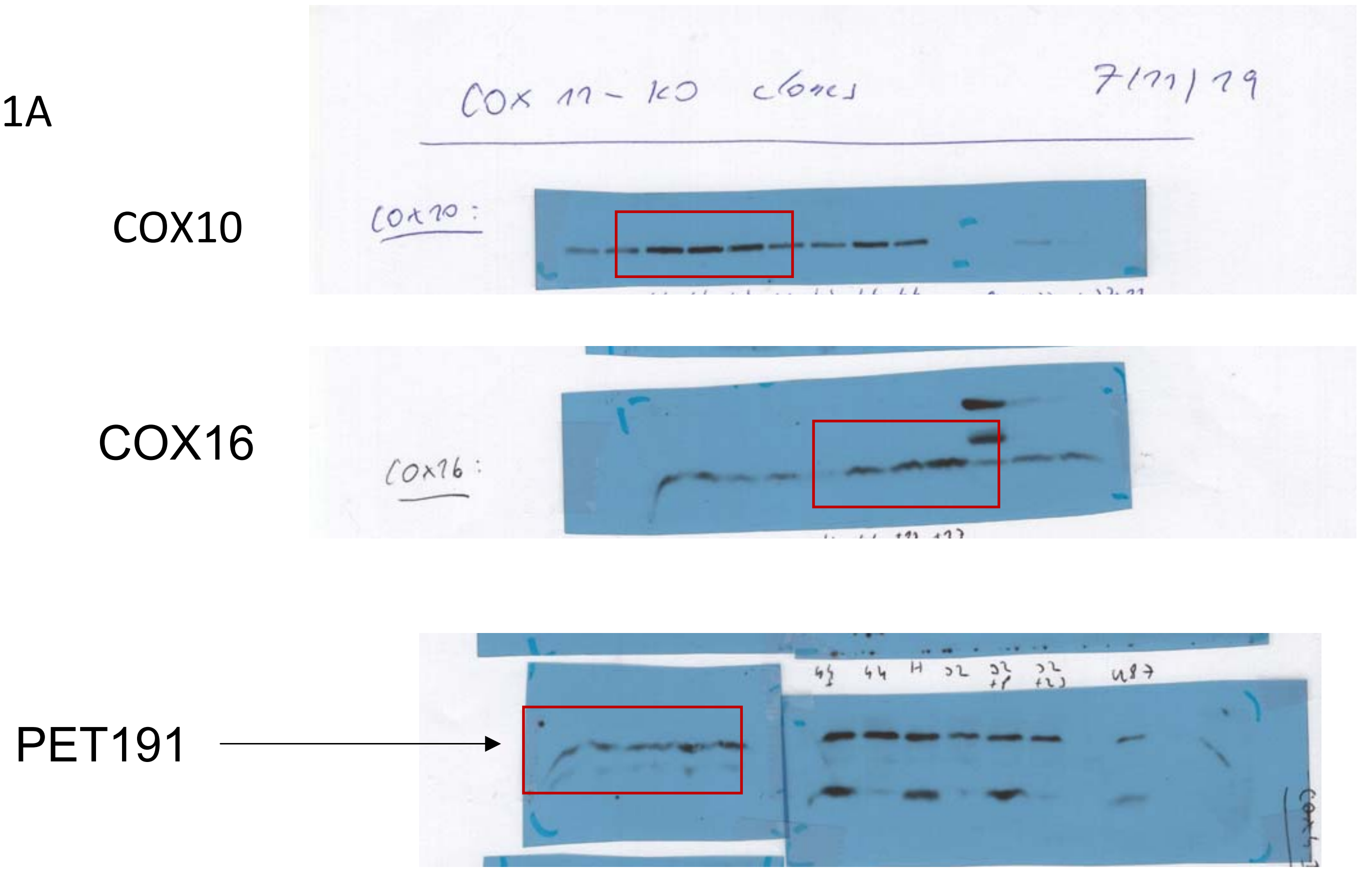

Fig 1A

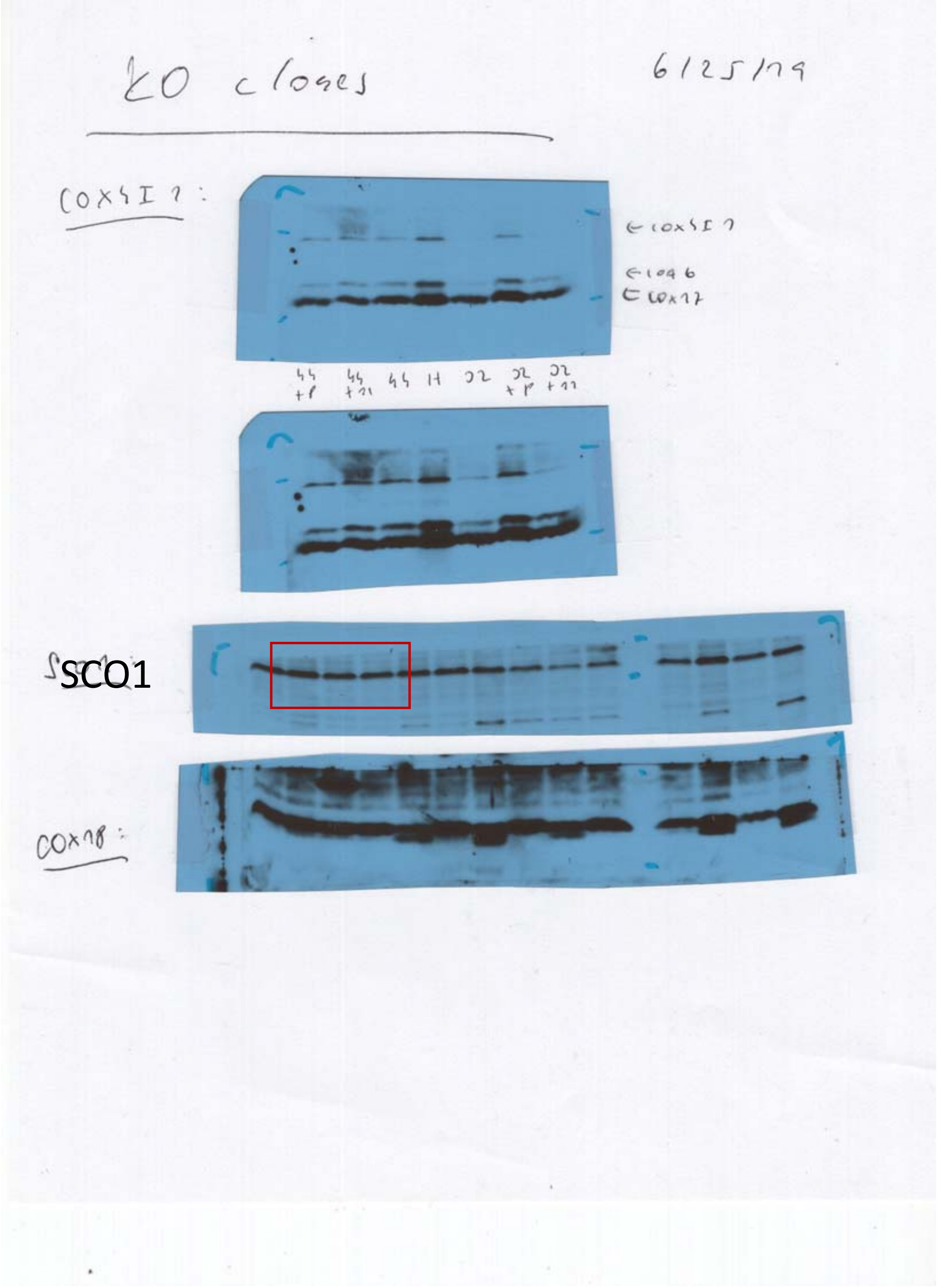

Fig 1A

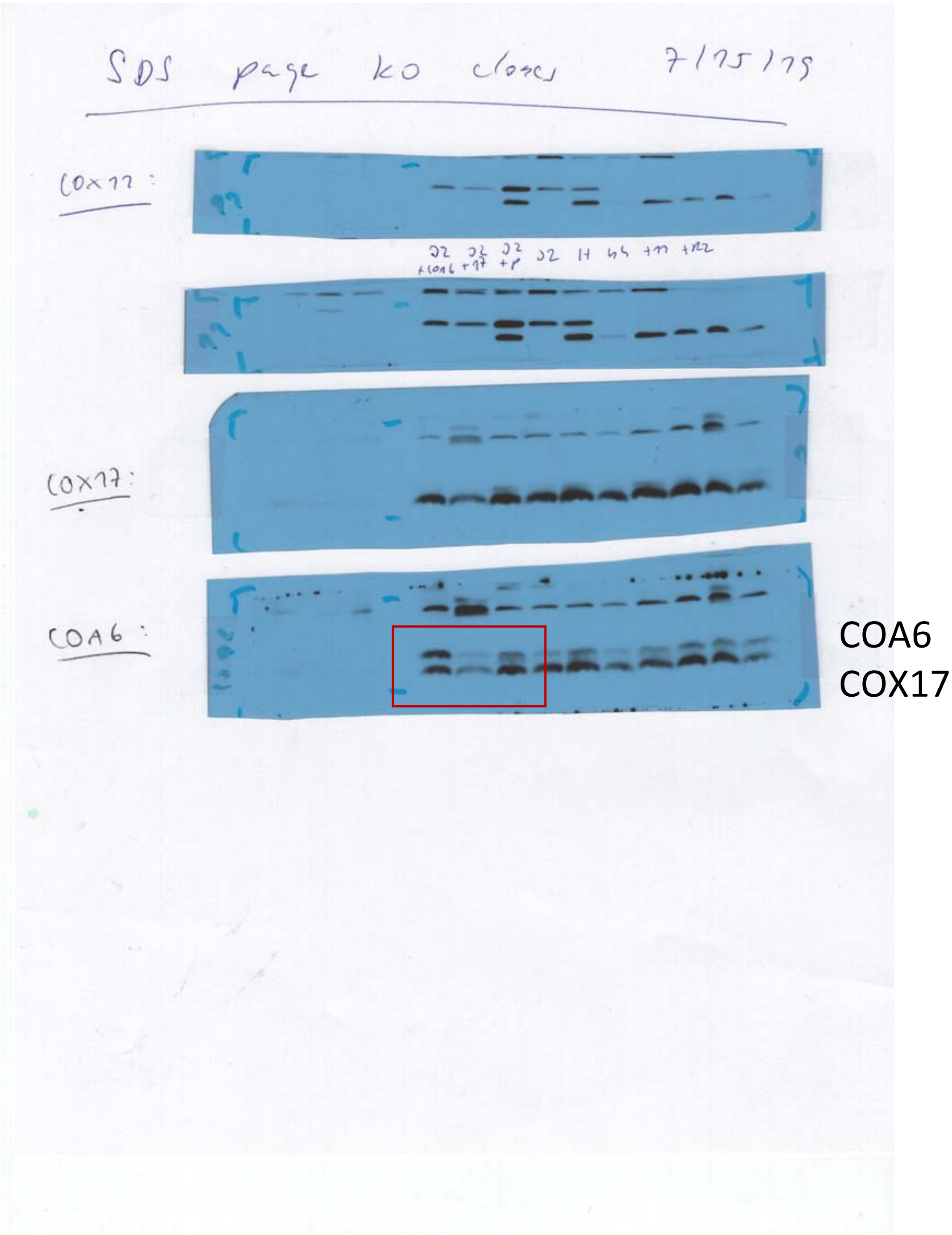

COA6
COX17

Fig 1A

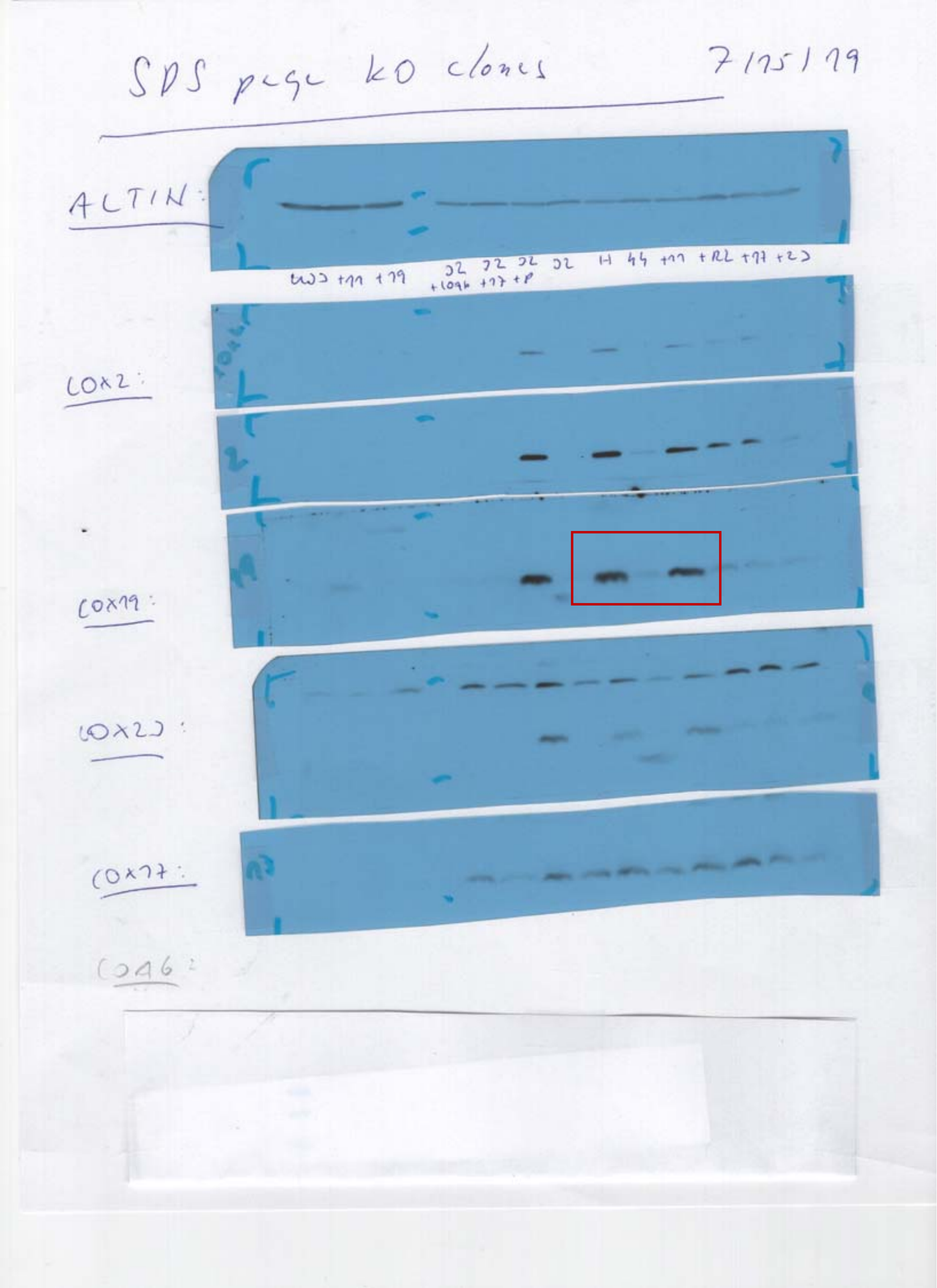

COX19

Fig 1A

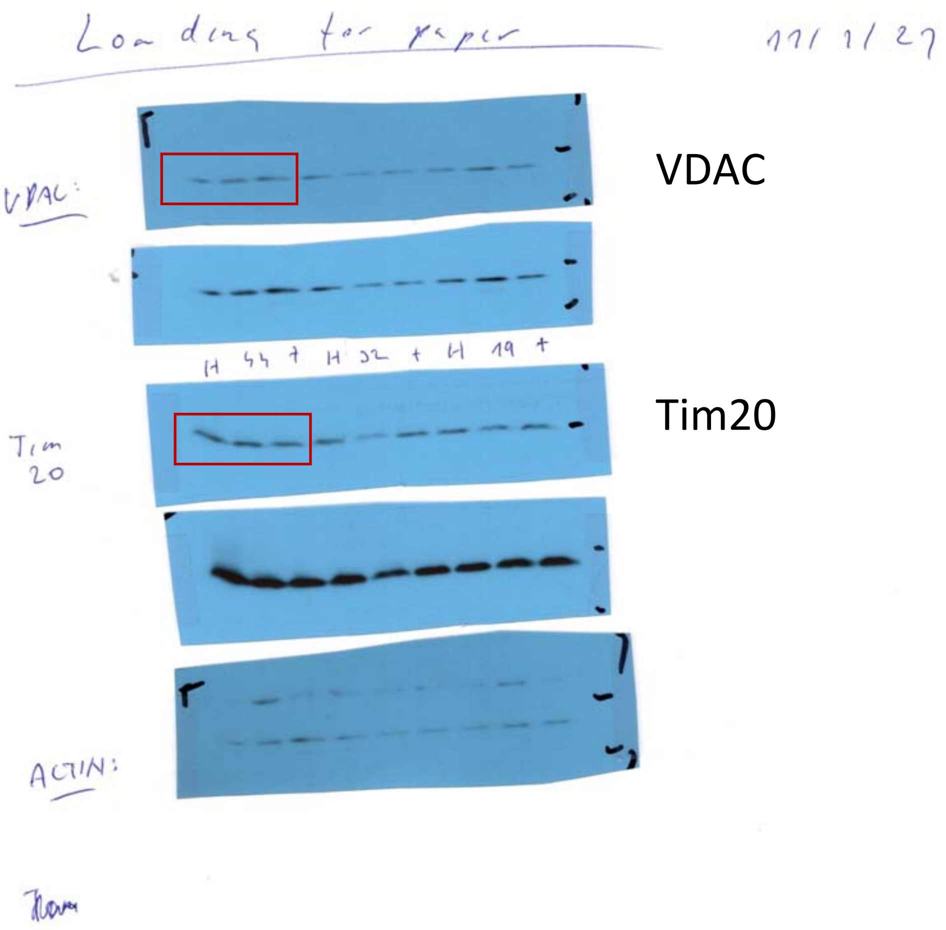

VDAC

Tim20

Fig 1C

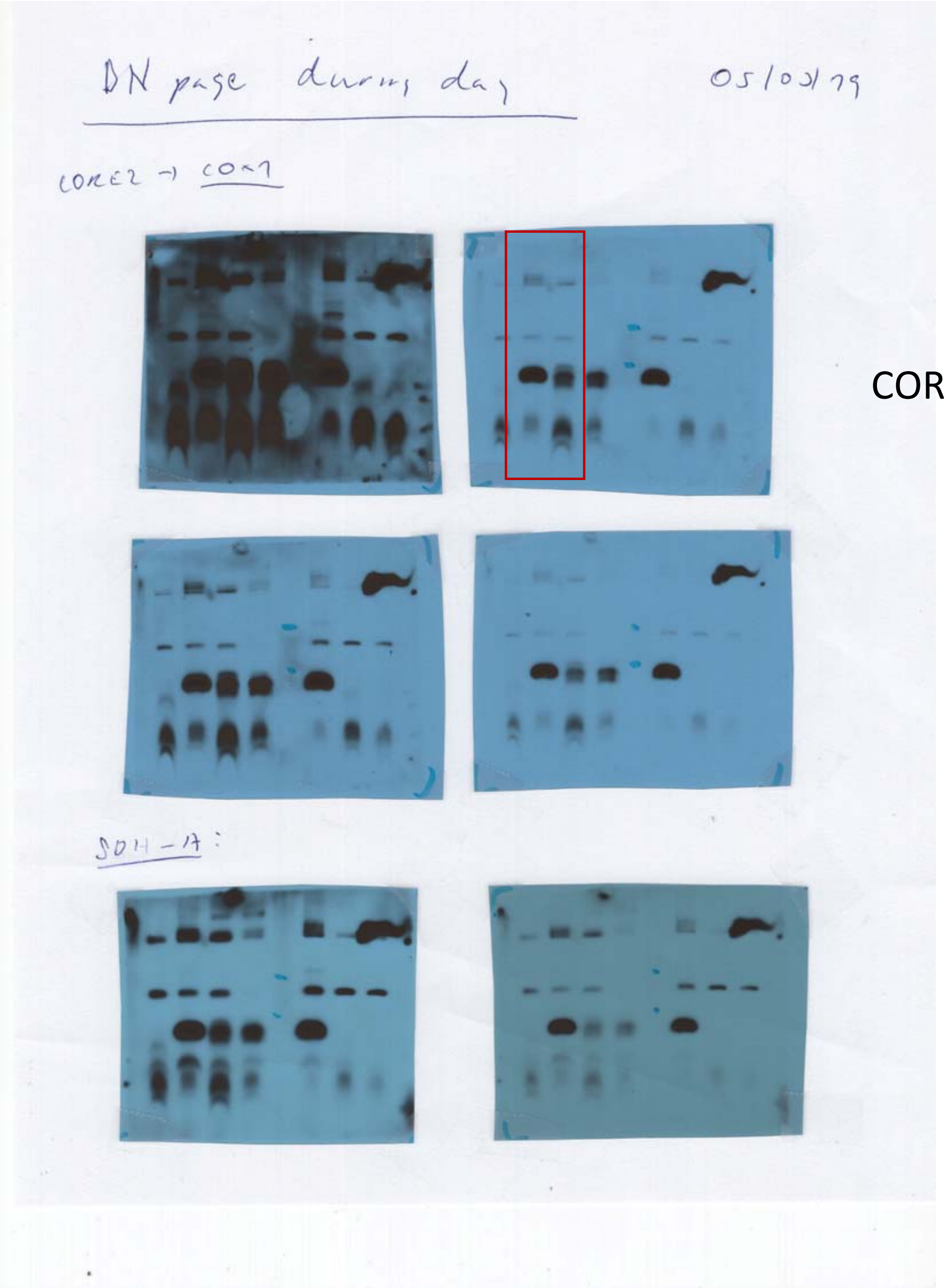

CORE2 + COX1

Fig 1C

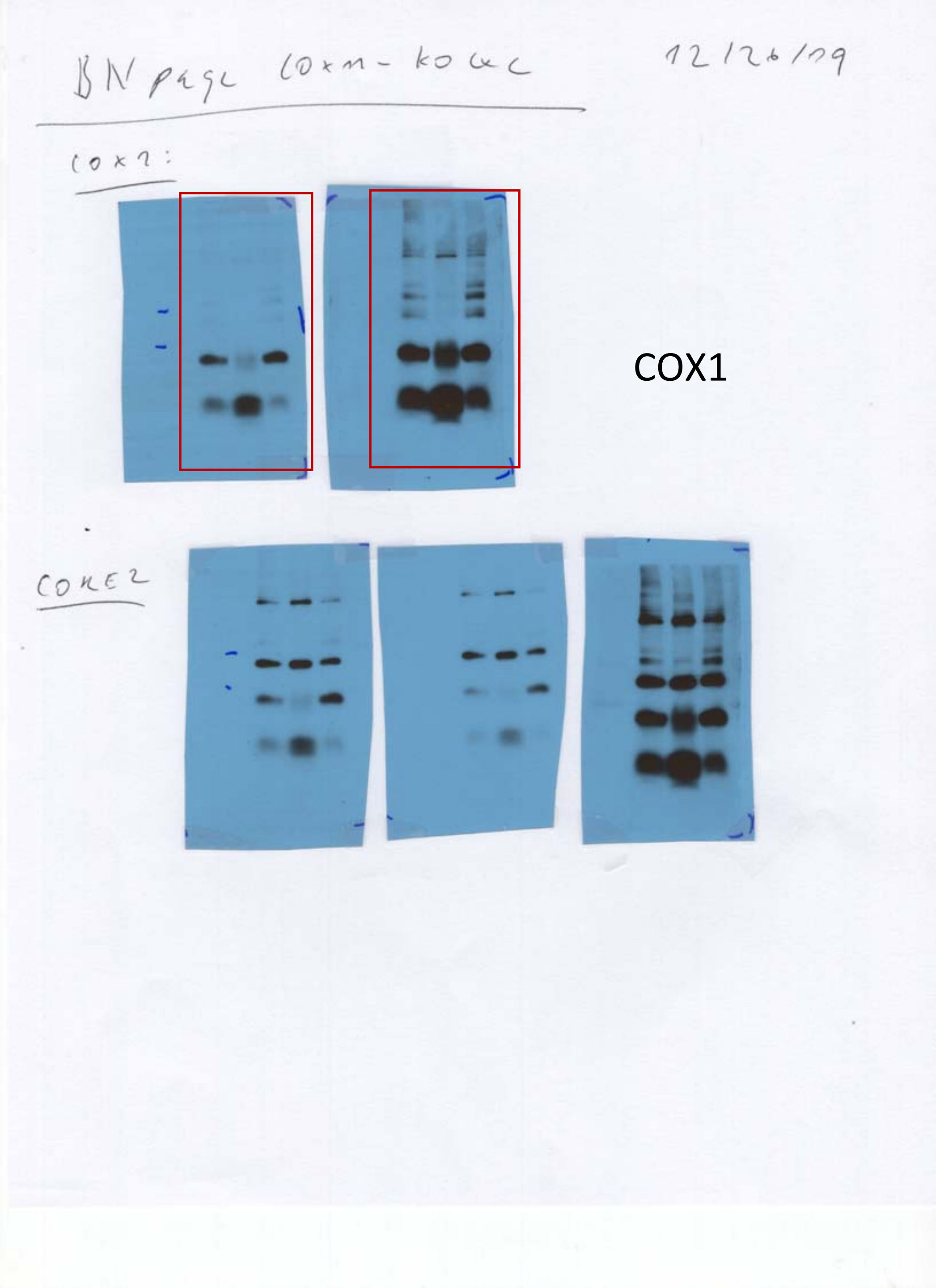

Fig 1K

COX2

ACTIN

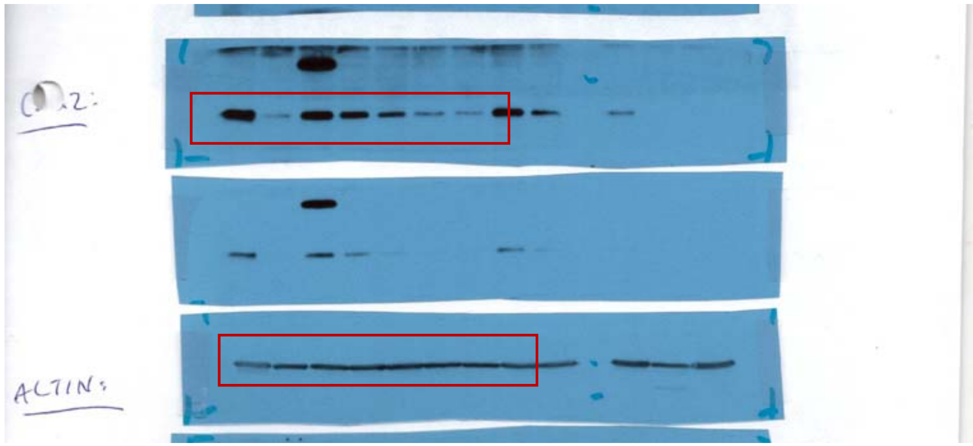

COX17

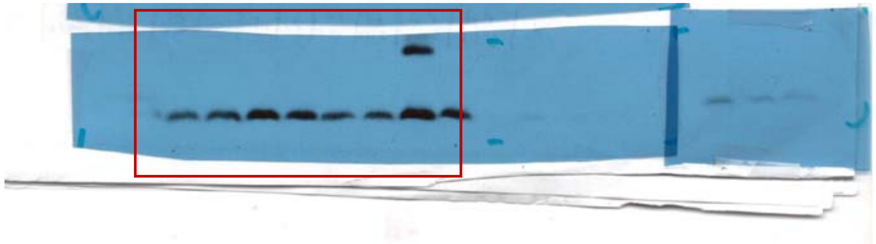

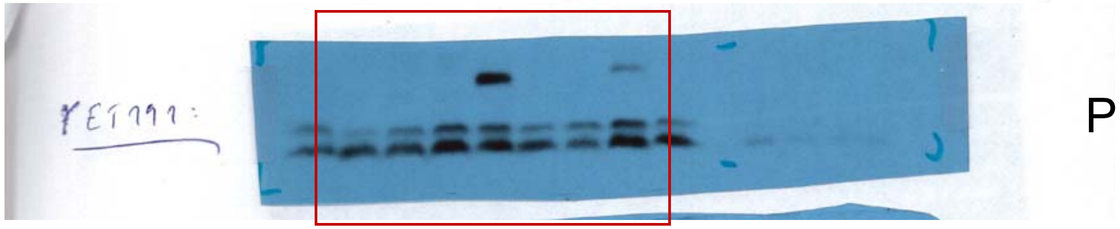

PET191

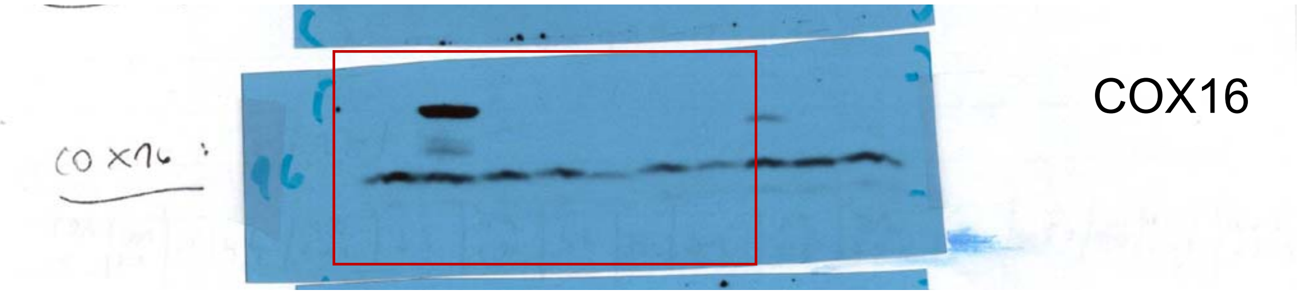

COX16

Fig 1K

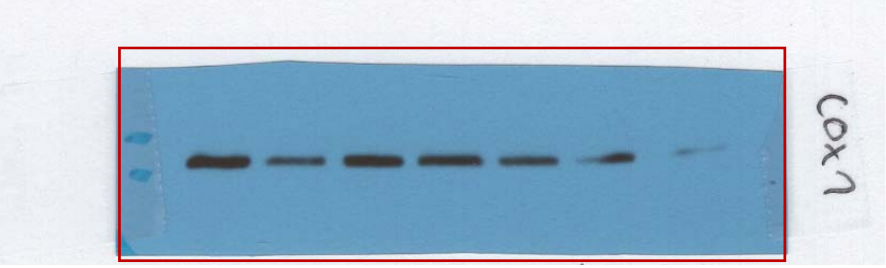

COX1

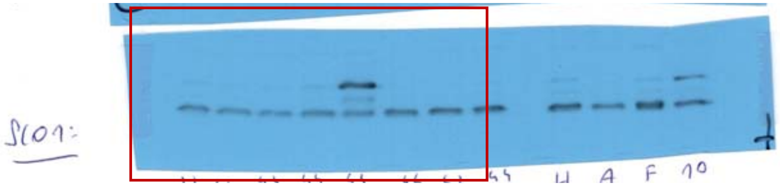

SCO1

Fig3A

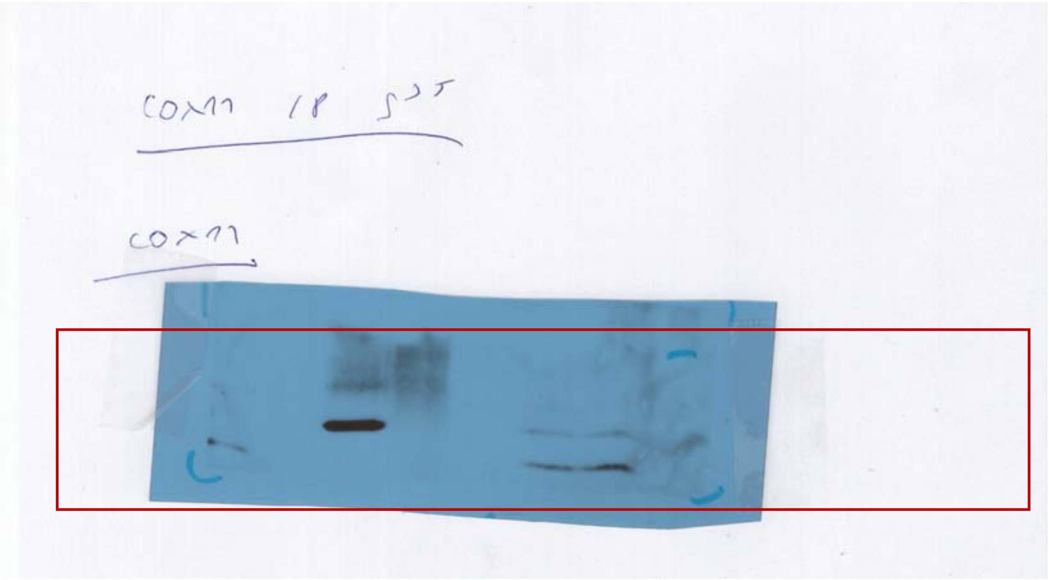

COX11

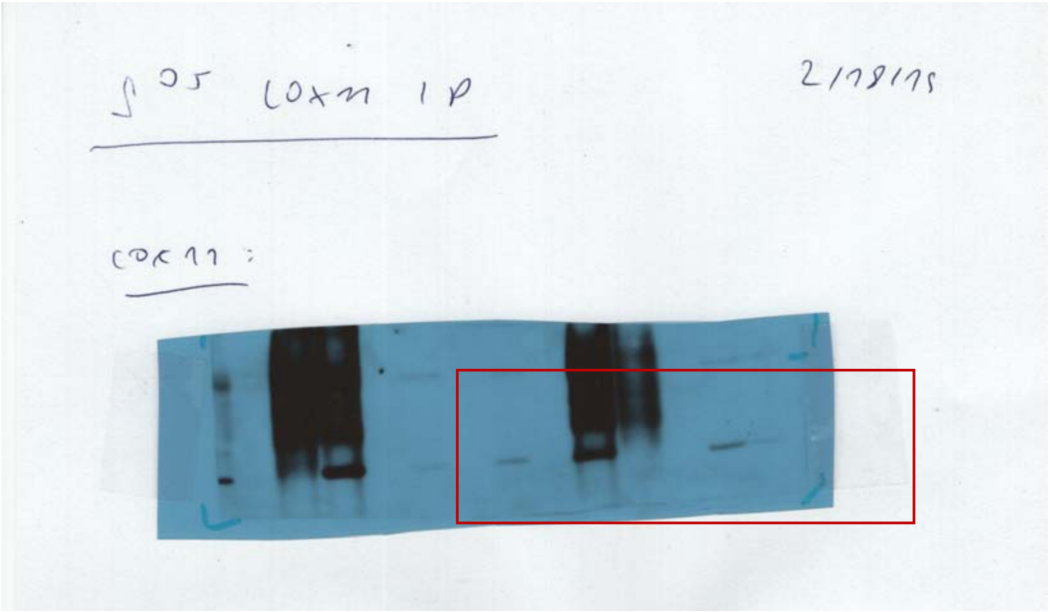

COX11

Fig3A

COX11

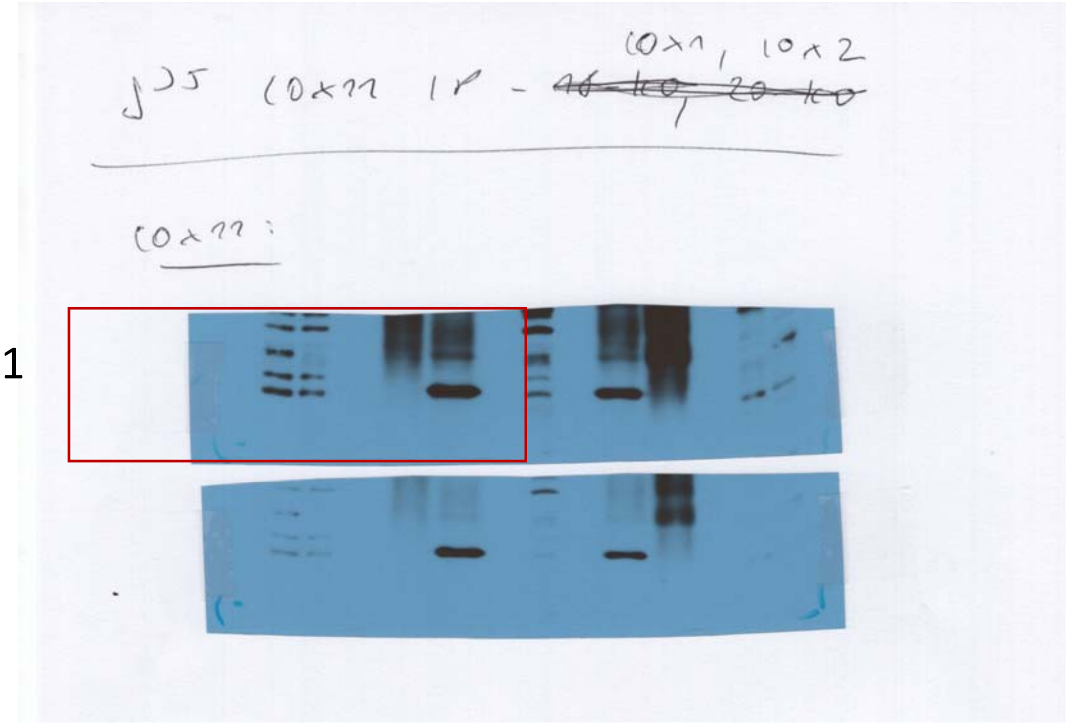

Fig3B

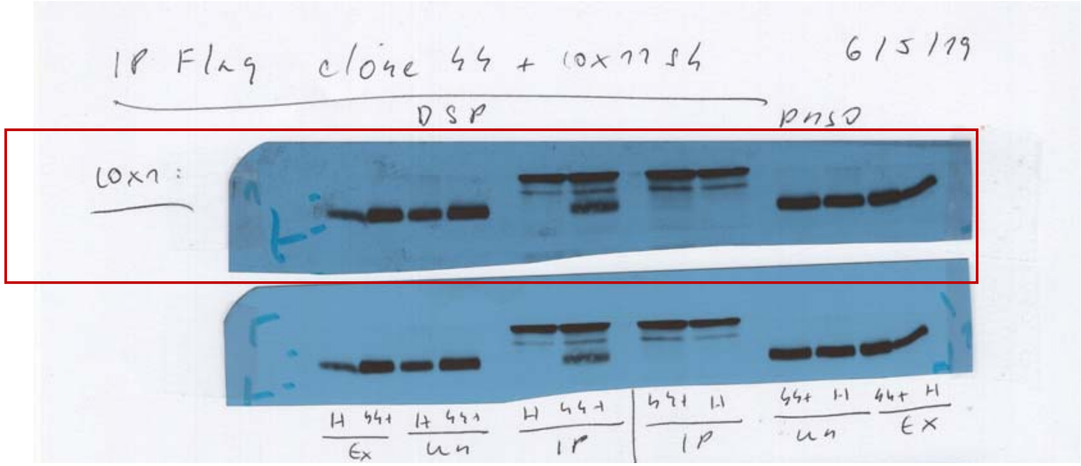

COX1

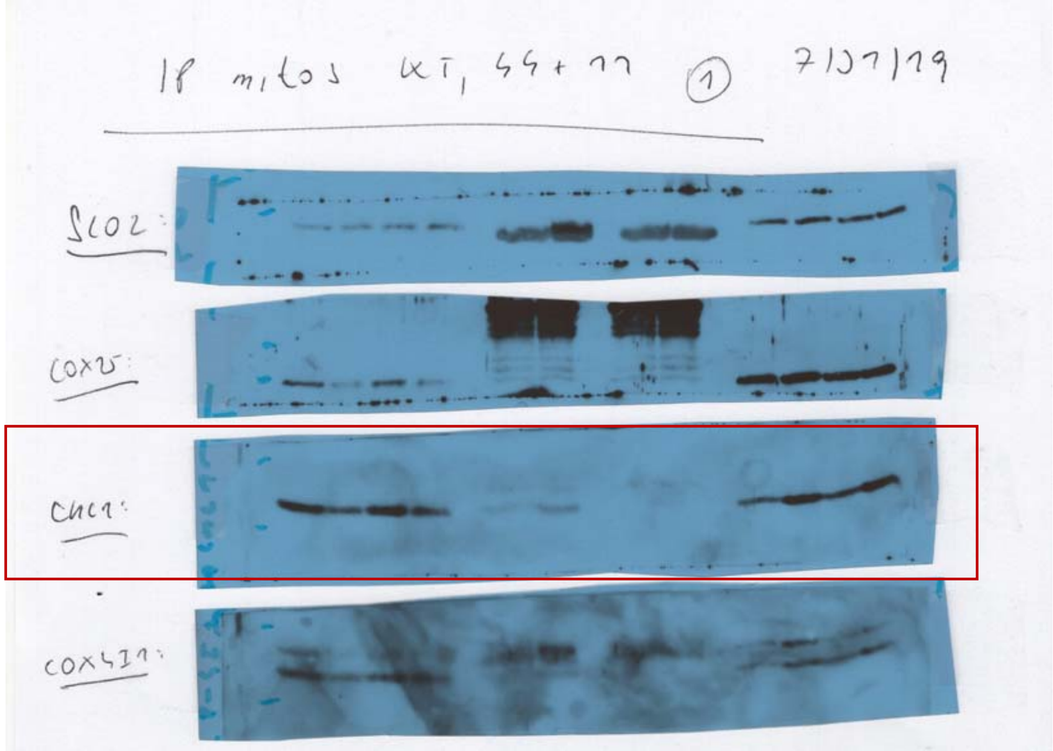

CMC1

Fig3B

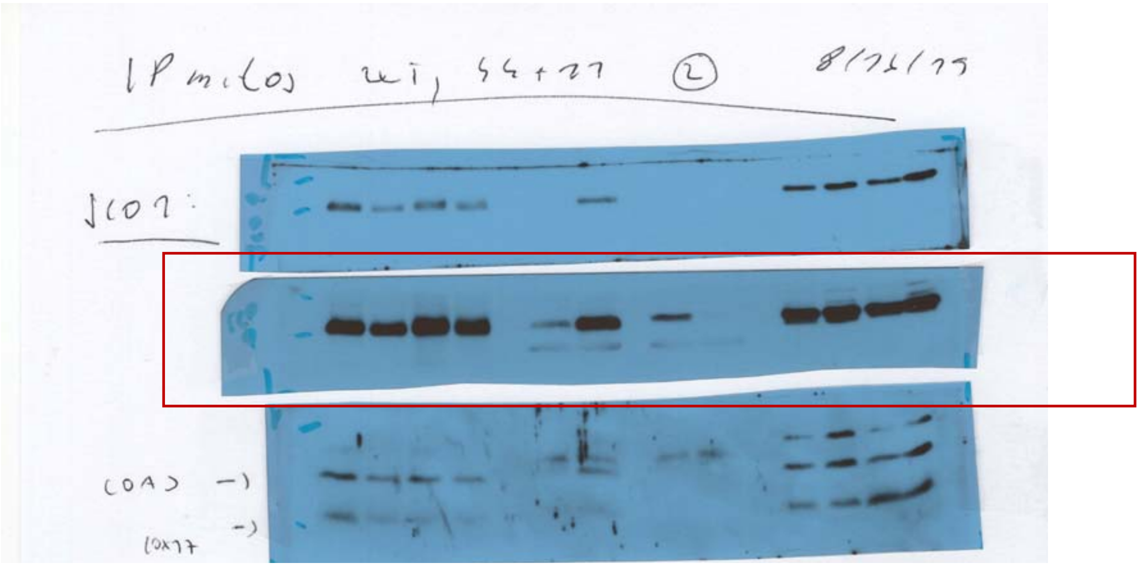

SCO1

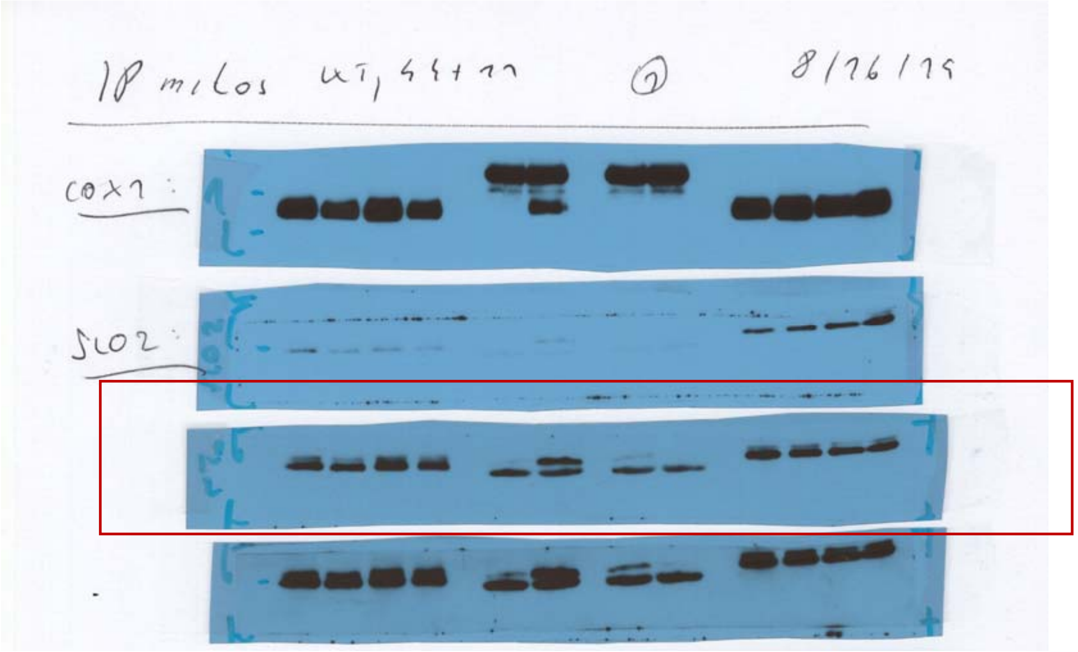

SCO2

Fig3B

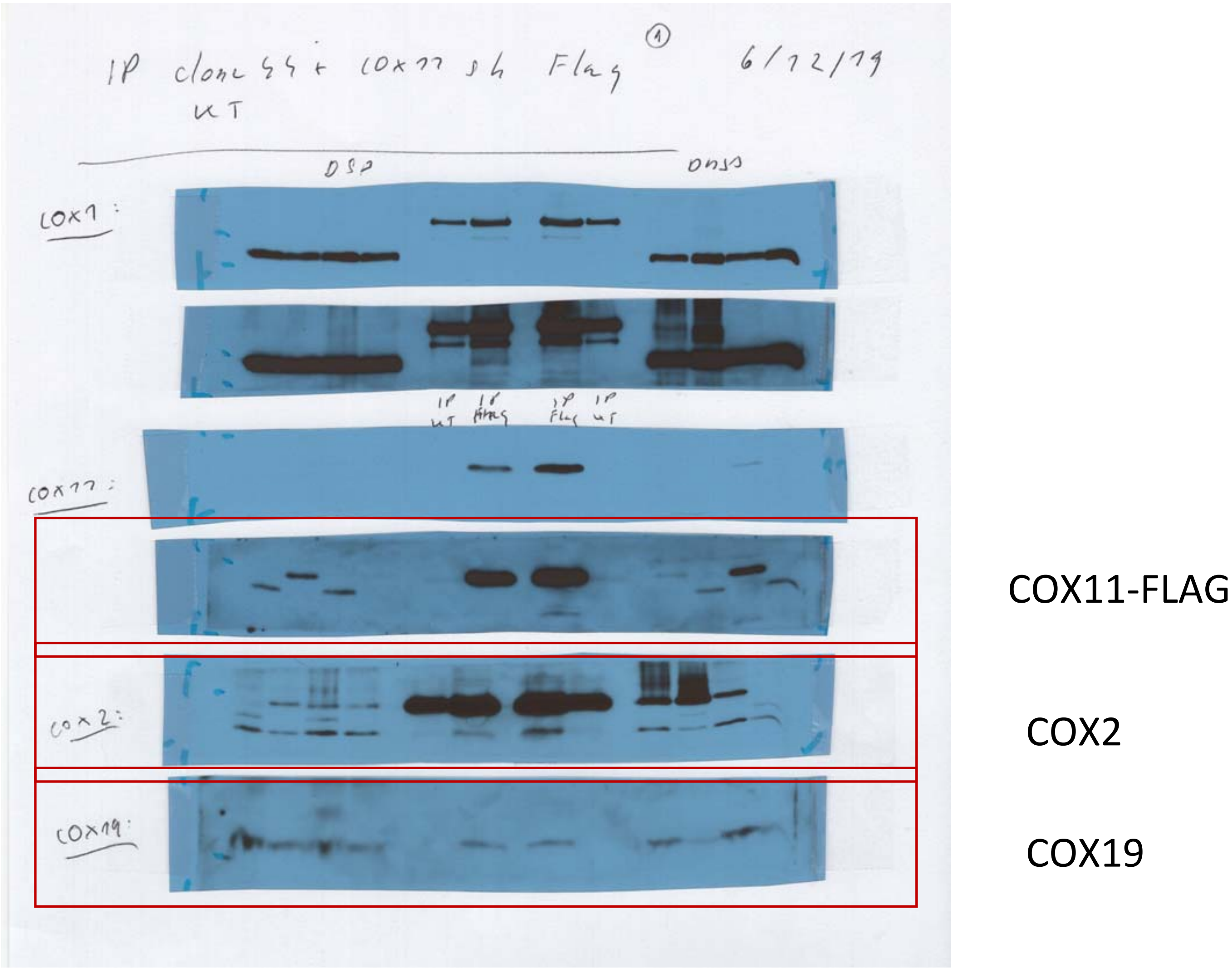

Fig3B

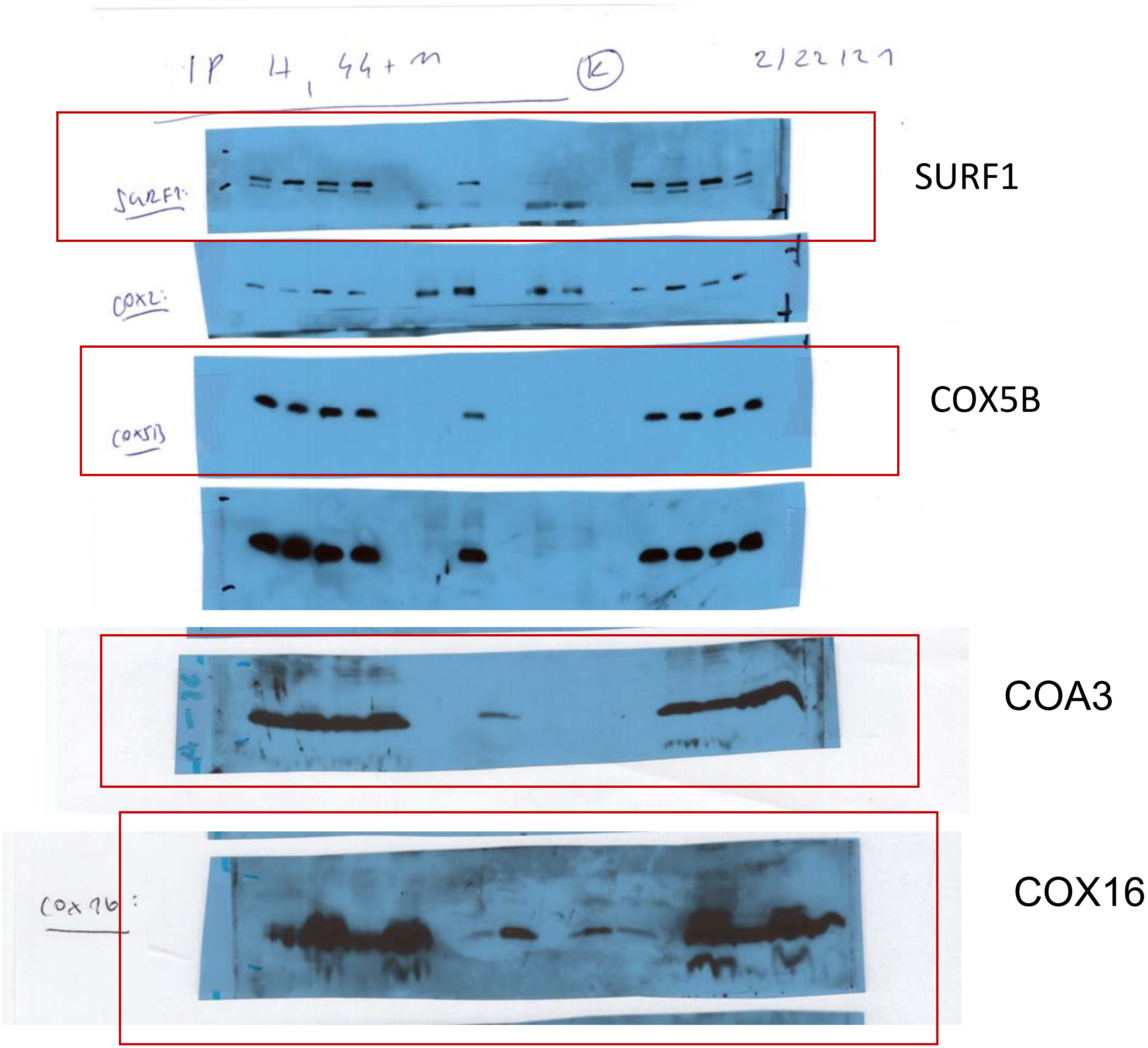

Fig3B

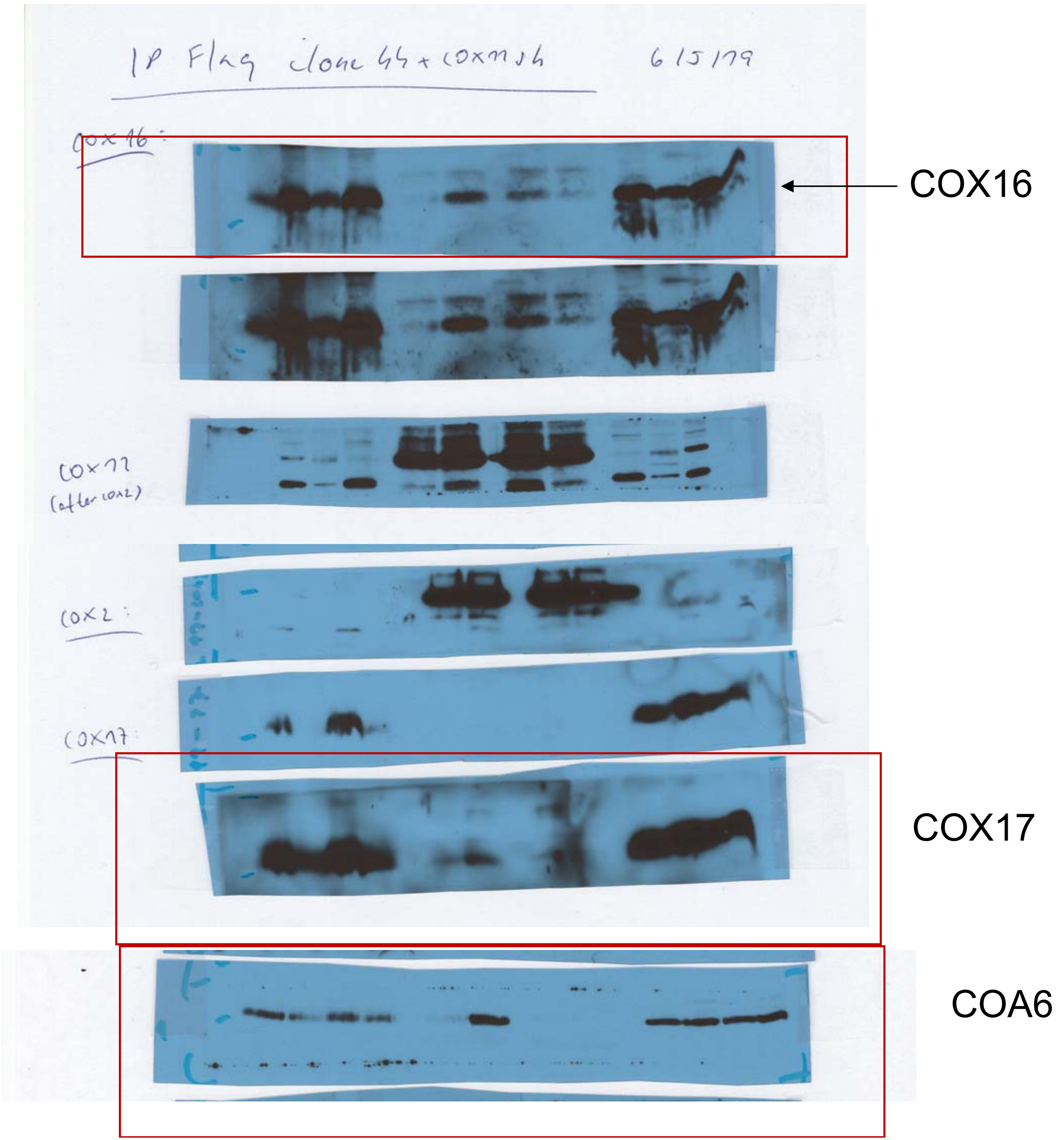
IP Flag clone 44 + COXmyh
6/5/19
COX16
COX16:
COX11
(after COX2)
COX2:
COX17:
COX17
COA6

Fig3B

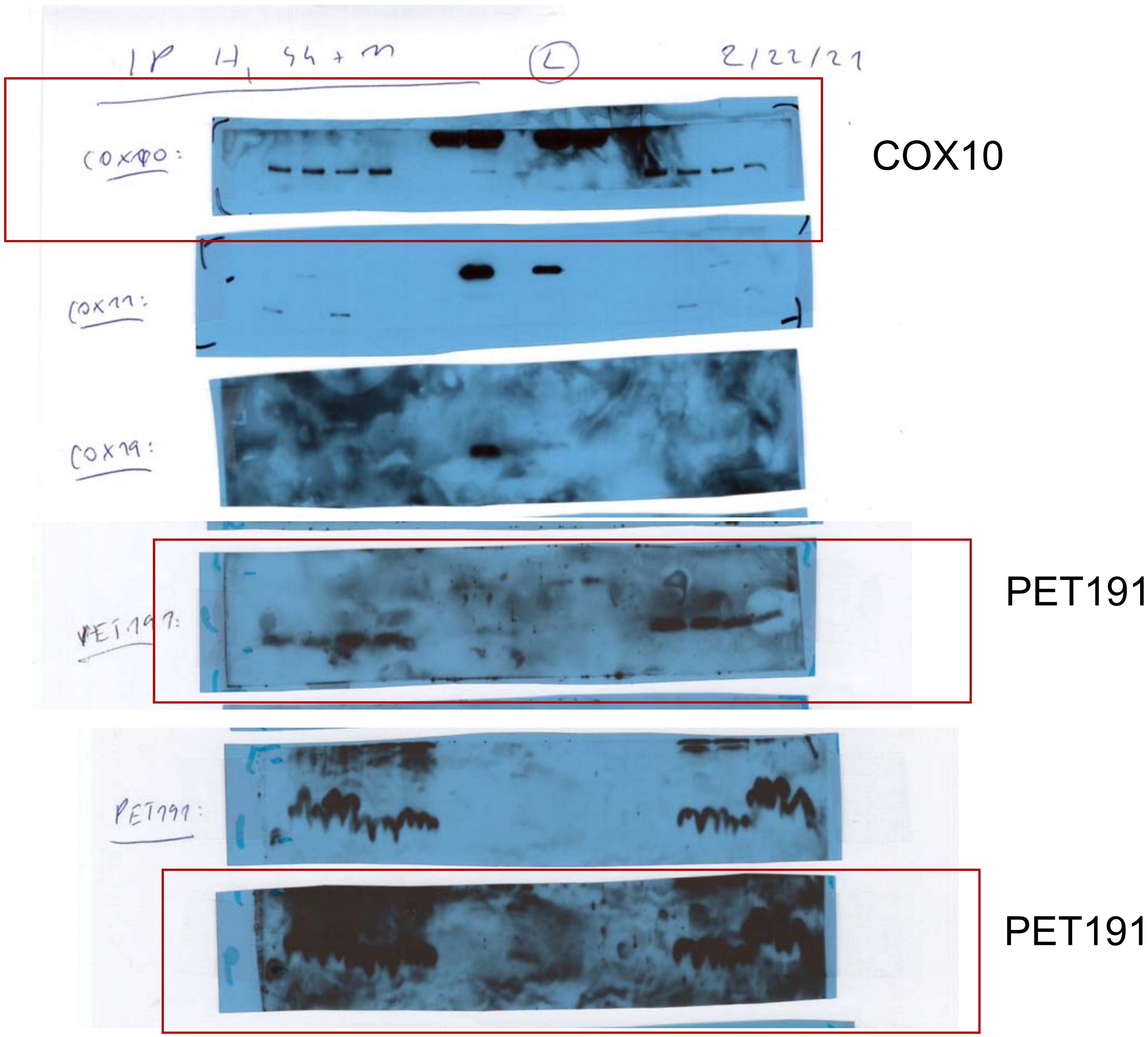

Fig5B

COX10

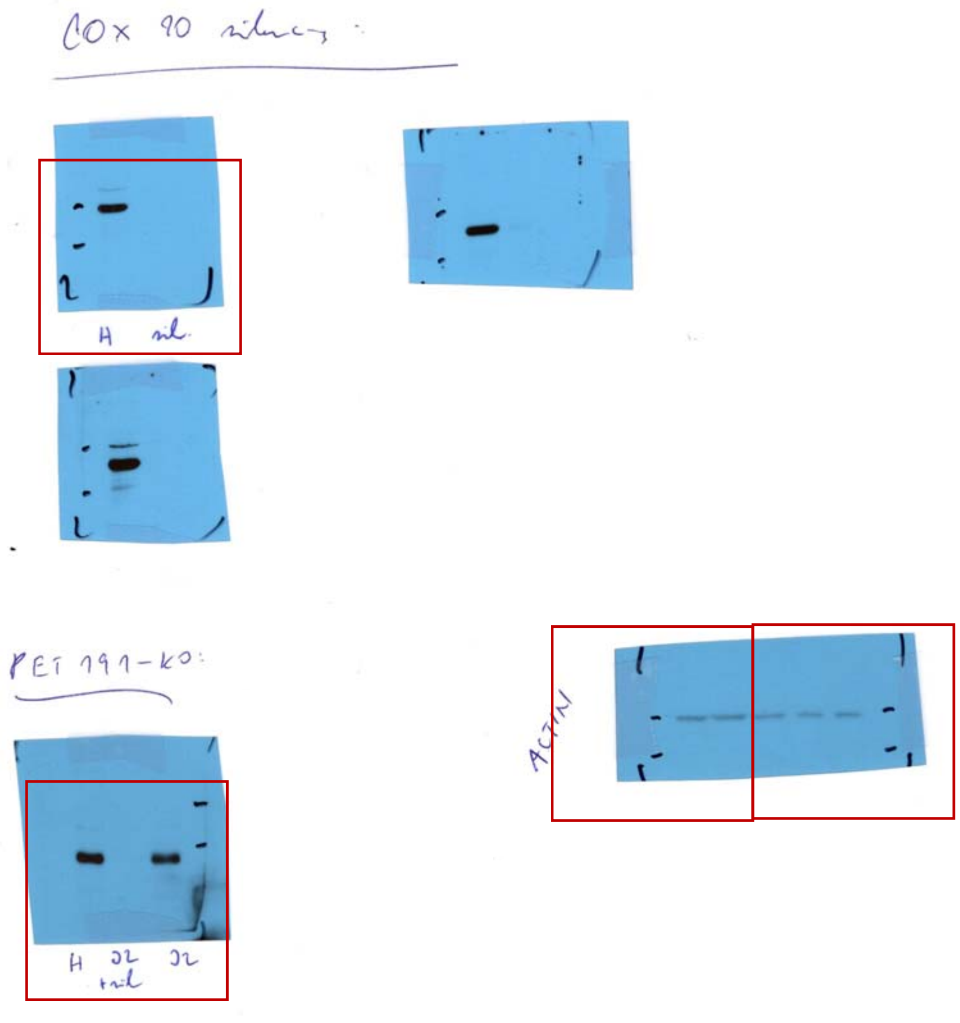

COX10

ACTIN
